# Bacterial Preferences for Specific Soil Particle Size Fractions Revealed by Community Analyses

**DOI:** 10.3389/fmicb.2018.00149

**Published:** 2018-02-23

**Authors:** Michael Hemkemeyer, Anja B. Dohrmann, Bent T. Christensen, Christoph C. Tebbe

**Affiliations:** ^1^Thünen Institute of Biodiversity, Federal Research Institute for Rural Areas, Forestry and Fisheries, Braunschweig, Germany; ^2^Department of Agroecology, Aarhus University, Aarhus, Denmark

**Keywords:** soil particle size fractions, soil DNA, soil archaea, soil bacteria, archaeal diversity, bacterial diversity, 16S rRNA gene amplicon sequencing

## Abstract

Genetic fingerprinting demonstrated in previous studies that differently sized soil particle fractions (PSFs; clay, silt, and sand with particulate organic matter (POM)) harbor microbial communities that differ in structure, functional potentials and sensitivity to environmental conditions. To elucidate whether specific bacterial or archaeal taxa exhibit preference for specific PSFs, we examined the diversity of PCR-amplified 16S rRNA genes by high-throughput sequencing using total DNA extracted from three long-term fertilization variants (unfertilized, fertilized with minerals, and fertilized with animal manure) of an agricultural loamy sand soil and their PSFs. The PSFs were obtained by gentle ultrasonic dispersion, wet sieving, and centrifugation. The abundance of bacterial taxa assigned to operational taxonomic units (OTUs) differed less than 2.7% between unfractionated soil and soil based on combined PSFs. Across the three soil variants, no archaeal OTUs, but many bacterial OTUs, the latter representing 34–56% of all amplicon sequences, showed significant preferences for specific PSFs. The sand-sized fraction with POM was the preferred site for members of *Bacteroidetes* and *Alphaproteobacteria*, while *Gemmatimonadales* preferred coarse silt, *Actinobacteria* and *Nitrosospira* fine silt, and *Planctomycetales* clay. *Firmicutes* were depleted in the sand-sized fraction. In contrast, archaea, which represented 0.8% of all 16S rRNA gene sequences, showed only little preference for specific PSFs. We conclude that differently sized soil particles represent distinct microenvironments that support specific bacterial taxa and that these preferences could strongly contribute to the spatial heterogeneity and bacterial diversity found in soils.

## Introduction

Primary organo-mineral complexes of different sizes (clay <2 μm; fine silt 2–20 μm; coarse silt 20–63 μm; sand 63–2000 μm) constitute the basic building blocks of soil structure and function (Christensen, 2001). While most of the soil organic matter is associated with the finer-sized particles (fine silt and clay), sand fractions typically contain most of the free particulate organic matter (POM) (Christensen, 2001). Soil particle size fractions (PSFs) differ in mineralogical composition (Acosta et al., 2011) as they represent different stages of weathering of primary minerals (Uroz et al., 2009). The characteristic mineralogical compositions cause differences in the surface reactivity and sorption behavior of PSFs, and, as a result, organic matter associated with different PSFs differs in concentration, chemical composition, and decomposability (Christensen, 1992). As a consequence of these properties, PSFs represent different micro-environments in terms of accessible water, nutrients and organic substrates. In turn, the microenvironment can be modified by the activity of microbial communities associated with a given particle surface.

The majority of soil microorganisms lives in close contact with surfaces rather than being freely suspended in soil water (Mills, 2003). Considering their characteristic surface properties and associated microenvironments, different PSFs most likely select for specifically adapted microbial entities in soil. Previous studies have revealed PSF-specific microbial activity, as indicated by measurements of soil enzymes (Stemmer et al., 1998; Kandeler et al., 2000; Marx et al., 2005), nitrogen mineralization, ammonification, nitrification and denitrification (Lensi et al., 1995; Nacro et al., 1996; Christensen and Olesen, 1998), methanogenesis (Zhang et al., 2007; Zheng et al., 2007), and sorption and mineralization of organic pollutants (Botterweck et al., 2014; Hemkemeyer et al., 2015). Furthermore, the abundance of a gene involved in phosphorous-cycling differed between PSFs (Luo et al., 2017). Profiling techniques based on phospholipid analyses (Poll et al., 2003; Zhang et al., 2015) and genetic fingerprinting of PCR-amplified prokaryotic 16S rRNA genes or fungal ITS sequences (Sessitsch et al., 2001; Zhang et al., 2007; Neumann et al., 2013) have provided strong indications that PSFs select for structurally different microbial communities. However, the identity of the microbial taxa exhibiting preferences for specific PSFs and their contribution to the total soil microbial community both have not yet been characterized.

Previous studies with agricultural and artificial soils suggested that the environmental conditions, as represented by different fertilization regimes or mineral compositions affect the bacterial community structure in a PSF-specific manner (Neumann et al., 2013; Hemkemeyer et al., 2014). The objective of the present study was to identify prokaryotic taxa associated with differently sized soil particles regardless of the environmental conditions. Therefore, we analyzed the prokaryotic communities in soil retrieved from the Askov Long-Term Experiment on Animal Manure and Mineral Fertilizers, initiated in 1894 at Askov Experimental Station, Denmark. Each replicate taken originated from a differently treated soil, i.e., one from an unfertilized (UNF), a second from a mineral fertilized (NPK) and a third from an animal manured (AM) field plot (Christensen et al., 2006). The three differently treated soils had a similar pH and a similar content of clay, fine silt, coarse silt and sand sized particles, but different quantities and qualities of soil organic carbon. We applied gentle ultrasonication, wet-sieving and centrifugation to isolate PSFs with most of their cells still attached. This soil fractionation protocol has previously been evaluated for its dispersion efficiency and for its applicability in analyses based on genetic fingerprinting and qPCR (Neumann et al., 2013; Hemkemeyer et al., 2014, 2015). High-throughput Illumina MiSeq sequencing of 16S rRNA gene amplicons was used to characterize the prokaryotic communities and distinguish taxa.

## Materials and methods

### Soil sampling and fractionation

Soil originated from the Askov Long-Term Experiments on Animal Manure and Mineral Fertilizers in Denmark (55°28.3′N, 09°06.7′E) (Christensen et al., 2006). The site is a Typic Hapludalf and loamy sand consisting of 11% clay (<2 μm) and 13% fine silt (2–20 μm). The coarser fractions are dominated by quartz and feldspars, while the main clay minerals are illite, smectite, and kaolinite. We sampled field plots kept unfertilized (UNF plot 124), receiving mineral fertilizer (100 kg N, 19 kg P and 87 kg K ha^−1^ annually; NPK plot 125), or receiving animal manure (37.5 t wet weight cattle slurry corresponding to 143 kg total-N, 30 kg P and 134 kg K ha^−1^ annually; AM plot 116). Sampling took place after harvest of a grass-clover ley used for cutting and 18 months after the last fertilizer applications. Twenty-five soil cores of each plot from a depth of 0–18 cm (Ap horizon) were sampled, pooled, and slowly air-dried in the laboratory for 6 h to allow sieving (mesh size 2 mm). The sieved samples were stored at 50–55% water holding capacity in the dark at 4°C for 9 months. Soil organic carbon content was 15.8, 18.3, and 22.5 mg g^−1^ dry soil for UNF, NPK, and AM, respectively, while pH was 6.2–6.5 (Hemkemeyer et al., 2015).

Each of these three fertilization treatments were fractionated with three technical replicates as previously described (Hemkemeyer et al., 2015) using the protocol of Amelung et al. (1998) as modified by Neumann et al. (2013). To disperse the aggregates with minimal detachment of microorganisms, the soil was suspended in distilled water at a ratio of 1:5 and treated ultrasonically with a Sonoplus HD 2200 homogeniser (Bandelin electronic, Berlin, Germany). The tip of the Sonotrode (Model VS 70T) was immersed 20 mm into the soil suspension (total instrument output of 70 W) and produced a low energy intensity of 30 J mL^−1^. The sand-sized fraction was isolated by wet-sieving using a mesh size of 63 μm. The <63 μm fraction was centrifuged at 25 × *g* twice for 15, 13, 12, and 11 min, respectively. After each centrifugation step, the supernatant containing the clay fraction was collected and the pellets then re-suspended. A MgCl_2_ solution was added to the supernatants containing clay to reach a final concentration of 3.3 mM and then clay was left to sediment overnight at 4°C. As the addition of MgCl_2_ did not accomplish a complete precipitation of clay, the precipitate and the remaining suspension were centrifuged at 2,450 × *g* for 10 min at room temperature and supernatant was decanted. The silt fraction was further separated into coarse and fine silt by wet-sieving using a mesh size of 20 μm and gravity sedimentation of the fine silt fraction. Overall, based on the replicates indicated above, the fractionations yielded a total of 45 samples, including for each replicate four PSFs and a non-fractionated soil sample.

### DNA extraction and quantification of 16S rRNA genes

DNA was extracted from 0.5 g fresh weight of unfractionated soil and from each of the three replicates of the above mentioned size-fractions. The material from the fraction 63–2000 μm was directly taken from the sieve. The fractions 20–63 and 2–20 μm were suspended in distilled water at a ratio of 1:5, the fraction <2 μm at a ratio of 1:18. A total of 1.8 mL of the suspensions was transferred to 2 mL extraction tubes derived from the extraction kit (see below). After centrifugation at 12,700 × *g* for 5 min, supernatant was removed by pipetting. Two further aliquots were used to determine the dry mass. The material used for DNA extraction corresponded to a dry weight of about 0.5 g of the sand-sized fractions, 0.4 g of each silt fraction, and about 0.1 g of the clay fraction.

The DNA extraction was conducted with the FastDNA™ SPIN Kit for Soil using the FastPrep®-24 Instrument (both MP Biomedicals, Santa Ana, USA) according to the manufacturer's instructions with minor modifications as described elsewhere (Hemkemeyer et al., 2015). The DNA bound to the binding matrix of the FastDNA™ SPIN Kit was washed twice with 1 mL 5.5 M guanidine thiocyanate (Carl Roth, Karlsruhe, Germany) to remove co-extracted contaminants.

To estimate abundances, partial 16S rRNA genes were quantified from the extracted DNA solutions by qPCR using the StepOnePlus™ Real-Time PCR System (Life Technologies/Thermo Fisher Scientific, Carlsbad, CA). Amplification was conducted using Maxima Probe qPCR Master Mix (2x) including ROX solution (Thermo Fisher Scientific, Waltham, MA). A total volume of 20 μl contained 500 nM of each primer and 200 nM of the probe, i.e. BAC338F, BAC805R, and BAC516F for bacteria and ARC787F, ARC1059R, and ARC915F for archaea (Yu et al., 2005). For all samples, 2 μl of a 10-fold dilution of soil DNA were used as template. For measurement of the 16S rRNA genes from the clay-sized fraction, 2 μl of a 50-fold dilution were used as template. The PCR reaction started with an initial denaturing step of 95°C for 10 min, followed by 45 cycles at 95°C for 15 s and 60°C for 1 min. DNA from pure cultures of *Bacillus subtilis* and *Methanobacterium oryzae*, respectively, were used creating the standard curves. The PCR efficiency Eff was calculated as follows: Eff = (−1+10^−1/slope of the standard curve^) x 100%. For bacteria from unfractionated soil Eff was 96.9% (*R*^2^ = 0.999) and from PSFs 92.8% (*R*^2^ = 0.999), for archaea it was 88.0% (*R*^2^ = 0.999) and 89.2% (*R*^2^ = 0.999), respectively.

### Illumina library generation

Sequencing was done following the dual-indexing approach of Kozich et al. (2013). To avoid interference of the appendices of the primers with genomic DNA, a nested PCR approach was used (Berry et al., 2011). The first PCR reaction amplifying the 16S rRNA gene was conducted separately for each domain using primers F27 (Lane, 1991) and 926r (Liu et al., 1997) for bacteria and A364aF (Burggraf et al., 1997) and A934bR (Grosskopf et al., 1998) for archaea, respectively. PCR conditions and subsequent purification have been described elsewhere (Hemkemeyer et al., 2015). In the second step, the V4 region was amplified using the primers S-D-ARCH-0519-a-S-15 and S-D-Bact-0785-a-A-21 (Klindworth et al., 2013). The primers used in this study contained adapters specifically to attach to the Illumina flow cell, a 8-bp-index allowing for multiplexing, a 10-bp-pad to adjust all primer combinations to approximately the same melting temperature of 65°C, and a 2-bp-sequence that linked the appendix to the proper primer and that was anti-complementary to known regions flanking the V4 region. For multiplexing 4 forward and 12 reverse primers were combined by dual-indexing to differentiate 48 samples (Table S1). The PCR was carried out in 50 μL volumes containing 10x FastStart High Fidelity Reaction Buffer (including 1.8 mM MgCl_2_), 200 μM of each dNTP (both Roche Diagnostics, Mannheim, Germany), 0.4 μM of each primer, 5% (v/v) dimethyl sulfoxide, 2.5 U FastStart High Fidelity Enzyme Blend (both Roche Diagnostics), and 2 μL template containing 5–250 ng DNA. PCR reaction was 95°C for 2 min, 35 cycles of 95°C for 30 s, 50°C for 30 s, and 72°C for 1 min and eventually 72°C for 5 min. For each replicate DNA sample, two separate PCR amplifications were conducted and afterwards united. Products were purified from agarose gels using Hi Yield® Gel/PCR DNA Fragments Extraction Kit (Süd-Laborbedarf GmbH, Gauting, Germany) and quantified with Quant-iT™ PicoGreen® dsDNA Assay Kit (Life Technologies / Thermo Fisher Scientific) in a Mithras LB 940 fluorimeter (Berthold Technologies, Bad Wildbad, Germany). Three replicates of a mock community consisting of 10 strains (Table S2) served as control for determining the sequencing error. From each bacterial sample and mock community 50 ng DNA was pooled. To reinstall an archaea-to-bacteria-ratio of about 1:100, archaeal samples were united and 20 ng DNA was added to the first mixture. The final mixture was sent to StarSEQ GmbH (Mainz, Germany) for 250-bp paired-end sequencing on an Illumina MiSeq instrument. Sequences have been deposited in the European Nucleotide Archive (EMBL-EBI; accession number PRJEB11366).

### Data processing

Environmental samples and the mock communities were processed separately for avoiding interference. In total 7,589,991 raw reads were obtained from the environmental samples (481,704 from the mock communities). Paired-end reads were merged with VSEARCH (version 1.9.5, github.com/torognes/vsearch) asking for a minimum 50 nt long overlap and a minimum 200 nt merged read length. This was achieved by 7,575,500 reads, i.e., 99.8% (mock: 480,061, 99.7%). Sequences with total expected errors E >1 were discarded with the fastq_filter command. Sequences shorter than 251 nt were discarded, and those longer were trimmed to 251 nt, followed by removing sequences with any ambiguous base or more than six nt long homopolymers using the screen.seqs command of mothur (version 1.31.2, Schloss et al., 2009). Thus, 5,668,815 (74.7%) good quality sequences (mock: 411,718, 85.5%) were retained. Using VSEARCH we removed singletons and chimeras which were identified by de novo chimera detection using the UCHIME algorithm (Edgar et al., 2011). Sequences were clustered into OTUs (operational taxonomic units) with USEARCH cluster_otus (version 8.1.1831, Edgar, 2010) at a threshold of 97% sequence identity and for each cluster a reference sequence was selected. Reference sequences were again checked for the presence of chimera against the reference database of the RDP trainset15_092015 (Cole et al., 2014). Ribosomal RNA sequences were extracted with Metaxa2 (Bengtsson-Palme et al., 2015). These curated sequences were taxonomically classified with mothur using the RDP reference database trainset14_032015 (Cole et al., 2014). Sequences not classified at the domain level and those classified as mitochondria or eukaryotes were removed from the dataset. These curated rRNA gene sequences were used as a reference database to map all good quality sequences including the previously removed singletons against it at a threshold of 97% identity with the usearch_global command of USEARCH. In total 4,720,785 (62.2%) good quality sequences were mapped to the seed of the environmental samples (mock: 405,481, 84.2%). Taxa classified as Cyanobacteria/Chloroplast were checked using Megablast search at the NCBI-website (Agarwala et al., 2016) and those not clearly identified as Cyanobacteria were removed from the data set. The sequencing error rate was estimated according to the MiSeq standard operating procedure (version from 18 April 2014 16:17, Kozich et al., 2013) using mothur and R (version 3.2.3, R Core Team, 2015). The mock communities served as proxy indicating the sequencing error rate was 0.06%. For the following analyses, bacterial and archaeal data were separated.

### Data analyses

Rarefaction curves were created using mothur and R. For calculating the abundance-based coverage estimator (ACE; Chao and Lee, 1992), the Shannon-Wiener Index (H′) expressed as N_1_ = e^H′^ (MacArthur, 1965), and H′-based evenness (J′), bacterial sequences were subsampled to the lowest number of reads (Gihring et al., 2012), i.e., 46,205 sequences per sample (the largest library was 169,863). The exponential form N_1_ was chosen, because it uses numbers of species as units and is therefore easier to interpret (Krebs, 1999). These indices and log-transformed qPCR data were analyzed using linear mixed effects models with the fraction set as fixed effect and the sample yielding the respective fractions defined as random effect, and Tukey's Honestly Significant Difference using R with the nlme package (version 3.1-131, Pinheiro et al., 2017) and the multcomp package (Hothorn et al., 2008), respectively. For calculating the *p*-value across all three treatments, “treatment” was included in the linear mixed effects models as random effect of highest rank.

Heatmaps with dendrograms based on Unweighted Pair Group Method with Arithmetic Mean were produced using the R-package gplots (version 2.17.0, Warnes et al., 2015). One-way Analysis of Similarity (ANOSIM) based on Bray-Curtis dissimilarity was performed with PAST (version 3.15, Hammer et al., 2001). With MEGA7 (version 7.0.18, Kumar et al., 2016) a Maximum Likelihood tree based on the Kimura 2-parameter model (Kimura, 1980) was constructed using 500-fold bootstrapping.

To identify preferences for PSFs, subpopulations derived from different fractions were compared using the R-package edgeR (version 3.12.0, Robinson et al., 2010). First, data were selected using a cut-off of 100 counts per million in at least three samples (Chen et al., 2015), followed by normalization based on the weighted trimmed mean of log expression ratios “TMM”-method (Robinson and Oshlack, 2010), analysis with the generalized linear model approach (McCarthy et al., 2012), and control of the false discovery rate using the algorithm by Benjamini and Hochberg (1995). In addition, the *p*-values were further adjusted using Bonferroni correction to account for multiple pair-wise comparisons. To consider meaningful rather than only statistical differences, the significant taxa were further selected. We proposed a meaningful difference when the higher sized subpopulation was four times bigger than the smaller one represented by >10 sequences, two times for >100, 1.5 times for >1000, and 1.25 times for >10,000 sequences. To determine the minimal meaningful difference (Diff_min_) between two subpopulations normalized to the library sizes' average a curve was fitted, which matched our proposed requirements with *R*^2^ = 0.9996 (Figure S1):

(1) $${\text{Diff}}_{\text{min}}=0.0104x^{4}-0.2199x^{3}+1.6701x^{2}-5.5083x+8.0079$$

with x being the decimal logarithm of the larger subpopulation's size. This equation is valid for a maximum subpopulation size of 60,635 sequences. For PSF preferences, only taxa significantly different in at least three pair-wise comparisons were considered, because this was the minimal number that allowed interpretation of these data. To reduce the number of taxa to be displayed in the preference-heatmap, only those taxa are shown which contributed with at least 0.1% to the community in at least one treatment or PSF. Taxa selected this way are displayed as ratios to the larger subpopulation's size by heatmap prepared with gplots. To provide an indicator for abundance a second heatmap based on the taxa's percentage was created and joined with the ratio-heatmap using CorelDraw X8 (version 18.0.0.448, Corel Corporation, Ottawa, Canada).

To evaluate the fractionation procedure the OTU-results were subjected to the following procedure: A virtual soil was constructed from the bacterial PSFs results for each replicate, i.e., UNF, NPK, and AM. After averaging the library sizes, the numbers of OTUs of the PSF-samples were multiplied with the bacterial 16S rRNA gene copy numbers in the respective proportions given by the particle size distribution, and summed up. The analysis was conducted on OTU-level as described above, except that all statistical differences were considered.

## Results

### Microbial abundances in unfractionated soil and particle size fractions

**Figure 1:**
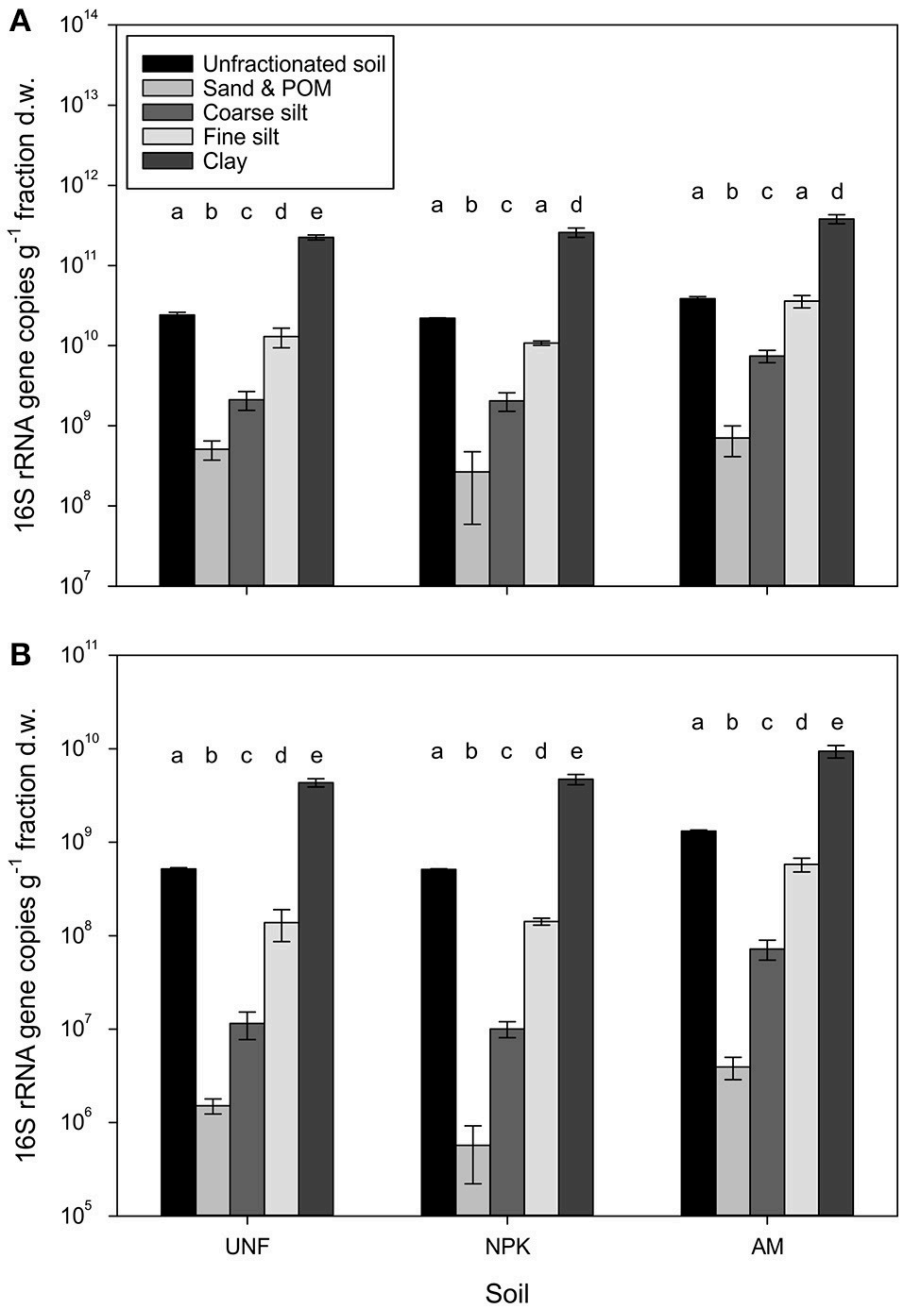
Bacterial **(A)** and archaeal **(B)** abundances represented by 16S rRNA gene copy numbers retrieved from three replicates, i.e., unfertilized soil (UNF), mineral fertilized soil (NPK), and animal manured soil (AM). Error bars represent standard deviations of technical triplicates. Different letters indicate significant differences between technical replicates between fractions within each fertilization treatment. The overall *p*-values between fractions across treatments for bacteria and archaea was <0.001 (*F* = 512.66) and <0.001 (*F* = 646.22), respectively.

The bacterial abundance in unfractionated soil ranged on average for 2.2 × 10^10^ to 3.9 × 10^10^ 16S rRNA gene copies per g dry soil (Figure 1A). Based on the dry weight of individual particle size fractions (PSFs), clay harbored 2.4–3.8 × 10^11^ gene copies. Gene numbers significantly decreased with increasing particle size by about one order of magnitude per size class (*p* < 0.001, Figure 1A, Table S3), reaching 4.9 × 10^8^ genes in the fraction containing sand and particulate organic matter (POM). In unfractionated soil the abundance of archaea was about two orders of magnitude lower than for bacteria ranging 0.5 × 10^9^ to 1.3 × 10^9^ genes (Figure 1B). Archaea also showed a significant decrease from clay (4.3–9.4 × 10^9^) to sand/POM (0.6–3.9 × 10^6^, *p* < 0.001, Figure 1B, Table S3). For both domains the pattern of decrease was the same for unfertilized soil (UNF), mineral fertilized soil (NPK), and soil receiving animal manure (AM).

### Efficacy of the soil fractionation procedure

**Figure 2:**
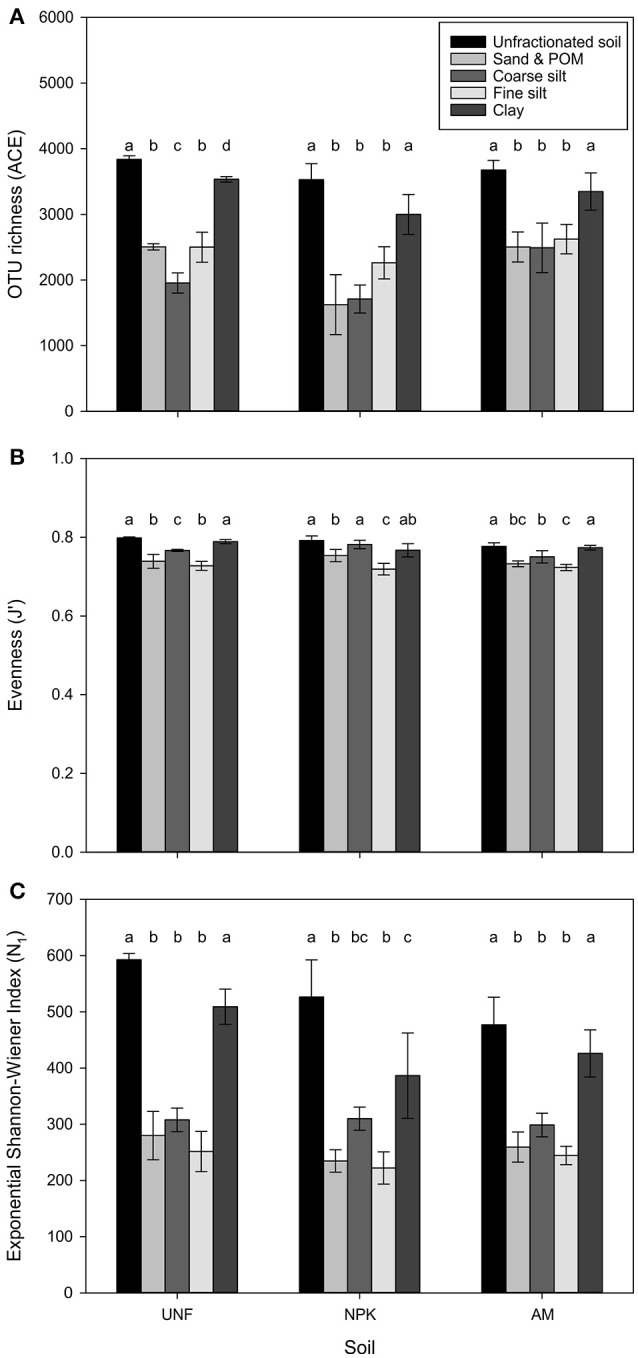
Estimates of bacterial OTU richness using the abundance-based coverage estimator (ACE) **(A)**, evenness (J′) based on the Shannon-Wiener Index (H′) **(B)**, and diversity H′ expressed in exponential form (e^H′^ = N_1_) **(C)** for three treatments, i.e., unfertilized soil (UNF), mineral fertilized soil (NPK), and animal manured soil (AM). Libraries were rarefied to 46,205 sequences. Error bars represent standard deviations of technical replicates and different letters significant differences between them for each fertilization treatment. The overall *p*-values between fractions across treatments was <0.001 each (F_ACE_ = 61.32, F_J′_ = 38.95, F_N1_ = 80.61).

Depending on soil nutrient treatment, 3,418 to 4,045 different bacterial OTUs were retrieved from unfractionated soils. The three coarsest fractions had comparably fewer OTUs, lower evenness, and smaller diversity (*p* < 0.001, Figures 2A–C, Table 1; Table S3). Compared with coarser fractions, clay clearly showed higher values for these indices. Rarefaction analysis indicated that most of the OTUs were detected with clay being similar to unfractionated soil and lower richness with the three coarsest fractions (Figure S2).

**Table 1:** Numbers of different bacterial OTUs (operational taxonomic units) from each treatment (UNF, unfertilized; NPK, mineral fertilized; AM, fertilized with animal manure) given as means and standard deviations from their three technical replicates.

	**UNF**	**NPK**	**AM**	**Mean**
Unfractionated soil	3, 784 ± 372	3, 418 ± 255	4, 045 ± 143	3, 749 ± 361
Sand with POM^a^	2, 374 ± 33	1, 670 ± 535	2, 594 ± 413	2, 213 ± 538
Coarse silt	1, 953 ± 146	1, 686 ± 178	2, 560 ± 423	2, 067 ± 456
Fine silt	2, 464 ± 186	2, 352 ± 180	2, 832 ± 148	2, 549 ± 264
Clay	3, 060 ± 12	3, 336 ± 59	3, 425 ± 220	3, 274 ± 200
Only found in unfractionated soil	227 ± 64	164 ± 32	167 ± 25	44 ± 10
Only found in size fractions	139 ± 28	149 ± 73	151 ± 41	46 ± 15

a POM, Particulate organic matter.

Between 164 and 227 bacterial OTUs present in unfractionated soil were not detected in any of the corresponding PSFs (Table 1), suggesting that they were lost in the soil fractionation process. However, these OTUs accounted for less than 0.4% of the sequences found in unfractionated soil (Table S4). Interestingly, PSFs also revealed OTUs not seen in the libraries of the unfractionated soil (Table 1), but these OTUs represented less than 0.9% of all sequences associated with the PSFs (Table S4).

The relative abundance of specific bacterial OTUs associated with PSFs was not always similar to that of the corresponding unfractionated soil as demonstrated by a comparison using virtually combined PSFs. Significant differences in the relative abundance were detected for 49–256 OTUs, depending on the replicate's origin (Table S5). Between 27 and 162 OTUs decreased significantly in relative abundance by 24–48%, and a total of 22 to 123 OTUs increased by 26–60%. The OTUs affected belonged mainly to *Actinobacteria, Alphaproteobacteria, Bacteroidetes*, and unclassified *Bacteria*. Considering all sequences, the decrease in OTUs following fractionation represented a loss of 0.1–2.5% of sequences isolated from unfractionated soils, while the increase accounted for 0.9–2.6% of PSF amplicon DNA sequences. Thus, the sums of DNA sequences obtained from PSFs agreed quantitatively with those detected in the unfractionated soil from which the PSFs were isolated.

### Bacterial diversity and preferences for soil particle size fractions

**Figure 3:**
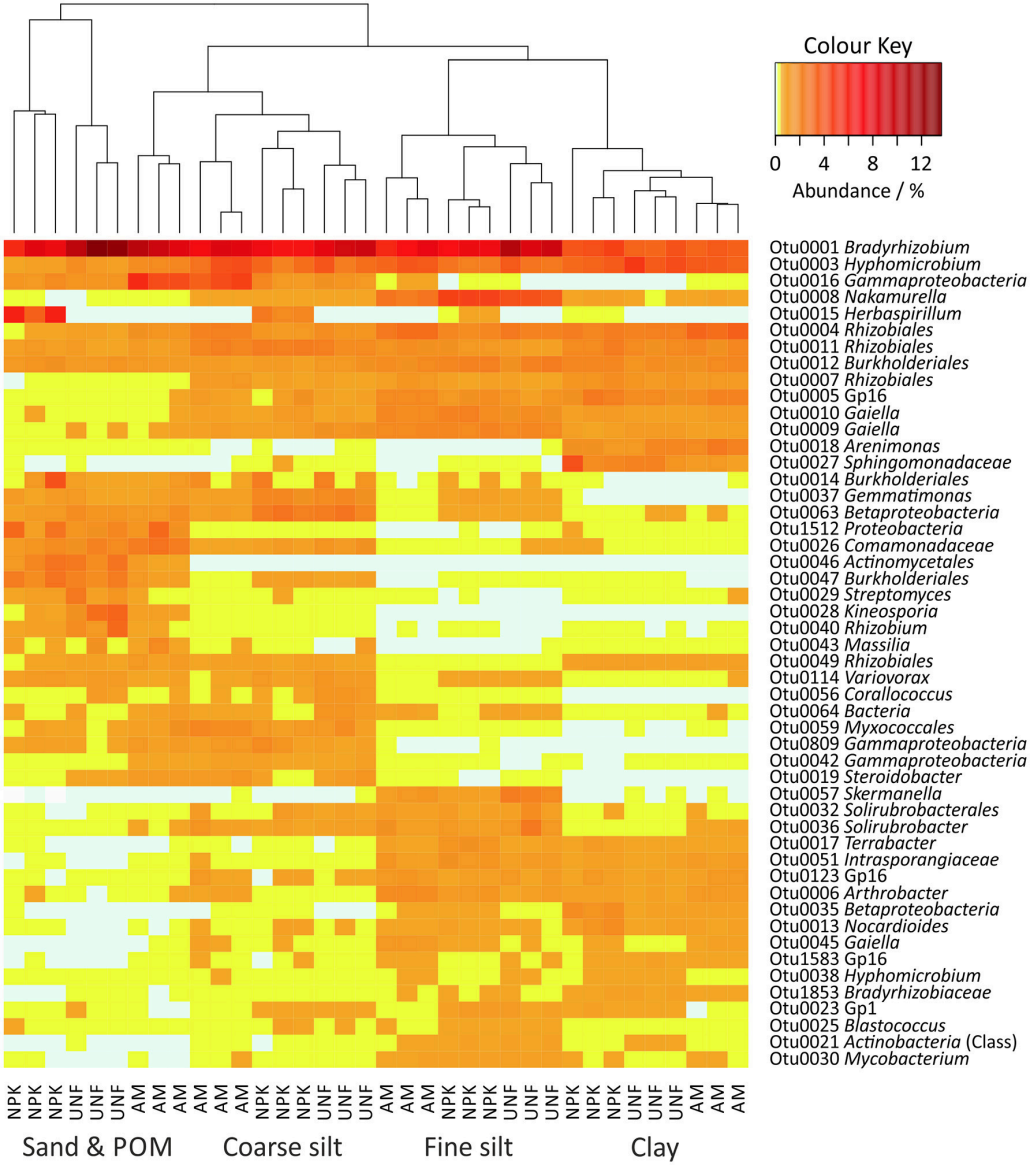
Heatmap of the 50 most abundant bacterial OTUs in soil particle size fractions (PSFs). The color key represents relative abundances as percentages.

A heatmap including the 50 most abundant bacterial OTUs retrieved from all PSFs (32–47% of all sequences) revealed that particle size was an important factor in structuring the bacterial community (Figure 3). Analysis of Similarity (ANOSIM) including all bacterial OTUs confirmed the uniqueness of the PSF-derived communities (*R* = 0.941, *p* < 0.001, Table S6).

To quantify the preference of specific bacterial taxa for a given PSF, the four differently sized PSFs were analyzed for each OTU and for higher taxonomical ranks. Among the 50 most abundant OTUs, the strongest preference was found for Otu0057 (*Skermanella*, Alphaproteobacteria, average abundance in preferred PSF 1.3 ± 0.6% of all sequences per sample, Supplementary Material S1, Figures S3, S4, Table S7). Depending on the replicate, this taxon was 35 to 364-fold more abundant in fine silt than in any other PSF. Similarly, the presence of Otu0028 (*Kineosporia*, Actinobacteria, 1.3 ± 1.0%) and Otu0046 (Actinomycetales, 1.6 ± 0.8%) were 23 to 175- and 21 to 75-fold, respectively, higher in sand/POM. Even stronger preferences were found for less abundant OTUs. For the 50 OTUs showing strongest differences, the differences between the smallest and largest PSF's subpopulation sizes ranged on average from 64–77- to 338-fold (only one replicate, Supplementary Material S1, Figures S3, S5, Table S7). The highest value was found for Otu6235 (*Comamonadaceae*, Betaproteobacteria, 0.1 ± 0.1%) and its preference in sand/POM.

**Figure 4:**
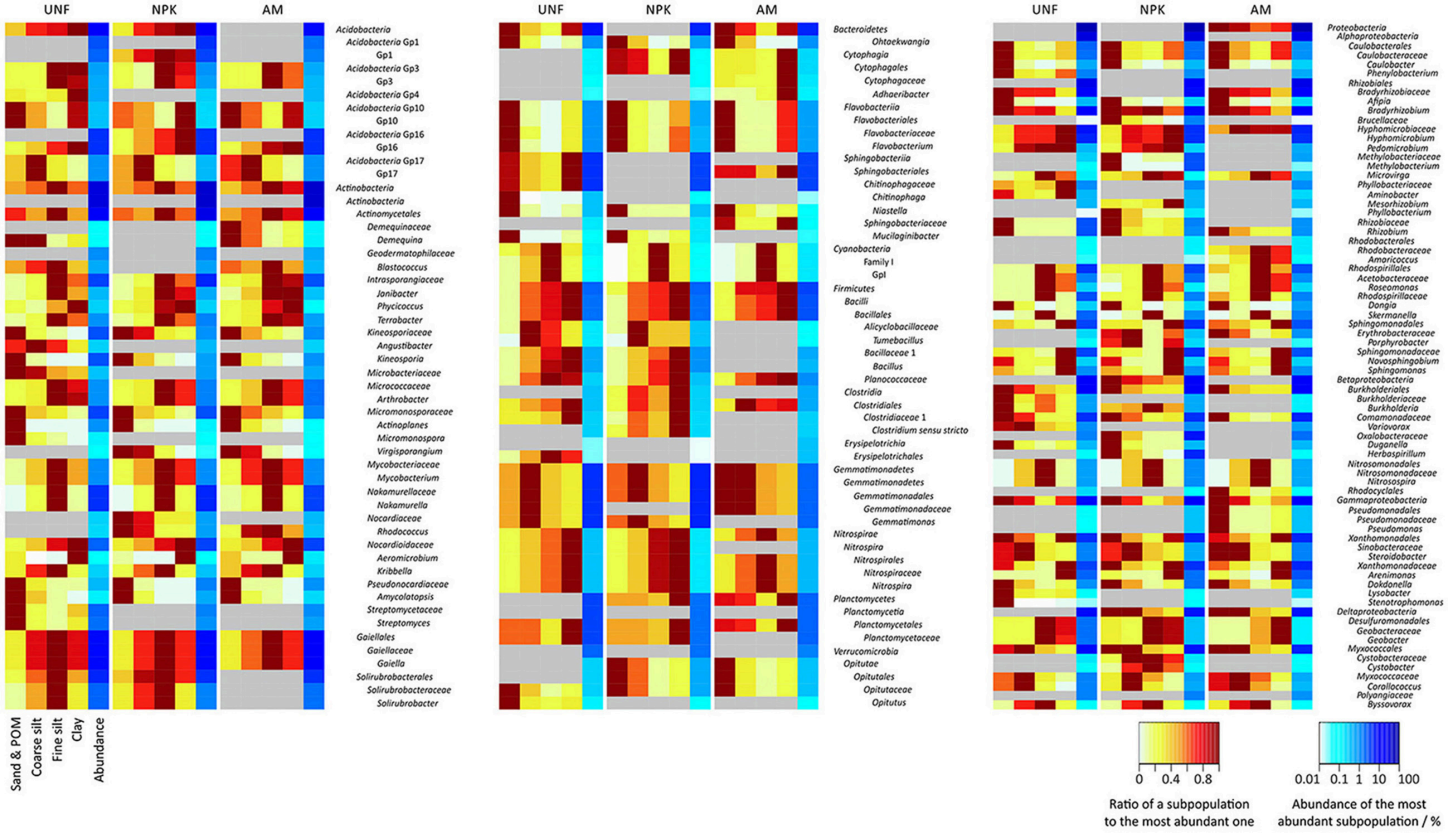
Heatmaps of bacterial taxa significantly differing between particle size fractions (PSFs) and contributing with at least 0.1% of total sequences in any PSF or soil variant. Differences between subpopulations are expressed as ratios to the most abundant one (red areas). Quantitative contributions to the communities are shown in blue on a logarithmic scale. Areas in gray indicate missing significance. For significance and variance see also in the Supplementary Material section S1 and Figures S3, S6–S8.

Depending on the fertilization treatment, the soils contained 321–472 OTUs with significant preferences for a specific PSF. These OTUs accounted for 67, 61, 64, and 52% of all sequences found in sand/POM, coarse silt, fine silt, and clay, respectively (Table 2). For some taxa, PSF preference was detected only in one fertilization treatment. However, 223 OTUs showed a preference in soil from all three nutrient treatments; these represented 34–56% of all sequences depending on the PSF (Table 2). Preference could differ between replicates originating from differently fertilized soils. For example, in replicates UNF and AM, the preference of *Mycobacterium* (Actinobacteria, 1.0 ± 0.1%) decreased in the order: fine silt > coarse silt = clay > sand/POM. The resolution was lower for NPK, where only a depletion in sand/POM was detected (Figure 4, Supplementary Material S1, Figures S3, S6, Table S12). Taxa showed no contradictory patterns of preference in the differently originating replicates (Figure 4, Figures S3–S8, also for OTUs not shown). Thus, the dominant bacterial taxa showed a distinct preference for a specific PSF regardless of the fertilization regime.

**Table 2:** Bacterial OTU numbers and the proportions exhibiting preferences for specific particle size fractions given as mean and standard deviation of corresponding technical replicates.

	**Number of OTUs**	**Contribution to total sequences/%**			
		**Sand with POM**	**Coarse silt**	**Fine silt**	**Clay**
UNF	406	70.4 ± 3.2	62.2 ± 0.9	65.5 ± 1.4	51.0 ± 1.2
NPK	472	71.3 ± 1.5	66.9 ± 0.5	71.4 ± 0.2	60.9 ± 0.8
AM	321	60.4 ± 1.5	53.6 ± 0.8	53.6 ± 0.6	44.4 ± 0.9
Preferences only found in UNF	79	4.9 ± 1.2	2.6 ± 0.3	3.2 ± 0.2	3.9 ± 0.1
Preferences only found in NPK	137	13.4 ± 4.5	7.4 ± 0.0	7.4 ± 0.2	9.1 ± 0.2
Preferences only found in AM	38	1.6 ± 0.4	1.7 ± 0.3	1.3 ± 0.0	1.2 ± 0.1
Preferences in all replicates					
UNF		54.0 ± 2.4	49.3 ± 1.0	50.8 ± 1.1	34.4 ± 1.0
NPK	223	48.8 ± 2.8	47.2 ± 1.2	50.5 ± 0.2	36.8 ± 1.5
AM		55.5 ± 1.6	47.3 ± 1.1	45.9 ± 0.9	36.8 ± 0.8

Taxa showing PSF-preferences in all three replicates are indicated in Figure 4 and Figures S6–S8 (see also Tables S8–S12). Sand/POM was the preferred site for *Kineosporia* and *Pseudonocardiaceae* (both Actinobacteria, average abundance in preferred PSF together 2.2 ± 1.0%), *Flavobacteriia* and other *Bacteroidetes* (together 1.6 ± 0.4%), *Caulobacteraceae, Rhizobium* and other *Alphaproteobacteria* (together 2.1 ± 1.9%). Only *Gemmatimonadales* (Gemmatimonadetes, 3.6 ± 1.3%) showed a preference for coarse silt. *Actinobacteria* (37.2 ± 3.0%, except the taxa mentioned above), GpI of the Cyanobacteria (0.1 ± 0.1%), *Acetobacteraceae* and *Skermanella* (both Alphaproteobacteria, together 2.6 ± 0.7%), and *Nitrosospira* (Betaproteobacteria, 0.7 ± 0.3%) were most abundant in fine silt. Highest relative abundance in clay was found for *Planctomycetales* (Planctomycetes, 2.2 ± 0.7%), *Sphingomonadaceae* (Alphaproteobacteria, 3.2 ± 1.1%), and *Arenimonas* (Gammaproteobacteria, 1.9 ± 0.4%). Several taxa showed a clear preference for more than one PSF, e.g. the two smallest fractions (fine silt and clay) with *Geobacter* (Deltaproteobacteria, 1.3 ± 0.9%).

The sand/POM fraction was significantly depleted in *Mycobacterium, Nakamurella*, and *Gaiella* (all Actinobacteria, average abundance in PSF with highest abundance together 13.7 ± 1.7%), most *Firmicutes* (2.2 ± 0.5%) and *Nitrosomonadaceae* (Betaproteobacteria, 0.7 ± 0.3%). Fine silt was low in *Acidobacteria* Gp10 (0.2 ± 0.0%), *Flavobacteriaceae* (Bacteroidetes, 0.6 ± 0.2%) and in *Xanthamonadaceae* (Gammaproteobacteria, 4.1 ± 0.8%). For clay, examples of depletion were *Acidobacteria* Gp17 (0.6 ± 0.3%) *Bradyrhizobium* (Alphaproteobacteria, 9.6 ± 2.5%) and *Corallococcus* (Deltaproteobacteria, 0.7 ± 0.4%). Interestingly, *Novosphingobium* (Alphaproteobacteria, 0.3 ± 0.0%) was depleted in both silt fractions.

### Archaeal diversity and response to particle size fractions

**Figure 5:**
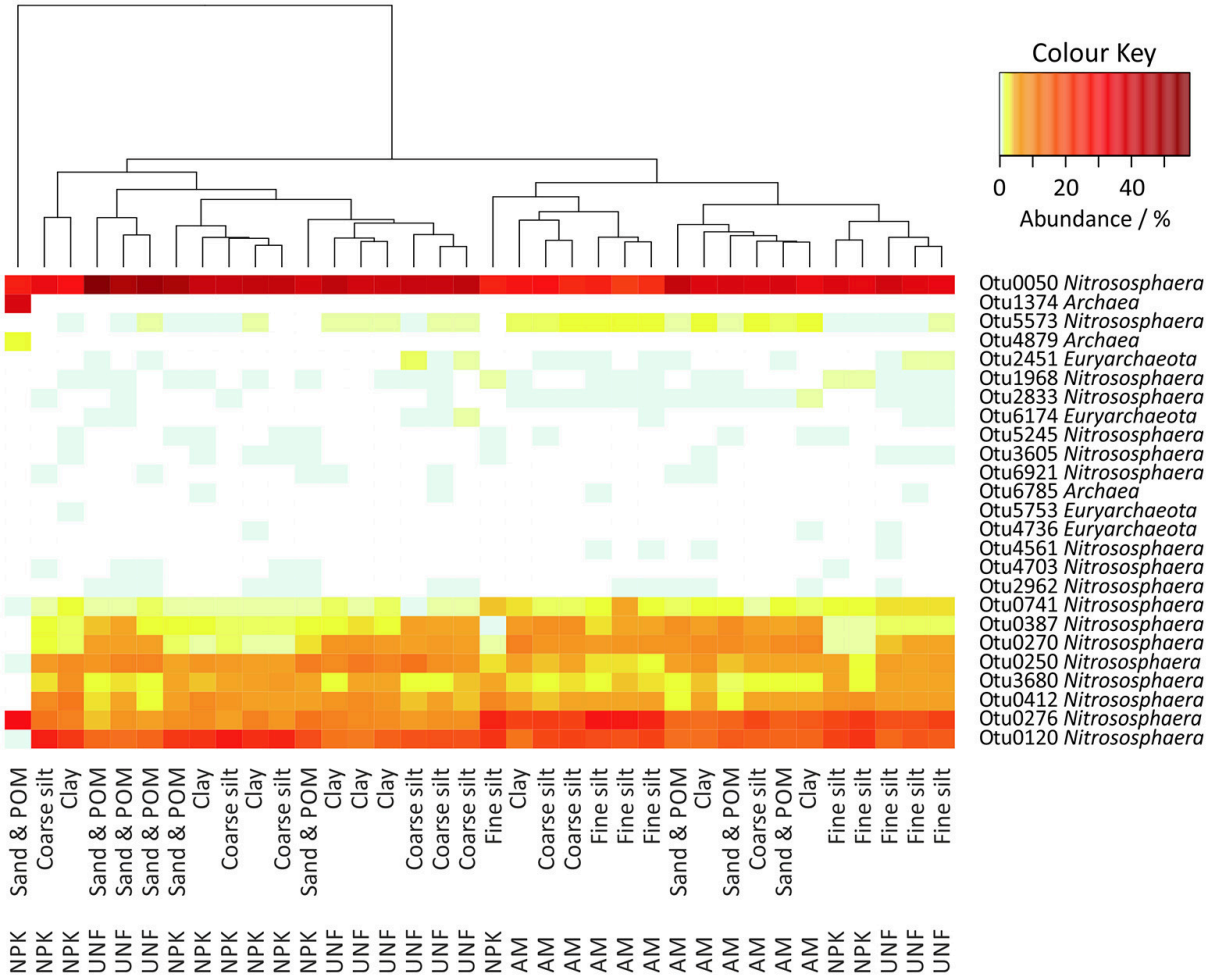
Heatmap of all archaeal OTUs in the soil particle size fractions (PSFs). The color key represents relative abundances.

A total of 37,887 archaeal sequences (0.8% of all prokaryotic sequences) were obtained with library sizes ranging from 319 to 1,432. Rarefaction curves indicated that the sampling effort captured most OTUs and thus was sufficient for comparisons (Figure S9). The sequences could be assigned to 25 OTUs. In each sample 98.3–100% of the sequences were classified as *Nitrososphaera*, except for one sand/POM sample from NPK that the authors considered an outlier (Figure 5). However, among these *Nitrososphaera*-assigned OTUs in a Maximum Likelihood tree, Otu0276 clustered together with *Candidatus* Nitrosocosmicus franklandus C13, and Otu4703 clustered together with two members of *Candidatus* Nitrosotalea (Figure S10). Megablast search at the NCBI-website (Agarwala et al., 2016) showed for the Euryarchaeotes (up to 1.7% of the sequences) equal or less than 82% 16S rRNA gene similarity to 16S rRNA genes of known methanogens.

A heatmap indicated stronger differences between the three different soil nutrient treatments than between different PSFs (Figure 5). However, fine silt isolated from UNF and NPK clustered together with PSFs from AM, while fine silt from AM clustered apart from UNF and NPK. ANOSIM confirmed the lack of clear differences between PSF-communities (*R* = 0.238, *p* < 0.001), although highlighting a subtle importance of fine silt (Table S6). Accordingly, a preference for fine silt was detected for Otu0276 (23.5 ± 3.9%) but only for soil from the UNF and NPK treatments and after excluding the outlier from the data set (Tables S13–S14).

## Discussion

Compared with conventional methods for soil particle size fractionation utilizing ultrasonic dispersion and centrifugation (Amelung et al., 1998), the present procedure applied a lower ultrasonic energy level to minimise detachment of cells from PSFs. This energy was sufficient to accomplish an adequate dispersion of this light textured sandy loam soil, as confirmed by soil texture data obtained with conventional methods (Hemkemeyer et al., 2015). Small losses of bacterial cells during fractionation were indicated by a comparison between 16S rRNA gene numbers derived from DNA in unfractionated soils and the sum of 16S rRNA gene numbers associated with individual PSFs. These losses were comparable to those Neumann et al. (2013) found by DNA content and qPCR analyses of the water after the fractionation of a loam. Certainly a transfer of detached bacterial cells between PSFs cannot be excluded. Suspended bacterial cells were probably collected together with the clay fraction given centrifugal force applied to sediment clay-sized particles (Peterson et al., 2012). However, the clay fraction represents the largest surface area (Neumann et al., 2013) and is associated with most of the soil microbial biomass (Jocteur Monrozier et al., 1991; Lensi et al., 1995; Stemmer et al., 1998). Any accumulation of detached cells in the clay-sized fraction is unlikely to have a strong effect on the composition of the bacterial taxa dominating with clay. In fact, the composition of communities derived from unfractionated soil and sums of those from corresponding PSFs was very similar.

Bacterial preferences for different PSFs support results from analyses of phospholipid-derived fatty acids in a clay loam paddy soil, where the finer fractions (<63 μm) were enriched in gram-positive bacteria (especially actinomycetes), while gram-negative bacteria were more abundant in coarser size fractions (Zhang et al., 2015). With the higher taxonomic resolution applied in the present study, we found that the sand/POM fraction from UNF and NPK soils was depleted in members of *Acidobacteria*, confirming a preference for smaller sized particles as previously suggested by sequencing of PSF-derived clone libraries from an arable clay loam (Sessitsch et al., 2001).

Up to 70% of the bacterial sequences represented OTUs with preference for a specific PSF. The preference of a high proportion of taxa for certain particle sizes can be explained by the different surface properties and microenvironments of PSFs. Soil particle surfaces provide specific conditions with respect to potential for cell attachment and colony formation, availability of nutrients, carbon, water and other essential growth factors. Most of these characteristics are affected by the mineralogical composition and modified by surface coatings of sesquioxides and organic matter (Guerin and Boyd, 1992; Rogers et al., 1998; Mauck and Roberts, 2007; Hemkemeyer et al., 2015). The mineral composition of the two coarser fractions (coarse silt and sand/POM) in the Askov soil is similar (dominated by quartz) and the major difference between the two PSFs relates to the presence of POM in the sand-sized fraction (Christensen, 1992, 2001). This may explain the preference of *Streptomycetaceae* in UNF, which are typically involved in the initial stages of decomposition (Chater et al., 2010). Particle-size preferences have also been shown for the polyaromatic hydrocarbon (PAH)-degrading *Mycobacterium* which was associated with the clay fraction known to accumulate most of the PAH in contaminated soils (Uyttebroek et al., 2006). Further, PSF-specific substrate preferences are indicated by studies of soil enzyme activities (Stemmer et al., 1998; Kandeler et al., 2000; Marx et al., 2005).

While most dominant bacterial taxa showed clear preferences for PSFs in the studied soil, the domain of archaea was less specific. Only for the single case of a *Candidatus* Nitrosocosmicus there was an increased relative abundance in fine silt. Interestingly, also the relative abundances of nitrifying bacteria, i.e., *Nitrosomonadales* and *Nitrospiraceae*, were highest in fine silt. Possibly surfaces of the silt particles represent hotspots for nitrification. Several studies have reported a correlation between N-mineralisation and smaller sized particles (Chichester, 1969, 1970; Cameron and Posner, 1979; Lowe and Hinds, 1983; Catroux and Schnitzer, 1987), but Nacro et al. (1996) suggested that most nitrifying organisms in a tropical soil reside in coarser fractions. Ammonification providing the substrate for nitrification was found to be located mainly in the finer fractions (Nacro et al., 1996; Bimüller et al., 2014). However, the majority of archaea in the Askov soil, which was dominated by *Nitrososphaera*, appeared to be independent of the nature of the PSFs. This is in contrast to findings from a study with an artificial soil where archaeal communities differed between fractions >20 and <20 μm (Hemkemeyer et al., 2014).

In structurally intact soil, most primary particles are incorporated into differently sized aggregates. While soil texture (the proportions of PSFs) is a relatively static property of a given soil, aggregates are structurally more dynamic and susceptible to mechanical soil disturbance. With regard to gas exchange, soil moisture, and substrate availability, soil aggregates represent a higher level of structural and functional complexity that ultimately shapes the microhabitats for bacteria and other members of the soil microbial community. Larger particles (coarse silt and sand) are less abundant in micro-aggregates (<250 μm; Kristiansen et al., 2006), and soil pores are smaller and gas exchange is restricted in micro-aggregates in periods when soils are wet. A large fraction of the coarse silt and sand-sized particles occur as individual particles or are incorporated into macro-aggregates (>250 μm). Microorganisms associated with macro-aggregates will be exposed to aerobic conditions for longer periods which could lead to a dominance of aerobic microorganisms (Sessitsch et al., 2001). Therefore, the preference of fermentative and nitrate respiring bacteria like *Opitutus* for the sand/POM fraction of UNF seems surprising (Chin et al., 2001). On the other hand, the outer and inner surface of a larger soil particle incorporated into macro-aggregates may be exposed to different microenvironments including redox conditions.

Interestingly, several taxa, e.g., Otu0098 (*Sphingomonas*, Alphaproteobacteria) and *Novosphingobium* were depleted in fine and coarse silt. Given the different mineralogical composition and the gradient in substrate quality from plant to microbial sources (Christensen, 1992; Ladd et al., 1996), a gradual change from coarser toward finer PSFs can be expected. Therefore, while depletion of certain bacterial components at the endpoints of this gradient can occur as a result of both abiotic and biotic restrictions, depletion in the medium-sized silt fractions may indicate competition or antagonism between microbial taxa.

The different fertilization regimes were chosen in this study as replicates to identify environmentally more robust preferences. Applied for more than 110 years, the fertilization treatments have induced differences in slowly changing soil properties such as soil organic matter, but the treatments also induce dynamic changes mediated by annual additions of plant nutrients and fresh substrates, i.e., crop residues and animal manure. Both categories of changes must have had an impact on the structure of the soil microbial community. Thus, PSF preferences found with all three replicates indicate environmental stability in the Askov soil, which was the case for up to 56% of the high quality bacterial 16S rRNA gene sequences. While this amplicon-based study demonstrates the appropriateness of the applied fractionation method to identify PSF preferences in the Askov loamy sand, more analyses including differently textured soils and different soil types as well as functional groups-based approaches are needed to enhance the insight into the importance of primary particle surface properties for supporting specific bacterial taxa, and more generally, spatial heterogeneity and bacterial diversity in soils.

## Author contributions

MH and CT designed the study and wrote the manuscript. MH collected all data and conducted the analysis. AD contributed to the analysis. BC sampled the soil, provided conceptual input and contributed finalizing the manuscript.

### Conflict of interest statement

The authors declare that the research was conducted in the absence of any commercial or financial relationships that could be construed as a potential conflict of interest.
