# Supplementary material for: Bacterial Preferences for Specific Soil Particle Size Fractions Revealed by Community Analyses
Source: Front Microbiol. 2018 Feb 23;9:149. doi: 10.3389/fmicb.2018.00149 (PMC5829042; doi:10.3389/fmicb.2018.00149)
Supplement: Supplementary file 1 [file Table1.DOCX]

Table S1 Sequences of proper primers and appendices

| **Forward illumina adapter** |  | **Forward index i5** |  | **Forward pad** |  | **Forward link** |  | **Forward primer: S-D-Arch-0519-a-S-19** |
| --- | --- | --- | --- | --- | --- | --- | --- | --- |
| AATGATACGGCGACCACCGAGATCTACAC |  | ACTATCTG |  | GATGGTAATC |  | AT |  | CAGCMGCCGCGGTAA |
| AATGATACGGCGACCACCGAGATCTACAC |  | TAGCGAGT |  | GATGGTAATC |  | AT |  | CAGCMGCCGCGGTAA |
| AATGATACGGCGACCACCGAGATCTACAC |  | CGATCTAC |  | GATGGTAATC |  | AT |  | CAGCMGCCGCGGTAA |
| AATGATACGGCGACCACCGAGATCTACAC |  | GTCGAGCA |  | GATGGTAATC |  | AT |  | CAGCMGCCGCGGTAA |
| **Reverse illumina adapter** |  | **Reverse index i7** |  | **Reverse pad** |  | **Reverse link** |  | **Reverse primer: S-D-Bact-00785-a-A-21** |
| CAAGCAGAAGACGGCATACGAGAT |  | AACTCTCG |  | AGTCAGTCAG |  | GA |  | GACTACHVGGGTATCTAATCC |
| CAAGCAGAAGACGGCATACGAGAT |  | CGTAGATC |  | AGTCAGTCAG |  | GA |  | GACTACHVGGGTATCTAATCC |
| CAAGCAGAAGACGGCATACGAGAT |  | GCGCACGT |  | AGTCAGTCAG |  | GA |  | GACTACHVGGGTATCTAATCC |
| CAAGCAGAAGACGGCATACGAGAT |  | TTAGTGAA |  | AGTCAGTCAG |  | GA |  | GACTACHVGGGTATCTAATCC |
| CAAGCAGAAGACGGCATACGAGAT |  | ACTATGTC |  | AGTCAGTCAG |  | GA |  | GACTACHVGGGTATCTAATCC |
| CAAGCAGAAGACGGCATACGAGAT |  | CAGTGAGT |  | AGTCAGTCAG |  | GA |  | GACTACHVGGGTATCTAATCC |
| CAAGCAGAAGACGGCATACGAGAT |  | GTCGCTCG |  | AGTCAGTCAG |  | GA |  | GACTACHVGGGTATCTAATCC |
| CAAGCAGAAGACGGCATACGAGAT |  | TGACACAA |  | AGTCAGTCAG |  | GA |  | GACTACHVGGGTATCTAATCC |
| CAAGCAGAAGACGGCATACGAGAT |  | CGTACTCA |  | AGTCAGTCAG |  | GA |  | GACTACHVGGGTATCTAATCC |
| CAAGCAGAAGACGGCATACGAGAT |  | ACATGCTG |  | AGTCAGTCAG |  | GA |  | GACTACHVGGGTATCTAATCC |
| CAAGCAGAAGACGGCATACGAGAT |  | GTCGTAGT |  | AGTCAGTCAG |  | GA |  | GACTACHVGGGTATCTAATCC |
| CAAGCAGAAGACGGCATACGAGAT |  | TAGCAGAC |  | AGTCAGTCAG |  | GA |  | GACTACHVGGGTATCTAATCC |
