# Supplementary material for: Bacterial Preferences for Specific Soil Particle Size Fractions Revealed by Community Analyses
Source: Front Microbiol. 2018 Feb 23;9:149. doi: 10.3389/fmicb.2018.00149 (PMC5829042; doi:10.3389/fmicb.2018.00149)
Supplement: Supplementary file 2 [file Table2.DOCX]

Table S2 Mock community composition combined with 5 ng DNA of each strain

| **Domain** | **Species** | **Strain** |
| --- | --- | --- |
| Bacteria | *Bacillus spec.* | ULT-145 |
| Bacteria | *Brachymonas denitrificans* | JCM9216 |
| Bacteria | *Comamonas nitrativorans* | DSM13191 |
| Bacteria | *Escherichia coli* | JM109 |
| Bacteria | *Paracoccus spec.* | I-Bh37-1 |
| Bacteria | *Pseudomonas stutzeri* | JM-300 |
| Bacteria | *Sinorhizobium meliloti* | Rm20115 |
| Archaea | Vector containing a 16S rRNA gene cloned from a soil sample | |
| Archaea | Vector containing a 16S rRNA gene cloned from a soil sample | |
| Archaea | Vector containing a 16S rRNA gene cloned from a soil sample | |
