# Supplementary material for: Bacterial Preferences for Specific Soil Particle Size Fractions Revealed by Community Analyses
Source: Front Microbiol. 2018 Feb 23;9:149. doi: 10.3389/fmicb.2018.00149 (PMC5829042; doi:10.3389/fmicb.2018.00149)
Supplement: Supplementary file 3 [file Table3.DOCX]

Table S3 Differences between unfractionated soil and each particle size fraction for the replicates unfertilised (UNF), mineral fertilised (NPK) animal manured soil (AM) given by F- and p-values derived from mixed effects models (*cf.* Figs. 1 and 2)

|  |  | **UNF** | |  | **NPK** | |  | **AM** | |
| --- | --- | --- | --- | --- | --- | --- | --- | --- | --- |
|  |  | **F** | **p** |  | **F** | **p** |  | **F** | **p** |
| Bacterial abundance | Intercept | 116,246.25 | < 0.001 |  | 43,222.79 | < 0.001 |  | 169,781.49 | < 0.001 |
|  | Fraction | 393.81 | < 0.001 |  | 248.62 | < 0.001 |  | 333.07 | < 0.001 |
| Archaeal abundance | Intercept | 57,329.62 | < 0.001 |  | 29,228.91 | < 0.001 |  | 154,296.20 | < 0.001 |
|  | Fraction | 663.84 | < 0.001 |  | 421.34 | < 0.001 |  | 722.51 | < 0.001 |
| Bacterial OTU richness | Intercept | 5,166.38 | < 0.001 |  | 946.86 | < 0.001 |  | 1,808.52 | < 0.001 |
|  | Fraction | 145.52 | < 0.001 |  | 21.96 | 0.002 |  | 12.97 | 0.008 |
| Bacterial evenness | Intercept | 90,850.58 | < 0.001 |  | 31,256.11 | < 0.001 |  | 88,590.67 | < 0.001 |
|  | Fraction | 29.26 | 0.001 |  | 13.76 | 0.007 |  | 17.93 | 0.004 |
| Bacterial diversity | Intercept | 2,341.02 | < 0.001 |  | 718.12 | < 0.001 |  | 833.50 | < 0.001 |
|  | Fraction | 74.94 | < 0.001 |  | 19.95 | 0.003 |  | 34.06 | 0.001 |
