# Supplementary material for: Bacterial Preferences for Specific Soil Particle Size Fractions Revealed by Community Analyses
Source: Front Microbiol. 2018 Feb 23;9:149. doi: 10.3389/fmicb.2018.00149 (PMC5829042; doi:10.3389/fmicb.2018.00149)
Supplement: Supplementary file 4 [file Table4.DOCX]

Table S4 Percentages of sequences found exclusively or not with unfractionated soil given as means of three technical replicates

|  | **Fraction** | **UNF** | **NPK** | **AM** |
| --- | --- | --- | --- | --- |
| Unfractionated soil only |  | 0.37 ± 0.03 | 0.28 ± 0.03 | 0.19 ± 0.01 |
| Absent from unfractionated soil | Sand & POM^a^ | 0.88 ± 0.05 | 0.83 ± 0.43 | 0.39 ± 0.04 |
|  | Coarse silt | 0.44 ± 0.04 | 0.40 ± 0.05 | 0.29 ± 0.01 |
|  | Fine silt | 0.30 ± 0.01 | 0.29 ± 0.01 | 0.20 ± 0.02 |
|  | Clay | 0.32 ± 0.00 | 0.33 ± 0.06 | 0.29 ± 0.02 |

^a^ POM = Particulate organic matter
