# Supplementary material for: Bacterial Preferences for Specific Soil Particle Size Fractions Revealed by Community Analyses
Source: Front Microbiol. 2018 Feb 23;9:149. doi: 10.3389/fmicb.2018.00149 (PMC5829042; doi:10.3389/fmicb.2018.00149)
Supplement: Supplementary file 5 [file Table5.DOCX]

Table S5 Comparison of unfractionated and virtually reconstructed soil

|  | **Taxa** | **UNF** | | | | |  | **NPK** | | | | |  | **AM** | | | | |
| --- | --- | --- | --- | --- | --- | --- | --- | --- | --- | --- | --- | --- | --- | --- | --- | --- | --- | --- |
|  |  | **Affected** | **Mean sum of sequences** | | | **Difference** |  | **Affected** | **Mean sum of sequences** | | | **Difference** |  | **Affected** | **Mean sum of sequences** | | | **Difference** |
|  |  | **OTUs** | **Unfraction-ated soil** |  | **Reconstruct-ed soil** | **/ %** |  | **OTUs** | **Unfraction-ated soil** |  | **Reconstruct-ed soil** | **/ %** |  | **OTUs** | **Unfraction-ated soil** |  | **Reconstruct-ed soil** | **/ %** |
| **Losses by fractionation** | *Acidobacteria* | 9 | 27.2 |  | 12.8 | -52.9 |  |  |  |  |  |  |  | 11 | 44.8 |  | 28.8 | -35.8 |
|  | *Actinobacteria* | 38 | 1,011.2 |  | 719.6 | -28.8 |  | 14 | 65.4 |  | 35.5 | -45.7 |  | 65 | 5,534.0 |  | 4,080.6 | -26.3 |
|  | *Bacteroidetes* | 9 | 74.9 |  | 40.2 | -46.3 |  | 2 | 1.4 |  | 0.8 | -40.5 |  | 15 | 202.0 |  | 153.5 | -24.0 |
|  | *Gemmatimonadetes* | 3 | 51.6 |  | 31.1 | -39.7 |  | 1 | 6.1 |  | 5.3 | -14.2 |  | 5 | 150.5 |  | 80.3 | -46.6 |
|  | *Latescibacteria* |  |  |  |  |  |  |  |  |  |  |  |  | 1 | 0.3 |  | 0.0 | -100.0 |
|  | *Planctomycetes* | 1 | 7.2 |  | 5.9 | -17.8 |  |  |  |  |  |  |  | 1 | 16.7 |  | 5.4 | -67.7 |
|  | *Proteobacteria* | 48 | 7,266.8 |  | 5,193.5 | -28.5 |  | 9 | 32.4 |  | 13.8 | -57.6 |  | 48 | 1,955.8 |  | 1,702.0 | -13.0 |
|  | *Alphaproteobacteria* | 28 | 5,540.1 |  | 3,946.3 | -28.8 |  | 5 | 24.1 |  | 10.8 | -55.2 |  | 22 | 428.1 |  | 362.7 | -15.3 |
|  | *Betaproteobacteria* | 5 | 69.8 |  | 36.5 | -47.7 |  |  |  |  |  |  |  | 7 | 86.0 |  | 49.0 | -43.0 |
|  | *Gammaproteobacteria* | 8 | 647.0 |  | 427.8 | -33.9 |  | 3 | 7.7 |  | 2.5 | -67.3 |  | 11 | 165.2 |  | 96.0 | -41.8 |
|  | *Deltaproteobacteria* | 6 | 955.1 |  | 745.0 | -22.0 |  | 1 | 0.7 |  | 0.5 | -31.6 |  | 7 | 1,275.3 |  | 1,194.2 | -6.4 |
|  | Unclassified  *Proteobacteria* | 1 | 54.8 |  | 37.9 | -30.9 |  |  |  |  |  |  |  | 1 | 1.3 |  | 0.1 | -95.8 |
|  | *Spirochaetes* | 1 | 1.2 |  | 0.5 | -53.6 |  |  |  |  |  |  |  |  |  |  |  |  |
|  | *Verrucomicrobia* | 14 | 557.5 |  | 408.5 | -26.7 |  |  |  |  |  |  |  | 2 | 17.7 |  | 10.5 | -40.5 |
|  | Unclassified *Bacteria* | 10 | 306.9 |  | 259.4 | -15.5 |  | 1 | 1.3 |  | 0.5 | -64.8 |  | 14 | 63.0 |  | 30.6 | -51.5 |
|  | **Total** | **133** | **9,304.4** |  | **6,671.6** | **-28.3** |  | **27** | **106.7** |  | **55.9** | **-47.7** |  | **162** | **7,984.7** |  | **6,091.5** | **-23.7** |
| **Gains by fractionation** | *Acidobacteria* | 5 | 29.7 |  | 34.9 | +17.3 |  |  |  |  |  |  |  | 5 | 29.5 |  | 41.8 | +41.5 |
|  | *Actinobacteria* | 38 | 5,712.5 |  | 6,778.6 | +18.7 |  | 14 | 1,140.6 |  | 2,002.3 | +75.5 |  | 29 | 2,241.3 |  | 3,966.7 | +77.0 |
|  | *Bacteroidetes* | 17 | 531.6 |  | 660.9 | +24.3 |  | 1 | 15.2 |  | 19.7 | +29.6 |  | 10 | 24.9 |  | 48.2 | +93.7 |
|  | *Firmicutes (Bacilli)* |  |  |  |  |  |  |  |  |  |  |  |  | 1 | 0.8 |  | 1.3 | +61.6 |
|  | *Gemmatimonadetes* | 2 | 37.7 |  | 40.2 | +6.7 |  |  |  |  |  |  |  | 3 | 26.1 |  | 29.3 | +12.2 |
|  | *Nitrospirae* | 1 | 351.1 |  | 645.9 | +84.0 |  |  |  |  |  |  |  |  |  |  |  |  |
|  | *Planctomycetes* | 2 | 16.2 |  | 17.3 | +7.1 |  |  |  |  |  |  |  |  |  |  |  |  |
|  | *Proteobacteria* | 28 | 1,220.8 |  | 1,573.8 | +28.9 |  | 5 | 392.4 |  | 450.5 | +14.8 |  | 27 | 795.3 |  | 1,032.9 | +29.9 |
|  | *Alphaproteobacteria* | 12 | 662.7 |  | 915.1 | +38.1 |  | 4 | 389.2 |  | 446.6 | +14.8 |  | 11 | 401.5 |  | 594.0 | +47.9 |
|  | *Betaproteobacteria* | 6 | 298.1 |  | 345.0 | +15.7 |  | 1 | 3.1 |  | 3.9 | +22.4 |  | 7 | 386.8 |  | 423.2 | +9.4 |
|  | *Gammaproteobacteria* | 8 | 243.8 |  | 290.9 | +19.4 |  |  |  |  |  |  |  | 4 | 4.0 |  | 9.7 | +141.3 |
|  | *Deltaproteobacteria* | 2 | 16.2 |  | 22.8 | +41.5 |  |  |  |  |  |  |  | 5 | 2.9 |  | 5.9 | +103.1 |
|  | *Verrucomicrobia* | 13 | 467.0 |  | 727.8 | +55.8 |  |  |  |  |  |  |  | 3 | 36.3 |  | 40.2 | +10.7 |
|  | Unclassified *Bacteria* | 17 | 878.2 |  | 1,131.3 | +28.8 |  | 2 | 1.6 |  | 3.2 | +96.4 |  | 15 | 325.4 |  | 373.2 | +14.7 |
|  | **Total** | **123** | **9,244.8** |  | **11,610.7** | **+25.6** |  | **22** | **1,549.8** |  | **2,475.7** | **+59.7** |  | **93** | **3,479.6** |  | **5,533.4** | **+59.0** |
