# Supplementary material for: Bacterial Preferences for Specific Soil Particle Size Fractions Revealed by Community Analyses
Source: Front Microbiol. 2018 Feb 23;9:149. doi: 10.3389/fmicb.2018.00149 (PMC5829042; doi:10.3389/fmicb.2018.00149)
Supplement: Supplementary file 6 [file Table6.DOCX]

Table S6 Pair-wise one-way Analysis of Similarity (ANOSIM) of operational taxonomic units (OTUs)

|  |  | **Coarse silt** | |  | **Fine silt** | |  | **Clay** | |
| --- | --- | --- | --- | --- | --- | --- | --- | --- | --- |
|  |  | **R** | **p** |  | **R** | **p** |  | **R** | **p** |
| Bacteria | Sand with POM^a^ | **0.826** | **0.001** |  | **1.000** | **< 0.001** |  | **1.000** | **< 0.001** |
|  | Coarse silt |  |  |  | **0.981** | **< 0.001** |  | **1.000** | **< 0.001** |
|  | Fine silt |  |  |  |  |  |  | **0.987** | **0.001** |
| Archaea | Sand with POM^a^ | 0.055 | 0.188 |  | 0.481 | **< 0.001** |  | 0.057 | 0.173 |
|  | Coarse silt |  |  |  | 0.349 | **0.006** |  | 0.005 | 0.358 |
|  | Fine silt |  |  |  |  |  |  | 0.430 | **< 0.001** |

^a^ POM = Particulate organic matter
In the case of significance at 5% level (bold p-values), well separated communities (R > 0.75) are indicated in bold. An R > 0.5 indicates separated, but overlapping communities, while R < 0.25 is considered to express barely separated communities.
