# Supplementary material for: Bacterial Preferences for Specific Soil Particle Size Fractions Revealed by Community Analyses
Source: Front Microbiol. 2018 Feb 23;9:149. doi: 10.3389/fmicb.2018.00149 (PMC5829042; doi:10.3389/fmicb.2018.00149)
Supplement: Supplementary file 7 [file Table7.DOCX]

Table S7 Significance values for bacterial operational taxonomic units (OUTs) after Bonferroni-correction to account for multiple pair-wise comparisons between sand & particulate organic matter (POM), coarse silt, fine silt, and clay for all three replicates, i.e. unfertilised soil (UNF), mineral fertilised soil (NPK), and soil receiving animal manure (AM)

| **OTU** | **UNF** | | | | | |  | **NPK** | | | | | |  | **AM** | | | | | |
| --- | --- | --- | --- | --- | --- | --- | --- | --- | --- | --- | --- | --- | --- | --- | --- | --- | --- | --- | --- | --- |
|  | **Sand/POM - Coarse silt** | **Sand/POM - Fine silt** | **Sand/POM - Clay** | **Coarse silt - Fine silt** | **Coarse silt - Clay** | **Fine silt - Clay** |  | **Sand/POM - Coarse silt** | **Sand/POM - Fine silt** | **Sand/POM - Clay** | **Coarse silt - Fine silt** | **Coarse silt - Clay** | **Fine silt - Clay** |  | **Sand/POM - Coarse silt** | **Sand/POM - Fine silt** | **Sand/POM - Clay** | **Coarse silt - Fine silt** | **Coarse silt - Clay** | **Fine silt - Clay** |
| OTU_1 | **0.001** | 0.777 | **< 0.001** | 1.000 | **< 0.001** | **< 0.001** |  | **0.001** | 1.000 | **< 0.001** | 0.612 | **0.029** | **< 0.001** |  | 1.000 | 1.000 | **< 0.001** | 1.000 | **< 0.001** | **< 0.001** |
| OTU_10 | **0.007** | **< 0.001** | **0.003** | **0.001** | 1.000 | **0.001** |  | **0.007** | **< 0.001** | **< 0.001** | **< 0.001** | 1.000 | **0.003** |  | 1.000 | **< 0.001** | 0.063 | **< 0.001** | 1.000 | **0.014** |
| OTU_100 | **0.002** | **< 0.001** | **< 0.001** | 1.000 | 0.127 | 1.000 |  | **0.002** | **< 0.001** | **< 0.001** | 0.539 | **0.035** | 1.000 |  | **0.004** | **< 0.001** | **< 0.001** | 1.000 | 0.124 | 0.390 |
| OTU_1000 | 1.000 | 1.000 | 1.000 | 1.000 | 1.000 | 1.000 |  | 1.000 | 1.000 | 1.000 | 1.000 | 1.000 | 1.000 |  | 1.000 | 0.894 | 1.000 | 0.197 | 0.637 | 1.000 |
| OTU_1001 | 1.000 | 1.000 | **0.004** | 1.000 | **0.006** | **< 0.001** |  | 1.000 | 1.000 | 0.180 | 1.000 | **0.035** | **0.025** |  | 1.000 | 1.000 | 0.666 | 1.000 | 1.000 | **0.028** |
| OTU_1002 | 1.000 | 1.000 | 1.000 | 0.532 | 0.122 | 1.000 |  | 1.000 | 1.000 | 1.000 | 1.000 | 1.000 | 1.000 |  | 1.000 | 1.000 | 1.000 | 1.000 | 0.292 | 1.000 |
| OTU_1004 | 1.000 | 1.000 | 1.000 | 1.000 | 1.000 | 1.000 |  | 1.000 | 0.073 | 0.217 | **0.007** | **0.018** | 1.000 |  | 1.000 | 0.116 | 1.000 | 1.000 | 1.000 | 1.000 |
| OTU_1005 | 1.000 | 1.000 | 1.000 | 1.000 | 1.000 | 1.000 |  | 1.000 | 1.000 | 1.000 | 1.000 | 1.000 | 1.000 |  | 1.000 | 1.000 | 1.000 | 1.000 | 0.354 | 1.000 |
| OTU_1006 | 1.000 | 1.000 | **0.001** | 1.000 | **0.024** | 0.077 |  | 1.000 | 1.000 | **0.037** | 0.086 | **< 0.001** | 0.933 |  | 1.000 | 1.000 | 0.621 | 1.000 | 1.000 | 1.000 |
| OTU_1007 | 1.000 | 1.000 | 1.000 | 1.000 | 1.000 | 1.000 |  | 1.000 | 1.000 | 1.000 | 0.087 | **0.029** | 1.000 |  | 1.000 | 1.000 | 1.000 | 1.000 | 1.000 | 1.000 |
| OTU_1009 | 0.792 | 0.590 | 1.000 | 1.000 | 0.773 | 1.000 |  | 0.792 | 1.000 | 0.077 | 1.000 | **0.003** | **0.006** |  | 1.000 | 0.132 | 1.000 | 1.000 | 0.103 | **0.008** |
| OTU_101 | 1.000 | 1.000 | **0.003** | 1.000 | **0.002** | **0.026** |  | 1.000 | 1.000 | 0.483 | 0.698 | **0.006** | 1.000 |  | 1.000 | 1.000 | 1.000 | 1.000 | **0.036** | 1.000 |
| OTU_1010 | 1.000 | 1.000 | 1.000 | 1.000 | 1.000 | 1.000 |  | 1.000 | 0.451 | 0.349 | 1.000 | 1.000 | 1.000 |  | 1.000 | 1.000 | 1.000 | 1.000 | 1.000 | 1.000 |
| OTU_1011 | 0.743 | 1.000 | 1.000 | 1.000 | 1.000 | 1.000 |  | 0.743 | 1.000 | 1.000 | 1.000 | 1.000 | 1.000 |  | 1.000 | 0.475 | 1.000 | 1.000 | 1.000 | 1.000 |
| OTU_1012 | **0.012** | 0.161 | 1.000 | 1.000 | **0.008** | 0.343 |  | **0.012** | 0.301 | 1.000 | 1.000 | **0.048** | 0.567 |  | 1.000 | 0.124 | 1.000 | 1.000 | 1.000 | 0.118 |
| OTU_1013 | 1.000 | **< 0.001** | 0.154 | 0.067 | 1.000 | 1.000 |  | 1.000 | 1.000 | 1.000 | 1.000 | 1.000 | 1.000 |  | 1.000 | **0.002** | 1.000 | **0.021** | 1.000 | 1.000 |
| OTU_1014 | 1.000 | 1.000 | 1.000 | 1.000 | 1.000 | 1.000 |  | 1.000 | 1.000 | 0.350 | 1.000 | 1.000 | 1.000 |  | 1.000 | 1.000 | 1.000 | 1.000 | 1.000 | 1.000 |
| OTU_1016 | 1.000 | **0.009** | 1.000 | **0.003** | 0.940 | 1.000 |  | 1.000 | **< 0.001** | **0.009** | **< 0.001** | **0.007** | 1.000 |  | 1.000 | **< 0.001** | 0.358 | **< 0.001** | 0.246 | 1.000 |
| OTU_1017 | **0.002** | **0.006** | **< 0.001** | 1.000 | 1.000 | 1.000 |  | **0.002** | 1.000 | 1.000 | 1.000 | 1.000 | 1.000 |  | 1.000 | 0.618 | 0.436 | 1.000 | 1.000 | 1.000 |
| OTU_1018 | 1.000 | 1.000 | 1.000 | 1.000 | 1.000 | 1.000 |  | 1.000 | 0.123 | **0.011** | 1.000 | 1.000 | 1.000 |  | 1.000 | 1.000 | 1.000 | 1.000 | 1.000 | 1.000 |
| OTU_1019 | 1.000 | 0.094 | 0.892 | 1.000 | 1.000 | 1.000 |  | 1.000 | **0.045** | **0.001** | 1.000 | 0.218 | 1.000 |  | 1.000 | **0.002** | 0.791 | 1.000 | 1.000 | 1.000 |
| OTU_102 | 0.208 | **< 0.001** | 0.060 | **< 0.001** | 1.000 | **< 0.001** |  | 0.208 | **< 0.001** | **< 0.001** | **< 0.001** | 1.000 | **< 0.001** |  | **0.020** | **< 0.001** | **< 0.001** | **< 0.001** | 1.000 | **< 0.001** |
| OTU_1020 | 1.000 | 1.000 | 1.000 | 1.000 | 1.000 | 1.000 |  | 1.000 | 1.000 | 1.000 | 1.000 | 0.724 | 1.000 |  | 0.259 | 0.774 | 1.000 | 1.000 | 1.000 | 1.000 |
| OTU_1021 | 1.000 | 0.338 | 1.000 | 1.000 | 1.000 | 1.000 |  | 1.000 | 0.910 | 0.290 | 1.000 | 1.000 | 1.000 |  | 1.000 | 1.000 | 1.000 | 1.000 | 1.000 | 1.000 |
| OTU_1022 | 1.000 | 1.000 | 0.120 | 1.000 | **0.006** | 0.851 |  | 1.000 | 1.000 | 1.000 | 1.000 | 1.000 | 1.000 |  | 1.000 | 1.000 | 1.000 | 1.000 | 1.000 | 1.000 |
| OTU_1023 | 1.000 | 0.133 | 1.000 | **< 0.001** | 0.100 | 1.000 |  | 1.000 | 0.110 | **0.042** | 0.161 | 0.051 | 1.000 |  | 1.000 | **< 0.001** | **< 0.001** | **0.011** | 0.147 | 1.000 |
| OTU_1024 | 0.830 | **< 0.001** | **0.032** | 0.127 | 1.000 | 1.000 |  | 0.830 | **< 0.001** | **< 0.001** | **< 0.001** | 0.394 | **0.047** |  | 1.000 | **< 0.001** | **0.002** | **0.005** | 1.000 | 1.000 |
| OTU_1025 | 1.000 | **0.029** | 1.000 | 1.000 | 1.000 | 0.694 |  | 1.000 | **0.004** | 1.000 | 1.000 | 1.000 | 0.116 |  | 0.426 | **0.006** | 1.000 | 1.000 | 1.000 | 0.483 |
| OTU_1026 | 1.000 | 1.000 | 0.064 | 0.916 | **0.035** | 1.000 |  | 1.000 | 0.291 | 1.000 | 1.000 | 1.000 | 1.000 |  | 1.000 | 1.000 | 0.060 | 1.000 | 0.176 | 1.000 |
| OTU_1027 | 0.217 | 1.000 | 0.651 | 1.000 | 1.000 | 1.000 |  | 0.217 | 1.000 | 0.346 | 1.000 | 1.000 | 1.000 |  | 1.000 | 1.000 | 1.000 | 1.000 | 1.000 | 1.000 |
| OTU_1028 | 1.000 | 1.000 | 1.000 | 1.000 | 1.000 | 1.000 |  | 1.000 | 1.000 | 1.000 | 1.000 | 1.000 | 1.000 |  | 1.000 | 1.000 | 0.207 | 1.000 | 0.242 | 1.000 |
| OTU_1029 | 1.000 | 1.000 | 0.761 | 1.000 | 1.000 | 1.000 |  | 1.000 | 1.000 | 1.000 | 1.000 | 1.000 | 1.000 |  | 0.916 | 0.515 | 1.000 | 1.000 | 0.491 | 0.869 |
| OTU_103 | 1.000 | 0.819 | 0.171 | 1.000 | 1.000 | 1.000 |  | 1.000 | **< 0.001** | **< 0.001** | 1.000 | 0.167 | 1.000 |  | 0.311 | 0.116 | 0.053 | 1.000 | 1.000 | 1.000 |
| OTU_1030 | 0.112 | 1.000 | 1.000 | 1.000 | 0.243 | 1.000 |  | 0.112 | 0.710 | 1.000 | 1.000 | 1.000 | 0.649 |  | 1.000 | 1.000 | 1.000 | 1.000 | 1.000 | 1.000 |
| OTU_1031 | 0.959 | 1.000 | 0.614 | 1.000 | 1.000 | 1.000 |  | 0.959 | 1.000 | 0.063 | 1.000 | 1.000 | 0.382 |  | 1.000 | 1.000 | 0.707 | 1.000 | 1.000 | 0.251 |
| OTU_1032 | 1.000 | 1.000 | 1.000 | 0.994 | 1.000 | 1.000 |  | 1.000 | **0.031** | 1.000 | 1.000 | 1.000 | 1.000 |  | 1.000 | 0.490 | 1.000 | **0.036** | 1.000 | 1.000 |
| OTU_1033 | 1.000 | 1.000 | 1.000 | 1.000 | 1.000 | 1.000 |  | 1.000 | 0.154 | 1.000 | 1.000 | 1.000 | 1.000 |  | 1.000 | 1.000 | 1.000 | 1.000 | 0.178 | 1.000 |
| OTU_1034 | 1.000 | 0.205 | **0.031** | 1.000 | 0.648 | 1.000 |  | 1.000 | 0.611 | 0.065 | 1.000 | 0.395 | 1.000 |  | 1.000 | 0.141 | **0.028** | 1.000 | 0.283 | 1.000 |
| OTU_1036 | 1.000 | 0.080 | **0.023** | 1.000 | 0.591 | 1.000 |  | 1.000 | 1.000 | 0.109 | 0.714 | 1.000 | 1.000 |  | 1.000 | 1.000 | 1.000 | 1.000 | 1.000 | 1.000 |
| OTU_1037 | 1.000 | 1.000 | 1.000 | 1.000 | 1.000 | 1.000 |  | 1.000 | 1.000 | 1.000 | 0.598 | 1.000 | 1.000 |  | 1.000 | 1.000 | 1.000 | 1.000 | 1.000 | 1.000 |
| OTU_1038 | 1.000 | 0.091 | **0.036** | **0.041** | **0.010** | 1.000 |  | 1.000 | **0.012** | 0.136 | 0.323 | 1.000 | 1.000 |  | 1.000 | 0.319 | 1.000 | 0.341 | 0.855 | 1.000 |
| OTU_1039 | 1.000 | 1.000 | 1.000 | 1.000 | 1.000 | 1.000 |  | 1.000 | **0.049** | 0.312 | 0.941 | 1.000 | 1.000 |  | 1.000 | 1.000 | 1.000 | 1.000 | 1.000 | 1.000 |
| OTU_104 | 1.000 | **< 0.001** | 1.000 | **< 0.001** | 1.000 | **< 0.001** |  | 1.000 | **< 0.001** | 1.000 | **< 0.001** | 1.000 | **< 0.001** |  | 1.000 | **< 0.001** | 0.164 | **< 0.001** | 0.512 | 0.205 |
| OTU_1040 | 1.000 | 1.000 | 0.422 | 0.830 | **0.012** | 1.000 |  | 1.000 | 1.000 | 0.852 | 1.000 | **0.026** | 1.000 |  | 1.000 | 1.000 | 0.571 | 1.000 | **0.018** | 1.000 |
| OTU_1042 | **0.025** | 1.000 | 1.000 | 0.351 | 0.061 | 1.000 |  | **0.025** | 1.000 | 1.000 | 1.000 | 1.000 | 1.000 |  | 1.000 | 1.000 | 1.000 | 1.000 | 1.000 | 1.000 |
| OTU_1044 | 0.952 | 0.094 | 1.000 | 1.000 | 0.828 | 0.224 |  | 0.952 | 1.000 | 1.000 | 1.000 | 1.000 | 1.000 |  | 1.000 | 1.000 | 1.000 | 1.000 | 1.000 | 1.000 |
| OTU_1045 | 1.000 | 1.000 | 1.000 | 1.000 | 1.000 | 1.000 |  | 1.000 | 1.000 | 1.000 | 1.000 | 1.000 | 1.000 |  | 1.000 | 1.000 | 1.000 | 1.000 | 1.000 | 1.000 |
| OTU_1046 | 1.000 | 1.000 | 1.000 | 1.000 | 1.000 | 1.000 |  | 1.000 | 1.000 | 1.000 | **0.022** | 1.000 | 0.620 |  | 1.000 | 1.000 | 1.000 | 1.000 | 1.000 | 1.000 |
| OTU_1047 | 1.000 | 0.224 | 1.000 | 0.187 | 1.000 | 1.000 |  | 1.000 | **< 0.001** | **< 0.001** | 1.000 | 1.000 | 1.000 |  | 1.000 | 1.000 | 1.000 | 1.000 | 1.000 | 1.000 |
| OTU_1049 | 1.000 | 1.000 | 1.000 | 1.000 | 1.000 | 1.000 |  | 1.000 | 0.121 | 0.205 | 1.000 | 1.000 | 1.000 |  | 1.000 | 1.000 | 1.000 | 1.000 | 1.000 | 1.000 |
| OTU_105 | **< 0.001** | **< 0.001** | **< 0.001** | **< 0.001** | **0.006** | 0.694 |  | **< 0.001** | **< 0.001** | **< 0.001** | **0.003** | 0.523 | **< 0.001** |  | **0.005** | **< 0.001** | **0.032** | **< 0.001** | 1.000 | **< 0.001** |
| OTU_1050 | 1.000 | **0.004** | **0.044** | 0.054 | 0.283 | 1.000 |  | 1.000 | 0.081 | 1.000 | **0.043** | 1.000 | 1.000 |  | 1.000 | 0.240 | 1.000 | **0.025** | 0.202 | 1.000 |
| OTU_1051 | 1.000 | 1.000 | 1.000 | 1.000 | 0.357 | 1.000 |  | 1.000 | 1.000 | 1.000 | 1.000 | 1.000 | 1.000 |  | 1.000 | **0.047** | **0.008** | 0.723 | 0.132 | 1.000 |
| OTU_1052 | 1.000 | 1.000 | 1.000 | 1.000 | 1.000 | 1.000 |  | 1.000 | 1.000 | 1.000 | 1.000 | 1.000 | 1.000 |  | 1.000 | 1.000 | 0.862 | 1.000 | 1.000 | 1.000 |
| OTU_1053 | 1.000 | 1.000 | 0.462 | 1.000 | 1.000 | 1.000 |  | 1.000 | 1.000 | 0.658 | 1.000 | 0.124 | 1.000 |  | 1.000 | 0.460 | 1.000 | 1.000 | 1.000 | 1.000 |
| OTU_1054 | 1.000 | 0.231 | 1.000 | 1.000 | 1.000 | 1.000 |  | 1.000 | **0.006** | 0.122 | **0.011** | 0.143 | 1.000 |  | 1.000 | 1.000 | 1.000 | 1.000 | 1.000 | 1.000 |
| OTU_1055 | 1.000 | 1.000 | 0.114 | 1.000 | 0.404 | **0.018** |  | 1.000 | 1.000 | 0.207 | 1.000 | 0.241 | 0.392 |  | 1.000 | 1.000 | 0.051 | 1.000 | 0.125 | 1.000 |
| OTU_1056 | **0.004** | **< 0.001** | **0.002** | 1.000 | 1.000 | 1.000 |  | **0.004** | **< 0.001** | **0.020** | 1.000 | 1.000 | 1.000 |  | **0.047** | **< 0.001** | **< 0.001** | 0.264 | 1.000 | 1.000 |
| OTU_1058 | 1.000 | 1.000 | 0.083 | 1.000 | 0.187 | 0.984 |  | 1.000 | 1.000 | 1.000 | 1.000 | 1.000 | 1.000 |  | 1.000 | 1.000 | 1.000 | 1.000 | 1.000 | 1.000 |
| OTU_1059 | 0.646 | 1.000 | 1.000 | 1.000 | 1.000 | 1.000 |  | 0.646 | 0.075 | 1.000 | 1.000 | 1.000 | 1.000 |  | 1.000 | 1.000 | 1.000 | 1.000 | 1.000 | 1.000 |
| OTU_106 | 1.000 | **< 0.001** | 0.544 | 0.981 | **< 0.001** | **< 0.001** |  | 1.000 | **< 0.001** | 1.000 | **0.043** | **0.002** | **< 0.001** |  | 0.360 | **< 0.001** | 0.165 | 1.000 | **< 0.001** | **< 0.001** |
| OTU_1060 | 0.228 | **< 0.001** | **< 0.001** | 1.000 | **0.013** | 1.000 |  | 0.228 | **< 0.001** | **< 0.001** | 1.000 | 0.271 | 1.000 |  | 1.000 | **0.031** | **< 0.001** | 1.000 | 0.246 | 1.000 |
| OTU_1061 | **0.044** | 0.088 | **0.003** | 1.000 | 1.000 | 1.000 |  | **0.044** | **0.035** | **< 0.001** | 1.000 | 1.000 | 0.796 |  | **0.014** | 0.056 | 0.126 | 1.000 | 1.000 | 1.000 |
| OTU_1063 | 1.000 | 1.000 | 1.000 | 1.000 | 0.152 | 1.000 |  | 1.000 | 0.673 | 1.000 | 1.000 | 0.258 | 1.000 |  | 0.770 | 1.000 | 1.000 | 1.000 | 0.092 | 1.000 |
| OTU_1065 | 0.546 | 0.061 | 0.790 | 1.000 | 1.000 | 1.000 |  | 0.546 | 1.000 | 1.000 | 1.000 | 1.000 | 1.000 |  | 1.000 | 1.000 | 1.000 | 1.000 | 1.000 | 1.000 |
| OTU_1066 | 1.000 | 1.000 | 0.172 | 1.000 | 0.165 | 1.000 |  | 1.000 | 0.864 | **< 0.001** | 1.000 | **0.034** | **0.015** |  | 1.000 | **0.005** | **0.046** | 1.000 | 1.000 | 1.000 |
| OTU_1067 | 1.000 | 1.000 | 1.000 | 1.000 | 0.086 | 1.000 |  | 1.000 | 0.548 | **0.037** | 1.000 | 0.669 | 1.000 |  | 1.000 | 1.000 | 0.052 | 1.000 | 0.332 | 1.000 |
| OTU_1068 | 1.000 | 1.000 | 1.000 | 1.000 | 1.000 | 1.000 |  | 1.000 | 1.000 | 1.000 | 0.071 | **0.023** | 1.000 |  | 1.000 | 0.334 | 0.916 | 0.437 | 0.873 | 1.000 |
| OTU_1069 | 1.000 | 1.000 | 1.000 | 1.000 | 1.000 | 1.000 |  | 1.000 | 0.497 | 1.000 | 1.000 | 1.000 | 1.000 |  | 1.000 | 1.000 | 1.000 | 1.000 | 1.000 | 1.000 |
| OTU_107 | 0.941 | **0.003** | 0.151 | 1.000 | 1.000 | 1.000 |  | 0.941 | **< 0.001** | **< 0.001** | 0.362 | 1.000 | 1.000 |  | 1.000 | 0.068 | 1.000 | 1.000 | 1.000 | 1.000 |
| OTU_1070 | 0.313 | 1.000 | 1.000 | 0.523 | **0.009** | 1.000 |  | 0.313 | 0.096 | **0.023** | 1.000 | 1.000 | 1.000 |  | 1.000 | 1.000 | 0.703 | 0.335 | **0.016** | 1.000 |
| OTU_1071 | 1.000 | 1.000 | 1.000 | 1.000 | 1.000 | 1.000 |  | 1.000 | 0.058 | 0.122 | 1.000 | 1.000 | 1.000 |  | 1.000 | 1.000 | 1.000 | 1.000 | 1.000 | 1.000 |
| OTU_1073 | 0.945 | 1.000 | 1.000 | 1.000 | 0.353 | 1.000 |  | 0.945 | 1.000 | 1.000 | 1.000 | 1.000 | 1.000 |  | 1.000 | 1.000 | 1.000 | 1.000 | 0.342 | 1.000 |
| OTU_1074 | 1.000 | 0.128 | 1.000 | 1.000 | 1.000 | 1.000 |  | 1.000 | 0.230 | 1.000 | 1.000 | 1.000 | 1.000 |  | 1.000 | 1.000 | 1.000 | 1.000 | 1.000 | 1.000 |
| OTU_1075 | 1.000 | 1.000 | 1.000 | 0.928 | 0.096 | 1.000 |  | 1.000 | 1.000 | 1.000 | 0.172 | 1.000 | 1.000 |  | 0.672 | 1.000 | 1.000 | **0.002** | 0.055 | 1.000 |
| OTU_1076 | 1.000 | 0.100 | 0.088 | 1.000 | 1.000 | 1.000 |  | 1.000 | **0.022** | **0.010** | 1.000 | 0.842 | 1.000 |  | 1.000 | 0.776 | 1.000 | 1.000 | 1.000 | 1.000 |
| OTU_1077 | 0.898 | **0.014** | 0.116 | 1.000 | 1.000 | 1.000 |  | 0.898 | 1.000 | 1.000 | 1.000 | 1.000 | 1.000 |  | 1.000 | 0.090 | 1.000 | 0.203 | 1.000 | 1.000 |
| OTU_1078 | 1.000 | 1.000 | 0.493 | 1.000 | 0.414 | 0.121 |  | 1.000 | 1.000 | **0.011** | 0.156 | 1.000 | 0.490 |  | 1.000 | 0.518 | 1.000 | 1.000 | 1.000 | 0.622 |
| OTU_1079 | 1.000 | 1.000 | 1.000 | 1.000 | 1.000 | 1.000 |  | 1.000 | 0.472 | 0.218 | 1.000 | 1.000 | 1.000 |  | 1.000 | 0.987 | 1.000 | 1.000 | 1.000 | 1.000 |
| OTU_108 | **< 0.001** | **< 0.001** | **< 0.001** | **< 0.001** | **< 0.001** | 1.000 |  | **< 0.001** | **< 0.001** | **< 0.001** | **< 0.001** | **< 0.001** | 1.000 |  | **< 0.001** | **< 0.001** | **< 0.001** | **< 0.001** | **< 0.001** | 1.000 |
| OTU_1080 | 1.000 | **0.029** | 1.000 | **0.020** | 1.000 | 0.261 |  | 1.000 | **< 0.001** | 0.862 | **0.026** | 1.000 | **< 0.001** |  | **0.003** | **< 0.001** | **0.009** | **< 0.001** | 1.000 | **< 0.001** |
| OTU_1081 | 0.141 | 1.000 | **< 0.001** | 1.000 | 1.000 | 0.139 |  | 0.141 | 0.607 | **0.017** | 1.000 | 0.639 | 1.000 |  | 1.000 | 1.000 | **< 0.001** | 1.000 | **< 0.001** | 0.283 |
| OTU_1082 | 1.000 | 0.787 | 1.000 | 1.000 | 1.000 | 1.000 |  | 1.000 | 1.000 | 1.000 | 1.000 | 1.000 | 1.000 |  | 1.000 | 1.000 | 1.000 | 1.000 | 1.000 | 1.000 |
| OTU_1083 | 1.000 | 1.000 | 1.000 | 1.000 | 1.000 | 1.000 |  | 1.000 | 0.459 | 0.225 | 0.617 | 0.261 | 1.000 |  | 1.000 | 1.000 | 0.226 | 1.000 | 0.600 | 1.000 |
| OTU_1084 | 1.000 | 1.000 | 1.000 | 1.000 | 0.877 | 1.000 |  | 1.000 | 1.000 | 1.000 | 1.000 | 1.000 | 1.000 |  | 0.804 | 0.467 | 1.000 | 1.000 | 1.000 | 1.000 |
| OTU_1085 | 1.000 | 1.000 | 1.000 | 1.000 | 0.470 | 0.908 |  | 1.000 | 1.000 | 1.000 | 0.091 | 0.146 | 1.000 |  | 1.000 | 1.000 | 0.206 | 1.000 | 0.305 | 1.000 |
| OTU_1087 | 1.000 | 0.361 | **0.004** | 0.761 | **0.007** | 1.000 |  | 1.000 | **0.022** | **0.011** | 0.738 | 0.504 | 1.000 |  | 1.000 | **< 0.001** | **< 0.001** | **0.029** | **0.001** | 1.000 |
| OTU_1088 | 1.000 | 1.000 | 1.000 | 1.000 | 1.000 | 1.000 |  | 1.000 | 1.000 | 1.000 | 1.000 | 1.000 | 1.000 |  | 1.000 | 1.000 | 1.000 | 1.000 | 1.000 | 1.000 |
| OTU_109 | **0.002** | **< 0.001** | 1.000 | 1.000 | **< 0.001** | **< 0.001** |  | **0.002** | **< 0.001** | 0.180 | 0.949 | 0.324 | **< 0.001** |  | 0.164 | **< 0.001** | 1.000 | 1.000 | **< 0.001** | **< 0.001** |
| OTU_1091 | 0.074 | 1.000 | 1.000 | 0.340 | **< 0.001** | 1.000 |  | 0.074 | 0.294 | 1.000 | 0.647 | 1.000 | 1.000 |  | 1.000 | 0.073 | 0.358 | 1.000 | 1.000 | 1.000 |
| OTU_1092 | 1.000 | 1.000 | 1.000 | 1.000 | 1.000 | 1.000 |  | 1.000 | 0.762 | 1.000 | 0.992 | 1.000 | 0.637 |  | 1.000 | **0.046** | 1.000 | 1.000 | 1.000 | 1.000 |
| OTU_1093 | 1.000 | 1.000 | 0.064 | 1.000 | 0.442 | 0.402 |  | 1.000 | 1.000 | 0.055 | 1.000 | 0.458 | 0.816 |  | 1.000 | 1.000 | 0.084 | 1.000 | 0.620 | 0.754 |
| OTU_1094 | 1.000 | 0.169 | 0.059 | 1.000 | 1.000 | 1.000 |  | 1.000 | 0.183 | 0.097 | 1.000 | 1.000 | 1.000 |  | 1.000 | 1.000 | 1.000 | 1.000 | 1.000 | 1.000 |
| OTU_1095 | 0.115 | **0.002** | 0.169 | 1.000 | 1.000 | 1.000 |  | 0.115 | **0.014** | **0.015** | 0.991 | 1.000 | 1.000 |  | 0.578 | **0.013** | 0.268 | 1.000 | 1.000 | 1.000 |
| OTU_1096 | 1.000 | 1.000 | 1.000 | 1.000 | 1.000 | 1.000 |  | 1.000 | **< 0.001** | **< 0.001** | **< 0.001** | **0.048** | 1.000 |  | 1.000 | 1.000 | 1.000 | 1.000 | 1.000 | 1.000 |
| OTU_1097 | 1.000 | 1.000 | 1.000 | 1.000 | 1.000 | 1.000 |  | 1.000 | **0.006** | 0.109 | **< 0.001** | **0.001** | 1.000 |  | 1.000 | 0.107 | 0.151 | 0.780 | 0.835 | 1.000 |
| OTU_1098 | 0.666 | 0.490 | 0.907 | 1.000 | **0.001** | **0.003** |  | 0.666 | 1.000 | **0.004** | 1.000 | **0.006** | 0.091 |  | 1.000 | 1.000 | 0.094 | 1.000 | **0.003** | 0.066 |
| OTU_1099 | 1.000 | 1.000 | 1.000 | 1.000 | 1.000 | 1.000 |  | 1.000 | 1.000 | 1.000 | 1.000 | 1.000 | 1.000 |  | 1.000 | 1.000 | 1.000 | 1.000 | 1.000 | 1.000 |
| OTU_11 | **0.005** | 1.000 | 0.326 | 0.292 | 1.000 | 1.000 |  | **0.005** | 1.000 | 0.213 | 1.000 | 1.000 | 1.000 |  | **0.015** | **0.021** | 0.224 | 1.000 | 1.000 | 1.000 |
| OTU_110 | **< 0.001** | **< 0.001** | **< 0.001** | 0.770 | 1.000 | 1.000 |  | **< 0.001** | **< 0.001** | **< 0.001** | **< 0.001** | 0.129 | 0.064 |  | 0.365 | **< 0.001** | **< 0.001** | 0.079 | 1.000 | 1.000 |
| OTU_1100 | 1.000 | 1.000 | 1.000 | 1.000 | 1.000 | 1.000 |  | 1.000 | 1.000 | 1.000 | 1.000 | 1.000 | 1.000 |  | 1.000 | 1.000 | 1.000 | 1.000 | 1.000 | 1.000 |
| OTU_1101 | 1.000 | 1.000 | 0.175 | 0.916 | **0.017** | 1.000 |  | 1.000 | 1.000 | 0.757 | 1.000 | 0.884 | 1.000 |  | 1.000 | 1.000 | 1.000 | 1.000 | 0.140 | 1.000 |
| OTU_1103 | 1.000 | **0.042** | **< 0.001** | 1.000 | **0.014** | 0.908 |  | 1.000 | 0.063 | **0.007** | 1.000 | 0.933 | 1.000 |  | 1.000 | 1.000 | 1.000 | 1.000 | 1.000 | 1.000 |
| OTU_1105 | 1.000 | 1.000 | 1.000 | 1.000 | 1.000 | 1.000 |  | 1.000 | 1.000 | 1.000 | 1.000 | 0.213 | 1.000 |  | 1.000 | 1.000 | 1.000 | 1.000 | 1.000 | 1.000 |
| OTU_1106 | 1.000 | 0.088 | 0.058 | 1.000 | 1.000 | 1.000 |  | 1.000 | 1.000 | 0.961 | 1.000 | 1.000 | 1.000 |  | 1.000 | 0.289 | 1.000 | 1.000 | 1.000 | 1.000 |
| OTU_1107 | 1.000 | 0.982 | 0.108 | **0.047** | **0.002** | 1.000 |  | 1.000 | 1.000 | **< 0.001** | 1.000 | **0.002** | 0.284 |  | 1.000 | 1.000 | **0.027** | 1.000 | 0.070 | 1.000 |
| OTU_1108 | 1.000 | 1.000 | 0.480 | 1.000 | 0.904 | 1.000 |  | 1.000 | 1.000 | 0.685 | 1.000 | 1.000 | 1.000 |  | 1.000 | 1.000 | 1.000 | 1.000 | 1.000 | 1.000 |
| OTU_1109 | 1.000 | 1.000 | 0.458 | 1.000 | 1.000 | 1.000 |  | 1.000 | 0.241 | 1.000 | 1.000 | 0.092 | 1.000 |  | 1.000 | 1.000 | 1.000 | 1.000 | 1.000 | 1.000 |
| OTU_111 | 1.000 | **< 0.001** | **< 0.001** | **< 0.001** | **< 0.001** | 1.000 |  | 1.000 | **< 0.001** | **< 0.001** | **< 0.001** | **< 0.001** | 1.000 |  | 1.000 | **< 0.001** | **< 0.001** | **< 0.001** | **< 0.001** | 1.000 |
| OTU_1111 | 1.000 | 1.000 | 1.000 | 1.000 | 1.000 | 1.000 |  | 1.000 | 1.000 | 1.000 | 1.000 | 1.000 | 1.000 |  | 1.000 | 1.000 | 1.000 | 1.000 | 0.835 | 1.000 |
| OTU_1112 | 1.000 | 1.000 | 1.000 | 1.000 | 1.000 | 1.000 |  | 1.000 | 1.000 | 1.000 | 1.000 | 1.000 | 1.000 |  | 0.549 | 0.145 | 0.060 | 1.000 | 1.000 | 1.000 |
| OTU_1114 | **< 0.001** | **< 0.001** | **< 0.001** | 1.000 | 1.000 | 1.000 |  | **< 0.001** | 0.095 | 1.000 | 1.000 | 1.000 | 1.000 |  | 1.000 | 0.802 | 1.000 | 1.000 | 1.000 | 1.000 |
| OTU_1116 | 1.000 | 0.103 | **0.044** | 1.000 | 1.000 | 1.000 |  | 1.000 | 1.000 | 1.000 | **0.029** | 0.127 | 1.000 |  | 1.000 | 1.000 | 1.000 | 1.000 | 1.000 | 1.000 |
| OTU_1117 | 1.000 | 1.000 | 1.000 | 1.000 | **0.026** | 0.057 |  | 1.000 | 1.000 | **< 0.001** | 1.000 | 0.098 | **0.025** |  | 1.000 | 1.000 | 0.175 | 1.000 | **0.041** | 1.000 |
| OTU_1118 | **0.036** | 1.000 | 1.000 | 0.099 | 0.477 | 1.000 |  | **0.036** | 1.000 | 1.000 | 1.000 | 1.000 | 1.000 |  | 1.000 | 1.000 | 1.000 | 1.000 | 1.000 | 1.000 |
| OTU_1119 | 0.177 | 0.173 | 1.000 | 1.000 | 1.000 | 1.000 |  | 0.177 | 1.000 | 1.000 | 1.000 | 1.000 | 1.000 |  | 1.000 | 1.000 | 1.000 | 1.000 | 1.000 | 1.000 |
| OTU_112 | **< 0.001** | **< 0.001** | 0.208 | **< 0.001** | **0.019** | **< 0.001** |  | **< 0.001** | **< 0.001** | 1.000 | **< 0.001** | 1.000 | **< 0.001** |  | **0.017** | **< 0.001** | 0.666 | **< 0.001** | 1.000 | **< 0.001** |
| OTU_1120 | 1.000 | 1.000 | 1.000 | 1.000 | 1.000 | 1.000 |  | 1.000 | 1.000 | 1.000 | 1.000 | 1.000 | 1.000 |  | 0.277 | 1.000 | 1.000 | **0.029** | **0.017** | 1.000 |
| OTU_1121 | 1.000 | 1.000 | 1.000 | 1.000 | 0.410 | 1.000 |  | 1.000 | 1.000 | 1.000 | 1.000 | 1.000 | 1.000 |  | 1.000 | 1.000 | 1.000 | 1.000 | 1.000 | 1.000 |
| OTU_1122 | 1.000 | 1.000 | **< 0.001** | 0.615 | **< 0.001** | 0.132 |  | 1.000 | **< 0.001** | **< 0.001** | 0.219 | **< 0.001** | 1.000 |  | 1.000 | 1.000 | **0.012** | 0.070 | **< 0.001** | 1.000 |
| OTU_1123 | 1.000 | 1.000 | 0.115 | 0.536 | **0.003** | 1.000 |  | 1.000 | 0.064 | **0.003** | **0.001** | **< 0.001** | 1.000 |  | 0.117 | 1.000 | 1.000 | 1.000 | 0.411 | 1.000 |
| OTU_1124 | 1.000 | 1.000 | 1.000 | 1.000 | 1.000 | 1.000 |  | 1.000 | 1.000 | 0.249 | 1.000 | 0.293 | 1.000 |  | 1.000 | 0.555 | 1.000 | 1.000 | 1.000 | 1.000 |
| OTU_1125 | **0.001** | **< 0.001** | **< 0.001** | 1.000 | 1.000 | 1.000 |  | **0.001** | **< 0.001** | **< 0.001** | **< 0.001** | 0.226 | 0.736 |  | 1.000 | **< 0.001** | **0.021** | 0.192 | 1.000 | 1.000 |
| OTU_1126 | 1.000 | 1.000 | 1.000 | 1.000 | 1.000 | 1.000 |  | 1.000 | 1.000 | 1.000 | 1.000 | 1.000 | 1.000 |  | 0.693 | 0.460 | 1.000 | 1.000 | 1.000 | 1.000 |
| OTU_1127 | 1.000 | 0.167 | **0.029** | 1.000 | 1.000 | 1.000 |  | 1.000 | 1.000 | 1.000 | 1.000 | 1.000 | 1.000 |  | 1.000 | 0.415 | 0.245 | 1.000 | 1.000 | 1.000 |
| OTU_1128 | 1.000 | 1.000 | 0.385 | 1.000 | 0.404 | 0.744 |  | 1.000 | 0.871 | **0.033** | 1.000 | **0.041** | 1.000 |  | 1.000 | 1.000 | 0.533 | 1.000 | 0.610 | 1.000 |
| OTU_1129 | 1.000 | 1.000 | 1.000 | 1.000 | 1.000 | 1.000 |  | 1.000 | **0.021** | 1.000 | 1.000 | **0.040** | 0.066 |  | 1.000 | 1.000 | 1.000 | 1.000 | 1.000 | 1.000 |
| OTU_113 | 0.723 | 0.525 | **< 0.001** | **< 0.001** | **< 0.001** | **< 0.001** |  | 0.723 | 0.159 | **< 0.001** | **< 0.001** | **< 0.001** | **< 0.001** |  | 1.000 | 0.192 | **< 0.001** | **0.013** | **< 0.001** | **< 0.001** |
| OTU_1130 | 1.000 | 1.000 | 1.000 | 1.000 | 1.000 | 1.000 |  | 1.000 | **0.002** | **0.001** | **0.004** | **0.001** | 1.000 |  | 1.000 | 1.000 | 1.000 | 1.000 | 1.000 | 1.000 |
| OTU_1132 | 0.083 | 0.114 | 0.468 | 1.000 | 1.000 | 1.000 |  | 0.083 | 1.000 | 1.000 | 0.594 | 1.000 | 1.000 |  | **0.011** | 0.779 | 1.000 | 1.000 | **0.006** | 1.000 |
| OTU_1135 | 1.000 | 1.000 | 1.000 | 0.829 | 0.148 | 1.000 |  | 1.000 | 1.000 | **0.023** | 1.000 | 0.054 | 0.507 |  | 1.000 | 0.226 | **0.002** | 0.626 | **0.005** | 1.000 |
| OTU_1138 | 0.566 | 0.958 | 1.000 | 1.000 | 0.275 | 1.000 |  | 0.566 | 0.365 | 0.953 | 0.946 | 1.000 | 1.000 |  | 1.000 | 1.000 | 1.000 | 1.000 | 1.000 | 1.000 |
| OTU_114 | 1.000 | **< 0.001** | **< 0.001** | **0.020** | **< 0.001** | 0.056 |  | 1.000 | **< 0.001** | **< 0.001** | 0.353 | **< 0.001** | 0.554 |  | 0.426 | **0.025** | **0.003** | 1.000 | 1.000 | 1.000 |
| OTU_1140 | 1.000 | 1.000 | 1.000 | 0.406 | 1.000 | 0.147 |  | 1.000 | 0.149 | 0.495 | 1.000 | 1.000 | 1.000 |  | 1.000 | 1.000 | 1.000 | 1.000 | 1.000 | 1.000 |
| OTU_1142 | 1.000 | 1.000 | 1.000 | 1.000 | 1.000 | 1.000 |  | 1.000 | 1.000 | 1.000 | 1.000 | 1.000 | 1.000 |  | 1.000 | 1.000 | 1.000 | 1.000 | 1.000 | 1.000 |
| OTU_1143 | 1.000 | 1.000 | 1.000 | 1.000 | 1.000 | 1.000 |  | 1.000 | 1.000 | **0.017** | 1.000 | 1.000 | 1.000 |  | 1.000 | 1.000 | 1.000 | 1.000 | 1.000 | 1.000 |
| OTU_1144 | 1.000 | 1.000 | 0.472 | 0.568 | **0.005** | 1.000 |  | 1.000 | 0.057 | **0.015** | 1.000 | 1.000 | 1.000 |  | 1.000 | 1.000 | 0.087 | 1.000 | 0.894 | 1.000 |
| OTU_1145 | 1.000 | 1.000 | 0.097 | 1.000 | 0.075 | 1.000 |  | 1.000 | 1.000 | 0.393 | 1.000 | 0.468 | 1.000 |  | 1.000 | 1.000 | 1.000 | 1.000 | 1.000 | 1.000 |
| OTU_1146 | 1.000 | **0.044** | 1.000 | 1.000 | 1.000 | 0.089 |  | 1.000 | 1.000 | 0.099 | 1.000 | 0.117 | 0.197 |  | 1.000 | 0.116 | 1.000 | 1.000 | 1.000 | 0.074 |
| OTU_1147 | 1.000 | 1.000 | 1.000 | 1.000 | 1.000 | 1.000 |  | 1.000 | 1.000 | 1.000 | 1.000 | 1.000 | 1.000 |  | 1.000 | 1.000 | 1.000 | 1.000 | 1.000 | 1.000 |
| OTU_1148 | 1.000 | 1.000 | 1.000 | 1.000 | 1.000 | 1.000 |  | 1.000 | 1.000 | 1.000 | 1.000 | 1.000 | 1.000 |  | 1.000 | 1.000 | 1.000 | 1.000 | 1.000 | 1.000 |
| OTU_1149 | 1.000 | 1.000 | 1.000 | 1.000 | 1.000 | 1.000 |  | 1.000 | 0.723 | 0.176 | 0.949 | 0.207 | 1.000 |  | 1.000 | 0.193 | 1.000 | **0.047** | 1.000 | 0.826 |
| OTU_115 | 0.704 | 1.000 | **0.025** | 1.000 | 1.000 | 1.000 |  | 0.704 | **< 0.001** | **< 0.001** | 1.000 | 1.000 | 1.000 |  | 1.000 | 0.639 | 0.462 | 1.000 | 1.000 | 1.000 |
| OTU_1150 | **< 0.001** | 1.000 | 1.000 | **0.042** | **0.003** | 1.000 |  | **< 0.001** | **< 0.001** | 1.000 | 0.077 | 1.000 | **0.028** |  | **0.035** | 1.000 | 1.000 | 0.798 | 0.053 | 1.000 |
| OTU_1151 | 1.000 | 1.000 | 1.000 | **0.049** | 0.208 | 1.000 |  | 1.000 | 1.000 | 0.335 | 1.000 | 1.000 | 1.000 |  | 1.000 | 1.000 | 1.000 | 1.000 | 1.000 | 1.000 |
| OTU_1153 | 1.000 | 1.000 | 1.000 | 1.000 | 1.000 | 1.000 |  | 1.000 | 1.000 | 1.000 | 1.000 | 1.000 | 1.000 |  | 1.000 | 1.000 | 1.000 | 1.000 | 0.825 | 1.000 |
| OTU_1154 | 1.000 | 1.000 | 1.000 | 1.000 | 1.000 | 1.000 |  | 1.000 | 0.174 | 1.000 | 1.000 | 0.617 | 1.000 |  | 1.000 | 1.000 | 1.000 | 1.000 | 1.000 | 1.000 |
| OTU_1155 | 0.953 | 0.856 | 1.000 | 1.000 | 1.000 | 1.000 |  | 0.953 | 1.000 | 1.000 | 0.074 | 0.898 | 1.000 |  | 1.000 | 1.000 | 1.000 | 1.000 | 1.000 | 1.000 |
| OTU_1156 | 1.000 | 0.405 | 1.000 | 1.000 | 1.000 | 1.000 |  | 1.000 | 1.000 | 0.941 | 1.000 | 1.000 | 1.000 |  | 1.000 | 1.000 | 1.000 | 1.000 | 1.000 | 1.000 |
| OTU_1158 | 1.000 | 0.515 | 0.448 | 0.897 | 0.647 | 1.000 |  | 1.000 | 1.000 | 0.558 | 1.000 | 1.000 | 1.000 |  | 1.000 | 0.445 | **0.047** | 0.189 | **0.014** | 1.000 |
| OTU_116 | 0.156 | 0.432 | **< 0.001** | 1.000 | **< 0.001** | **< 0.001** |  | 0.156 | 1.000 | **< 0.001** | 1.000 | **< 0.001** | **< 0.001** |  | 1.000 | 1.000 | **< 0.001** | 1.000 | **< 0.001** | **< 0.001** |
| OTU_1160 | 1.000 | 1.000 | 1.000 | 1.000 | 0.905 | 1.000 |  | 1.000 | 1.000 | 1.000 | 1.000 | **0.009** | 1.000 |  | 1.000 | 1.000 | 0.178 | 1.000 | 0.177 | 0.975 |
| OTU_1161 | 1.000 | 1.000 | **< 0.001** | 1.000 | **< 0.001** | **0.001** |  | 1.000 | 1.000 | 1.000 | 1.000 | 1.000 | 1.000 |  | 1.000 | 1.000 | 1.000 | 1.000 | 1.000 | 1.000 |
| OTU_1162 | 1.000 | 1.000 | 1.000 | 1.000 | 1.000 | 1.000 |  | 1.000 | 1.000 | 1.000 | 1.000 | 0.504 | 1.000 |  | 1.000 | 1.000 | 1.000 | 1.000 | 1.000 | 1.000 |
| OTU_1163 | 0.281 | **0.002** | **0.014** | 1.000 | 1.000 | 1.000 |  | 0.281 | **0.009** | **0.003** | 1.000 | 1.000 | 1.000 |  | 0.704 | **0.009** | **0.037** | 1.000 | 1.000 | 1.000 |
| OTU_1164 | 1.000 | 1.000 | 1.000 | 1.000 | 1.000 | 1.000 |  | 1.000 | 1.000 | 1.000 | 1.000 | 1.000 | 1.000 |  | 1.000 | 1.000 | 1.000 | 1.000 | 1.000 | 1.000 |
| OTU_1166 | 1.000 | 0.352 | **0.003** | 1.000 | 0.216 | 1.000 |  | 1.000 | 1.000 | **< 0.001** | 0.199 | 0.456 | **< 0.001** |  | 1.000 | 1.000 | **0.002** | 1.000 | 0.098 | 0.069 |
| OTU_1167 | 0.702 | 0.503 | 1.000 | 1.000 | 1.000 | 1.000 |  | 0.702 | 1.000 | 1.000 | 1.000 | 1.000 | 1.000 |  | 1.000 | 0.488 | 1.000 | 1.000 | 1.000 | 1.000 |
| OTU_1168 | 0.376 | **< 0.001** | 1.000 | 0.679 | 1.000 | 0.057 |  | 0.376 | **0.004** | 1.000 | 0.193 | 1.000 | 0.125 |  | 1.000 | 0.799 | 1.000 | 0.396 | 1.000 | 0.159 |
| OTU_1169 | 1.000 | 1.000 | 1.000 | 1.000 | 1.000 | 1.000 |  | 1.000 | 1.000 | 1.000 | 1.000 | 1.000 | 1.000 |  | 1.000 | 1.000 | 1.000 | 1.000 | 1.000 | 1.000 |
| OTU_117 | 1.000 | **< 0.001** | **< 0.001** | **< 0.001** | 0.070 | 0.552 |  | 1.000 | **< 0.001** | **< 0.001** | **0.004** | 1.000 | 1.000 |  | **0.047** | **< 0.001** | **< 0.001** | **0.012** | 1.000 | 1.000 |
| OTU_1170 | 1.000 | 1.000 | 1.000 | 1.000 | 1.000 | 1.000 |  | 1.000 | 1.000 | 1.000 | 0.362 | 0.566 | 1.000 |  | 1.000 | 1.000 | 1.000 | 1.000 | 1.000 | 1.000 |
| OTU_1171 | **< 0.001** | **< 0.001** | 1.000 | 1.000 | **0.018** | 0.052 |  | **< 0.001** | **0.021** | 1.000 | 1.000 | 1.000 | 1.000 |  | 1.000 | **< 0.001** | 1.000 | 0.227 | 1.000 | 0.141 |
| OTU_1172 | 1.000 | **< 0.001** | **0.003** | **< 0.001** | 1.000 | 0.301 |  | 1.000 | **< 0.001** | **< 0.001** | **< 0.001** | **< 0.001** | **0.004** |  | 1.000 | **0.005** | 1.000 | **0.002** | 0.794 | 1.000 |
| OTU_1173 | 1.000 | **0.031** | 0.160 | 1.000 | 1.000 | 1.000 |  | 1.000 | 0.626 | 1.000 | 0.145 | 0.648 | 1.000 |  | 1.000 | **0.002** | 0.054 | 0.693 | 1.000 | 1.000 |
| OTU_1174 | 1.000 | 1.000 | 1.000 | 1.000 | 1.000 | 1.000 |  | 1.000 | 1.000 | 1.000 | 1.000 | 1.000 | 1.000 |  | 1.000 | 0.057 | 1.000 | 1.000 | 1.000 | 1.000 |
| OTU_1175 | 0.240 | 1.000 | 0.126 | 1.000 | 1.000 | 1.000 |  | 0.240 | 1.000 | 1.000 | 1.000 | 1.000 | 1.000 |  | 1.000 | 0.635 | 1.000 | 1.000 | 1.000 | 1.000 |
| OTU_1176 | 1.000 | 0.217 | 1.000 | 1.000 | 1.000 | 1.000 |  | 1.000 | 0.569 | 0.052 | 1.000 | 1.000 | 1.000 |  | 1.000 | 1.000 | 1.000 | 1.000 | 1.000 | 1.000 |
| OTU_1177 | 1.000 | 0.286 | 1.000 | 1.000 | 1.000 | 1.000 |  | 1.000 | 1.000 | 0.151 | 1.000 | 1.000 | 1.000 |  | 1.000 | 1.000 | 1.000 | 1.000 | 1.000 | 1.000 |
| OTU_1178 | 1.000 | 1.000 | 1.000 | 1.000 | 1.000 | 1.000 |  | 1.000 | 1.000 | 1.000 | 1.000 | 1.000 | 1.000 |  | 1.000 | 0.754 | 1.000 | 1.000 | 1.000 | 1.000 |
| OTU_118 | 0.115 | 1.000 | 0.709 | 1.000 | **< 0.001** | **0.016** |  | 0.115 | 1.000 | 1.000 | 0.876 | **< 0.001** | 0.588 |  | **0.004** | 0.150 | 1.000 | 1.000 | **< 0.001** | 0.093 |
| OTU_1180 | 1.000 | 0.856 | 1.000 | 0.060 | 0.244 | 1.000 |  | 1.000 | 1.000 | 1.000 | 1.000 | 1.000 | 1.000 |  | 1.000 | 1.000 | 0.790 | 0.544 | 0.135 | 1.000 |
| OTU_1181 | 0.621 | 1.000 | 0.153 | 1.000 | **< 0.001** | 0.079 |  | 0.621 | 1.000 | **< 0.001** | 1.000 | **< 0.001** | 0.196 |  | 1.000 | 1.000 | **0.011** | 1.000 | 0.420 | 1.000 |
| OTU_1182 | 1.000 | 1.000 | 1.000 | 1.000 | 1.000 | 1.000 |  | 1.000 | **0.014** | 1.000 | 1.000 | 0.106 | **0.008** |  | 1.000 | 1.000 | 1.000 | 1.000 | 1.000 | 1.000 |
| OTU_1183 | 1.000 | 1.000 | 1.000 | 1.000 | 1.000 | 1.000 |  | 1.000 | 1.000 | 1.000 | 1.000 | 1.000 | 1.000 |  | 1.000 | 1.000 | 1.000 | 1.000 | 1.000 | 1.000 |
| OTU_1187 | 1.000 | 1.000 | 1.000 | 1.000 | 1.000 | 1.000 |  | 1.000 | 1.000 | 1.000 | 0.750 | 1.000 | 1.000 |  | 1.000 | 1.000 | 1.000 | 1.000 | 1.000 | 1.000 |
| OTU_1189 | **0.036** | **0.022** | 0.859 | 1.000 | 1.000 | 1.000 |  | **0.036** | 1.000 | 1.000 | 1.000 | 1.000 | 1.000 |  | 1.000 | 1.000 | 1.000 | 1.000 | 1.000 | 1.000 |
| OTU_119 | **0.031** | **< 0.001** | **< 0.001** | 1.000 | 1.000 | 1.000 |  | **0.031** | **< 0.001** | **< 0.001** | 0.070 | 0.070 | 1.000 |  | 0.079 | **< 0.001** | **< 0.001** | 0.424 | 1.000 | 1.000 |
| OTU_1192 | 1.000 | **0.014** | **< 0.001** | 1.000 | 0.096 | 1.000 |  | 1.000 | 1.000 | **0.004** | 1.000 | **0.008** | 1.000 |  | 1.000 | **0.031** | **< 0.001** | **0.005** | **< 0.001** | 1.000 |
| OTU_1193 | 1.000 | 1.000 | 1.000 | 1.000 | 1.000 | 1.000 |  | 1.000 | 1.000 | 1.000 | 1.000 | 1.000 | 1.000 |  | 1.000 | 1.000 | 1.000 | 1.000 | 1.000 | 1.000 |
| OTU_1194 | 0.152 | **0.009** | 0.154 | 1.000 | 1.000 | 1.000 |  | 0.152 | 0.111 | **0.017** | 1.000 | 1.000 | 1.000 |  | 0.053 | 0.073 | **0.013** | 1.000 | 1.000 | 1.000 |
| OTU_1195 | 1.000 | 1.000 | 1.000 | 1.000 | 1.000 | 1.000 |  | 1.000 | 1.000 | 1.000 | 1.000 | 1.000 | 1.000 |  | 1.000 | 1.000 | 1.000 | 1.000 | 1.000 | 1.000 |
| OTU_1198 | 1.000 | 1.000 | 1.000 | 1.000 | 1.000 | 1.000 |  | 1.000 | 1.000 | 1.000 | 1.000 | 1.000 | 1.000 |  | 1.000 | 1.000 | 1.000 | 1.000 | 1.000 | 1.000 |
| OTU_1199 | 1.000 | **0.004** | 1.000 | 0.058 | 1.000 | 0.150 |  | 1.000 | 1.000 | 1.000 | 1.000 | 1.000 | 1.000 |  | 1.000 | 0.491 | 0.992 | 1.000 | 1.000 | 1.000 |
| OTU_12 | 1.000 | 0.112 | 1.000 | **0.001** | 1.000 | **< 0.001** |  | 1.000 | **0.033** | 1.000 | **< 0.001** | **< 0.001** | 0.788 |  | 1.000 | **0.005** | 1.000 | **< 0.001** | 0.144 | 1.000 |
| OTU_1200 | 1.000 | 1.000 | 1.000 | 1.000 | 1.000 | 1.000 |  | 1.000 | 1.000 | 1.000 | 1.000 | 1.000 | 1.000 |  | 1.000 | 1.000 | 1.000 | 1.000 | 1.000 | 1.000 |
| OTU_1201 | 1.000 | 1.000 | 0.188 | 1.000 | 0.154 | 1.000 |  | 1.000 | 1.000 | 0.108 | 1.000 | 1.000 | 1.000 |  | 1.000 | 1.000 | 1.000 | 1.000 | 0.385 | 1.000 |
| OTU_1203 | 1.000 | 1.000 | 1.000 | 1.000 | 1.000 | 1.000 |  | 1.000 | 1.000 | 1.000 | 1.000 | 1.000 | 1.000 |  | 1.000 | 1.000 | 1.000 | 1.000 | 1.000 | 1.000 |
| OTU_1204 | 1.000 | 1.000 | 1.000 | 1.000 | 1.000 | 1.000 |  | 1.000 | 1.000 | 1.000 | 1.000 | 1.000 | 1.000 |  | 1.000 | 1.000 | 1.000 | 1.000 | 1.000 | 1.000 |
| OTU_1205 | 1.000 | 1.000 | 0.075 | 1.000 | 1.000 | 1.000 |  | 1.000 | **0.049** | **0.010** | 1.000 | 1.000 | 1.000 |  | 1.000 | 0.552 | 1.000 | 1.000 | 1.000 | 1.000 |
| OTU_1206 | 1.000 | 1.000 | 1.000 | 1.000 | 0.490 | 1.000 |  | 1.000 | 1.000 | 0.919 | 1.000 | 1.000 | 1.000 |  | 1.000 | 1.000 | 1.000 | 1.000 | 0.621 | 1.000 |
| OTU_1207 | 0.798 | 1.000 | 1.000 | 1.000 | 1.000 | 1.000 |  | 0.798 | 1.000 | 1.000 | 1.000 | 1.000 | 1.000 |  | 1.000 | 1.000 | 1.000 | 1.000 | 1.000 | 1.000 |
| OTU_121 | **0.006** | 1.000 | **< 0.001** | 0.347 | **< 0.001** | **< 0.001** |  | **0.006** | 1.000 | **0.001** | 1.000 | **< 0.001** | **< 0.001** |  | 1.000 | 1.000 | **< 0.001** | 1.000 | **< 0.001** | **< 0.001** |
| OTU_1210 | 1.000 | 0.055 | 0.457 | 1.000 | 1.000 | 1.000 |  | 1.000 | **0.010** | **0.004** | 1.000 | 1.000 | 1.000 |  | 1.000 | 0.988 | 1.000 | 1.000 | 1.000 | 1.000 |
| OTU_1212 | 1.000 | 1.000 | 1.000 | 1.000 | 0.815 | 1.000 |  | 1.000 | 1.000 | 1.000 | 1.000 | 1.000 | 1.000 |  | 1.000 | 0.645 | 0.467 | 1.000 | 0.820 | 1.000 |
| OTU_1213 | 1.000 | 0.687 | 1.000 | 1.000 | 1.000 | 0.822 |  | 1.000 | 1.000 | 1.000 | 1.000 | 1.000 | 0.341 |  | 1.000 | 1.000 | 1.000 | 1.000 | 1.000 | 1.000 |
| OTU_1216 | 1.000 | 0.063 | 1.000 | **0.003** | 0.096 | 1.000 |  | 1.000 | 1.000 | 1.000 | 1.000 | 1.000 | 1.000 |  | 1.000 | 1.000 | 1.000 | 1.000 | 1.000 | 1.000 |
| OTU_1219 | 0.076 | 0.508 | **0.045** | 1.000 | 1.000 | 1.000 |  | 0.076 | 1.000 | 0.731 | 1.000 | 1.000 | 1.000 |  | 1.000 | 1.000 | 1.000 | 1.000 | 1.000 | 1.000 |
| OTU_122 | 1.000 | 1.000 | **< 0.001** | 1.000 | **< 0.001** | **< 0.001** |  | 1.000 | 1.000 | **< 0.001** | 1.000 | **< 0.001** | **< 0.001** |  | 1.000 | 1.000 | **< 0.001** | 1.000 | **< 0.001** | **< 0.001** |
| OTU_1220 | 0.729 | 1.000 | 1.000 | 1.000 | 1.000 | 1.000 |  | 0.729 | 1.000 | 1.000 | 1.000 | 0.798 | 1.000 |  | 1.000 | 1.000 | 1.000 | 1.000 | 1.000 | 1.000 |
| OTU_1222 | 1.000 | 1.000 | 1.000 | 1.000 | 1.000 | 1.000 |  | 1.000 | 0.540 | 1.000 | **0.007** | 0.083 | 1.000 |  | 1.000 | 1.000 | 1.000 | 1.000 | 1.000 | 1.000 |
| OTU_1224 | 1.000 | 1.000 | 1.000 | 1.000 | 0.423 | 1.000 |  | 1.000 | **0.012** | **0.005** | 0.769 | 0.359 | 1.000 |  | 1.000 | 1.000 | 1.000 | 1.000 | 0.288 | 1.000 |
| OTU_1226 | 1.000 | 1.000 | 1.000 | 1.000 | 1.000 | 1.000 |  | 1.000 | 1.000 | 1.000 | 1.000 | 1.000 | 1.000 |  | 1.000 | 1.000 | 1.000 | 1.000 | 0.174 | 1.000 |
| OTU_1227 | 1.000 | 0.782 | 1.000 | 0.736 | 0.842 | 1.000 |  | 1.000 | 1.000 | 1.000 | 1.000 | 1.000 | 1.000 |  | 1.000 | 1.000 | 1.000 | 1.000 | 1.000 | 1.000 |
| OTU_1228 | 1.000 | 0.081 | 0.070 | 0.143 | 0.095 | 1.000 |  | 1.000 | 0.782 | 1.000 | 1.000 | 1.000 | 1.000 |  | 1.000 | 0.597 | 1.000 | 1.000 | 1.000 | 1.000 |
| OTU_1229 | 1.000 | 1.000 | 1.000 | 1.000 | 1.000 | 1.000 |  | 1.000 | 1.000 | 1.000 | 1.000 | 1.000 | 1.000 |  | 1.000 | 1.000 | 1.000 | 1.000 | 1.000 | 1.000 |
| OTU_123 | 1.000 | **< 0.001** | **0.030** | **0.024** | 1.000 | 1.000 |  | 1.000 | **< 0.001** | **< 0.001** | 0.724 | 1.000 | 1.000 |  | 1.000 | **< 0.001** | 0.064 | 0.674 | 1.000 | 1.000 |
| OTU_1231 | 0.057 | **< 0.001** | **< 0.001** | **< 0.001** | **< 0.001** | 1.000 |  | 0.057 | **< 0.001** | **< 0.001** | **< 0.001** | **< 0.001** | 1.000 |  | 1.000 | **< 0.001** | **< 0.001** | **< 0.001** | **< 0.001** | 1.000 |
| OTU_1232 | 1.000 | 1.000 | 1.000 | 1.000 | 1.000 | 1.000 |  | 1.000 | 0.117 | 0.510 | 1.000 | 1.000 | 1.000 |  | 1.000 | 1.000 | 1.000 | 1.000 | 1.000 | 1.000 |
| OTU_1234 | 1.000 | 1.000 | 1.000 | 0.100 | 1.000 | 1.000 |  | 1.000 | 0.243 | 1.000 | **0.007** | 0.114 | 1.000 |  | 1.000 | 1.000 | 1.000 | 1.000 | 0.069 | 1.000 |
| OTU_1235 | 1.000 | 1.000 | 0.088 | 1.000 | 0.098 | 0.333 |  | 1.000 | 0.380 | **< 0.001** | 1.000 | **0.001** | 0.081 |  | 0.383 | 1.000 | 0.392 | **0.008** | **< 0.001** | 1.000 |
| OTU_1236 | 1.000 | 0.768 | 0.358 | 1.000 | 0.881 | 1.000 |  | 1.000 | **0.020** | 1.000 | 1.000 | 1.000 | 1.000 |  | 1.000 | 1.000 | 1.000 | 1.000 | 1.000 | 1.000 |
| OTU_1237 | 1.000 | 1.000 | **0.001** | 1.000 | 0.074 | **0.032** |  | 1.000 | 1.000 | **0.002** | 1.000 | **0.003** | 1.000 |  | 1.000 | 1.000 | 0.075 | **0.048** | **< 0.001** | 1.000 |
| OTU_1238 | 0.513 | 1.000 | 1.000 | 1.000 | 0.319 | 1.000 |  | 0.513 | 1.000 | 0.173 | 0.715 | 1.000 | 0.348 |  | 1.000 | 1.000 | 1.000 | 1.000 | 1.000 | 1.000 |
| OTU_1239 | 1.000 | 0.058 | 1.000 | 1.000 | 1.000 | 0.748 |  | 1.000 | 0.116 | 1.000 | 1.000 | 1.000 | 1.000 |  | 1.000 | 1.000 | 1.000 | 1.000 | 1.000 | 1.000 |
| OTU_124 | 1.000 | **0.002** | **< 0.001** | **< 0.001** | **< 0.001** | **0.016** |  | 1.000 | **< 0.001** | **< 0.001** | **< 0.001** | **< 0.001** | **0.013** |  | **0.010** | **< 0.001** | **< 0.001** | **< 0.001** | **< 0.001** | 0.961 |
| OTU_1241 | 1.000 | 1.000 | 1.000 | 1.000 | 1.000 | 1.000 |  | 1.000 | 0.163 | 0.896 | 1.000 | 1.000 | 1.000 |  | 1.000 | 1.000 | 1.000 | 1.000 | 1.000 | 1.000 |
| OTU_1242 | 1.000 | 1.000 | 1.000 | 1.000 | 1.000 | 1.000 |  | 1.000 | 1.000 | 1.000 | 0.096 | 1.000 | 0.770 |  | 1.000 | 1.000 | 1.000 | 1.000 | 1.000 | 1.000 |
| OTU_1243 | 1.000 | 1.000 | 1.000 | 1.000 | 1.000 | 1.000 |  | 1.000 | 1.000 | 1.000 | 0.105 | 1.000 | 1.000 |  | 1.000 | 1.000 | 1.000 | 1.000 | 1.000 | 1.000 |
| OTU_1244 | 0.412 | 0.183 | 1.000 | 1.000 | 1.000 | 1.000 |  | 0.412 | 1.000 | 0.589 | 1.000 | 1.000 | 1.000 |  | 1.000 | 1.000 | 1.000 | 1.000 | 1.000 | 1.000 |
| OTU_1249 | 1.000 | 1.000 | **0.016** | 1.000 | **< 0.001** | **0.046** |  | 1.000 | 1.000 | **0.024** | 1.000 | 1.000 | 0.066 |  | 1.000 | 1.000 | **< 0.001** | 1.000 | **0.002** | **< 0.001** |
| OTU_125 | **0.039** | **< 0.001** | **0.002** | 0.550 | 1.000 | 1.000 |  | **0.039** | **< 0.001** | **< 0.001** | **< 0.001** | **< 0.001** | 1.000 |  | 1.000 | **< 0.001** | **0.003** | **< 0.001** | 0.258 | 0.114 |
| OTU_1250 | 1.000 | 1.000 | 1.000 | 1.000 | 1.000 | 1.000 |  | 1.000 | 1.000 | 1.000 | 1.000 | 1.000 | 1.000 |  | 0.157 | 1.000 | 1.000 | 1.000 | 1.000 | 1.000 |
| OTU_1252 | 0.620 | 0.208 | 0.086 | 1.000 | 1.000 | 1.000 |  | 0.620 | 0.373 | 1.000 | 0.194 | 1.000 | 1.000 |  | 1.000 | 1.000 | 1.000 | 1.000 | 1.000 | 1.000 |
| OTU_1254 | 1.000 | 1.000 | 1.000 | 1.000 | 1.000 | 1.000 |  | 1.000 | 0.146 | 0.060 | 1.000 | 1.000 | 1.000 |  | 1.000 | **0.006** | **0.003** | 0.505 | 0.270 | 1.000 |
| OTU_1255 | 1.000 | 1.000 | 1.000 | 0.440 | 1.000 | 1.000 |  | 1.000 | 1.000 | 1.000 | 1.000 | 1.000 | 1.000 |  | 1.000 | 1.000 | 0.305 | 1.000 | 1.000 | 1.000 |
| OTU_1256 | **< 0.001** | **< 0.001** | 0.226 | 1.000 | **0.016** | 0.370 |  | **< 0.001** | **0.002** | **0.003** | 1.000 | 1.000 | 1.000 |  | 0.981 | **< 0.001** | **0.046** | 0.847 | 1.000 | 1.000 |
| OTU_1257 | 0.477 | 0.655 | 1.000 | 1.000 | 1.000 | 1.000 |  | 0.477 | 0.394 | 1.000 | 1.000 | 0.813 | 1.000 |  | 1.000 | 1.000 | 1.000 | 1.000 | 1.000 | 1.000 |
| OTU_1259 | 1.000 | 0.119 | 1.000 | 1.000 | 1.000 | 0.532 |  | 1.000 | **< 0.001** | 0.075 | 1.000 | 1.000 | 0.466 |  | 1.000 | **< 0.001** | 1.000 | **0.021** | 1.000 | 0.068 |
| OTU_126 | 1.000 | 0.139 | **< 0.001** | 1.000 | **0.007** | 1.000 |  | 1.000 | **< 0.001** | **< 0.001** | **0.002** | **< 0.001** | 1.000 |  | 1.000 | **0.042** | 0.381 | **0.042** | 0.303 | 1.000 |
| OTU_1260 | 1.000 | 0.111 | 0.324 | 0.060 | 0.152 | 1.000 |  | 1.000 | **0.009** | **< 0.001** | 0.136 | **0.011** | 1.000 |  | 1.000 | 0.259 | 0.217 | 1.000 | 1.000 | 1.000 |
| OTU_1261 | 1.000 | 1.000 | 1.000 | 1.000 | 1.000 | 1.000 |  | 1.000 | 1.000 | 1.000 | 1.000 | 1.000 | 1.000 |  | 1.000 | 1.000 | 1.000 | 1.000 | 1.000 | 1.000 |
| OTU_1263 | 1.000 | 1.000 | 1.000 | 1.000 | 1.000 | 1.000 |  | 1.000 | 1.000 | 1.000 | 1.000 | 1.000 | 1.000 |  | 1.000 | 1.000 | 1.000 | 1.000 | 1.000 | 1.000 |
| OTU_1266 | 1.000 | 0.651 | 1.000 | 0.384 | 1.000 | 1.000 |  | 1.000 | **0.005** | **0.048** | 0.227 | 1.000 | 1.000 |  | 1.000 | 1.000 | 1.000 | 1.000 | 1.000 | 1.000 |
| OTU_127 | 1.000 | **< 0.001** | **< 0.001** | **0.001** | **< 0.001** | 1.000 |  | 1.000 | **< 0.001** | **< 0.001** | **0.004** | **0.004** | 1.000 |  | 1.000 | **< 0.001** | **< 0.001** | **< 0.001** | **< 0.001** | 1.000 |
| OTU_1270 | 1.000 | 1.000 | 1.000 | 1.000 | 1.000 | 1.000 |  | 1.000 | 1.000 | 1.000 | 1.000 | 1.000 | 1.000 |  | 1.000 | 1.000 | 1.000 | 1.000 | 1.000 | 1.000 |
| OTU_1271 | 1.000 | **0.027** | **0.005** | **0.016** | **0.001** | 1.000 |  | 1.000 | **< 0.001** | **< 0.001** | **< 0.001** | **< 0.001** | 1.000 |  | 1.000 | 1.000 | 1.000 | 0.520 | 1.000 | 1.000 |
| OTU_1275 | 1.000 | 1.000 | 1.000 | 1.000 | 1.000 | 1.000 |  | 1.000 | 1.000 | 1.000 | **0.002** | 0.159 | 1.000 |  | 1.000 | 1.000 | 1.000 | 1.000 | 1.000 | 1.000 |
| OTU_1276 | 1.000 | 1.000 | 0.117 | 1.000 | 1.000 | 1.000 |  | 1.000 | 1.000 | 1.000 | 1.000 | 1.000 | 1.000 |  | 1.000 | 1.000 | 1.000 | 1.000 | 1.000 | 1.000 |
| OTU_1277 | 0.493 | 1.000 | 0.830 | 0.050 | **< 0.001** | 1.000 |  | 0.493 | 1.000 | 0.060 | 1.000 | 0.072 | 1.000 |  | 1.000 | 0.491 | **0.008** | 1.000 | 0.061 | 1.000 |
| OTU_1278 | 1.000 | 1.000 | 1.000 | 0.315 | **0.025** | 1.000 |  | 1.000 | 0.090 | 0.335 | 1.000 | 1.000 | 1.000 |  | 1.000 | 1.000 | 1.000 | 1.000 | 1.000 | 1.000 |
| OTU_1279 | 1.000 | 1.000 | 1.000 | 1.000 | 1.000 | 1.000 |  | 1.000 | **0.007** | 1.000 | 1.000 | 0.056 | 1.000 |  | **0.008** | 0.263 | 1.000 | 1.000 | **0.015** | 1.000 |
| OTU_128 | 1.000 | 1.000 | **< 0.001** | 1.000 | **< 0.001** | **< 0.001** |  | 1.000 | 1.000 | **< 0.001** | 1.000 | **< 0.001** | **< 0.001** |  | 1.000 | 1.000 | **< 0.001** | 1.000 | **< 0.001** | **< 0.001** |
| OTU_1282 | 1.000 | 1.000 | 1.000 | 1.000 | 1.000 | 1.000 |  | 1.000 | 1.000 | 1.000 | 1.000 | 1.000 | 1.000 |  | 1.000 | **0.025** | **0.029** | 1.000 | 1.000 | 1.000 |
| OTU_1283 | 1.000 | 1.000 | 1.000 | 1.000 | 1.000 | 1.000 |  | 1.000 | 1.000 | 1.000 | 1.000 | 1.000 | 1.000 |  | 1.000 | 0.211 | **0.008** | 0.626 | **0.022** | 1.000 |
| OTU_1284 | 1.000 | 0.107 | 1.000 | 0.581 | 1.000 | 1.000 |  | 1.000 | 1.000 | **0.046** | 1.000 | 0.056 | 1.000 |  | 1.000 | 1.000 | 1.000 | 1.000 | 1.000 | 1.000 |
| OTU_1286 | 1.000 | 1.000 | 1.000 | 1.000 | 1.000 | 1.000 |  | 1.000 | 1.000 | 1.000 | 1.000 | 0.723 | 1.000 |  | 1.000 | 1.000 | 0.137 | 1.000 | 0.266 | 1.000 |
| OTU_1287 | 1.000 | 1.000 | 1.000 | 1.000 | 1.000 | 1.000 |  | 1.000 | 1.000 | 1.000 | 1.000 | 0.481 | 1.000 |  | 1.000 | 1.000 | 0.487 | 1.000 | 0.103 | 1.000 |
| OTU_1288 | 1.000 | 1.000 | 1.000 | 1.000 | 1.000 | 1.000 |  | 1.000 | 1.000 | 1.000 | 0.119 | 0.159 | 1.000 |  | 1.000 | 1.000 | 1.000 | 1.000 | 1.000 | 1.000 |
| OTU_1289 | 0.123 | 0.734 | 1.000 | 1.000 | 0.314 | 1.000 |  | 0.123 | 0.497 | 1.000 | 1.000 | 0.271 | 0.301 |  | 1.000 | 1.000 | 1.000 | 1.000 | 1.000 | 0.802 |
| OTU_129 | 1.000 | 1.000 | **< 0.001** | 1.000 | **< 0.001** | **< 0.001** |  | 1.000 | 1.000 | **< 0.001** | 1.000 | **< 0.001** | **< 0.001** |  | 0.234 | 1.000 | **< 0.001** | 1.000 | **< 0.001** | **< 0.001** |
| OTU_1290 | 1.000 | 1.000 | 1.000 | 1.000 | 1.000 | 1.000 |  | 1.000 | 0.871 | 1.000 | 1.000 | 0.525 | 1.000 |  | 1.000 | 1.000 | 1.000 | 1.000 | 1.000 | 0.396 |
| OTU_1291 | 1.000 | 1.000 | 0.647 | 1.000 | 0.549 | 0.748 |  | 1.000 | 1.000 | 1.000 | 1.000 | 0.969 | 1.000 |  | 1.000 | 1.000 | 1.000 | 1.000 | 1.000 | 1.000 |
| OTU_1292 | 1.000 | 1.000 | 1.000 | 1.000 | 1.000 | 1.000 |  | 1.000 | 0.800 | 1.000 | 1.000 | 0.652 | 1.000 |  | 1.000 | 1.000 | 1.000 | 1.000 | 1.000 | 1.000 |
| OTU_1294 | **0.014** | **0.008** | 0.051 | 1.000 | 1.000 | 1.000 |  | **0.014** | **0.002** | **< 0.001** | 1.000 | 1.000 | 1.000 |  | 0.996 | 0.588 | 0.405 | 1.000 | 1.000 | 1.000 |
| OTU_1295 | 1.000 | 1.000 | 1.000 | 1.000 | 1.000 | 1.000 |  | 1.000 | 0.583 | **0.048** | 1.000 | 1.000 | 1.000 |  | 1.000 | 1.000 | 1.000 | 1.000 | 1.000 | 1.000 |
| OTU_1296 | 1.000 | 1.000 | 1.000 | 1.000 | 1.000 | 1.000 |  | 1.000 | **0.041** | 1.000 | **0.004** | 1.000 | **0.008** |  | 1.000 | 0.300 | 1.000 | 0.220 | 1.000 | 1.000 |
| OTU_1297 | 1.000 | 1.000 | 1.000 | 1.000 | 1.000 | 1.000 |  | 1.000 | 1.000 | 1.000 | 1.000 | 1.000 | 1.000 |  | **0.003** | 0.163 | 1.000 | 1.000 | **0.010** | 1.000 |
| OTU_1298 | 1.000 | **< 0.001** | **0.017** | 0.125 | 1.000 | 1.000 |  | 1.000 | 0.430 | 1.000 | 1.000 | 1.000 | 1.000 |  | 0.200 | **0.002** | 0.087 | 1.000 | 1.000 | 1.000 |
| OTU_1299 | **< 0.001** | **< 0.001** | 1.000 | 1.000 | 0.262 | 0.107 |  | **< 0.001** | **< 0.001** | **0.012** | **0.037** | 1.000 | 0.284 |  | 0.636 | **< 0.001** | 1.000 | 0.838 | 1.000 | 0.247 |
| OTU_13 | 1.000 | **< 0.001** | **< 0.001** | 0.803 | 0.586 | 1.000 |  | 1.000 | **0.003** | **< 0.001** | 1.000 | 0.334 | 1.000 |  | 0.360 | **< 0.001** | **< 0.001** | 1.000 | 1.000 | 1.000 |
| OTU_130 | 1.000 | **< 0.001** | **< 0.001** | **< 0.001** | **< 0.001** | 1.000 |  | 1.000 | **< 0.001** | **< 0.001** | **< 0.001** | **< 0.001** | 1.000 |  | **< 0.001** | **< 0.001** | **< 0.001** | 0.471 | 1.000 | 0.077 |
| OTU_1302 | 1.000 | 0.282 | 1.000 | 0.620 | 1.000 | 1.000 |  | 1.000 | 1.000 | 1.000 | 1.000 | 0.083 | 1.000 |  | 1.000 | 1.000 | 1.000 | 1.000 | 1.000 | 1.000 |
| OTU_1304 | **< 0.001** | **< 0.001** | **0.002** | 1.000 | 1.000 | 1.000 |  | **< 0.001** | **0.033** | **0.028** | 1.000 | 1.000 | 1.000 |  | 1.000 | 1.000 | 0.586 | 1.000 | 1.000 | 1.000 |
| OTU_1307 | 1.000 | **0.036** | 0.391 | 1.000 | 1.000 | 1.000 |  | 1.000 | 0.220 | 1.000 | 1.000 | 1.000 | 1.000 |  | 1.000 | **< 0.001** | 1.000 | 0.109 | 1.000 | 0.137 |
| OTU_1308 | 1.000 | **0.035** | 0.980 | 0.124 | 1.000 | 1.000 |  | 1.000 | 0.516 | 1.000 | 0.111 | 1.000 | 1.000 |  | 1.000 | 1.000 | 1.000 | 1.000 | 1.000 | 1.000 |
| OTU_131 | 1.000 | **< 0.001** | **< 0.001** | **< 0.001** | **< 0.001** | 1.000 |  | 1.000 | **0.007** | **< 0.001** | **< 0.001** | **< 0.001** | 1.000 |  | 1.000 | **0.045** | **< 0.001** | 0.134 | **0.002** | 1.000 |
| OTU_1310 | 1.000 | **< 0.001** | **< 0.001** | 0.066 | **0.006** | 1.000 |  | 1.000 | **< 0.001** | **< 0.001** | 0.170 | **0.004** | 1.000 |  | 1.000 | **0.005** | **0.003** | 0.345 | 0.172 | 1.000 |
| OTU_1313 | **< 0.001** | **< 0.001** | **< 0.001** | 1.000 | 1.000 | 1.000 |  | **< 0.001** | **< 0.001** | **< 0.001** | 0.232 | 0.137 | 1.000 |  | 1.000 | 1.000 | 1.000 | 1.000 | 1.000 | 1.000 |
| OTU_1314 | **0.017** | 0.071 | **0.008** | 1.000 | 1.000 | 1.000 |  | **0.017** | **0.010** | 0.203 | 0.340 | 1.000 | 1.000 |  | **0.027** | **0.001** | **< 0.001** | 1.000 | 1.000 | 1.000 |
| OTU_1315 | 1.000 | 0.272 | 1.000 | 1.000 | 1.000 | 1.000 |  | 1.000 | 1.000 | 1.000 | 0.616 | 1.000 | 1.000 |  | 1.000 | 1.000 | 1.000 | 1.000 | 0.864 | 1.000 |
| OTU_1316 | 0.290 | 1.000 | **0.024** | 1.000 | 1.000 | 1.000 |  | 0.290 | 0.198 | 0.071 | 1.000 | 1.000 | 1.000 |  | 1.000 | 0.157 | **0.001** | 0.305 | **0.002** | 1.000 |
| OTU_1318 | 1.000 | 0.179 | 1.000 | 1.000 | 1.000 | 1.000 |  | 1.000 | **0.002** | 1.000 | **0.035** | 1.000 | 0.080 |  | 0.608 | 0.218 | 0.708 | 1.000 | 1.000 | 1.000 |
| OTU_1319 | **0.003** | 1.000 | 1.000 | 0.252 | **0.019** | 1.000 |  | **0.003** | 1.000 | 0.512 | 1.000 | 1.000 | 1.000 |  | 0.846 | 1.000 | 1.000 | 1.000 | 1.000 | 1.000 |
| OTU_132 | 1.000 | **0.040** | **0.004** | 0.147 | **0.013** | 1.000 |  | 1.000 | **0.014** | **0.001** | 0.096 | **0.010** | 1.000 |  | 0.847 | **< 0.001** | **< 0.001** | 0.109 | 0.209 | 1.000 |
| OTU_1320 | 1.000 | 1.000 | 1.000 | 1.000 | 1.000 | 1.000 |  | 1.000 | 1.000 | 1.000 | 1.000 | 1.000 | 1.000 |  | 1.000 | 1.000 | 1.000 | 1.000 | 1.000 | 1.000 |
| OTU_1322 | 1.000 | 1.000 | 1.000 | 1.000 | 0.768 | 1.000 |  | 1.000 | 0.090 | 0.260 | 1.000 | 1.000 | 1.000 |  | 1.000 | 1.000 | 0.600 | 1.000 | 0.931 | 1.000 |
| OTU_1327 | 1.000 | 1.000 | 1.000 | 1.000 | 1.000 | 1.000 |  | 1.000 | 1.000 | 1.000 | 1.000 | 1.000 | 1.000 |  | 1.000 | **0.031** | **0.019** | 1.000 | 0.993 | 1.000 |
| OTU_133 | 1.000 | 0.951 | **0.002** | 1.000 | **0.002** | 1.000 |  | 1.000 | 1.000 | 1.000 | 1.000 | 1.000 | 1.000 |  | 0.833 | 1.000 | 1.000 | 1.000 | **0.012** | 0.194 |
| OTU_1330 | 1.000 | 0.056 | 1.000 | **0.050** | 1.000 | 0.065 |  | 1.000 | 0.914 | 1.000 | 1.000 | 1.000 | 1.000 |  | 0.443 | **< 0.001** | 1.000 | 0.905 | 1.000 | 0.066 |
| OTU_1333 | 1.000 | **0.007** | 0.163 | 0.237 | 1.000 | 1.000 |  | 1.000 | **0.005** | 0.056 | **< 0.001** | **0.006** | 1.000 |  | 0.169 | **< 0.001** | **< 0.001** | 0.189 | 1.000 | 1.000 |
| OTU_1336 | 1.000 | 1.000 | 1.000 | 0.225 | 0.786 | 1.000 |  | 1.000 | **0.021** | **0.009** | 1.000 | 1.000 | 1.000 |  | 1.000 | 1.000 | 1.000 | 1.000 | 1.000 | 1.000 |
| OTU_1339 | 1.000 | 1.000 | 1.000 | 1.000 | 1.000 | 1.000 |  | 1.000 | 0.116 | 1.000 | 0.192 | 1.000 | 1.000 |  | 1.000 | 1.000 | 1.000 | 1.000 | 1.000 | 1.000 |
| OTU_134 | 1.000 | **< 0.001** | **< 0.001** | **0.017** | **0.028** | 1.000 |  | 1.000 | **< 0.001** | **< 0.001** | 0.264 | 1.000 | 0.808 |  | **< 0.001** | **< 0.001** | **< 0.001** | 1.000 | 1.000 | 1.000 |
| OTU_1345 | 1.000 | 1.000 | 1.000 | 1.000 | 1.000 | 1.000 |  | 1.000 | 1.000 | 1.000 | 1.000 | 1.000 | 1.000 |  | 0.170 | 1.000 | 1.000 | 0.471 | 0.302 | 1.000 |
| OTU_1348 | 1.000 | 0.398 | 1.000 | 1.000 | 1.000 | 1.000 |  | 1.000 | 1.000 | 1.000 | 0.097 | 0.054 | 1.000 |  | 1.000 | 1.000 | 1.000 | 1.000 | 1.000 | 1.000 |
| OTU_135 | 0.107 | 1.000 | **< 0.001** | 1.000 | **< 0.001** | **< 0.001** |  | 0.107 | 1.000 | **< 0.001** | 1.000 | **< 0.001** | **< 0.001** |  | 1.000 | 1.000 | **< 0.001** | 1.000 | **< 0.001** | **< 0.001** |
| OTU_1355 | 1.000 | 1.000 | 1.000 | 1.000 | 1.000 | 1.000 |  | 1.000 | **0.012** | 1.000 | 1.000 | 0.178 | **0.008** |  | 1.000 | 0.073 | 1.000 | 0.564 | 1.000 | 0.058 |
| OTU_1357 | 1.000 | 1.000 | 1.000 | 1.000 | 1.000 | 1.000 |  | 1.000 | **0.011** | 0.134 | **0.018** | 0.158 | 1.000 |  | 1.000 | 1.000 | 1.000 | 1.000 | 0.743 | 1.000 |
| OTU_1359 | 1.000 | 1.000 | 1.000 | 1.000 | 0.349 | 0.665 |  | 1.000 | 1.000 | 0.447 | 1.000 | 1.000 | 1.000 |  | 1.000 | 1.000 | 1.000 | 1.000 | 1.000 | 1.000 |
| OTU_136 | 1.000 | 1.000 | 1.000 | 1.000 | 1.000 | 1.000 |  | 1.000 | 1.000 | 1.000 | 1.000 | 1.000 | 1.000 |  | 1.000 | 1.000 | 1.000 | 1.000 | 1.000 | 1.000 |
| OTU_1361 | 1.000 | 1.000 | 1.000 | 1.000 | 1.000 | 1.000 |  | 1.000 | 1.000 | 1.000 | 1.000 | 1.000 | 1.000 |  | 1.000 | 1.000 | 1.000 | 0.229 | 1.000 | 1.000 |
| OTU_1362 | 0.095 | 0.063 | 0.053 | 1.000 | 1.000 | 1.000 |  | 0.095 | **< 0.001** | **< 0.001** | 1.000 | 1.000 | 1.000 |  | **0.045** | 0.179 | **0.011** | 1.000 | 1.000 | 1.000 |
| OTU_1363 | 1.000 | 1.000 | 1.000 | 1.000 | 1.000 | 1.000 |  | 1.000 | 1.000 | 1.000 | 1.000 | 1.000 | 1.000 |  | 1.000 | 1.000 | 1.000 | 1.000 | 1.000 | 1.000 |
| OTU_1366 | 1.000 | 1.000 | 1.000 | 1.000 | 1.000 | 1.000 |  | 1.000 | 0.778 | 1.000 | 1.000 | 1.000 | 1.000 |  | 1.000 | 1.000 | 1.000 | 1.000 | 1.000 | 1.000 |
| OTU_1367 | 1.000 | 0.051 | **0.036** | 1.000 | 1.000 | 1.000 |  | 1.000 | **0.015** | 1.000 | **0.025** | 1.000 | 1.000 |  | 1.000 | 1.000 | 1.000 | 0.227 | 0.621 | 1.000 |
| OTU_1368 | 1.000 | 0.712 | 1.000 | 1.000 | 1.000 | 1.000 |  | 1.000 | **0.001** | 1.000 | 1.000 | 1.000 | 0.321 |  | 1.000 | 0.852 | 1.000 | 0.429 | 1.000 | 1.000 |
| OTU_137 | 1.000 | 1.000 | **0.006** | 1.000 | **0.015** | **0.023** |  | 1.000 | 0.230 | **< 0.001** | 1.000 | **0.009** | 0.078 |  | 1.000 | 1.000 | **0.001** | 1.000 | **0.005** | 0.241 |
| OTU_1371 | 0.322 | 1.000 | 1.000 | 1.000 | 0.312 | 1.000 |  | 0.322 | 1.000 | 0.101 | 1.000 | 1.000 | 1.000 |  | 1.000 | 1.000 | 1.000 | 1.000 | 1.000 | 1.000 |
| OTU_1375 | 1.000 | 1.000 | 1.000 | 1.000 | 1.000 | 1.000 |  | 1.000 | 1.000 | 1.000 | 1.000 | 1.000 | 1.000 |  | 1.000 | 1.000 | 1.000 | 1.000 | 1.000 | 1.000 |
| OTU_1377 | 1.000 | 1.000 | 1.000 | 1.000 | 1.000 | 1.000 |  | 1.000 | 1.000 | **0.026** | 1.000 | 1.000 | 1.000 |  | 1.000 | 1.000 | 0.447 | 0.558 | **0.008** | 1.000 |
| OTU_1378 | 1.000 | 1.000 | 1.000 | 1.000 | 1.000 | 0.934 |  | 1.000 | 1.000 | 1.000 | 1.000 | 1.000 | 1.000 |  | 1.000 | 1.000 | 1.000 | 1.000 | 1.000 | 1.000 |
| OTU_1379 | 1.000 | 1.000 | 1.000 | 1.000 | 1.000 | 1.000 |  | 1.000 | 1.000 | 1.000 | 1.000 | 0.572 | 1.000 |  | 1.000 | 1.000 | 1.000 | 1.000 | 1.000 | 1.000 |
| OTU_138 | **< 0.001** | **< 0.001** | **< 0.001** | 0.554 | 0.281 | 1.000 |  | **< 0.001** | **< 0.001** | **< 0.001** | 1.000 | 0.905 | 1.000 |  | **0.018** | **< 0.001** | **< 0.001** | **0.025** | **0.041** | 1.000 |
| OTU_1382 | 1.000 | 1.000 | 1.000 | 1.000 | 1.000 | 1.000 |  | 1.000 | 1.000 | 1.000 | 1.000 | 1.000 | 1.000 |  | 1.000 | 1.000 | 1.000 | 1.000 | 1.000 | 1.000 |
| OTU_1383 | 0.650 | 1.000 | 1.000 | 1.000 | 1.000 | 1.000 |  | 0.650 | 1.000 | 1.000 | 1.000 | 1.000 | 1.000 |  | 1.000 | 1.000 | 1.000 | 1.000 | 0.839 | 1.000 |
| OTU_1384 | 1.000 | 1.000 | 1.000 | 1.000 | 1.000 | 1.000 |  | 1.000 | 0.254 | 0.696 | 1.000 | 1.000 | 1.000 |  | 1.000 | 1.000 | 1.000 | 1.000 | 1.000 | 1.000 |
| OTU_1386 | 1.000 | **0.001** | 0.251 | 0.059 | 1.000 | 1.000 |  | 1.000 | **< 0.001** | **0.002** | **0.004** | 1.000 | 1.000 |  | 1.000 | **0.010** | 0.189 | 0.106 | 0.935 | 1.000 |
| OTU_1387 | 1.000 | 1.000 | 0.583 | 1.000 | 1.000 | 1.000 |  | 1.000 | 1.000 | 1.000 | 0.328 | 0.146 | 1.000 |  | 1.000 | 1.000 | 1.000 | 1.000 | 1.000 | 1.000 |
| OTU_139 | 1.000 | **< 0.001** | 0.067 | **0.002** | 1.000 | 0.816 |  | 1.000 | **< 0.001** | **< 0.001** | **0.002** | 0.830 | 1.000 |  | 1.000 | **< 0.001** | 0.440 | 0.162 | 1.000 | 1.000 |
| OTU_1391 | 1.000 | 1.000 | 1.000 | 1.000 | 1.000 | 1.000 |  | 1.000 | 1.000 | 0.294 | 1.000 | 1.000 | 0.959 |  | 1.000 | 1.000 | 1.000 | 1.000 | 1.000 | 1.000 |
| OTU_1393 | 1.000 | 1.000 | 1.000 | 1.000 | 1.000 | 1.000 |  | 1.000 | 1.000 | 1.000 | 1.000 | 1.000 | 1.000 |  | 1.000 | 1.000 | 1.000 | 1.000 | 1.000 | 1.000 |
| OTU_1397 | 1.000 | 1.000 | 1.000 | 1.000 | 1.000 | 1.000 |  | 1.000 | 0.365 | 0.068 | 1.000 | 1.000 | 1.000 |  | 1.000 | 1.000 | 1.000 | 1.000 | 1.000 | 1.000 |
| OTU_1399 | 1.000 | 1.000 | 0.479 | 1.000 | 0.168 | 1.000 |  | 1.000 | 1.000 | 1.000 | 1.000 | 0.079 | 0.132 |  | 1.000 | 1.000 | 1.000 | 1.000 | 1.000 | 1.000 |
| OTU_14 | 1.000 | 1.000 | **0.003** | 1.000 | **< 0.001** | 0.306 |  | 1.000 | 0.523 | **< 0.001** | 1.000 | 0.084 | 0.562 |  | 1.000 | 0.898 | 0.279 | 1.000 | 0.431 | 1.000 |
| OTU_140 | 1.000 | **< 0.001** | **< 0.001** | **0.002** | **< 0.001** | 1.000 |  | 1.000 | 0.577 | **0.011** | 0.282 | **0.003** | 1.000 |  | 1.000 | **< 0.001** | **< 0.001** | **0.041** | **0.032** | 1.000 |
| OTU_1402 | **< 0.001** | **< 0.001** | **< 0.001** | 1.000 | 1.000 | 1.000 |  | **< 0.001** | **< 0.001** | **< 0.001** | 1.000 | 1.000 | 1.000 |  | **< 0.001** | **< 0.001** | **< 0.001** | 1.000 | 1.000 | 1.000 |
| OTU_1403 | **0.035** | **0.006** | **0.005** | 1.000 | 1.000 | 1.000 |  | **0.035** | **< 0.001** | **< 0.001** | 0.117 | **0.031** | 1.000 |  | 1.000 | 0.488 | **0.038** | 1.000 | 1.000 | 1.000 |
| OTU_1405 | 1.000 | 1.000 | 0.107 | 1.000 | 1.000 | 0.250 |  | 1.000 | 1.000 | 0.980 | **0.033** | 1.000 | 1.000 |  | 1.000 | 1.000 | **0.026** | 1.000 | 0.259 | 0.664 |
| OTU_141 | 0.063 | 1.000 | 1.000 | 0.085 | **0.002** | 1.000 |  | 0.063 | 1.000 | 1.000 | **0.020** | **< 0.001** | 1.000 |  | **< 0.001** | **0.015** | 1.000 | 0.278 | **< 0.001** | 0.242 |
| OTU_1410 | 1.000 | 1.000 | 1.000 | 1.000 | 1.000 | 1.000 |  | 1.000 | **< 0.001** | **0.010** | 1.000 | 1.000 | 1.000 |  | 1.000 | 1.000 | 1.000 | 1.000 | 1.000 | 1.000 |
| OTU_1413 | 1.000 | 0.129 | 0.185 | 1.000 | 1.000 | 1.000 |  | 1.000 | **0.018** | 0.077 | 1.000 | 1.000 | 1.000 |  | 1.000 | 1.000 | 1.000 | 1.000 | 1.000 | 1.000 |
| OTU_1417 | 1.000 | 1.000 | 1.000 | 1.000 | 1.000 | 1.000 |  | 1.000 | 0.538 | 1.000 | 1.000 | 1.000 | 0.175 |  | 1.000 | 1.000 | 1.000 | 1.000 | 1.000 | 1.000 |
| OTU_1418 | 1.000 | 1.000 | 1.000 | 1.000 | 1.000 | 1.000 |  | 1.000 | 1.000 | 0.366 | 1.000 | 1.000 | 1.000 |  | 1.000 | 1.000 | 1.000 | 1.000 | 1.000 | 1.000 |
| OTU_1419 | 1.000 | 0.115 | 0.493 | 1.000 | 1.000 | 1.000 |  | 1.000 | 1.000 | 1.000 | 1.000 | 1.000 | 1.000 |  | 0.926 | 1.000 | 0.378 | 1.000 | 1.000 | 1.000 |
| OTU_142 | **0.009** | 0.871 | **< 0.001** | 1.000 | **< 0.001** | **< 0.001** |  | **0.009** | 1.000 | **< 0.001** | 0.541 | **< 0.001** | **< 0.001** |  | **0.030** | 1.000 | **< 0.001** | 1.000 | **< 0.001** | **< 0.001** |
| OTU_1421 | 1.000 | 0.332 | 0.747 | 1.000 | 1.000 | 1.000 |  | 1.000 | 0.357 | 1.000 | 1.000 | 1.000 | 1.000 |  | 1.000 | 0.318 | 0.178 | 1.000 | 1.000 | 1.000 |
| OTU_1422 | **< 0.001** | **< 0.001** | **< 0.001** | 1.000 | 1.000 | 1.000 |  | **< 0.001** | 0.880 | 1.000 | 1.000 | 1.000 | 0.856 |  | **< 0.001** | **< 0.001** | **< 0.001** | 1.000 | 1.000 | 1.000 |
| OTU_1424 | 1.000 | 1.000 | 1.000 | 1.000 | 1.000 | 1.000 |  | 1.000 | 1.000 | 0.594 | 1.000 | 1.000 | 1.000 |  | 1.000 | 1.000 | 1.000 | 1.000 | 1.000 | 1.000 |
| OTU_1429 | 1.000 | 0.679 | 1.000 | **0.007** | 0.470 | 1.000 |  | 1.000 | **0.017** | 0.472 | 1.000 | 1.000 | 1.000 |  | 1.000 | 0.095 | 1.000 | 1.000 | 1.000 | 1.000 |
| OTU_143 | 1.000 | 1.000 | 1.000 | 1.000 | 0.169 | 1.000 |  | 1.000 | 1.000 | 1.000 | **0.014** | **< 0.001** | 1.000 |  | 1.000 | 1.000 | 1.000 | 1.000 | 1.000 | 1.000 |
| OTU_1435 | 0.233 | **< 0.001** | **< 0.001** | 1.000 | 1.000 | 1.000 |  | 0.233 | **< 0.001** | **0.003** | 0.270 | 1.000 | 0.516 |  | 1.000 | 0.218 | 0.105 | 1.000 | 1.000 | 1.000 |
| OTU_1438 | 1.000 | 1.000 | 1.000 | 1.000 | 1.000 | 0.716 |  | 1.000 | 1.000 | 1.000 | 1.000 | 1.000 | 1.000 |  | 1.000 | 1.000 | 1.000 | 1.000 | 0.116 | 1.000 |
| OTU_144 | **< 0.001** | **< 0.001** | **< 0.001** | 1.000 | 1.000 | 1.000 |  | **< 0.001** | **< 0.001** | **< 0.001** | 0.451 | 1.000 | **0.043** |  | **< 0.001** | **< 0.001** | **< 0.001** | 0.697 | 1.000 | 1.000 |
| OTU_1440 | 1.000 | 1.000 | 1.000 | 1.000 | **0.035** | 1.000 |  | 1.000 | 1.000 | 1.000 | 1.000 | 0.850 | 1.000 |  | 1.000 | 1.000 | 0.229 | 1.000 | 0.221 | 0.493 |
| OTU_1443 | 1.000 | 1.000 | 1.000 | 1.000 | 1.000 | 1.000 |  | 1.000 | 1.000 | 1.000 | 1.000 | 1.000 | 1.000 |  | 1.000 | 1.000 | 1.000 | 1.000 | 1.000 | 1.000 |
| OTU_1444 | 1.000 | **0.032** | 1.000 | 1.000 | 1.000 | 1.000 |  | 1.000 | 0.291 | 1.000 | 1.000 | 1.000 | 0.702 |  | 1.000 | 0.124 | 1.000 | 1.000 | 1.000 | 1.000 |
| OTU_1446 | 1.000 | 1.000 | 1.000 | 1.000 | 1.000 | 1.000 |  | 1.000 | 1.000 | 1.000 | 1.000 | 1.000 | 1.000 |  | 1.000 | 1.000 | **< 0.001** | 1.000 | **0.014** | **0.001** |
| OTU_1447 | 0.107 | 0.163 | 0.124 | 1.000 | 1.000 | 1.000 |  | 0.107 | 0.634 | 1.000 | 1.000 | 1.000 | 1.000 |  | 1.000 | 0.056 | **0.036** | 1.000 | 1.000 | 1.000 |
| OTU_1448 | **0.007** | **0.043** | 1.000 | 1.000 | **0.018** | 0.376 |  | **0.007** | 0.607 | 1.000 | 1.000 | 1.000 | 0.474 |  | 0.132 | 1.000 | 1.000 | 1.000 | **0.016** | 1.000 |
| OTU_1449 | 1.000 | 1.000 | 1.000 | 1.000 | 1.000 | 1.000 |  | 1.000 | **0.010** | 1.000 | 0.257 | 1.000 | 1.000 |  | 1.000 | 1.000 | 1.000 | 1.000 | 1.000 | 1.000 |
| OTU_145 | 1.000 | **< 0.001** | **< 0.001** | **< 0.001** | **< 0.001** | **0.010** |  | 1.000 | **0.014** | **< 0.001** | **0.002** | **< 0.001** | **0.006** |  | 1.000 | 0.276 | **< 0.001** | 0.297 | **< 0.001** | **< 0.001** |
| OTU_1450 | 1.000 | 1.000 | 1.000 | 1.000 | 1.000 | 1.000 |  | 1.000 | 1.000 | 1.000 | 1.000 | 1.000 | 1.000 |  | 1.000 | 1.000 | 1.000 | 1.000 | 1.000 | 1.000 |
| OTU_1451 | 1.000 | 1.000 | 1.000 | 1.000 | 1.000 | 1.000 |  | 1.000 | 1.000 | 0.820 | 1.000 | 0.208 | 1.000 |  | 1.000 | 1.000 | 0.228 | 1.000 | 0.906 | 1.000 |
| OTU_1453 | 1.000 | 1.000 | 0.172 | 1.000 | 0.113 | 0.056 |  | 1.000 | 0.608 | 1.000 | 1.000 | **0.004** | **0.019** |  | 1.000 | 1.000 | 1.000 | 1.000 | 1.000 | 0.213 |
| OTU_1455 | 1.000 | 1.000 | 1.000 | 1.000 | 1.000 | 1.000 |  | 1.000 | 1.000 | 1.000 | 1.000 | 1.000 | 1.000 |  | 1.000 | 0.947 | 1.000 | 1.000 | 1.000 | 1.000 |
| OTU_1456 | 1.000 | 1.000 | 1.000 | 1.000 | 1.000 | 1.000 |  | 1.000 | **0.004** | **< 0.001** | 1.000 | 1.000 | 1.000 |  | 0.903 | **< 0.001** | **0.047** | 1.000 | 1.000 | 1.000 |
| OTU_1457 | 1.000 | 0.173 | 0.259 | 1.000 | 1.000 | 1.000 |  | 1.000 | 1.000 | **0.004** | 1.000 | **0.006** | 1.000 |  | 1.000 | 1.000 | 0.554 | 1.000 | 0.621 | 1.000 |
| OTU_1458 | 1.000 | 1.000 | 1.000 | 1.000 | 1.000 | 1.000 |  | 1.000 | 0.787 | 0.468 | 1.000 | 1.000 | 1.000 |  | 0.592 | 1.000 | 1.000 | 1.000 | 1.000 | 1.000 |
| OTU_1459 | **0.018** | **0.012** | 0.628 | 1.000 | 1.000 | 1.000 |  | **0.018** | **< 0.001** | 0.166 | 1.000 | 0.404 | 0.637 |  | **0.004** | **0.001** | 0.506 | 1.000 | 0.601 | 1.000 |
| OTU_146 | 1.000 | **< 0.001** | **< 0.001** | **< 0.001** | **< 0.001** | **< 0.001** |  | 1.000 | **< 0.001** | **< 0.001** | **< 0.001** | **< 0.001** | 0.062 |  | 1.000 | **< 0.001** | **< 0.001** | **< 0.001** | **< 0.001** | 0.061 |
| OTU_1460 | 1.000 | 0.332 | 0.288 | 1.000 | 1.000 | 1.000 |  | 1.000 | 0.577 | 1.000 | 1.000 | 1.000 | 1.000 |  | 1.000 | **0.023** | 0.233 | 1.000 | 1.000 | 1.000 |
| OTU_1462 | 1.000 | 1.000 | 1.000 | 1.000 | 1.000 | 1.000 |  | 1.000 | 1.000 | 1.000 | 1.000 | 1.000 | 1.000 |  | 1.000 | 1.000 | 1.000 | 1.000 | 1.000 | 1.000 |
| OTU_1463 | 1.000 | 1.000 | 1.000 | 1.000 | 1.000 | 1.000 |  | 1.000 | **0.011** | **< 0.001** | 1.000 | 1.000 | 1.000 |  | 1.000 | 1.000 | 1.000 | 1.000 | 0.640 | 1.000 |
| OTU_1464 | 1.000 | 1.000 | 1.000 | 1.000 | 1.000 | 1.000 |  | 1.000 | 1.000 | 0.674 | 1.000 | 0.233 | 1.000 |  | 1.000 | 1.000 | 1.000 | 1.000 | 1.000 | 1.000 |
| OTU_1465 | **0.028** | **< 0.001** | 0.099 | 1.000 | 1.000 | 1.000 |  | **0.028** | **0.038** | 0.677 | 1.000 | 1.000 | 1.000 |  | 1.000 | 0.712 | 0.059 | 1.000 | 0.604 | 1.000 |
| OTU_1467 | 1.000 | 1.000 | 0.480 | 1.000 | 1.000 | 1.000 |  | 1.000 | **0.028** | **0.020** | 1.000 | 0.922 | 1.000 |  | 1.000 | 1.000 | 1.000 | 1.000 | 1.000 | 1.000 |
| OTU_1468 | 1.000 | 1.000 | **0.028** | 1.000 | **< 0.001** | 0.229 |  | 1.000 | 1.000 | **< 0.001** | 1.000 | 1.000 | 0.244 |  | 1.000 | 1.000 | **0.007** | 1.000 | 0.993 | **0.021** |
| OTU_1469 | 1.000 | 1.000 | 0.175 | 1.000 | **0.006** | 0.118 |  | 1.000 | 1.000 | 0.498 | 0.949 | **0.005** | 1.000 |  | 1.000 | 1.000 | 0.063 | 0.341 | **0.006** | 1.000 |
| OTU_147 | 1.000 | **0.022** | **0.008** | 0.124 | **0.039** | 1.000 |  | 1.000 | **< 0.001** | **< 0.001** | **< 0.001** | **< 0.001** | 1.000 |  | 1.000 | 0.058 | 1.000 | **0.002** | 1.000 | **0.048** |
| OTU_1474 | 0.360 | 1.000 | 0.252 | 1.000 | 1.000 | 1.000 |  | 0.360 | 1.000 | 1.000 | 1.000 | 1.000 | 1.000 |  | 1.000 | **0.002** | 0.306 | 0.063 | 1.000 | 1.000 |
| OTU_1475 | 0.252 | 1.000 | 1.000 | 0.722 | 0.375 | 1.000 |  | 0.252 | 0.932 | 1.000 | 1.000 | 1.000 | 1.000 |  | 1.000 | 1.000 | 1.000 | 1.000 | 1.000 | 1.000 |
| OTU_148 | 1.000 | 1.000 | **< 0.001** | 1.000 | **< 0.001** | **< 0.001** |  | 1.000 | 1.000 | **< 0.001** | 1.000 | **< 0.001** | **< 0.001** |  | 1.000 | 1.000 | **< 0.001** | 1.000 | **< 0.001** | **< 0.001** |
| OTU_1481 | 0.421 | 0.578 | 1.000 | 1.000 | 1.000 | 1.000 |  | 0.421 | 1.000 | 0.285 | 1.000 | 0.927 | 0.466 |  | 1.000 | 1.000 | 1.000 | 1.000 | 1.000 | 1.000 |
| OTU_1484 | 1.000 | 1.000 | 1.000 | 1.000 | 1.000 | 1.000 |  | 1.000 | 1.000 | 1.000 | 1.000 | 0.933 | 1.000 |  | 1.000 | 1.000 | 1.000 | 1.000 | 1.000 | 1.000 |
| OTU_1485 | 1.000 | 1.000 | 1.000 | 1.000 | 1.000 | 1.000 |  | 1.000 | 0.773 | 1.000 | 1.000 | 1.000 | 1.000 |  | 0.378 | 0.366 | 1.000 | 1.000 | 1.000 | 1.000 |
| OTU_149 | 0.089 | **< 0.001** | 0.699 | **0.004** | 1.000 | **< 0.001** |  | 0.089 | **< 0.001** | **< 0.001** | **< 0.001** | 1.000 | **< 0.001** |  | 0.855 | **< 0.001** | **0.042** | **0.001** | 1.000 | **0.024** |
| OTU_1491 | 1.000 | 0.252 | 0.217 | 1.000 | 1.000 | 1.000 |  | 1.000 | **0.029** | **0.010** | 1.000 | 1.000 | 1.000 |  | 1.000 | 1.000 | 1.000 | 1.000 | 1.000 | 1.000 |
| OTU_1496 | 1.000 | 1.000 | 1.000 | 1.000 | 1.000 | 1.000 |  | 1.000 | 0.749 | **0.025** | 1.000 | 0.728 | 1.000 |  | 1.000 | 1.000 | 1.000 | 1.000 | 1.000 | 1.000 |
| OTU_1499 | 0.151 | **0.011** | 1.000 | 1.000 | 0.470 | 0.130 |  | 0.151 | 0.340 | 1.000 | 1.000 | 0.066 | 0.110 |  | 1.000 | 0.089 | 1.000 | 0.590 | 1.000 | **0.004** |
| OTU_15 | 1.000 | 1.000 | 0.504 | 1.000 | 1.000 | 1.000 |  | 1.000 | **< 0.001** | **< 0.001** | **< 0.001** | **< 0.001** | **< 0.001** |  | 1.000 | 1.000 | 1.000 | 0.130 | 1.000 | 1.000 |
| OTU_150 | 1.000 | 1.000 | 1.000 | 1.000 | 1.000 | 1.000 |  | 1.000 | 0.626 | 0.079 | 1.000 | 1.000 | 1.000 |  | 1.000 | 0.623 | 0.115 | 1.000 | 1.000 | 1.000 |
| OTU_1501 | 1.000 | 1.000 | 1.000 | 1.000 | 1.000 | 1.000 |  | 1.000 | 1.000 | 1.000 | 1.000 | 0.092 | 1.000 |  | 1.000 | 1.000 | 1.000 | 1.000 | **0.034** | 0.091 |
| OTU_1503 | 0.082 | 0.317 | **0.044** | 1.000 | 1.000 | 1.000 |  | 0.082 | 1.000 | 1.000 | 1.000 | 1.000 | 1.000 |  | 1.000 | 0.493 | 0.330 | 1.000 | 1.000 | 1.000 |
| OTU_1505 | 1.000 | 1.000 | 0.209 | 1.000 | 1.000 | 1.000 |  | 1.000 | 1.000 | 1.000 | 1.000 | 1.000 | 1.000 |  | 1.000 | 1.000 | 1.000 | 1.000 | 1.000 | 1.000 |
| OTU_151 | **0.012** | 1.000 | **< 0.001** | 0.801 | **< 0.001** | **< 0.001** |  | **0.012** | 0.534 | **< 0.001** | 0.365 | **< 0.001** | **< 0.001** |  | 1.000 | 1.000 | **< 0.001** | 0.706 | **< 0.001** | **< 0.001** |
| OTU_1512 | **< 0.001** | **< 0.001** | **< 0.001** | 1.000 | 1.000 | 1.000 |  | **< 0.001** | **< 0.001** | **< 0.001** | 1.000 | 1.000 | 1.000 |  | **< 0.001** | **< 0.001** | **< 0.001** | 0.928 | 1.000 | 1.000 |
| OTU_1516 | 1.000 | 1.000 | 1.000 | 1.000 | 1.000 | 1.000 |  | 1.000 | 1.000 | 1.000 | 1.000 | 0.732 | 1.000 |  | 1.000 | 1.000 | 0.131 | 1.000 | 1.000 | 1.000 |
| OTU_1518 | 1.000 | 0.354 | 0.097 | 1.000 | 1.000 | 1.000 |  | 1.000 | **0.031** | **0.029** | 1.000 | 1.000 | 1.000 |  | 1.000 | 1.000 | 1.000 | 1.000 | 1.000 | 1.000 |
| OTU_1519 | 1.000 | 1.000 | 1.000 | 1.000 | 0.499 | 0.900 |  | 1.000 | 1.000 | 0.217 | 1.000 | 0.104 | 1.000 |  | 1.000 | 1.000 | 1.000 | 1.000 | 0.710 | 0.573 |
| OTU_152 | 1.000 | **< 0.001** | 0.216 | **< 0.001** | 1.000 | **< 0.001** |  | 1.000 | **< 0.001** | **< 0.001** | **< 0.001** | **< 0.001** | **< 0.001** |  | 1.000 | **< 0.001** | 0.350 | **< 0.001** | 1.000 | **< 0.001** |
| OTU_1520 | 0.267 | 0.174 | 1.000 | 1.000 | 1.000 | 1.000 |  | 0.267 | 1.000 | 1.000 | 1.000 | 1.000 | 1.000 |  | 1.000 | 0.180 | 0.496 | 1.000 | 1.000 | 1.000 |
| OTU_1524 | 1.000 | 1.000 | 1.000 | 1.000 | 1.000 | 1.000 |  | 1.000 | 0.053 | 0.123 | 0.150 | 0.264 | 1.000 |  | 1.000 | 1.000 | 1.000 | 1.000 | 1.000 | 1.000 |
| OTU_1529 | 1.000 | 1.000 | 1.000 | 1.000 | 0.164 | 1.000 |  | 1.000 | 0.111 | **< 0.001** | 0.162 | **< 0.001** | 1.000 |  | 1.000 | 1.000 | 1.000 | 1.000 | 0.169 | 1.000 |
| OTU_153 | **< 0.001** | **< 0.001** | **< 0.001** | **0.009** | **< 0.001** | **< 0.001** |  | **< 0.001** | **< 0.001** | **< 0.001** | **< 0.001** | **< 0.001** | **< 0.001** |  | **< 0.001** | **< 0.001** | **< 0.001** | **0.001** | **< 0.001** | 0.110 |
| OTU_1530 | 0.156 | **< 0.001** | **< 0.001** | 0.093 | 1.000 | 1.000 |  | 0.156 | **< 0.001** | **< 0.001** | **< 0.001** | 0.061 | 0.365 |  | 0.565 | **< 0.001** | **0.010** | **0.041** | 1.000 | 0.854 |
| OTU_1532 | 1.000 | 1.000 | **< 0.001** | 1.000 | 0.152 | 0.176 |  | 1.000 | 0.067 | **0.001** | 1.000 | 1.000 | 1.000 |  | 1.000 | **0.004** | **0.004** | 0.520 | 0.404 | 1.000 |
| OTU_1533 | 0.344 | 1.000 | 1.000 | 1.000 | **0.014** | 0.509 |  | 0.344 | 1.000 | 1.000 | 1.000 | 0.135 | 0.228 |  | 1.000 | 1.000 | 1.000 | 1.000 | 1.000 | 1.000 |
| OTU_1534 | 1.000 | 1.000 | 1.000 | 0.948 | 1.000 | 1.000 |  | 1.000 | 1.000 | 1.000 | 1.000 | 1.000 | 1.000 |  | 1.000 | 1.000 | 1.000 | 1.000 | 1.000 | 1.000 |
| OTU_1537 | 1.000 | 0.066 | 0.441 | 1.000 | 1.000 | 1.000 |  | 1.000 | **0.001** | 1.000 | 0.220 | 1.000 | **0.028** |  | 1.000 | 1.000 | 1.000 | 1.000 | 1.000 | 1.000 |
| OTU_1538 | 1.000 | 1.000 | 0.052 | 1.000 | 0.597 | 1.000 |  | 1.000 | 1.000 | 1.000 | 1.000 | 1.000 | 1.000 |  | 1.000 | 0.056 | **< 0.001** | 1.000 | **0.016** | 1.000 |
| OTU_1539 | 1.000 | 1.000 | 1.000 | 1.000 | 1.000 | 1.000 |  | 1.000 | 1.000 | 1.000 | 0.586 | 1.000 | 1.000 |  | 1.000 | 1.000 | 1.000 | 1.000 | 1.000 | 1.000 |
| OTU_154 | **0.004** | **< 0.001** | **< 0.001** | 1.000 | 1.000 | 1.000 |  | **0.004** | **< 0.001** | **< 0.001** | 1.000 | 0.650 | 1.000 |  | 0.916 | **< 0.001** | **< 0.001** | 0.840 | 0.809 | 1.000 |
| OTU_1542 | 1.000 | 1.000 | 1.000 | 1.000 | 1.000 | 1.000 |  | 1.000 | 1.000 | 0.432 | 1.000 | 1.000 | 1.000 |  | 1.000 | 1.000 | 1.000 | 1.000 | 1.000 | 1.000 |
| OTU_1544 | 0.376 | 0.136 | 1.000 | 1.000 | 1.000 | 1.000 |  | 0.376 | **< 0.001** | 1.000 | 1.000 | **0.009** | **0.016** |  | 0.411 | **0.008** | 1.000 | 1.000 | 1.000 | 0.293 |
| OTU_1548 | 1.000 | 0.181 | 1.000 | 1.000 | 1.000 | 1.000 |  | 1.000 | 1.000 | 1.000 | 1.000 | 1.000 | 1.000 |  | 1.000 | 1.000 | 1.000 | 1.000 | 1.000 | 1.000 |
| OTU_155 | 0.806 | 1.000 | **< 0.001** | **0.004** | **< 0.001** | **< 0.001** |  | 0.806 | 1.000 | **< 0.001** | **0.001** | **< 0.001** | **< 0.001** |  | 0.205 | 1.000 | **< 0.001** | **0.004** | **< 0.001** | **< 0.001** |
| OTU_1550 | 1.000 | 1.000 | 0.114 | 1.000 | 1.000 | 1.000 |  | 1.000 | 0.064 | 1.000 | 0.097 | 1.000 | 1.000 |  | 1.000 | 0.193 | **0.005** | 1.000 | 0.131 | 1.000 |
| OTU_1551 | 1.000 | 1.000 | 1.000 | 1.000 | 1.000 | 1.000 |  | 1.000 | 1.000 | 1.000 | 1.000 | 1.000 | 1.000 |  | 1.000 | 1.000 | 1.000 | 1.000 | 1.000 | 1.000 |
| OTU_1553 | 1.000 | 0.175 | 1.000 | **0.050** | 0.234 | 1.000 |  | 1.000 | 1.000 | 1.000 | 0.748 | 0.575 | 1.000 |  | 1.000 | 1.000 | 1.000 | 1.000 | 1.000 | 1.000 |
| OTU_1557 | 1.000 | **0.047** | 1.000 | 0.447 | 1.000 | 1.000 |  | 1.000 | 0.076 | 1.000 | 1.000 | 1.000 | 0.679 |  | 1.000 | 1.000 | 1.000 | 1.000 | 1.000 | 1.000 |
| OTU_1559 | 0.159 | 1.000 | 1.000 | 1.000 | 0.430 | 1.000 |  | 0.159 | 0.121 | 0.757 | 1.000 | 1.000 | 1.000 |  | 1.000 | 0.134 | 1.000 | 1.000 | 1.000 | 1.000 |
| OTU_156 | 0.501 | **< 0.001** | **< 0.001** | **< 0.001** | **< 0.001** | 1.000 |  | 0.501 | **< 0.001** | **< 0.001** | **< 0.001** | **< 0.001** | **0.001** |  | 1.000 | **0.004** | **< 0.001** | **< 0.001** | **< 0.001** | 1.000 |
| OTU_1564 | 1.000 | 1.000 | 1.000 | 1.000 | 1.000 | 1.000 |  | 1.000 | 1.000 | 1.000 | 1.000 | 1.000 | 1.000 |  | 1.000 | 1.000 | 1.000 | 1.000 | 1.000 | 1.000 |
| OTU_1565 | 0.461 | 1.000 | 1.000 | 0.261 | 1.000 | 1.000 |  | 0.461 | 0.438 | 1.000 | 1.000 | 0.425 | 1.000 |  | 0.262 | **< 0.001** | 0.093 | 1.000 | 1.000 | 1.000 |
| OTU_1566 | **0.036** | **< 0.001** | 0.140 | 1.000 | 1.000 | 0.640 |  | **0.036** | 0.481 | 1.000 | 0.992 | 1.000 | **0.043** |  | 1.000 | 1.000 | 1.000 | 1.000 | 1.000 | 0.636 |
| OTU_1567 | 1.000 | 1.000 | 1.000 | 1.000 | 1.000 | 1.000 |  | 1.000 | 1.000 | 1.000 | 1.000 | 1.000 | 1.000 |  | 1.000 | 1.000 | 1.000 | 1.000 | 1.000 | 1.000 |
| OTU_157 | 1.000 | 1.000 | 1.000 | 1.000 | 1.000 | 1.000 |  | 1.000 | 1.000 | 1.000 | 1.000 | 1.000 | 1.000 |  | 1.000 | 1.000 | 1.000 | 1.000 | 1.000 | 1.000 |
| OTU_1570 | 1.000 | 1.000 | 1.000 | 1.000 | 1.000 | 1.000 |  | 1.000 | **0.008** | **0.014** | 1.000 | 1.000 | 1.000 |  | 1.000 | **0.046** | 1.000 | 1.000 | 1.000 | 0.213 |
| OTU_1572 | 1.000 | 1.000 | 1.000 | 1.000 | 1.000 | 1.000 |  | 1.000 | 0.092 | 0.234 | 0.335 | 0.720 | 1.000 |  | 0.579 | 1.000 | 0.217 | 1.000 | 1.000 | 1.000 |
| OTU_1577 | 1.000 | 0.707 | 1.000 | 1.000 | 1.000 | 0.970 |  | 1.000 | 1.000 | 1.000 | 1.000 | 1.000 | 1.000 |  | 1.000 | 0.624 | 1.000 | 1.000 | 1.000 | 1.000 |
| OTU_158 | 1.000 | 0.143 | 1.000 | 0.689 | 1.000 | 1.000 |  | 1.000 | 0.497 | 1.000 | 0.136 | 1.000 | 1.000 |  | 1.000 | **0.005** | 0.787 | 0.118 | 1.000 | 1.000 |
| OTU_1580 | 1.000 | 0.526 | 0.438 | 1.000 | 1.000 | 1.000 |  | 1.000 | 1.000 | 1.000 | 1.000 | 1.000 | 1.000 |  | 1.000 | 1.000 | 1.000 | 1.000 | 1.000 | 1.000 |
| OTU_1581 | 1.000 | 1.000 | 1.000 | 1.000 | 1.000 | 1.000 |  | 1.000 | 1.000 | 1.000 | 1.000 | 1.000 | 1.000 |  | 0.311 | 1.000 | 1.000 | **0.009** | 0.253 | 1.000 |
| OTU_1582 | 1.000 | 1.000 | 1.000 | 1.000 | 1.000 | 1.000 |  | 1.000 | 1.000 | 1.000 | 1.000 | 1.000 | 1.000 |  | 1.000 | 1.000 | 1.000 | 1.000 | 1.000 | 1.000 |
| OTU_1583 | 1.000 | **0.006** | **0.002** | 1.000 | 1.000 | 1.000 |  | 1.000 | **0.011** | **0.002** | 1.000 | 1.000 | 1.000 |  | 1.000 | **0.002** | 0.074 | 0.484 | 1.000 | 1.000 |
| OTU_159 | 0.785 | **< 0.001** | **0.002** | 0.984 | 1.000 | 1.000 |  | 0.785 | **< 0.001** | **< 0.001** | 1.000 | 1.000 | 1.000 |  | 1.000 | **0.004** | **0.018** | 0.359 | 0.755 | 1.000 |
| OTU_1590 | 1.000 | 0.432 | 1.000 | 1.000 | 1.000 | 1.000 |  | 1.000 | 1.000 | 1.000 | 0.612 | 1.000 | 1.000 |  | 1.000 | 1.000 | 1.000 | 1.000 | 1.000 | 1.000 |
| OTU_1598 | 1.000 | 1.000 | **0.016** | 1.000 | **0.008** | 0.489 |  | 1.000 | 0.582 | **0.003** | 0.769 | **0.005** | 1.000 |  | 1.000 | 1.000 | 1.000 | 1.000 | 0.266 | 1.000 |
| OTU_16 | 1.000 | **< 0.001** | **< 0.001** | **< 0.001** | **< 0.001** | **< 0.001** |  | 1.000 | **< 0.001** | **< 0.001** | **< 0.001** | **< 0.001** | **0.008** |  | 1.000 | **< 0.001** | **< 0.001** | **< 0.001** | **< 0.001** | **< 0.001** |
| OTU_160 | 0.460 | **< 0.001** | **0.037** | 0.257 | 1.000 | 1.000 |  | 0.460 | 1.000 | 1.000 | **< 0.001** | **< 0.001** | 1.000 |  | **0.010** | 1.000 | 1.000 | 1.000 | 0.188 | 1.000 |
| OTU_1602 | **0.034** | **< 0.001** | **0.014** | 1.000 | 1.000 | 1.000 |  | **0.034** | 0.175 | 1.000 | 0.208 | 1.000 | 1.000 |  | 1.000 | **0.041** | 1.000 | 1.000 | 1.000 | 1.000 |
| OTU_1607 | 1.000 | 1.000 | **0.009** | 1.000 | **0.004** | **0.012** |  | 1.000 | 1.000 | 0.639 | 1.000 | **0.022** | **0.036** |  | 1.000 | 1.000 | **0.005** | 1.000 | 0.053 | 0.110 |
| OTU_1609 | 1.000 | 1.000 | 1.000 | 1.000 | 0.192 | 0.152 |  | 1.000 | 1.000 | **0.047** | 0.157 | 1.000 | 0.518 |  | 1.000 | 1.000 | **0.013** | 1.000 | 0.080 | 0.067 |
| OTU_161 | 1.000 | **0.045** | **0.001** | 1.000 | 0.773 | 1.000 |  | 1.000 | 0.076 | **0.025** | 1.000 | 1.000 | 1.000 |  | 0.117 | **< 0.001** | **0.008** | 1.000 | 1.000 | 1.000 |
| OTU_1611 | 0.432 | **0.009** | 1.000 | 1.000 | 1.000 | 0.529 |  | 0.432 | 0.110 | 0.642 | 1.000 | 1.000 | 1.000 |  | 1.000 | 1.000 | 1.000 | 1.000 | 1.000 | 1.000 |
| OTU_1612 | 1.000 | **0.028** | 0.102 | 1.000 | 1.000 | 1.000 |  | 1.000 | **< 0.001** | **0.007** | **0.034** | 1.000 | 1.000 |  | 1.000 | **0.021** | 1.000 | 0.177 | 1.000 | 1.000 |
| OTU_1618 | 1.000 | 1.000 | 1.000 | 1.000 | 1.000 | 1.000 |  | 1.000 | 1.000 | 1.000 | 1.000 | 1.000 | 1.000 |  | 1.000 | 1.000 | 1.000 | 1.000 | 1.000 | 1.000 |
| OTU_162 | 1.000 | 0.126 | **< 0.001** | 0.619 | **< 0.001** | 1.000 |  | 1.000 | **< 0.001** | **< 0.001** | 0.468 | **< 0.001** | 0.554 |  | 1.000 | **< 0.001** | **< 0.001** | **0.033** | **0.015** | 1.000 |
| OTU_1624 | 1.000 | 1.000 | 1.000 | 1.000 | 1.000 | 1.000 |  | 1.000 | 1.000 | 1.000 | 1.000 | 1.000 | 1.000 |  | 1.000 | 1.000 | 1.000 | 1.000 | 1.000 | 1.000 |
| OTU_1627 | **0.026** | 0.151 | 1.000 | 1.000 | **0.008** | 0.178 |  | **0.026** | 0.625 | 1.000 | 0.131 | 1.000 | 0.051 |  | 0.256 | 0.178 | 1.000 | 1.000 | 1.000 | 1.000 |
| OTU_163 | 0.074 | **< 0.001** | **0.003** | 1.000 | 1.000 | 1.000 |  | 0.074 | **< 0.001** | **< 0.001** | 1.000 | 1.000 | 1.000 |  | **< 0.001** | **0.007** | **0.003** | 1.000 | 1.000 | 1.000 |
| OTU_1638 | 1.000 | 0.432 | 1.000 | 0.378 | 1.000 | 1.000 |  | 1.000 | **< 0.001** | 1.000 | 1.000 | 0.659 | **0.025** |  | 1.000 | **0.032** | 1.000 | **0.047** | 1.000 | 0.315 |
| OTU_1639 | **< 0.001** | **< 0.001** | **< 0.001** | 1.000 | 1.000 | 1.000 |  | **< 0.001** | **< 0.001** | **< 0.001** | 1.000 | 1.000 | 1.000 |  | 0.396 | **< 0.001** | **< 0.001** | **0.010** | **0.003** | 1.000 |
| OTU_1648 | 1.000 | 0.153 | 0.134 | 0.385 | 0.261 | 1.000 |  | 1.000 | 1.000 | 1.000 | 0.219 | 1.000 | 1.000 |  | 1.000 | 1.000 | 1.000 | 1.000 | 1.000 | 1.000 |
| OTU_165 | 1.000 | 1.000 | 0.079 | 1.000 | 0.075 | 1.000 |  | 1.000 | **0.006** | **0.033** | **0.010** | **0.040** | 1.000 |  | 1.000 | 1.000 | 1.000 | 1.000 | 1.000 | 1.000 |
| OTU_1655 | 1.000 | 1.000 | **0.013** | 1.000 | **0.040** | 0.144 |  | 1.000 | 1.000 | **0.009** | 1.000 | 0.234 | 0.183 |  | 1.000 | 1.000 | 0.057 | 1.000 | **0.002** | **0.023** |
| OTU_1656 | 0.863 | **0.009** | 0.968 | 1.000 | 1.000 | 1.000 |  | 0.863 | **0.001** | 0.427 | **0.002** | 0.501 | 0.884 |  | 1.000 | **0.006** | 1.000 | **0.018** | 1.000 | 0.213 |
| OTU_1659 | 1.000 | 1.000 | **< 0.001** | 1.000 | **< 0.001** | 0.063 |  | 1.000 | 0.873 | **< 0.001** | 1.000 | **0.016** | 0.183 |  | 1.000 | 1.000 | **< 0.001** | 1.000 | **0.008** | **< 0.001** |
| OTU_166 | 1.000 | 1.000 | 1.000 | 0.737 | 1.000 | 1.000 |  | 1.000 | 1.000 | 0.619 | 1.000 | 1.000 | 1.000 |  | 1.000 | 1.000 | 1.000 | 1.000 | 1.000 | 1.000 |
| OTU_1660 | 1.000 | 0.493 | 1.000 | 0.070 | 1.000 | 1.000 |  | 1.000 | 1.000 | 1.000 | 1.000 | 1.000 | 1.000 |  | 1.000 | 1.000 | 1.000 | 1.000 | 1.000 | 1.000 |
| OTU_1667 | 1.000 | 0.585 | 0.305 | 0.278 | 0.105 | 1.000 |  | 1.000 | 1.000 | 1.000 | 1.000 | 1.000 | 1.000 |  | 1.000 | 1.000 | 1.000 | 1.000 | 1.000 | 1.000 |
| OTU_167 | 1.000 | **< 0.001** | **< 0.001** | 0.224 | **< 0.001** | 1.000 |  | 1.000 | **0.032** | **< 0.001** | **0.023** | **< 0.001** | 1.000 |  | 1.000 | **< 0.001** | **< 0.001** | **0.012** | **< 0.001** | 1.000 |
| OTU_1671 | 1.000 | 1.000 | 1.000 | 1.000 | 1.000 | 1.000 |  | 1.000 | 1.000 | 1.000 | 1.000 | 1.000 | 1.000 |  | 1.000 | 1.000 | 1.000 | 1.000 | 1.000 | 1.000 |
| OTU_1676 | 1.000 | 1.000 | **0.046** | 1.000 | 1.000 | 0.301 |  | 1.000 | 0.063 | 1.000 | 1.000 | 0.322 | 0.132 |  | 1.000 | 1.000 | 1.000 | 1.000 | 0.460 | 0.406 |
| OTU_168 | **< 0.001** | 0.885 | **< 0.001** | 0.064 | 1.000 | **0.014** |  | **< 0.001** | 0.071 | 1.000 | 0.320 | 1.000 | 0.196 |  | 1.000 | 1.000 | 1.000 | 1.000 | 1.000 | 1.000 |
| OTU_169 | 1.000 | 0.078 | 1.000 | 1.000 | 1.000 | 1.000 |  | 1.000 | **0.001** | 0.411 | 0.480 | 1.000 | 1.000 |  | 0.596 | **< 0.001** | 1.000 | 0.090 | **0.041** | **< 0.001** |
| OTU_1697 | 1.000 | 0.900 | 1.000 | 1.000 | 1.000 | 1.000 |  | 1.000 | 1.000 | 1.000 | 1.000 | 1.000 | 1.000 |  | 1.000 | 1.000 | 1.000 | 1.000 | 1.000 | 1.000 |
| OTU_17 | 1.000 | **< 0.001** | **0.002** | **< 0.001** | **0.001** | **0.041** |  | 1.000 | **< 0.001** | **< 0.001** | **< 0.001** | **< 0.001** | 0.108 |  | 1.000 | **< 0.001** | **< 0.001** | **< 0.001** | **< 0.001** | 1.000 |
| OTU_1704 | 1.000 | **< 0.001** | **0.031** | **0.014** | 1.000 | 1.000 |  | 1.000 | **< 0.001** | **< 0.001** | **0.001** | **0.005** | 1.000 |  | 1.000 | **0.040** | 0.089 | 0.520 | 0.815 | 1.000 |
| OTU_1705 | 1.000 | 0.967 | 1.000 | 1.000 | 1.000 | 1.000 |  | 1.000 | 0.608 | 1.000 | 0.542 | 1.000 | 1.000 |  | 1.000 | 0.524 | 1.000 | 1.000 | 1.000 | 1.000 |
| OTU_1706 | 0.137 | 0.089 | 1.000 | 1.000 | **0.037** | 0.080 |  | 0.137 | 1.000 | 1.000 | 0.091 | **0.043** | 1.000 |  | 1.000 | 1.000 | 1.000 | 1.000 | 1.000 | 1.000 |
| OTU_1708 | 0.639 | **< 0.001** | **0.005** | 0.527 | 1.000 | 1.000 |  | 0.639 | **0.013** | **0.025** | 1.000 | 1.000 | 1.000 |  | 1.000 | 1.000 | 1.000 | 1.000 | 1.000 | 1.000 |
| OTU_171 | 1.000 | 0.742 | 1.000 | 1.000 | 1.000 | 1.000 |  | 1.000 | 0.238 | 1.000 | 1.000 | 1.000 | 1.000 |  | 1.000 | **0.003** | 0.580 | 0.541 | 1.000 | 1.000 |
| OTU_1712 | 1.000 | **0.028** | 1.000 | 1.000 | 1.000 | 0.762 |  | 1.000 | 1.000 | 1.000 | 1.000 | 1.000 | 1.000 |  | 1.000 | 0.471 | 1.000 | 1.000 | 1.000 | 1.000 |
| OTU_1713 | 1.000 | 0.094 | **< 0.001** | 0.127 | **< 0.001** | 0.758 |  | 1.000 | 0.115 | **0.002** | 1.000 | 0.238 | 1.000 |  | 1.000 | **0.011** | **< 0.001** | **0.001** | **< 0.001** | 1.000 |
| OTU_1718 | 1.000 | 1.000 | 1.000 | 1.000 | 1.000 | 1.000 |  | 1.000 | 1.000 | 1.000 | 1.000 | 1.000 | 1.000 |  | 1.000 | 1.000 | 1.000 | 1.000 | 1.000 | 1.000 |
| OTU_172 | 1.000 | 0.224 | **< 0.001** | 0.102 | **< 0.001** | **0.026** |  | 1.000 | 0.060 | **0.002** | **0.017** | **< 0.001** | 1.000 |  | 1.000 | 0.189 | **0.010** | **0.006** | **< 0.001** | 1.000 |
| OTU_1727 | 1.000 | 1.000 | 1.000 | 1.000 | 1.000 | 1.000 |  | 1.000 | 1.000 | 1.000 | 0.281 | **0.004** | 1.000 |  | 1.000 | 1.000 | 1.000 | 1.000 | 0.262 | 0.432 |
| OTU_173 | 1.000 | **0.029** | 1.000 | 1.000 | 1.000 | 1.000 |  | 1.000 | **< 0.001** | **< 0.001** | 1.000 | 1.000 | 1.000 |  | **0.047** | **< 0.001** | **< 0.001** | 0.319 | 0.925 | 1.000 |
| OTU_1735 | 1.000 | 0.060 | 1.000 | 1.000 | 1.000 | 1.000 |  | 1.000 | 1.000 | 1.000 | 1.000 | 1.000 | 1.000 |  | 0.598 | 1.000 | 1.000 | 1.000 | 1.000 | 1.000 |
| OTU_1736 | 1.000 | 1.000 | 1.000 | 0.697 | 1.000 | 0.159 |  | 1.000 | 0.694 | 1.000 | 0.162 | 1.000 | 1.000 |  | 1.000 | 1.000 | 1.000 | 1.000 | 1.000 | 0.622 |
| OTU_1737 | 0.643 | 0.259 | 1.000 | 1.000 | 0.282 | 0.306 |  | 0.643 | 1.000 | 1.000 | 1.000 | 1.000 | 1.000 |  | 0.358 | 0.594 | 1.000 | 1.000 | 0.573 | 1.000 |
| OTU_174 | **0.011** | **< 0.001** | 0.140 | **< 0.001** | 1.000 | **< 0.001** |  | **0.011** | **< 0.001** | **< 0.001** | **< 0.001** | 1.000 | **< 0.001** |  | **0.004** | **< 0.001** | **0.019** | **< 0.001** | 1.000 | **< 0.001** |
| OTU_1745 | **< 0.001** | **< 0.001** | **< 0.001** | 1.000 | 1.000 | 1.000 |  | **< 0.001** | **< 0.001** | 0.110 | 1.000 | 0.270 | 0.436 |  | **< 0.001** | **< 0.001** | **< 0.001** | 1.000 | 1.000 | 1.000 |
| OTU_175 | 1.000 | 1.000 | 1.000 | 1.000 | 1.000 | 1.000 |  | 1.000 | **< 0.001** | **< 0.001** | 0.741 | **0.001** | 1.000 |  | 1.000 | 0.329 | 1.000 | 1.000 | 1.000 | **0.004** |
| OTU_1751 | 1.000 | 1.000 | **< 0.001** | 1.000 | **0.012** | **0.023** |  | 1.000 | 1.000 | 0.776 | 1.000 | **0.029** | 0.077 |  | 1.000 | 1.000 | 0.278 | 1.000 | **0.002** | 1.000 |
| OTU_1754 | 1.000 | 1.000 | 1.000 | 1.000 | 1.000 | 0.957 |  | 1.000 | 1.000 | 1.000 | 1.000 | 1.000 | 1.000 |  | 1.000 | 1.000 | 1.000 | 1.000 | 1.000 | 1.000 |
| OTU_1755 | 1.000 | 1.000 | 1.000 | 0.378 | 0.490 | 1.000 |  | 1.000 | 1.000 | 1.000 | 1.000 | 1.000 | 1.000 |  | 1.000 | 1.000 | **0.014** | 1.000 | **0.015** | 1.000 |
| OTU_1756 | **< 0.001** | **< 0.001** | **< 0.001** | **0.021** | **0.042** | 1.000 |  | **< 0.001** | **< 0.001** | **< 0.001** | **< 0.001** | 1.000 | 0.493 |  | **< 0.001** | **< 0.001** | **< 0.001** | 0.077 | 1.000 | 1.000 |
| OTU_1757 | 1.000 | 1.000 | 1.000 | 1.000 | 1.000 | 1.000 |  | 1.000 | 1.000 | 1.000 | 0.658 | **0.015** | 1.000 |  | 1.000 | 1.000 | 1.000 | 1.000 | 1.000 | 1.000 |
| OTU_1758 | 1.000 | 1.000 | 1.000 | 1.000 | 1.000 | 1.000 |  | 1.000 | **0.009** | 1.000 | 0.099 | 1.000 | 1.000 |  | 1.000 | 1.000 | 1.000 | 0.463 | 1.000 | 1.000 |
| OTU_176 | 1.000 | **0.008** | 0.061 | 0.054 | 0.230 | 1.000 |  | 1.000 | 0.086 | 0.205 | **0.002** | **0.005** | 1.000 |  | 1.000 | **0.002** | **< 0.001** | 1.000 | 0.271 | 1.000 |
| OTU_1760 | 1.000 | 1.000 | 0.059 | 1.000 | 0.752 | 1.000 |  | 1.000 | 0.123 | **0.002** | 0.178 | **0.003** | 1.000 |  | 1.000 | 1.000 | 1.000 | 1.000 | 0.826 | 1.000 |
| OTU_1761 | 1.000 | 1.000 | 1.000 | 1.000 | 1.000 | 1.000 |  | 1.000 | 1.000 | 1.000 | 0.305 | 0.168 | 1.000 |  | 1.000 | 1.000 | 1.000 | 0.821 | 0.194 | 1.000 |
| OTU_1763 | 1.000 | 1.000 | 0.080 | 1.000 | 0.055 | 1.000 |  | 1.000 | 1.000 | 1.000 | 1.000 | 0.051 | 0.522 |  | 1.000 | 1.000 | 1.000 | 1.000 | 1.000 | 1.000 |
| OTU_1767 | 0.220 | **0.011** | **0.002** | 1.000 | 1.000 | 1.000 |  | 0.220 | **< 0.001** | **0.004** | 1.000 | 1.000 | 1.000 |  | 0.537 | **0.004** | **0.015** | 1.000 | 1.000 | 1.000 |
| OTU_1769 | 0.254 | 1.000 | 1.000 | 1.000 | 0.270 | 1.000 |  | 0.254 | 1.000 | 0.154 | 1.000 | 0.088 | 1.000 |  | 1.000 | 1.000 | 1.000 | 1.000 | 0.274 | 1.000 |
| OTU_177 | 1.000 | **0.025** | 1.000 | 0.111 | 1.000 | **0.027** |  | 1.000 | **< 0.001** | **< 0.001** | 0.096 | 1.000 | 0.526 |  | 1.000 | **0.002** | 1.000 | 0.316 | 1.000 | **0.008** |
| OTU_1772 | 1.000 | 0.901 | 1.000 | 1.000 | 1.000 | 1.000 |  | 1.000 | 1.000 | 1.000 | 1.000 | 1.000 | 1.000 |  | 1.000 | 1.000 | 1.000 | 1.000 | 1.000 | 1.000 |
| OTU_1773 | 1.000 | 0.390 | 0.346 | 1.000 | 1.000 | 1.000 |  | 1.000 | 1.000 | 1.000 | 1.000 | 1.000 | 1.000 |  | 1.000 | 1.000 | 1.000 | 1.000 | 1.000 | 1.000 |
| OTU_1775 | 1.000 | 1.000 | 1.000 | 1.000 | 0.693 | 0.463 |  | 1.000 | 1.000 | 1.000 | 1.000 | 0.459 | 1.000 |  | 0.524 | 0.673 | 1.000 | 1.000 | 0.208 | 0.948 |
| OTU_1776 | 1.000 | 1.000 | **< 0.001** | 0.917 | **< 0.001** | 0.076 |  | 1.000 | 0.052 | **< 0.001** | 1.000 | 0.079 | 0.179 |  | 1.000 | 1.000 | 0.199 | 1.000 | 0.956 | 1.000 |
| OTU_1777 | 1.000 | 1.000 | 1.000 | 1.000 | 1.000 | 1.000 |  | 1.000 | 1.000 | 1.000 | 1.000 | 1.000 | 1.000 |  | 1.000 | 1.000 | 1.000 | 1.000 | 1.000 | 1.000 |
| OTU_178 | 1.000 | **< 0.001** | 0.052 | **< 0.001** | 1.000 | **< 0.001** |  | 1.000 | **< 0.001** | **< 0.001** | **< 0.001** | 0.061 | **0.038** |  | 1.000 | **< 0.001** | **< 0.001** | **< 0.001** | 0.185 | 0.110 |
| OTU_179 | 1.000 | 1.000 | 1.000 | 1.000 | 1.000 | 1.000 |  | 1.000 | **< 0.001** | **< 0.001** | 1.000 | **0.003** | 1.000 |  | 0.960 | 1.000 | 1.000 | 1.000 | 1.000 | 1.000 |
| OTU_1796 | 1.000 | 1.000 | 0.942 | 1.000 | 1.000 | 1.000 |  | 1.000 | 1.000 | 1.000 | 1.000 | 1.000 | 1.000 |  | 1.000 | 1.000 | 1.000 | 1.000 | 1.000 | 1.000 |
| OTU_18 | **0.025** | **< 0.001** | **< 0.001** | 1.000 | **< 0.001** | **< 0.001** |  | **0.025** | **< 0.001** | **0.002** | 1.000 | **< 0.001** | **< 0.001** |  | 0.138 | **< 0.001** | **< 0.001** | 1.000 | **< 0.001** | **< 0.001** |
| OTU_180 | 1.000 | 0.447 | 1.000 | 1.000 | 1.000 | 1.000 |  | 1.000 | 1.000 | 1.000 | 1.000 | 1.000 | 1.000 |  | 1.000 | **0.002** | **0.014** | **0.033** | 0.133 | 1.000 |
| OTU_1800 | 1.000 | 1.000 | 1.000 | 1.000 | 1.000 | 1.000 |  | 1.000 | 1.000 | 1.000 | 1.000 | 1.000 | 1.000 |  | 1.000 | 1.000 | 1.000 | 1.000 | 0.222 | 0.084 |
| OTU_1803 | 1.000 | 1.000 | 1.000 | 1.000 | 1.000 | 1.000 |  | 1.000 | 0.120 | 0.369 | 1.000 | 1.000 | 1.000 |  | 1.000 | 0.109 | 0.207 | 1.000 | 1.000 | 1.000 |
| OTU_1804 | 1.000 | 1.000 | **0.006** | 1.000 | **0.006** | **0.021** |  | 1.000 | 1.000 | 0.140 | 1.000 | 1.000 | 1.000 |  | 1.000 | 1.000 | 0.222 | 0.144 | **< 0.001** | 1.000 |
| OTU_1809 | 1.000 | 0.750 | 1.000 | 1.000 | 1.000 | 1.000 |  | 1.000 | 1.000 | 1.000 | 0.833 | 1.000 | 1.000 |  | 1.000 | **0.041** | **0.015** | **0.011** | **0.002** | 1.000 |
| OTU_181 | 0.756 | 1.000 | 1.000 | 0.403 | **0.025** | 1.000 |  | 0.756 | 1.000 | 1.000 | 0.155 | 1.000 | 1.000 |  | 1.000 | 0.056 | 0.447 | 0.060 | 0.377 | 1.000 |
| OTU_182 | 0.074 | **0.005** | 0.070 | 1.000 | 1.000 | 1.000 |  | 0.074 | **< 0.001** | **< 0.001** | 0.374 | 1.000 | 1.000 |  | 1.000 | **< 0.001** | **< 0.001** | **0.005** | 0.227 | 1.000 |
| OTU_1820 | 1.000 | 1.000 | 0.964 | 1.000 | 1.000 | 1.000 |  | 1.000 | 0.179 | **0.036** | 1.000 | 1.000 | 1.000 |  | 0.945 | 0.534 | 1.000 | 1.000 | 1.000 | 1.000 |
| OTU_1823 | 0.419 | 1.000 | 1.000 | 1.000 | 1.000 | 1.000 |  | 0.419 | 1.000 | 1.000 | 0.462 | 1.000 | 1.000 |  | 1.000 | 1.000 | 1.000 | 1.000 | 1.000 | 1.000 |
| OTU_1824 | 1.000 | 1.000 | 1.000 | 1.000 | 0.717 | 1.000 |  | 1.000 | 1.000 | 0.152 | 1.000 | 0.177 | 1.000 |  | 1.000 | 1.000 | 1.000 | 1.000 | 1.000 | 1.000 |
| OTU_1828 | 0.334 | **0.042** | 1.000 | 1.000 | 1.000 | 0.543 |  | 0.334 | 1.000 | 1.000 | 1.000 | 0.732 | 0.867 |  | 1.000 | 1.000 | 1.000 | 1.000 | 1.000 | 1.000 |
| OTU_183 | 1.000 | **0.022** | 0.572 | 0.801 | 1.000 | 1.000 |  | 1.000 | 1.000 | 0.632 | 1.000 | 1.000 | 1.000 |  | 1.000 | 0.191 | 0.084 | 1.000 | 1.000 | 1.000 |
| OTU_1838 | 1.000 | 1.000 | 1.000 | 1.000 | 1.000 | 1.000 |  | 1.000 | 0.642 | 1.000 | 0.817 | 1.000 | 1.000 |  | 1.000 | 1.000 | 0.408 | 1.000 | 0.722 | 1.000 |
| OTU_184 | 1.000 | 1.000 | 1.000 | 1.000 | 1.000 | 1.000 |  | 1.000 | 1.000 | 1.000 | 1.000 | 1.000 | 1.000 |  | 1.000 | 1.000 | 1.000 | 1.000 | 1.000 | 1.000 |
| OTU_1847 | 1.000 | 0.286 | 1.000 | 1.000 | 1.000 | 0.787 |  | 1.000 | 0.688 | 1.000 | 0.482 | 1.000 | 0.084 |  | 1.000 | 0.592 | 1.000 | 1.000 | 1.000 | 0.321 |
| OTU_185 | **0.006** | **< 0.001** | **< 0.001** | **0.002** | **0.002** | 1.000 |  | **0.006** | **< 0.001** | **< 0.001** | 1.000 | **0.003** | 1.000 |  | **< 0.001** | **< 0.001** | **< 0.001** | 0.362 | 1.000 | 1.000 |
| OTU_1853 | 1.000 | 0.133 | **0.001** | 0.055 | **< 0.001** | 1.000 |  | 1.000 | **< 0.001** | **< 0.001** | **0.032** | **0.006** | 1.000 |  | 1.000 | **0.005** | **0.002** | 0.341 | 0.123 | 1.000 |
| OTU_186 | 0.254 | **< 0.001** | **< 0.001** | **< 0.001** | **< 0.001** | 1.000 |  | 0.254 | **< 0.001** | **< 0.001** | **< 0.001** | 0.125 | 0.212 |  | 0.227 | **< 0.001** | **< 0.001** | **0.007** | **< 0.001** | 1.000 |
| OTU_1865 | 1.000 | 1.000 | 1.000 | 1.000 | 1.000 | 1.000 |  | 1.000 | 1.000 | 1.000 | 1.000 | 1.000 | 1.000 |  | 1.000 | 1.000 | 1.000 | 1.000 | 1.000 | 1.000 |
| OTU_1866 | 1.000 | 1.000 | 1.000 | 1.000 | 1.000 | 1.000 |  | 1.000 | 1.000 | 1.000 | 1.000 | 1.000 | 1.000 |  | 1.000 | 1.000 | 0.900 | 1.000 | 1.000 | 1.000 |
| OTU_1869 | 1.000 | 1.000 | 1.000 | 1.000 | 0.986 | 1.000 |  | 1.000 | 1.000 | 0.348 | 0.262 | **0.019** | 1.000 |  | 1.000 | 1.000 | 0.287 | 1.000 | 1.000 | 1.000 |
| OTU_187 | 1.000 | 1.000 | 1.000 | 1.000 | 1.000 | 0.827 |  | 1.000 | 1.000 | 1.000 | 0.991 | 1.000 | 1.000 |  | 1.000 | 1.000 | 1.000 | 1.000 | 1.000 | 1.000 |
| OTU_1870 | 0.639 | 0.469 | 0.400 | 1.000 | 1.000 | 1.000 |  | 0.639 | 1.000 | 1.000 | 1.000 | 1.000 | 1.000 |  | 0.352 | 1.000 | 1.000 | 1.000 | 1.000 | 1.000 |
| OTU_1877 | 1.000 | 1.000 | 1.000 | 1.000 | 1.000 | 1.000 |  | 1.000 | 1.000 | 1.000 | 1.000 | 1.000 | 1.000 |  | 0.441 | 0.836 | 1.000 | 1.000 | 1.000 | 1.000 |
| OTU_188 | 1.000 | 1.000 | 1.000 | 1.000 | 1.000 | 1.000 |  | 1.000 | 0.371 | 1.000 | 1.000 | 1.000 | 1.000 |  | 1.000 | **0.025** | 1.000 | 1.000 | 1.000 | 0.322 |
| OTU_1880 | 1.000 | 1.000 | 0.209 | 1.000 | 1.000 | 1.000 |  | 1.000 | 1.000 | 1.000 | 1.000 | 1.000 | 1.000 |  | 1.000 | 1.000 | 1.000 | 1.000 | 1.000 | 1.000 |
| OTU_1885 | 1.000 | 1.000 | **0.006** | 1.000 | **0.035** | 0.406 |  | 1.000 | 1.000 | 1.000 | 1.000 | 0.220 | 1.000 |  | 1.000 | 1.000 | 1.000 | 1.000 | 1.000 | 0.393 |
| OTU_1886 | 1.000 | 1.000 | 1.000 | 1.000 | 1.000 | 1.000 |  | 1.000 | 1.000 | 1.000 | 1.000 | 1.000 | 1.000 |  | 1.000 | 0.556 | 1.000 | 1.000 | 1.000 | 1.000 |
| OTU_189 | 1.000 | 1.000 | **< 0.001** | 1.000 | **< 0.001** | **< 0.001** |  | 1.000 | 1.000 | **< 0.001** | **0.047** | **< 0.001** | **< 0.001** |  | 1.000 | 1.000 | **< 0.001** | 1.000 | **< 0.001** | **0.005** |
| OTU_1893 | 0.786 | **< 0.001** | **0.003** | **< 0.001** | 1.000 | **0.021** |  | 0.786 | **0.008** | 0.299 | **0.043** | 0.830 | 1.000 |  | 1.000 | **< 0.001** | 0.087 | 0.081 | 1.000 | 0.670 |
| OTU_1896 | 1.000 | 1.000 | 0.087 | 1.000 | 0.074 | 0.057 |  | 1.000 | 1.000 | **0.031** | 0.315 | 1.000 | 0.115 |  | 1.000 | 1.000 | 0.302 | 1.000 | 0.403 | **0.017** |
| OTU_1897 | 1.000 | 1.000 | 1.000 | 1.000 | 1.000 | 1.000 |  | 1.000 | 1.000 | 1.000 | 1.000 | 1.000 | 1.000 |  | 1.000 | 1.000 | 1.000 | 1.000 | 1.000 | 1.000 |
| OTU_19 | 1.000 | **< 0.001** | **< 0.001** | **< 0.001** | **< 0.001** | **< 0.001** |  | 1.000 | 0.252 | **< 0.001** | **0.001** | **< 0.001** | **< 0.001** |  | 1.000 | **< 0.001** | **< 0.001** | **< 0.001** | **< 0.001** | **< 0.001** |
| OTU_190 | 1.000 | **< 0.001** | **< 0.001** | 0.340 | **0.004** | 1.000 |  | 1.000 | **< 0.001** | **< 0.001** | **< 0.001** | **< 0.001** | 1.000 |  | 1.000 | **< 0.001** | **< 0.001** | **0.048** | **0.044** | 1.000 |
| OTU_1901 | 0.798 | 0.437 | 1.000 | 1.000 | 1.000 | 1.000 |  | 0.798 | 1.000 | 0.259 | 1.000 | 0.909 | 1.000 |  | 1.000 | 1.000 | 1.000 | 1.000 | 1.000 | 1.000 |
| OTU_1903 | 1.000 | 1.000 | 1.000 | 1.000 | 1.000 | 0.504 |  | 1.000 | 1.000 | **0.030** | 1.000 | **0.041** | 0.231 |  | 1.000 | 1.000 | 1.000 | 1.000 | 1.000 | 1.000 |
| OTU_1908 | 1.000 | 1.000 | 0.441 | 1.000 | 0.056 | 0.875 |  | 1.000 | 0.183 | **< 0.001** | 0.265 | **< 0.001** | 1.000 |  | 1.000 | 1.000 | 0.205 | 1.000 | 1.000 | 1.000 |
| OTU_191 | 1.000 | 0.477 | **0.002** | 0.062 | **< 0.001** | 1.000 |  | 1.000 | 1.000 | 0.246 | 1.000 | 0.104 | 1.000 |  | 1.000 | 1.000 | **< 0.001** | 1.000 | **< 0.001** | **0.028** |
| OTU_1910 | 1.000 | 0.114 | **0.003** | **0.023** | **< 0.001** | 1.000 |  | 1.000 | 0.096 | **0.009** | 0.142 | **0.011** | 1.000 |  | 1.000 | **0.003** | **< 0.001** | **0.005** | **< 0.001** | 1.000 |
| OTU_1913 | 1.000 | 1.000 | 1.000 | 1.000 | 0.764 | 1.000 |  | 1.000 | 1.000 | 1.000 | 1.000 | 1.000 | 1.000 |  | 1.000 | 1.000 | 0.787 | 1.000 | 1.000 | 1.000 |
| OTU_192 | 1.000 | 0.282 | **< 0.001** | 0.795 | **< 0.001** | 0.310 |  | 1.000 | **0.002** | **< 0.001** | 1.000 | **0.038** | 0.337 |  | 1.000 | 0.393 | **0.002** | 1.000 | **0.025** | 1.000 |
| OTU_1924 | 1.000 | 1.000 | 1.000 | 1.000 | 1.000 | 1.000 |  | 1.000 | 1.000 | 1.000 | 1.000 | 1.000 | 1.000 |  | 1.000 | 1.000 | 1.000 | 1.000 | 1.000 | 1.000 |
| OTU_1925 | 0.952 | 0.171 | 0.557 | 1.000 | 1.000 | 1.000 |  | 0.952 | 1.000 | 1.000 | 1.000 | 1.000 | 1.000 |  | 1.000 | 1.000 | 0.461 | 1.000 | 1.000 | 1.000 |
| OTU_193 | 1.000 | **< 0.001** | 1.000 | **0.006** | 1.000 | **< 0.001** |  | 1.000 | **< 0.001** | **0.020** | **< 0.001** | 1.000 | **< 0.001** |  | 1.000 | **0.016** | 1.000 | 1.000 | 1.000 | 0.116 |
| OTU_194 | 1.000 | 1.000 | **0.008** | 1.000 | **< 0.001** | **0.002** |  | 1.000 | 1.000 | **0.018** | **0.001** | **< 0.001** | 1.000 |  | 1.000 | 1.000 | 1.000 | 1.000 | 1.000 | 1.000 |
| OTU_1940 | 0.313 | **< 0.001** | **< 0.001** | 0.534 | 0.247 | 1.000 |  | 0.313 | 0.067 | **< 0.001** | 1.000 | 0.527 | 1.000 |  | **0.045** | **< 0.001** | **< 0.001** | **0.027** | 0.624 | 1.000 |
| OTU_195 | **< 0.001** | **< 0.001** | **< 0.001** | 1.000 | **0.050** | 1.000 |  | **< 0.001** | **0.007** | **< 0.001** | 1.000 | 1.000 | 1.000 |  | 1.000 | 0.912 | **0.005** | 1.000 | 0.513 | 1.000 |
| OTU_1958 | 1.000 | 1.000 | 1.000 | 1.000 | 1.000 | 1.000 |  | 1.000 | 1.000 | 1.000 | 1.000 | 1.000 | 1.000 |  | 1.000 | 1.000 | 1.000 | 1.000 | 1.000 | 1.000 |
| OTU_196 | **< 0.001** | **< 0.001** | **< 0.001** | 1.000 | 1.000 | 1.000 |  | **< 0.001** | **< 0.001** | **< 0.001** | 1.000 | 1.000 | 1.000 |  | **< 0.001** | **< 0.001** | 0.196 | 1.000 | 0.292 | **0.004** |
| OTU_1967 | 0.740 | **< 0.001** | **0.008** | **0.050** | 1.000 | 1.000 |  | 0.740 | **0.013** | 0.140 | 0.439 | 1.000 | 1.000 |  | 0.433 | 0.059 | 1.000 | 1.000 | 1.000 | 1.000 |
| OTU_197 | 1.000 | 1.000 | **< 0.001** | 1.000 | **< 0.001** | **< 0.001** |  | 1.000 | 0.121 | **< 0.001** | 0.620 | **< 0.001** | 0.177 |  | 1.000 | 0.145 | **< 0.001** | 1.000 | **< 0.001** | **0.043** |
| OTU_1975 | 1.000 | 1.000 | 0.473 | 1.000 | 0.196 | 1.000 |  | 1.000 | 1.000 | **0.037** | 1.000 | 0.306 | 1.000 |  | 1.000 | 1.000 | 1.000 | 1.000 | 1.000 | 1.000 |
| OTU_198 | 1.000 | 1.000 | 0.110 | **0.042** | **< 0.001** | 1.000 |  | 1.000 | 1.000 | 1.000 | 0.060 | 0.095 | 1.000 |  | 0.433 | 1.000 | 1.000 | 0.836 | **0.003** | 1.000 |
| OTU_1980 | 1.000 | 1.000 | 1.000 | 1.000 | 1.000 | 1.000 |  | 1.000 | 0.282 | 1.000 | 1.000 | 1.000 | 1.000 |  | 1.000 | **0.008** | 0.749 | 0.274 | 1.000 | 1.000 |
| OTU_1985 | 1.000 | 1.000 | 1.000 | 1.000 | 1.000 | 1.000 |  | 1.000 | 1.000 | 1.000 | 1.000 | 1.000 | 1.000 |  | 1.000 | 1.000 | 1.000 | 1.000 | 1.000 | 1.000 |
| OTU_1994 | **0.034** | 1.000 | **0.018** | 0.372 | 1.000 | 0.469 |  | **0.034** | 0.387 | 0.305 | 1.000 | 1.000 | 1.000 |  | 1.000 | 1.000 | 1.000 | 1.000 | 1.000 | 1.000 |
| OTU_1998 | 0.344 | 0.224 | 1.000 | 1.000 | 1.000 | 1.000 |  | 0.344 | **0.029** | **0.011** | 1.000 | 1.000 | 1.000 |  | 1.000 | 1.000 | 1.000 | 1.000 | 1.000 | 1.000 |
| OTU_1999 | 1.000 | 1.000 | 1.000 | 1.000 | 0.170 | 1.000 |  | 1.000 | 0.252 | 1.000 | 1.000 | 1.000 | 0.364 |  | 1.000 | 1.000 | 1.000 | 1.000 | 1.000 | 1.000 |
| OTU_20 | 1.000 | 0.754 | **< 0.001** | 1.000 | **< 0.001** | **< 0.001** |  | 1.000 | 0.224 | **0.004** | 1.000 | **< 0.001** | **< 0.001** |  | 1.000 | 1.000 | **< 0.001** | 1.000 | **< 0.001** | **< 0.001** |
| OTU_200 | 1.000 | 0.079 | 0.204 | 1.000 | 1.000 | 1.000 |  | 1.000 | 1.000 | 0.471 | 1.000 | 0.528 | 1.000 |  | 1.000 | **0.044** | 0.115 | 1.000 | 1.000 | 1.000 |
| OTU_2005 | 1.000 | 1.000 | 1.000 | 1.000 | 1.000 | 1.000 |  | 1.000 | 1.000 | 0.310 | 1.000 | 1.000 | 1.000 |  | 1.000 | 0.156 | 0.257 | 1.000 | 1.000 | 1.000 |
| OTU_201 | **0.002** | **< 0.001** | **< 0.001** | **0.002** | **0.002** | 1.000 |  | **0.002** | **< 0.001** | **< 0.001** | 0.052 | **< 0.001** | 1.000 |  | **< 0.001** | **< 0.001** | **< 0.001** | 1.000 | 0.086 | 1.000 |
| OTU_2011 | 1.000 | 1.000 | **0.008** | 1.000 | 0.207 | 1.000 |  | 1.000 | 1.000 | 0.233 | 1.000 | 0.162 | 1.000 |  | 1.000 | 0.677 | **0.047** | 1.000 | 0.440 | 1.000 |
| OTU_2014 | 0.644 | **0.006** | 1.000 | 1.000 | 1.000 | **0.047** |  | 0.644 | **< 0.001** | **0.026** | **0.001** | 1.000 | 0.168 |  | 1.000 | 0.960 | 1.000 | 1.000 | 1.000 | 1.000 |
| OTU_202 | 1.000 | 1.000 | **< 0.001** | 1.000 | **< 0.001** | **0.001** |  | 1.000 | 1.000 | 0.076 | 1.000 | **< 0.001** | 0.066 |  | 1.000 | 1.000 | **0.003** | 0.052 | **< 0.001** | 1.000 |
| OTU_2020 | 0.354 | 0.227 | 0.197 | 1.000 | 1.000 | 1.000 |  | 0.354 | 1.000 | 1.000 | 1.000 | 1.000 | 1.000 |  | 1.000 | 1.000 | 1.000 | 1.000 | 1.000 | 1.000 |
| OTU_203 | **0.014** | 1.000 | **< 0.001** | **< 0.001** | **< 0.001** | **< 0.001** |  | **0.014** | 1.000 | **< 0.001** | 0.244 | **< 0.001** | **< 0.001** |  | 1.000 | 0.156 | **< 0.001** | 0.446 | **< 0.001** | **< 0.001** |
| OTU_2038 | 1.000 | 1.000 | **0.005** | 1.000 | **0.002** | 0.215 |  | 1.000 | 0.114 | **< 0.001** | 0.165 | **< 0.001** | 1.000 |  | 1.000 | 1.000 | 0.051 | 1.000 | **0.005** | **0.012** |
| OTU_204 | 1.000 | **< 0.001** | 1.000 | 0.055 | 1.000 | **0.009** |  | 1.000 | **0.027** | 1.000 | **0.002** | 1.000 | **0.036** |  | 1.000 | **0.006** | 1.000 | 0.193 | 1.000 | 0.114 |
| OTU_2044 | 1.000 | 1.000 | 1.000 | 1.000 | 1.000 | 1.000 |  | 1.000 | 0.607 | **0.004** | 1.000 | 1.000 | 1.000 |  | 0.437 | 1.000 | 0.990 | 1.000 | 1.000 | 1.000 |
| OTU_2046 | 1.000 | 1.000 | 1.000 | 1.000 | 1.000 | 1.000 |  | 1.000 | 0.676 | **0.009** | 1.000 | 0.224 | 1.000 |  | 0.714 | 0.992 | **0.043** | 1.000 | 1.000 | 1.000 |
| OTU_205 | 0.307 | **< 0.001** | **< 0.001** | 0.240 | 0.327 | 1.000 |  | 0.307 | **< 0.001** | **< 0.001** | **0.034** | 0.058 | 1.000 |  | **0.029** | **< 0.001** | **< 0.001** | **0.005** | 0.106 | 1.000 |
| OTU_2051 | 1.000 | 1.000 | **0.013** | 1.000 | 0.154 | **< 0.001** |  | 1.000 | 0.248 | 1.000 | 1.000 | 1.000 | 1.000 |  | 1.000 | 1.000 | 0.330 | 1.000 | 0.450 | **0.024** |
| OTU_2053 | **0.011** | **< 0.001** | **< 0.001** | 0.142 | **< 0.001** | 1.000 |  | **0.011** | **< 0.001** | **< 0.001** | 0.060 | **< 0.001** | 1.000 |  | 1.000 | **< 0.001** | **< 0.001** | **0.002** | **< 0.001** | 1.000 |
| OTU_206 | **< 0.001** | **< 0.001** | **< 0.001** | 1.000 | **< 0.001** | **< 0.001** |  | **< 0.001** | **< 0.001** | **< 0.001** | 1.000 | **< 0.001** | **< 0.001** |  | **< 0.001** | **< 0.001** | **< 0.001** | 1.000 | **< 0.001** | 0.315 |
| OTU_2064 | 1.000 | **0.010** | 0.473 | 0.066 | 1.000 | 1.000 |  | 1.000 | **< 0.001** | 0.071 | 0.194 | 1.000 | 1.000 |  | 1.000 | **0.039** | 0.712 | 0.467 | 1.000 | 1.000 |
| OTU_2068 | 1.000 | 1.000 | 0.115 | 1.000 | 1.000 | 1.000 |  | 1.000 | 1.000 | 1.000 | 1.000 | 1.000 | 1.000 |  | 1.000 | 1.000 | **0.003** | 1.000 | **0.005** | **0.028** |
| OTU_2069 | 0.523 | 1.000 | 1.000 | 1.000 | 0.887 | 1.000 |  | 0.523 | 1.000 | 1.000 | 1.000 | 0.959 | 1.000 |  | 1.000 | 1.000 | 1.000 | 1.000 | 1.000 | 1.000 |
| OTU_207 | 1.000 | **0.008** | **< 0.001** | 0.123 | **< 0.001** | 1.000 |  | 1.000 | **< 0.001** | **< 0.001** | **0.027** | **< 0.001** | 1.000 |  | 1.000 | **0.035** | **< 0.001** | 0.415 | **0.001** | 1.000 |
| OTU_208 | 1.000 | 0.059 | **< 0.001** | **< 0.001** | **< 0.001** | 0.745 |  | 1.000 | **< 0.001** | **< 0.001** | 0.985 | **0.005** | 1.000 |  | 1.000 | 0.260 | **< 0.001** | 0.319 | **< 0.001** | 0.861 |
| OTU_2089 | 1.000 | 1.000 | **0.009** | 1.000 | **0.008** | **0.032** |  | 1.000 | 1.000 | 0.232 | 1.000 | 0.076 | 0.196 |  | 1.000 | 1.000 | 1.000 | 1.000 | 1.000 | 1.000 |
| OTU_209 | 0.141 | **< 0.001** | **< 0.001** | **0.020** | 0.055 | 1.000 |  | 0.141 | **< 0.001** | **< 0.001** | **0.006** | 0.785 | 1.000 |  | **0.027** | **< 0.001** | **< 0.001** | **0.015** | 0.422 | 1.000 |
| OTU_2092 | 1.000 | 1.000 | 1.000 | 1.000 | 1.000 | 1.000 |  | 1.000 | 1.000 | 0.117 | 1.000 | **0.030** | 1.000 |  | 1.000 | **0.026** | 1.000 | **< 0.001** | **0.010** | 1.000 |
| OTU_21 | **0.031** | **< 0.001** | **< 0.001** | **< 0.001** | 1.000 | **0.003** |  | **0.031** | **< 0.001** | **< 0.001** | **< 0.001** | 0.235 | **< 0.001** |  | 0.169 | **< 0.001** | **< 0.001** | **< 0.001** | 1.000 | **< 0.001** |
| OTU_210 | 0.076 | 0.549 | **0.001** | 1.000 | 1.000 | 1.000 |  | 0.076 | 1.000 | **0.035** | 1.000 | 1.000 | 1.000 |  | 0.596 | 0.479 | 1.000 | 1.000 | 1.000 | 1.000 |
| OTU_211 | 1.000 | 1.000 | **0.022** | 1.000 | **0.006** | 0.079 |  | 1.000 | 0.095 | 1.000 | 1.000 | 0.271 | **< 0.001** |  | 0.097 | **< 0.001** | 1.000 | 1.000 | **< 0.001** | **< 0.001** |
| OTU_2117 | 1.000 | 1.000 | 1.000 | 1.000 | 1.000 | 1.000 |  | 1.000 | 1.000 | **0.022** | 1.000 | **0.029** | 1.000 |  | 1.000 | 1.000 | 1.000 | 1.000 | 1.000 | 1.000 |
| OTU_212 | 1.000 | 1.000 | 0.392 | 1.000 | 1.000 | 1.000 |  | 1.000 | **< 0.001** | **< 0.001** | 0.161 | **< 0.001** | 1.000 |  | 0.383 | **0.039** | 0.054 | 1.000 | 1.000 | 1.000 |
| OTU_2126 | 1.000 | **0.006** | **0.021** | 0.403 | 0.895 | 1.000 |  | 1.000 | 0.054 | 1.000 | 0.430 | 1.000 | 1.000 |  | 0.960 | **0.038** | 0.339 | 1.000 | 1.000 | 1.000 |
| OTU_213 | 1.000 | **0.002** | **< 0.001** | **< 0.001** | **< 0.001** | 1.000 |  | 1.000 | 0.352 | **0.033** | **< 0.001** | **< 0.001** | 1.000 |  | 1.000 | **0.003** | **< 0.001** | **< 0.001** | **< 0.001** | 1.000 |
| OTU_2138 | 1.000 | 0.270 | 1.000 | 0.364 | 1.000 | 1.000 |  | 1.000 | 1.000 | 1.000 | 1.000 | 0.282 | 1.000 |  | 1.000 | 1.000 | 1.000 | 1.000 | 1.000 | 1.000 |
| OTU_214 | 1.000 | 0.286 | **0.001** | 0.131 | **< 0.001** | 1.000 |  | 1.000 | **0.003** | **< 0.001** | 1.000 | 0.233 | 1.000 |  | 1.000 | 1.000 | 0.107 | 1.000 | 0.713 | 1.000 |
| OTU_215 | 1.000 | 0.526 | **< 0.001** | **0.024** | **< 0.001** | **0.004** |  | 1.000 | **< 0.001** | **< 0.001** | **< 0.001** | **< 0.001** | 1.000 |  | 1.000 | 1.000 | **< 0.001** | 1.000 | **< 0.001** | **< 0.001** |
| OTU_2157 | 1.000 | 1.000 | 1.000 | 0.059 | 0.070 | 1.000 |  | 1.000 | 0.054 | **0.010** | 1.000 | 1.000 | 1.000 |  | 1.000 | **< 0.001** | 0.119 | 0.065 | 1.000 | 1.000 |
| OTU_216 | **0.049** | **< 0.001** | 0.192 | 0.203 | 1.000 | **0.027** |  | **0.049** | **< 0.001** | 0.920 | 1.000 | 1.000 | **0.036** |  | 0.141 | **< 0.001** | 1.000 | 1.000 | 0.268 | **0.004** |
| OTU_2167 | 0.471 | 1.000 | 1.000 | 0.786 | 0.338 | 1.000 |  | 0.471 | **0.005** | **0.009** | **0.030** | **0.043** | 1.000 |  | 0.326 | 1.000 | 1.000 | 1.000 | 1.000 | 1.000 |
| OTU_2169 | 1.000 | **0.003** | 1.000 | **< 0.001** | 1.000 | **0.006** |  | 1.000 | 0.084 | 1.000 | 0.126 | 1.000 | 1.000 |  | 1.000 | **0.010** | 1.000 | **< 0.001** | 0.276 | 1.000 |
| OTU_217 | 1.000 | **< 0.001** | **0.001** | **< 0.001** | **0.003** | **< 0.001** |  | 1.000 | **< 0.001** | **< 0.001** | **< 0.001** | 1.000 | 0.289 |  | **0.023** | **< 0.001** | **< 0.001** | **< 0.001** | 1.000 | 0.064 |
| OTU_218 | 1.000 | 0.302 | 1.000 | 1.000 | 1.000 | 1.000 |  | 1.000 | **0.019** | 1.000 | 1.000 | **0.018** | 1.000 |  | 1.000 | 1.000 | 1.000 | 1.000 | 1.000 | 1.000 |
| OTU_2183 | 1.000 | 0.659 | 1.000 | 1.000 | 1.000 | 1.000 |  | 1.000 | 1.000 | 1.000 | 1.000 | 1.000 | 1.000 |  | 1.000 | 0.107 | 0.129 | 1.000 | 1.000 | 1.000 |
| OTU_2184 | 1.000 | 1.000 | 1.000 | 1.000 | **0.008** | 0.484 |  | 1.000 | **0.023** | 0.387 | 1.000 | 0.313 | 1.000 |  | 1.000 | 1.000 | 1.000 | 1.000 | 0.131 | 1.000 |
| OTU_219 | 1.000 | 0.379 | **< 0.001** | **0.002** | **< 0.001** | **< 0.001** |  | 1.000 | 0.118 | **< 0.001** | **0.025** | **< 0.001** | **< 0.001** |  | 1.000 | 1.000 | **< 0.001** | 0.297 | **< 0.001** | **0.039** |
| OTU_22 | 1.000 | **< 0.001** | **< 0.001** | **< 0.001** | 0.052 | **< 0.001** |  | 1.000 | **< 0.001** | **< 0.001** | **< 0.001** | **< 0.001** | **< 0.001** |  | 1.000 | **< 0.001** | **< 0.001** | **< 0.001** | **< 0.001** | 0.288 |
| OTU_220 | 1.000 | **0.003** | 1.000 | **< 0.001** | 1.000 | 0.129 |  | 1.000 | **0.024** | 1.000 | **0.003** | 1.000 | **0.005** |  | 1.000 | **< 0.001** | 1.000 | **< 0.001** | 1.000 | **0.007** |
| OTU_2201 | 1.000 | 1.000 | 1.000 | 1.000 | 0.501 | 1.000 |  | 1.000 | 1.000 | 0.815 | 1.000 | 1.000 | 1.000 |  | 1.000 | 1.000 | 0.440 | 1.000 | 0.935 | 1.000 |
| OTU_221 | **< 0.001** | **< 0.001** | **< 0.001** | 1.000 | 1.000 | 1.000 |  | **< 0.001** | **< 0.001** | **< 0.001** | 1.000 | 1.000 | 1.000 |  | **0.034** | **< 0.001** | **< 0.001** | 1.000 | 1.000 | 1.000 |
| OTU_2211 | 1.000 | 1.000 | 1.000 | 1.000 | 1.000 | 1.000 |  | 1.000 | 0.879 | 0.491 | 1.000 | 1.000 | 1.000 |  | 1.000 | 1.000 | 1.000 | 1.000 | 1.000 | 1.000 |
| OTU_2219 | 1.000 | 1.000 | 1.000 | 1.000 | 1.000 | 1.000 |  | 1.000 | 1.000 | 1.000 | 1.000 | 1.000 | 1.000 |  | 1.000 | 1.000 | 1.000 | 1.000 | 1.000 | 0.969 |
| OTU_222 | 1.000 | **0.004** | 1.000 | 0.054 | 1.000 | **< 0.001** |  | 1.000 | **0.033** | 1.000 | 0.151 | 1.000 | 0.144 |  | 1.000 | 1.000 | 1.000 | 1.000 | 1.000 | 1.000 |
| OTU_2221 | **0.027** | 0.637 | 1.000 | 1.000 | 0.051 | 1.000 |  | **0.027** | 1.000 | **0.036** | 1.000 | 1.000 | 1.000 |  | 1.000 | 1.000 | 1.000 | 1.000 | 1.000 | 1.000 |
| OTU_223 | 1.000 | 1.000 | 1.000 | 1.000 | 1.000 | 1.000 |  | 1.000 | 0.070 | 0.077 | 1.000 | 1.000 | 1.000 |  | 1.000 | 1.000 | 1.000 | 1.000 | 1.000 | 1.000 |
| OTU_2238 | 1.000 | 1.000 | 1.000 | 1.000 | 1.000 | 1.000 |  | 1.000 | 1.000 | 0.901 | 1.000 | 1.000 | 1.000 |  | 1.000 | 1.000 | 0.708 | 1.000 | 0.241 | 1.000 |
| OTU_224 | 1.000 | 0.566 | 1.000 | 1.000 | 1.000 | 1.000 |  | 1.000 | 1.000 | 1.000 | 1.000 | 1.000 | 1.000 |  | 1.000 | 0.053 | 0.063 | 1.000 | 1.000 | 1.000 |
| OTU_2243 | 1.000 | 1.000 | 0.300 | 1.000 | 0.860 | 1.000 |  | 1.000 | **< 0.001** | **< 0.001** | **< 0.001** | **0.007** | 1.000 |  | **0.009** | 0.053 | **0.001** | 1.000 | 1.000 | 1.000 |
| OTU_225 | 1.000 | **0.004** | 0.185 | 1.000 | 1.000 | 1.000 |  | 1.000 | **< 0.001** | **< 0.001** | 0.376 | 0.969 | 1.000 |  | 1.000 | **0.027** | 0.652 | 1.000 | 1.000 | 1.000 |
| OTU_2254 | 1.000 | 0.404 | 0.321 | 1.000 | 1.000 | 1.000 |  | 1.000 | 0.221 | 1.000 | 0.432 | 1.000 | 0.170 |  | 0.095 | 1.000 | 1.000 | 1.000 | 0.931 | 1.000 |
| OTU_226 | 1.000 | **< 0.001** | 1.000 | **< 0.001** | 1.000 | **< 0.001** |  | 1.000 | **0.001** | 1.000 | 0.774 | 1.000 | **0.002** |  | **0.003** | **< 0.001** | 0.440 | 0.079 | 1.000 | **< 0.001** |
| OTU_227 | 1.000 | 1.000 | 0.251 | 1.000 | **< 0.001** | 0.057 |  | 1.000 | 1.000 | **< 0.001** | 1.000 | **< 0.001** | **0.002** |  | 1.000 | 1.000 | 1.000 | 1.000 | 0.117 | 1.000 |
| OTU_2276 | 1.000 | 1.000 | 1.000 | 1.000 | 1.000 | 1.000 |  | 1.000 | 1.000 | 1.000 | 1.000 | 1.000 | 1.000 |  | 1.000 | 0.332 | 1.000 | 1.000 | 1.000 | 1.000 |
| OTU_228 | 0.066 | 1.000 | 1.000 | **0.005** | 1.000 | 0.469 |  | 0.066 | 1.000 | 1.000 | 1.000 | 1.000 | 1.000 |  | 1.000 | 1.000 | 1.000 | **0.040** | 0.836 | 1.000 |
| OTU_229 | 0.403 | **< 0.001** | **< 0.001** | **< 0.001** | **< 0.001** | 1.000 |  | 0.403 | **< 0.001** | **< 0.001** | **0.028** | **0.023** | 1.000 |  | 0.596 | **< 0.001** | **< 0.001** | **< 0.001** | **< 0.001** | 1.000 |
| OTU_2291 | 0.428 | **0.016** | 1.000 | 1.000 | 0.313 | **0.040** |  | 0.428 | **0.014** | 1.000 | 1.000 | 0.289 | 0.197 |  | 1.000 | **0.012** | 1.000 | 1.000 | 1.000 | 0.051 |
| OTU_23 | 1.000 | **0.003** | 1.000 | **0.014** | 1.000 | **0.003** |  | 1.000 | **< 0.001** | 0.122 | **0.006** | 1.000 | **0.015** |  | 1.000 | **< 0.001** | 1.000 | **< 0.001** | 1.000 | **< 0.001** |
| OTU_230 | 0.382 | **0.031** | 1.000 | 1.000 | 0.201 | 0.052 |  | 0.382 | 0.364 | 1.000 | 0.539 | 1.000 | **0.016** |  | 1.000 | **0.002** | 1.000 | 0.345 | 1.000 | **0.002** |
| OTU_231 | 1.000 | **0.007** | **< 0.001** | 0.122 | **< 0.001** | **0.019** |  | 1.000 | **0.005** | **< 0.001** | **0.002** | **< 0.001** | **0.004** |  | 1.000 | **0.044** | **< 0.001** | 0.504 | **< 0.001** | **< 0.001** |
| OTU_232 | 1.000 | 1.000 | 1.000 | 0.278 | 1.000 | 0.215 |  | 1.000 | 0.346 | 1.000 | **< 0.001** | 1.000 | **0.014** |  | 1.000 | 0.083 | 1.000 | 0.134 | 1.000 | 0.149 |
| OTU_2323 | 1.000 | 1.000 | 1.000 | 1.000 | 1.000 | 1.000 |  | 1.000 | 1.000 | 1.000 | 0.297 | 1.000 | 0.891 |  | 1.000 | 1.000 | 1.000 | 1.000 | 1.000 | 1.000 |
| OTU_233 | 1.000 | 1.000 | **0.027** | 1.000 | 0.442 | 0.289 |  | 1.000 | 1.000 | **0.028** | 1.000 | 0.114 | 0.284 |  | 1.000 | 1.000 | **< 0.001** | 1.000 | **< 0.001** | 0.084 |
| OTU_234 | 1.000 | 1.000 | **0.006** | 1.000 | **0.043** | 1.000 |  | 1.000 | **< 0.001** | **< 0.001** | 0.199 | 1.000 | 1.000 |  | 0.951 | **< 0.001** | 0.071 | 0.774 | 1.000 | 1.000 |
| OTU_235 | 1.000 | 1.000 | **< 0.001** | 1.000 | **< 0.001** | **< 0.001** |  | 1.000 | **0.010** | **< 0.001** | 1.000 | **< 0.001** | **< 0.001** |  | 1.000 | 0.053 | **< 0.001** | **0.002** | **< 0.001** | 1.000 |
| OTU_236 | 1.000 | **< 0.001** | **< 0.001** | **0.003** | 0.132 | 1.000 |  | 1.000 | **< 0.001** | **< 0.001** | **< 0.001** | **0.011** | 1.000 |  | 1.000 | **< 0.001** | **< 0.001** | **< 0.001** | 0.510 | 1.000 |
| OTU_2364 | **0.021** | **< 0.001** | **< 0.001** | **< 0.001** | **0.004** | 1.000 |  | **0.021** | **< 0.001** | **< 0.001** | **< 0.001** | **< 0.001** | 0.574 |  | **0.010** | **< 0.001** | **< 0.001** | **< 0.001** | **< 0.001** | 1.000 |
| OTU_237 | **< 0.001** | **< 0.001** | **< 0.001** | 1.000 | 1.000 | 1.000 |  | **< 0.001** | **< 0.001** | **< 0.001** | 1.000 | 1.000 | 1.000 |  | 0.275 | **< 0.001** | 0.239 | 1.000 | 1.000 | 0.991 |
| OTU_2376 | 0.203 | 1.000 | 0.648 | 1.000 | 1.000 | 1.000 |  | 0.203 | 1.000 | 1.000 | 1.000 | 1.000 | 1.000 |  | 1.000 | 1.000 | 1.000 | 1.000 | 1.000 | 1.000 |
| OTU_238 | **< 0.001** | **< 0.001** | **0.008** | 1.000 | 0.945 | **0.005** |  | **< 0.001** | **< 0.001** | 1.000 | 0.949 | 1.000 | **0.002** |  | 1.000 | 1.000 | 1.000 | 1.000 | 1.000 | 0.247 |
| OTU_2381 | 0.371 | 1.000 | 1.000 | 0.124 | **0.001** | 1.000 |  | 0.371 | **< 0.001** | **< 0.001** | 0.528 | **0.011** | 1.000 |  | 1.000 | 1.000 | 1.000 | 0.759 | 1.000 | 1.000 |
| OTU_2386 | 1.000 | 1.000 | 0.223 | 1.000 | 0.952 | 1.000 |  | 1.000 | **< 0.001** | **< 0.001** | 1.000 | 1.000 | 1.000 |  | 1.000 | 1.000 | 1.000 | 1.000 | 1.000 | 1.000 |
| OTU_239 | **< 0.001** | **< 0.001** | **< 0.001** | 1.000 | 1.000 | 1.000 |  | **< 0.001** | **< 0.001** | **< 0.001** | 1.000 | 1.000 | 1.000 |  | 0.421 | **0.009** | **0.050** | 1.000 | 1.000 | 1.000 |
| OTU_2396 | 1.000 | 0.076 | 1.000 | 1.000 | 1.000 | **0.036** |  | 1.000 | 0.243 | 1.000 | 1.000 | 1.000 | 1.000 |  | 1.000 | 1.000 | 1.000 | 1.000 | 1.000 | 0.346 |
| OTU_24 | **< 0.001** | **< 0.001** | **< 0.001** | 0.055 | 1.000 | 1.000 |  | **< 0.001** | **< 0.001** | **< 0.001** | 0.094 | **0.011** | 1.000 |  | **< 0.001** | **< 0.001** | **< 0.001** | 0.157 | 1.000 | 1.000 |
| OTU_240 | 1.000 | 0.220 | 0.822 | 0.070 | 0.249 | 1.000 |  | 1.000 | 0.191 | 1.000 | **0.010** | 0.096 | 1.000 |  | 1.000 | **0.048** | 0.500 | **0.033** | 0.301 | 1.000 |
| OTU_2401 | 1.000 | **< 0.001** | **0.003** | **< 0.001** | **< 0.001** | **< 0.001** |  | 1.000 | **< 0.001** | **< 0.001** | **< 0.001** | **0.004** | **< 0.001** |  | 1.000 | **< 0.001** | 1.000 | **< 0.001** | 0.406 | **< 0.001** |
| OTU_241 | **< 0.001** | **< 0.001** | **< 0.001** | 1.000 | 1.000 | 1.000 |  | **< 0.001** | **< 0.001** | **< 0.001** | **0.020** | 1.000 | 1.000 |  | **0.032** | **< 0.001** | **< 0.001** | 0.621 | 1.000 | 1.000 |
| OTU_242 | 1.000 | 0.368 | 0.618 | 1.000 | 0.325 | **< 0.001** |  | 1.000 | 1.000 | 0.471 | 1.000 | 1.000 | 0.052 |  | 1.000 | 1.000 | 0.922 | 0.379 | 1.000 | 0.134 |
| OTU_2423 | 1.000 | **0.001** | **0.001** | 0.604 | 0.404 | 1.000 |  | 1.000 | 1.000 | 1.000 | 1.000 | 1.000 | 1.000 |  | 1.000 | 0.095 | **< 0.001** | 0.401 | **< 0.001** | 0.390 |
| OTU_2428 | 1.000 | 1.000 | 1.000 | 1.000 | 1.000 | 1.000 |  | 1.000 | 0.277 | 0.290 | 0.390 | 0.341 | 1.000 |  | 1.000 | 1.000 | 1.000 | 1.000 | 1.000 | 1.000 |
| OTU_243 | **0.001** | **0.005** | **< 0.001** | 1.000 | 1.000 | 0.948 |  | **0.001** | **0.008** | **< 0.001** | 1.000 | 1.000 | 1.000 |  | 1.000 | 1.000 | 1.000 | 1.000 | 1.000 | 1.000 |
| OTU_2433 | 1.000 | **< 0.001** | 0.200 | **< 0.001** | 0.492 | 0.155 |  | 1.000 | **< 0.001** | **< 0.001** | **< 0.001** | 0.363 | 0.073 |  | 1.000 | **< 0.001** | 1.000 | **0.012** | 1.000 | 0.066 |
| OTU_2438 | 1.000 | 1.000 | 1.000 | 1.000 | 1.000 | 1.000 |  | 1.000 | 1.000 | 1.000 | 1.000 | 1.000 | 1.000 |  | 0.055 | **0.003** | 1.000 | 1.000 | 0.576 | 0.149 |
| OTU_244 | **0.002** | **< 0.001** | **< 0.001** | 1.000 | **< 0.001** | **< 0.001** |  | **0.002** | **< 0.001** | **< 0.001** | 1.000 | **< 0.001** | **0.005** |  | 0.059 | **0.004** | **< 0.001** | 1.000 | 1.000 | 1.000 |
| OTU_2446 | **0.001** | **< 0.001** | **< 0.001** | 1.000 | 1.000 | 1.000 |  | **0.001** | **0.002** | **< 0.001** | 0.954 | **0.017** | 1.000 |  | 0.177 | 0.575 | **< 0.001** | 1.000 | 1.000 | 0.636 |
| OTU_245 | 1.000 | **< 0.001** | **< 0.001** | 0.404 | 0.225 | 1.000 |  | 1.000 | 0.143 | 0.330 | **< 0.001** | **< 0.001** | 1.000 |  | 1.000 | **< 0.001** | **0.009** | 0.292 | 1.000 | 1.000 |
| OTU_246 | 1.000 | 1.000 | 1.000 | 1.000 | 1.000 | 1.000 |  | 1.000 | 1.000 | 1.000 | 1.000 | 1.000 | 1.000 |  | 1.000 | 0.418 | **0.026** | 1.000 | 1.000 | 1.000 |
| OTU_2460 | 1.000 | 0.354 | **0.016** | 1.000 | 1.000 | 1.000 |  | 1.000 | 0.181 | **0.022** | 1.000 | 1.000 | 1.000 |  | 1.000 | 1.000 | 1.000 | 1.000 | 1.000 | 1.000 |
| OTU_247 | 1.000 | 1.000 | 1.000 | 0.292 | 1.000 | 1.000 |  | 1.000 | 1.000 | **0.037** | 0.709 | 1.000 | 1.000 |  | 1.000 | 1.000 | 1.000 | 0.675 | 1.000 | 0.432 |
| OTU_2470 | 1.000 | 1.000 | 1.000 | 1.000 | 0.244 | 0.465 |  | 1.000 | 1.000 | 1.000 | 1.000 | 1.000 | 1.000 |  | 0.063 | **0.028** | 1.000 | 1.000 | 0.206 | 0.370 |
| OTU_248 | 1.000 | 1.000 | 1.000 | 1.000 | 1.000 | 1.000 |  | 1.000 | **< 0.001** | **< 0.001** | 1.000 | 1.000 | 1.000 |  | **< 0.001** | **< 0.001** | **< 0.001** | 1.000 | 1.000 | 1.000 |
| OTU_249 | 1.000 | 0.750 | 1.000 | 1.000 | 1.000 | 0.572 |  | 1.000 | 1.000 | 1.000 | 1.000 | 1.000 | 0.291 |  | 1.000 | **0.045** | 1.000 | 1.000 | 1.000 | 1.000 |
| OTU_25 | 1.000 | 0.109 | 0.074 | 0.437 | **0.032** | **< 0.001** |  | 1.000 | 0.169 | 0.581 | 0.476 | 0.419 | **< 0.001** |  | 1.000 | 0.091 | 1.000 | **0.035** | 1.000 | **0.009** |
| OTU_2501 | 0.735 | 1.000 | 1.000 | 1.000 | 1.000 | 1.000 |  | 0.735 | 0.475 | **0.039** | 1.000 | 1.000 | 1.000 |  | 1.000 | 1.000 | 1.000 | 1.000 | 1.000 | 1.000 |
| OTU_2503 | 1.000 | 1.000 | 1.000 | 1.000 | 1.000 | 1.000 |  | 1.000 | 1.000 | 0.768 | 1.000 | 1.000 | 1.000 |  | 0.062 | **< 0.001** | **0.022** | **< 0.001** | 1.000 | **< 0.001** |
| OTU_2504 | 0.689 | 1.000 | 1.000 | 1.000 | 0.133 | 1.000 |  | 0.689 | 1.000 | 1.000 | 0.641 | 0.282 | 1.000 |  | 1.000 | 1.000 | 1.000 | 1.000 | 1.000 | 1.000 |
| OTU_2507 | 0.123 | **0.012** | **< 0.001** | 1.000 | 1.000 | 1.000 |  | 0.123 | 0.296 | 0.805 | 0.273 | 0.575 | 1.000 |  | 0.960 | **0.040** | 0.801 | 1.000 | 1.000 | 1.000 |
| OTU_2509 | **0.013** | 0.234 | 0.346 | 1.000 | 1.000 | 1.000 |  | **0.013** | 1.000 | 1.000 | 1.000 | 1.000 | 1.000 |  | 1.000 | 1.000 | 1.000 | 1.000 | 1.000 | 1.000 |
| OTU_251 | 1.000 | **< 0.001** | **< 0.001** | **< 0.001** | **0.012** | 0.406 |  | 1.000 | **< 0.001** | **< 0.001** | **< 0.001** | 0.448 | 1.000 |  | 1.000 | **< 0.001** | **< 0.001** | **< 0.001** | 0.071 | 1.000 |
| OTU_252 | 1.000 | 1.000 | **0.011** | 1.000 | **0.023** | 0.282 |  | 1.000 | 1.000 | 1.000 | 1.000 | 1.000 | 1.000 |  | 1.000 | 1.000 | 1.000 | 1.000 | 1.000 | 1.000 |
| OTU_253 | 1.000 | 0.472 | 1.000 | 0.830 | 1.000 | 1.000 |  | 1.000 | 1.000 | 1.000 | 1.000 | 1.000 | 1.000 |  | 1.000 | 0.364 | 1.000 | 1.000 | 1.000 | 1.000 |
| OTU_2532 | 1.000 | 0.076 | 1.000 | **0.004** | 1.000 | 0.504 |  | 1.000 | **0.012** | 0.205 | 0.165 | 1.000 | 1.000 |  | 0.755 | 0.430 | 0.571 | 1.000 | 1.000 | 1.000 |
| OTU_254 | 0.327 | 1.000 | **< 0.001** | 0.826 | 1.000 | **0.004** |  | 0.327 | 1.000 | 1.000 | 0.058 | 1.000 | 0.287 |  | 1.000 | 1.000 | 0.123 | 1.000 | 1.000 | 1.000 |
| OTU_2540 | **0.022** | **< 0.001** | 0.176 | 0.609 | 1.000 | **0.045** |  | **0.022** | **0.019** | 1.000 | 1.000 | 0.829 | **0.021** |  | 1.000 | 0.166 | 1.000 | 1.000 | 1.000 | 0.517 |
| OTU_255 | **< 0.001** | **< 0.001** | **0.025** | **0.009** | 0.081 | **< 0.001** |  | **< 0.001** | **< 0.001** | 0.374 | 0.985 | 0.089 | **< 0.001** |  | **0.019** | **< 0.001** | 1.000 | **0.024** | 0.191 | **< 0.001** |
| OTU_2557 | 1.000 | 0.422 | 1.000 | 0.975 | 1.000 | 1.000 |  | 1.000 | 1.000 | 0.280 | 1.000 | 1.000 | 1.000 |  | 1.000 | 1.000 | 0.131 | 1.000 | 0.174 | 1.000 |
| OTU_256 | 0.756 | **< 0.001** | 1.000 | 1.000 | 0.792 | **0.002** |  | 0.756 | **< 0.001** | 0.454 | 1.000 | 1.000 | 0.629 |  | 1.000 | 1.000 | 0.586 | 1.000 | 0.073 | **0.024** |
| OTU_2560 | 1.000 | 1.000 | 1.000 | 1.000 | 1.000 | 1.000 |  | 1.000 | 1.000 | 1.000 | 0.687 | 1.000 | 1.000 |  | 1.000 | 1.000 | 1.000 | 1.000 | 1.000 | 1.000 |
| OTU_2568 | 1.000 | **< 0.001** | 0.290 | **< 0.001** | 1.000 | 0.201 |  | 1.000 | **< 0.001** | 1.000 | 0.091 | 1.000 | **0.011** |  | 1.000 | **< 0.001** | 0.670 | **< 0.001** | 1.000 | **0.037** |
| OTU_2569 | 0.510 | **0.002** | **0.046** | 1.000 | 1.000 | 1.000 |  | 0.510 | 1.000 | 1.000 | 1.000 | 1.000 | 1.000 |  | **0.010** | **0.027** | 0.258 | 1.000 | 1.000 | 1.000 |
| OTU_257 | 1.000 | 1.000 | **< 0.001** | 1.000 | **< 0.001** | **< 0.001** |  | 1.000 | **0.033** | **< 0.001** | 0.093 | **< 0.001** | **< 0.001** |  | 1.000 | 1.000 | **< 0.001** | 1.000 | **< 0.001** | **0.037** |
| OTU_2574 | 1.000 | 0.153 | 0.438 | 0.059 | 0.143 | 1.000 |  | 1.000 | **0.018** | **0.001** | 0.362 | **0.041** | 1.000 |  | 1.000 | **0.048** | 0.561 | 0.587 | 1.000 | 1.000 |
| OTU_2579 | 1.000 | 1.000 | 1.000 | 0.416 | 1.000 | 1.000 |  | 1.000 | 1.000 | 1.000 | 1.000 | 1.000 | 1.000 |  | 1.000 | 1.000 | 1.000 | 1.000 | 1.000 | 1.000 |
| OTU_258 | 1.000 | 1.000 | 0.264 | 1.000 | 0.083 | 1.000 |  | 1.000 | 1.000 | 1.000 | 1.000 | 0.343 | 1.000 |  | 1.000 | 1.000 | **0.002** | 0.538 | **< 0.001** | 0.521 |
| OTU_2583 | 1.000 | 1.000 | 1.000 | 1.000 | 0.237 | 0.848 |  | 1.000 | 1.000 | 1.000 | 1.000 | 0.122 | 1.000 |  | 1.000 | 1.000 | 1.000 | 1.000 | 1.000 | 1.000 |
| OTU_2584 | **0.002** | **< 0.001** | **< 0.001** | 1.000 | 1.000 | 1.000 |  | **0.002** | **< 0.001** | 0.134 | 0.358 | 1.000 | 0.370 |  | 1.000 | **0.005** | 0.402 | 0.826 | 1.000 | 1.000 |
| OTU_2589 | 1.000 | 0.636 | 1.000 | 1.000 | 1.000 | 0.975 |  | 1.000 | 0.166 | 1.000 | 0.428 | 1.000 | 0.205 |  | 1.000 | 1.000 | 1.000 | 1.000 | 1.000 | 0.675 |
| OTU_259 | 1.000 | 1.000 | 0.381 | 1.000 | **0.005** | 1.000 |  | 1.000 | 0.080 | **< 0.001** | 0.406 | **< 0.001** | 1.000 |  | 1.000 | 1.000 | 1.000 | 1.000 | **0.033** | 1.000 |
| OTU_2597 | 1.000 | 1.000 | 0.290 | 1.000 | 1.000 | 1.000 |  | 1.000 | 1.000 | 1.000 | 1.000 | 1.000 | 1.000 |  | 1.000 | 1.000 | 1.000 | 1.000 | 1.000 | 1.000 |
| OTU_26 | **0.039** | **< 0.001** | **< 0.001** | **< 0.001** | **< 0.001** | 0.230 |  | **0.039** | **< 0.001** | **< 0.001** | **0.007** | **0.007** | 1.000 |  | **< 0.001** | **< 0.001** | **< 0.001** | **0.041** | **< 0.001** | 1.000 |
| OTU_260 | 1.000 | 0.687 | **< 0.001** | 1.000 | **< 0.001** | **< 0.001** |  | 1.000 | **< 0.001** | **< 0.001** | 1.000 | **< 0.001** | **< 0.001** |  | 0.982 | 1.000 | **< 0.001** | 1.000 | **< 0.001** | **< 0.001** |
| OTU_261 | 1.000 | **< 0.001** | **< 0.001** | **0.013** | **< 0.001** | 1.000 |  | 1.000 | **< 0.001** | **< 0.001** | **0.002** | **< 0.001** | 1.000 |  | 0.685 | **0.008** | **< 0.001** | 1.000 | **0.034** | 1.000 |
| OTU_262 | **0.001** | **< 0.001** | **< 0.001** | 1.000 | 1.000 | 1.000 |  | **0.001** | **< 0.001** | **< 0.001** | 0.430 | 0.914 | 1.000 |  | **0.006** | **< 0.001** | **< 0.001** | **0.012** | 0.427 | 1.000 |
| OTU_263 | **< 0.001** | **< 0.001** | **< 0.001** | 1.000 | 1.000 | 1.000 |  | **< 0.001** | **< 0.001** | **< 0.001** | 1.000 | 1.000 | 1.000 |  | **< 0.001** | **0.009** | **0.003** | 1.000 | 1.000 | 1.000 |
| OTU_2632 | 1.000 | 1.000 | 1.000 | **0.009** | **0.016** | 1.000 |  | 1.000 | 1.000 | 1.000 | 1.000 | 1.000 | 1.000 |  | 1.000 | 1.000 | 1.000 | 1.000 | 1.000 | 1.000 |
| OTU_264 | 1.000 | 1.000 | 1.000 | 1.000 | 0.859 | 1.000 |  | 1.000 | 1.000 | 1.000 | 0.913 | 0.579 | 1.000 |  | 0.361 | 1.000 | 1.000 | 1.000 | 0.078 | 1.000 |
| OTU_2641 | 1.000 | 1.000 | **< 0.001** | 1.000 | 0.142 | 0.055 |  | 1.000 | 1.000 | **< 0.001** | 1.000 | **0.007** | **0.032** |  | 1.000 | 0.094 | **< 0.001** | 1.000 | **0.018** | 0.736 |
| OTU_2642 | 1.000 | **< 0.001** | **< 0.001** | **0.036** | 0.130 | 1.000 |  | 1.000 | **< 0.001** | **< 0.001** | **0.002** | **0.012** | 1.000 |  | 1.000 | **< 0.001** | **0.001** | 0.052 | 0.267 | 1.000 |
| OTU_2647 | 0.666 | 1.000 | 1.000 | 1.000 | 1.000 | 1.000 |  | 0.666 | 1.000 | 1.000 | 1.000 | 1.000 | 1.000 |  | 1.000 | 1.000 | 1.000 | 1.000 | 1.000 | 1.000 |
| OTU_2649 | 1.000 | 1.000 | 1.000 | 1.000 | 1.000 | 1.000 |  | 1.000 | 1.000 | 1.000 | 1.000 | 1.000 | 1.000 |  | 1.000 | 1.000 | 1.000 | 1.000 | 1.000 | 1.000 |
| OTU_265 | 1.000 | **0.008** | 1.000 | 1.000 | 1.000 | 0.062 |  | 1.000 | 0.314 | 1.000 | 0.089 | 1.000 | 0.069 |  | 1.000 | 1.000 | 1.000 | 1.000 | 1.000 | 1.000 |
| OTU_2657 | 1.000 | 1.000 | 1.000 | 1.000 | 1.000 | 1.000 |  | 1.000 | 1.000 | 1.000 | 1.000 | 1.000 | 1.000 |  | 1.000 | 1.000 | 1.000 | 1.000 | 1.000 | 1.000 |
| OTU_266 | **< 0.001** | **< 0.001** | **0.006** | 1.000 | 1.000 | 1.000 |  | **< 0.001** | **0.002** | 0.459 | 1.000 | 1.000 | 1.000 |  | 0.619 | 0.121 | 1.000 | 1.000 | 1.000 | 1.000 |
| OTU_2668 | 1.000 | 0.063 | 1.000 | 1.000 | 1.000 | **0.037** |  | 1.000 | 0.562 | 1.000 | 1.000 | 1.000 | 0.385 |  | 1.000 | **< 0.001** | 0.161 | **0.006** | 1.000 | 1.000 |
| OTU_267 | 1.000 | **< 0.001** | **0.002** | **< 0.001** | **0.012** | 0.250 |  | 1.000 | **< 0.001** | **< 0.001** | **< 0.001** | **< 0.001** | **0.038** |  | 1.000 | **< 0.001** | **0.002** | **< 0.001** | 1.000 | 0.076 |
| OTU_2671 | 1.000 | 1.000 | 1.000 | **0.042** | 1.000 | 1.000 |  | 1.000 | **0.022** | **0.012** | 1.000 | 1.000 | 1.000 |  | 1.000 | 1.000 | 1.000 | 1.000 | 1.000 | 1.000 |
| OTU_2675 | 1.000 | 1.000 | 1.000 | 1.000 | 1.000 | 1.000 |  | 1.000 | 1.000 | 1.000 | 1.000 | 1.000 | 1.000 |  | 1.000 | 1.000 | 1.000 | 0.788 | 1.000 | 0.826 |
| OTU_268 | 1.000 | 0.110 | **< 0.001** | 0.708 | **< 0.001** | 1.000 |  | 1.000 | 1.000 | **0.009** | 0.077 | **< 0.001** | 1.000 |  | 1.000 | 1.000 | **0.003** | 1.000 | **0.002** | 1.000 |
| OTU_269 | 1.000 | 0.114 | 0.974 | 1.000 | 1.000 | 1.000 |  | 1.000 | 0.863 | 1.000 | 1.000 | 1.000 | 1.000 |  | 1.000 | 1.000 | 1.000 | 1.000 | 1.000 | 1.000 |
| OTU_2694 | 1.000 | 1.000 | 1.000 | 1.000 | 1.000 | 1.000 |  | 1.000 | 0.229 | 0.078 | 1.000 | 1.000 | 1.000 |  | 1.000 | 1.000 | 1.000 | 1.000 | 1.000 | 1.000 |
| OTU_27 | 1.000 | 1.000 | **< 0.001** | 1.000 | **< 0.001** | **< 0.001** |  | 1.000 | 0.102 | **< 0.001** | 1.000 | **< 0.001** | **< 0.001** |  | 0.575 | **0.041** | **< 0.001** | 1.000 | **< 0.001** | **< 0.001** |
| OTU_2708 | 1.000 | 1.000 | 1.000 | 1.000 | 1.000 | 1.000 |  | 1.000 | 1.000 | 1.000 | 1.000 | 0.313 | 1.000 |  | 1.000 | 1.000 | 1.000 | 1.000 | 1.000 | 1.000 |
| OTU_2709 | 1.000 | 1.000 | **0.003** | 1.000 | 0.475 | 0.700 |  | 1.000 | 1.000 | 1.000 | 1.000 | 1.000 | 1.000 |  | 0.411 | 1.000 | 1.000 | 1.000 | 0.074 | 1.000 |
| OTU_271 | 1.000 | 0.100 | **0.012** | 1.000 | 0.640 | 1.000 |  | 1.000 | 1.000 | 1.000 | 1.000 | 1.000 | 1.000 |  | 1.000 | 0.471 | 0.122 | 1.000 | 1.000 | 1.000 |
| OTU_2711 | 1.000 | 1.000 | 1.000 | 1.000 | 1.000 | 0.970 |  | 1.000 | 1.000 | 1.000 | 1.000 | 1.000 | 1.000 |  | 1.000 | 1.000 | 1.000 | 1.000 | 1.000 | 1.000 |
| OTU_272 | 1.000 | **< 0.001** | **< 0.001** | **< 0.001** | **< 0.001** | 0.816 |  | 1.000 | **< 0.001** | **< 0.001** | **0.011** | **0.037** | 1.000 |  | 0.960 | **< 0.001** | **< 0.001** | **< 0.001** | **0.017** | 0.942 |
| OTU_273 | 1.000 | **0.001** | **< 0.001** | **< 0.001** | **< 0.001** | 1.000 |  | 1.000 | **0.004** | **< 0.001** | 1.000 | 0.504 | 1.000 |  | 0.437 | **< 0.001** | **< 0.001** | 0.172 | **0.038** | 1.000 |
| OTU_2735 | 1.000 | 1.000 | 0.445 | 1.000 | 0.327 | 1.000 |  | 1.000 | 0.598 | 0.386 | 1.000 | 1.000 | 1.000 |  | 1.000 | 1.000 | 1.000 | 1.000 | 1.000 | 1.000 |
| OTU_2737 | 1.000 | **< 0.001** | **0.006** | **0.049** | 0.187 | 1.000 |  | 1.000 | **< 0.001** | **0.007** | **0.009** | 0.079 | 1.000 |  | 1.000 | **0.037** | **0.022** | 0.727 | 0.404 | 1.000 |
| OTU_274 | **0.027** | **< 0.001** | **< 0.001** | **0.024** | 0.255 | 1.000 |  | **0.027** | **< 0.001** | **0.003** | **0.011** | **0.029** | 1.000 |  | 0.430 | **0.001** | **< 0.001** | 1.000 | **0.004** | 0.736 |
| OTU_275 | 1.000 | 0.343 | **< 0.001** | 0.830 | **< 0.001** | 0.295 |  | 1.000 | **0.006** | **< 0.001** | 1.000 | 1.000 | 1.000 |  | 1.000 | **0.002** | **< 0.001** | 1.000 | 0.167 | 1.000 |
| OTU_2761 | **0.005** | 1.000 | 1.000 | 0.172 | **0.003** | 1.000 |  | **0.005** | 1.000 | 1.000 | 0.178 | **0.033** | 1.000 |  | 1.000 | 1.000 | 1.000 | 1.000 | 1.000 | 1.000 |
| OTU_277 | 0.786 | 0.100 | **< 0.001** | 1.000 | 0.132 | 0.906 |  | 0.786 | 1.000 | **0.002** | 0.328 | 1.000 | **0.021** |  | 1.000 | 1.000 | 1.000 | 1.000 | 1.000 | 0.524 |
| OTU_278 | 1.000 | **< 0.001** | **0.005** | **< 0.001** | **0.004** | 1.000 |  | 1.000 | **< 0.001** | 0.203 | **< 0.001** | **0.006** | 0.344 |  | 1.000 | **0.006** | 1.000 | **< 0.001** | 0.204 | 1.000 |
| OTU_279 | 1.000 | **< 0.001** | **< 0.001** | **< 0.001** | **< 0.001** | **< 0.001** |  | 1.000 | **< 0.001** | **< 0.001** | **< 0.001** | **< 0.001** | **< 0.001** |  | 1.000 | **< 0.001** | **< 0.001** | **< 0.001** | **0.026** | **< 0.001** |
| OTU_28 | **< 0.001** | **< 0.001** | **< 0.001** | **0.024** | **< 0.001** | **< 0.001** |  | **< 0.001** | **< 0.001** | **< 0.001** | **0.001** | **< 0.001** | 0.062 |  | **< 0.001** | **< 0.001** | **< 0.001** | **< 0.001** | **< 0.001** | 0.095 |
| OTU_280 | **0.001** | **< 0.001** | 1.000 | 1.000 | **0.016** | **0.002** |  | **0.001** | **< 0.001** | 1.000 | 1.000 | **< 0.001** | **0.002** |  | 0.223 | 0.052 | 1.000 | 1.000 | **0.024** | **0.025** |
| OTU_2808 | 0.767 | 0.258 | 1.000 | 1.000 | **0.002** | **0.001** |  | 0.767 | 1.000 | 0.545 | 1.000 | **0.027** | **0.005** |  | 1.000 | 1.000 | **0.039** | 1.000 | 0.066 | **0.026** |
| OTU_281 | 0.099 | **< 0.001** | **0.002** | **0.005** | 1.000 | 0.155 |  | 0.099 | **< 0.001** | **< 0.001** | **< 0.001** | 0.852 | 0.299 |  | 0.112 | **< 0.001** | **< 0.001** | 0.081 | 1.000 | 1.000 |
| OTU_282 | 1.000 | **< 0.001** | **< 0.001** | **< 0.001** | **< 0.001** | 1.000 |  | 1.000 | **< 0.001** | **< 0.001** | **< 0.001** | **< 0.001** | 1.000 |  | 1.000 | **< 0.001** | **< 0.001** | **< 0.001** | **< 0.001** | 1.000 |
| OTU_2824 | 1.000 | 1.000 | 1.000 | 1.000 | 1.000 | 1.000 |  | 1.000 | **< 0.001** | 0.705 | 1.000 | 0.813 | 0.485 |  | 1.000 | 0.391 | 1.000 | 1.000 | 1.000 | 1.000 |
| OTU_2826 | 1.000 | 1.000 | 1.000 | 1.000 | 1.000 | 1.000 |  | 1.000 | **0.006** | **< 0.001** | 1.000 | 0.295 | 1.000 |  | 1.000 | 0.290 | 0.069 | 0.471 | 0.090 | 1.000 |
| OTU_283 | 1.000 | 0.173 | 1.000 | **0.004** | 1.000 | 1.000 |  | 1.000 | 0.183 | 1.000 | **0.018** | 1.000 | 1.000 |  | 1.000 | 0.106 | 1.000 | **0.023** | 1.000 | 1.000 |
| OTU_2831 | 1.000 | 1.000 | 1.000 | 1.000 | 1.000 | 1.000 |  | 1.000 | 1.000 | 1.000 | **0.044** | 0.147 | 1.000 |  | 1.000 | 1.000 | 1.000 | 1.000 | 1.000 | 1.000 |
| OTU_2839 | 1.000 | 1.000 | 0.639 | 1.000 | 1.000 | 1.000 |  | 1.000 | 1.000 | 0.555 | 1.000 | 0.650 | 1.000 |  | 1.000 | 1.000 | 0.079 | 1.000 | 0.391 | 1.000 |
| OTU_284 | 1.000 | 1.000 | **< 0.001** | 1.000 | **< 0.001** | **0.002** |  | 1.000 | 1.000 | 0.079 | 1.000 | **< 0.001** | **< 0.001** |  | 1.000 | 1.000 | 0.143 | 1.000 | **0.002** | 0.082 |
| OTU_2848 | 1.000 | 1.000 | 0.553 | 1.000 | 0.643 | 1.000 |  | 1.000 | 0.568 | **0.003** | 1.000 | 0.193 | 1.000 |  | 1.000 | 1.000 | 1.000 | 1.000 | 0.201 | 1.000 |
| OTU_285 | **< 0.001** | **< 0.001** | **< 0.001** | 0.065 | **0.020** | 1.000 |  | **< 0.001** | **< 0.001** | **< 0.001** | **0.010** | **< 0.001** | 1.000 |  | **< 0.001** | **< 0.001** | **< 0.001** | 1.000 | 1.000 | 1.000 |
| OTU_2858 | 1.000 | 0.513 | 1.000 | 0.217 | 1.000 | 0.342 |  | 1.000 | 1.000 | 1.000 | 0.287 | 1.000 | 0.273 |  | 1.000 | 1.000 | 1.000 | 1.000 | 1.000 | 1.000 |
| OTU_286 | 1.000 | 1.000 | 1.000 | 1.000 | 1.000 | 1.000 |  | 1.000 | 1.000 | 1.000 | 1.000 | 1.000 | 1.000 |  | 1.000 | **0.039** | 0.104 | 1.000 | 1.000 | 1.000 |
| OTU_2862 | 1.000 | **< 0.001** | 0.242 | **0.005** | 0.609 | 1.000 |  | 1.000 | **< 0.001** | **< 0.001** | **0.010** | 0.808 | 1.000 |  | 0.079 | **< 0.001** | **0.001** | 0.063 | 1.000 | 0.787 |
| OTU_287 | 1.000 | **< 0.001** | **< 0.001** | **< 0.001** | **0.002** | 0.875 |  | 1.000 | **< 0.001** | **< 0.001** | **< 0.001** | **< 0.001** | 1.000 |  | 1.000 | **< 0.001** | **< 0.001** | **< 0.001** | **0.003** | 1.000 |
| OTU_288 | 1.000 | 1.000 | 1.000 | 1.000 | 0.150 | 1.000 |  | 1.000 | 1.000 | 0.265 | 0.208 | **0.021** | 1.000 |  | 1.000 | 1.000 | 1.000 | 1.000 | 1.000 | 1.000 |
| OTU_289 | 1.000 | **< 0.001** | **< 0.001** | **< 0.001** | **< 0.001** | 1.000 |  | 1.000 | **< 0.001** | **< 0.001** | **< 0.001** | **< 0.001** | 1.000 |  | **0.006** | **< 0.001** | **< 0.001** | **< 0.001** | **0.001** | 1.000 |
| OTU_2891 | 1.000 | 1.000 | 0.096 | 1.000 | 1.000 | 1.000 |  | 1.000 | **0.003** | **< 0.001** | 1.000 | 1.000 | 0.091 |  | 1.000 | 1.000 | 1.000 | 1.000 | 1.000 | 1.000 |
| OTU_29 | **< 0.001** | **< 0.001** | **< 0.001** | 0.217 | 1.000 | 0.729 |  | **< 0.001** | **< 0.001** | **< 0.001** | 0.287 | 1.000 | 0.983 |  | 1.000 | **0.038** | 1.000 | 1.000 | 1.000 | 0.617 |
| OTU_290 | **0.002** | 1.000 | **< 0.001** | **0.017** | **< 0.001** | **< 0.001** |  | **0.002** | 1.000 | **< 0.001** | **0.012** | **< 0.001** | **< 0.001** |  | 1.000 | 1.000 | **0.001** | 0.702 | **< 0.001** | 0.094 |
| OTU_2903 | 1.000 | 1.000 | 0.980 | 1.000 | 1.000 | 0.056 |  | 1.000 | 1.000 | 0.350 | 1.000 | 1.000 | 0.626 |  | 1.000 | 1.000 | 1.000 | 0.674 | 1.000 | 0.825 |
| OTU_291 | **0.001** | **0.002** | 1.000 | 1.000 | **< 0.001** | **0.002** |  | **0.001** | 1.000 | 0.652 | 1.000 | **0.043** | **0.013** |  | 0.074 | **0.002** | 1.000 | 1.000 | 0.802 | 0.189 |
| OTU_2915 | 1.000 | 0.333 | 0.122 | 1.000 | 1.000 | 1.000 |  | 1.000 | 1.000 | 1.000 | 1.000 | 1.000 | 1.000 |  | 1.000 | 1.000 | 1.000 | 1.000 | 1.000 | 1.000 |
| OTU_292 | **< 0.001** | **< 0.001** | **0.001** | 0.347 | **0.016** | 1.000 |  | **< 0.001** | **0.003** | 0.100 | 1.000 | 0.064 | 1.000 |  | 0.432 | 0.085 | 1.000 | 1.000 | 1.000 | 1.000 |
| OTU_293 | **< 0.001** | **< 0.001** | **< 0.001** | **0.031** | 1.000 | **0.004** |  | **< 0.001** | **< 0.001** | 0.083 | 0.449 | 1.000 | **0.026** |  | **< 0.001** | **< 0.001** | 0.051 | 1.000 | 0.062 | **0.009** |
| OTU_2939 | 1.000 | 1.000 | 1.000 | 1.000 | 1.000 | 1.000 |  | 1.000 | 1.000 | 1.000 | 1.000 | 1.000 | 1.000 |  | 1.000 | 1.000 | 1.000 | 1.000 | 1.000 | 1.000 |
| OTU_294 | 1.000 | 1.000 | 1.000 | 0.239 | 0.763 | 1.000 |  | 1.000 | 1.000 | 1.000 | 1.000 | 1.000 | 1.000 |  | 1.000 | 1.000 | 0.326 | 1.000 | 0.597 | 1.000 |
| OTU_2941 | 1.000 | 1.000 | 1.000 | 1.000 | 1.000 | 1.000 |  | 1.000 | 1.000 | 1.000 | 1.000 | 1.000 | 1.000 |  | 1.000 | 1.000 | 1.000 | 0.681 | 1.000 | 1.000 |
| OTU_2949 | 1.000 | 1.000 | 1.000 | 1.000 | 0.194 | 0.825 |  | 1.000 | 1.000 | 0.141 | 1.000 | 1.000 | 1.000 |  | 1.000 | 1.000 | 0.131 | 1.000 | 0.621 | 1.000 |
| OTU_295 | 0.171 | **< 0.001** | 0.152 | 1.000 | 1.000 | 1.000 |  | 0.171 | **< 0.001** | **< 0.001** | 0.374 | 0.474 | 1.000 |  | **< 0.001** | **< 0.001** | **0.003** | 1.000 | 1.000 | 1.000 |
| OTU_2958 | 0.735 | 1.000 | 0.950 | 1.000 | 1.000 | 1.000 |  | 0.735 | 1.000 | 1.000 | 1.000 | 1.000 | 1.000 |  | 0.227 | 1.000 | 1.000 | 0.084 | 1.000 | 1.000 |
| OTU_296 | **< 0.001** | **< 0.001** | **0.002** | 1.000 | **< 0.001** | 0.152 |  | **< 0.001** | **< 0.001** | **< 0.001** | 0.094 | **< 0.001** | 0.479 |  | **< 0.001** | **< 0.001** | 1.000 | 1.000 | **< 0.001** | 0.468 |
| OTU_2961 | 0.220 | 1.000 | 1.000 | 0.058 | **0.027** | 1.000 |  | 0.220 | 1.000 | 1.000 | 0.460 | 0.262 | 1.000 |  | 1.000 | 1.000 | 1.000 | 0.484 | **0.041** | 1.000 |
| OTU_297 | 1.000 | 1.000 | **0.035** | 1.000 | **< 0.001** | 0.080 |  | 1.000 | 1.000 | **0.004** | 1.000 | 0.169 | **0.006** |  | 1.000 | 1.000 | 1.000 | 1.000 | 1.000 | 1.000 |
| OTU_298 | 0.371 | 1.000 | 1.000 | **0.040** | 0.505 | 1.000 |  | 0.371 | 0.164 | 0.733 | 0.740 | 1.000 | 1.000 |  | 1.000 | 1.000 | 1.000 | 1.000 | 0.424 | 1.000 |
| OTU_299 | **0.001** | **< 0.001** | **< 0.001** | 0.308 | **0.002** | 1.000 |  | **0.001** | 0.057 | **0.004** | 0.905 | 0.096 | 1.000 |  | 1.000 | **0.046** | **0.001** | 1.000 | **0.050** | 1.000 |
| OTU_2999 | 1.000 | 1.000 | 0.282 | 1.000 | 0.098 | 1.000 |  | 1.000 | 1.000 | 1.000 | 1.000 | 0.759 | 1.000 |  | 1.000 | 1.000 | **0.034** | 1.000 | 0.266 | 1.000 |
| OTU_3 | **0.020** | **< 0.001** | **< 0.001** | 1.000 | 1.000 | 1.000 |  | **0.020** | **< 0.001** | **< 0.001** | 1.000 | 1.000 | 1.000 |  | **0.017** | **< 0.001** | 0.089 | 1.000 | 1.000 | 1.000 |
| OTU_30 | 0.076 | **< 0.001** | **0.005** | **< 0.001** | 1.000 | **< 0.001** |  | 0.076 | **< 0.001** | **0.002** | **< 0.001** | 0.922 | **0.015** |  | **< 0.001** | **< 0.001** | **< 0.001** | **0.025** | 1.000 | **0.011** |
| OTU_300 | 1.000 | 1.000 | 0.479 | 1.000 | 1.000 | 1.000 |  | 1.000 | **0.033** | **0.001** | 1.000 | 1.000 | 1.000 |  | 1.000 | 0.914 | 0.359 | 1.000 | 1.000 | 1.000 |
| OTU_301 | 1.000 | 1.000 | 1.000 | 1.000 | 1.000 | 1.000 |  | 1.000 | **0.021** | 0.074 | 1.000 | 1.000 | 1.000 |  | 1.000 | 1.000 | 1.000 | 1.000 | 1.000 | 1.000 |
| OTU_3012 | 1.000 | **0.016** | 1.000 | 1.000 | 1.000 | 0.080 |  | 1.000 | **< 0.001** | **< 0.001** | 0.439 | 1.000 | 0.204 |  | 1.000 | **< 0.001** | 1.000 | **0.035** | 1.000 | 0.542 |
| OTU_302 | 1.000 | 1.000 | 0.850 | 0.784 | 0.150 | 1.000 |  | 1.000 | 1.000 | 0.245 | 1.000 | 0.926 | 1.000 |  | **0.005** | **< 0.001** | **< 0.001** | 1.000 | 1.000 | 1.000 |
| OTU_303 | 1.000 | 1.000 | 1.000 | 1.000 | 1.000 | 1.000 |  | 1.000 | 1.000 | **0.020** | 0.446 | **< 0.001** | 1.000 |  | 1.000 | 1.000 | 1.000 | 1.000 | 1.000 | 1.000 |
| OTU_3030 | 1.000 | 1.000 | 1.000 | 1.000 | 1.000 | 1.000 |  | 1.000 | 1.000 | 1.000 | 1.000 | 1.000 | 1.000 |  | 1.000 | 1.000 | 1.000 | 1.000 | 1.000 | 1.000 |
| OTU_3031 | 1.000 | 0.078 | 1.000 | 1.000 | 1.000 | 1.000 |  | 1.000 | 0.054 | 1.000 | 1.000 | 1.000 | 1.000 |  | 0.651 | **< 0.001** | **0.007** | 0.888 | 1.000 | 1.000 |
| OTU_3032 | 1.000 | 1.000 | 1.000 | 1.000 | 1.000 | 1.000 |  | 1.000 | 1.000 | 1.000 | 1.000 | 1.000 | 1.000 |  | 1.000 | 1.000 | 1.000 | 1.000 | 1.000 | 1.000 |
| OTU_3039 | 0.702 | **0.002** | 1.000 | 1.000 | 1.000 | **0.023** |  | 0.702 | **0.003** | 0.201 | **0.005** | 0.232 | 1.000 |  | 0.714 | **< 0.001** | **0.013** | 0.317 | 1.000 | 1.000 |
| OTU_3049 | 1.000 | 0.837 | **0.012** | 1.000 | 1.000 | 1.000 |  | 1.000 | **0.016** | **0.002** | 1.000 | 0.581 | 1.000 |  | 1.000 | 1.000 | 0.412 | 1.000 | 0.780 | 1.000 |
| OTU_305 | 1.000 | 0.051 | 1.000 | **0.010** | 0.761 | 1.000 |  | 1.000 | **0.005** | 0.401 | 0.097 | 1.000 | 1.000 |  | 1.000 | **< 0.001** | 0.243 | **< 0.001** | 0.604 | 1.000 |
| OTU_3051 | 0.123 | **0.003** | 0.067 | 1.000 | 1.000 | 1.000 |  | 0.123 | **< 0.001** | **0.008** | 1.000 | 1.000 | 1.000 |  | 0.246 | 0.120 | 1.000 | 1.000 | 1.000 | 1.000 |
| OTU_3053 | 1.000 | **0.033** | 0.190 | 0.231 | **0.047** | **< 0.001** |  | 1.000 | **< 0.001** | 1.000 | 0.567 | 0.517 | **< 0.001** |  | 0.223 | **< 0.001** | 1.000 | **0.014** | 1.000 | **< 0.001** |
| OTU_3058 | 1.000 | 0.497 | 0.418 | 1.000 | 1.000 | 1.000 |  | 1.000 | 1.000 | 1.000 | 1.000 | 1.000 | 1.000 |  | 1.000 | 1.000 | 1.000 | 1.000 | 1.000 | 1.000 |
| OTU_3059 | **0.024** | **0.013** | 0.076 | 1.000 | 1.000 | 1.000 |  | **0.024** | 1.000 | 1.000 | 1.000 | 1.000 | 1.000 |  | 1.000 | 0.141 | 1.000 | 1.000 | 1.000 | 1.000 |
| OTU_306 | 1.000 | 1.000 | 1.000 | 1.000 | 1.000 | 1.000 |  | 1.000 | 1.000 | 1.000 | 1.000 | 1.000 | 1.000 |  | 1.000 | 1.000 | 1.000 | 1.000 | 1.000 | 1.000 |
| OTU_307 | **0.006** | **0.014** | **0.004** | 1.000 | 1.000 | 1.000 |  | **0.006** | **0.004** | 0.128 | 1.000 | 1.000 | 1.000 |  | 0.352 | **0.004** | 0.231 | 1.000 | 1.000 | 1.000 |
| OTU_3074 | 0.406 | 1.000 | 1.000 | 1.000 | **0.003** | 0.079 |  | 0.406 | 0.644 | 1.000 | 1.000 | **0.032** | 1.000 |  | 1.000 | 0.471 | 0.340 | 1.000 | 0.131 | **0.001** |
| OTU_308 | 0.134 | **< 0.001** | **0.047** | 1.000 | 1.000 | 1.000 |  | 0.134 | 0.050 | 1.000 | 1.000 | 1.000 | 1.000 |  | **0.014** | **0.012** | 0.213 | 1.000 | 1.000 | 1.000 |
| OTU_309 | 1.000 | 0.434 | 1.000 | 1.000 | 0.414 | **0.024** |  | 1.000 | 1.000 | 1.000 | 1.000 | **0.040** | 0.532 |  | 0.411 | **0.027** | 1.000 | 1.000 | 1.000 | 1.000 |
| OTU_3090 | 1.000 | 1.000 | 1.000 | 1.000 | 1.000 | 1.000 |  | 1.000 | 1.000 | 1.000 | 1.000 | 1.000 | 1.000 |  | 1.000 | 1.000 | 1.000 | 1.000 | 1.000 | 1.000 |
| OTU_3091 | 1.000 | 1.000 | **< 0.001** | 1.000 | **< 0.001** | **0.011** |  | 1.000 | **0.028** | **< 0.001** | 0.052 | **< 0.001** | 1.000 |  | 1.000 | 1.000 | **0.016** | 1.000 | **< 0.001** | **< 0.001** |
| OTU_3096 | 1.000 | **< 0.001** | **0.032** | **< 0.001** | 1.000 | **< 0.001** |  | 1.000 | **< 0.001** | **< 0.001** | **< 0.001** | 1.000 | **0.024** |  | **0.002** | **< 0.001** | **< 0.001** | **< 0.001** | 1.000 | **< 0.001** |
| OTU_31 | 0.064 | **< 0.001** | **0.007** | **0.022** | 1.000 | 0.095 |  | 0.064 | **< 0.001** | **0.002** | 0.136 | 1.000 | 0.508 |  | **0.002** | **< 0.001** | **< 0.001** | 0.471 | 1.000 | 1.000 |
| OTU_310 | 1.000 | 0.241 | **< 0.001** | 1.000 | **< 0.001** | **0.002** |  | 1.000 | **0.011** | **< 0.001** | 1.000 | **< 0.001** | **< 0.001** |  | 0.149 | **0.046** | **< 0.001** | 1.000 | **0.004** | **0.009** |
| OTU_311 | **< 0.001** | **< 0.001** | **< 0.001** | 1.000 | 1.000 | 1.000 |  | **< 0.001** | **< 0.001** | **< 0.001** | 1.000 | 1.000 | 1.000 |  | **0.045** | **< 0.001** | **< 0.001** | 1.000 | 1.000 | 1.000 |
| OTU_312 | 1.000 | 1.000 | 1.000 | 1.000 | 1.000 | 1.000 |  | 1.000 | 0.165 | 0.488 | 1.000 | 1.000 | 1.000 |  | 1.000 | **0.022** | 1.000 | 1.000 | 1.000 | 0.708 |
| OTU_3120 | 1.000 | 0.617 | 1.000 | **0.003** | 0.170 | 1.000 |  | 1.000 | 0.908 | 1.000 | 1.000 | 1.000 | 1.000 |  | 1.000 | 1.000 | 1.000 | 1.000 | 1.000 | 1.000 |
| OTU_313 | 0.141 | **< 0.001** | **0.006** | 1.000 | 1.000 | 1.000 |  | 0.141 | 1.000 | 0.642 | 1.000 | 1.000 | 1.000 |  | 1.000 | **0.001** | **< 0.001** | **0.003** | **< 0.001** | 1.000 |
| OTU_314 | 1.000 | 1.000 | 1.000 | 1.000 | 1.000 | 1.000 |  | 1.000 | 1.000 | 1.000 | 1.000 | 1.000 | 1.000 |  | 1.000 | 1.000 | 0.431 | 1.000 | 1.000 | 1.000 |
| OTU_315 | **< 0.001** | **< 0.001** | **< 0.001** | 0.664 | 1.000 | 0.160 |  | **< 0.001** | **< 0.001** | 0.357 | 1.000 | 1.000 | 0.357 |  | 0.443 | **< 0.001** | 0.459 | 1.000 | 1.000 | 0.880 |
| OTU_3156 | 1.000 | 1.000 | **0.032** | 1.000 | **0.031** | 0.235 |  | 1.000 | **< 0.001** | **< 0.001** | 1.000 | 0.214 | 1.000 |  | 1.000 | 1.000 | 0.132 | 1.000 | 1.000 | 1.000 |
| OTU_316 | **0.004** | **0.002** | 1.000 | 1.000 | **0.004** | **0.007** |  | **0.004** | 1.000 | 1.000 | 1.000 | 0.313 | 1.000 |  | **< 0.001** | **0.008** | 1.000 | 1.000 | **< 0.001** | **0.009** |
| OTU_3169 | 1.000 | 0.053 | **0.011** | **0.048** | **0.006** | 1.000 |  | 1.000 | **< 0.001** | **< 0.001** | **0.003** | 0.079 | 1.000 |  | 1.000 | **< 0.001** | **0.028** | 0.090 | 1.000 | 1.000 |
| OTU_317 | **< 0.001** | **< 0.001** | **< 0.001** | 0.114 | 1.000 | 1.000 |  | **< 0.001** | **< 0.001** | **< 0.001** | **0.021** | 0.275 | 1.000 |  | **< 0.001** | **< 0.001** | **< 0.001** | **0.002** | **0.050** | 1.000 |
| OTU_318 | 1.000 | 1.000 | 0.381 | 1.000 | 1.000 | 1.000 |  | 1.000 | 1.000 | 1.000 | 1.000 | 0.435 | 1.000 |  | 1.000 | 0.400 | 1.000 | 1.000 | 1.000 | 1.000 |
| OTU_3185 | 1.000 | 1.000 | **0.023** | 1.000 | 0.851 | 1.000 |  | 1.000 | 1.000 | 1.000 | 0.868 | 0.198 | 1.000 |  | 1.000 | 0.224 | 0.062 | 1.000 | 1.000 | 1.000 |
| OTU_3189 | 1.000 | 1.000 | 1.000 | 1.000 | 1.000 | 1.000 |  | 1.000 | 1.000 | 1.000 | 0.065 | 0.403 | 1.000 |  | 1.000 | 0.463 | 1.000 | 1.000 | 1.000 | 1.000 |
| OTU_319 | 1.000 | **0.006** | **0.038** | 1.000 | 1.000 | 1.000 |  | 1.000 | 0.997 | 0.308 | 1.000 | 0.713 | 1.000 |  | 1.000 | 0.095 | **0.016** | 1.000 | 0.368 | 1.000 |
| OTU_32 | **0.001** | **< 0.001** | 1.000 | 0.143 | 0.163 | **< 0.001** |  | **0.001** | **< 0.001** | 0.980 | 1.000 | 0.111 | **0.002** |  | **0.007** | **< 0.001** | 0.508 | 1.000 | 1.000 | **0.009** |
| OTU_320 | **< 0.001** | **< 0.001** | 0.668 | 1.000 | 0.174 | 0.543 |  | **< 0.001** | 0.175 | 0.588 | 1.000 | 1.000 | 1.000 |  | 1.000 | **0.029** | 1.000 | 1.000 | 1.000 | 0.662 |
| OTU_3203 | 1.000 | **< 0.001** | 0.061 | **0.013** | 1.000 | 1.000 |  | 1.000 | 1.000 | 1.000 | 1.000 | 1.000 | 1.000 |  | 1.000 | 1.000 | 0.688 | 1.000 | 1.000 | 1.000 |
| OTU_3205 | 1.000 | 1.000 | 1.000 | 1.000 | 1.000 | 1.000 |  | 1.000 | 1.000 | 1.000 | 1.000 | 1.000 | 1.000 |  | 1.000 | 0.159 | 1.000 | 1.000 | 1.000 | 1.000 |
| OTU_3206 | 1.000 | **0.005** | **< 0.001** | 0.378 | **0.005** | 1.000 |  | 1.000 | **< 0.001** | **< 0.001** | 1.000 | 0.089 | 1.000 |  | 1.000 | 0.411 | 0.066 | 1.000 | 0.985 | 1.000 |
| OTU_3208 | **< 0.001** | 0.417 | **< 0.001** | 0.884 | **< 0.001** | **< 0.001** |  | **< 0.001** | 1.000 | **< 0.001** | **0.010** | **< 0.001** | **< 0.001** |  | 1.000 | 1.000 | **< 0.001** | 1.000 | **< 0.001** | **< 0.001** |
| OTU_321 | 1.000 | 0.830 | **0.015** | 1.000 | **0.013** | 1.000 |  | 1.000 | 1.000 | **< 0.001** | 1.000 | **0.026** | 0.695 |  | 1.000 | 0.167 | **0.001** | 1.000 | **0.027** | 1.000 |
| OTU_322 | **0.002** | 1.000 | 1.000 | 0.064 | **< 0.001** | 0.466 |  | **0.002** | 1.000 | 0.053 | 0.954 | **< 0.001** | **0.004** |  | 0.422 | 1.000 | **0.002** | 0.184 | **< 0.001** | **0.011** |
| OTU_323 | 1.000 | **< 0.001** | 0.057 | 0.740 | 1.000 | 1.000 |  | 1.000 | 0.196 | 1.000 | 1.000 | 1.000 | 1.000 |  | 1.000 | 1.000 | 1.000 | 1.000 | 1.000 | 1.000 |
| OTU_3235 | 1.000 | 1.000 | 1.000 | 1.000 | 1.000 | 1.000 |  | 1.000 | 1.000 | 1.000 | 1.000 | 1.000 | 1.000 |  | 1.000 | 1.000 | 0.339 | 1.000 | 1.000 | 1.000 |
| OTU_3237 | 1.000 | 1.000 | 1.000 | 1.000 | 1.000 | 1.000 |  | 1.000 | 1.000 | 0.776 | 1.000 | 0.315 | 0.679 |  | 1.000 | 1.000 | **0.022** | 1.000 | **0.041** | 0.517 |
| OTU_324 | 1.000 | **< 0.001** | **< 0.001** | **< 0.001** | 0.071 | 1.000 |  | 1.000 | **< 0.001** | **< 0.001** | **0.001** | 1.000 | 0.313 |  | 1.000 | **< 0.001** | 0.171 | 0.103 | 1.000 | 1.000 |
| OTU_3242 | 1.000 | 1.000 | 1.000 | 1.000 | 1.000 | 1.000 |  | 1.000 | 1.000 | 1.000 | 1.000 | 1.000 | 1.000 |  | 1.000 | 1.000 | 1.000 | 1.000 | 1.000 | 1.000 |
| OTU_325 | **0.003** | **0.002** | **< 0.001** | 1.000 | 1.000 | 1.000 |  | **0.003** | **< 0.001** | **< 0.001** | **0.002** | **< 0.001** | 1.000 |  | 1.000 | 0.053 | **< 0.001** | 0.170 | **< 0.001** | 1.000 |
| OTU_3255 | 1.000 | 1.000 | 1.000 | 1.000 | 1.000 | 1.000 |  | 1.000 | 1.000 | 1.000 | 1.000 | 1.000 | 1.000 |  | 1.000 | 0.156 | 1.000 | 1.000 | 1.000 | 1.000 |
| OTU_326 | 1.000 | **0.007** | **0.011** | 0.753 | 0.765 | 1.000 |  | 1.000 | **< 0.001** | **< 0.001** | 1.000 | 1.000 | 1.000 |  | **< 0.001** | **< 0.001** | **< 0.001** | 1.000 | 1.000 | 1.000 |
| OTU_3265 | 1.000 | 1.000 | 1.000 | 1.000 | 1.000 | 1.000 |  | 1.000 | 1.000 | 1.000 | 1.000 | 1.000 | 1.000 |  | 1.000 | 1.000 | 1.000 | 1.000 | 1.000 | 1.000 |
| OTU_3267 | 1.000 | 1.000 | 1.000 | 1.000 | 1.000 | 1.000 |  | 1.000 | 1.000 | 0.323 | 1.000 | 1.000 | 1.000 |  | 1.000 | 1.000 | 1.000 | 1.000 | 0.500 | 1.000 |
| OTU_327 | **0.009** | **< 0.001** | 1.000 | 1.000 | 0.446 | **0.013** |  | **0.009** | **< 0.001** | **0.019** | 1.000 | 0.324 | 0.144 |  | **0.019** | **< 0.001** | 0.340 | 1.000 | 1.000 | 0.364 |
| OTU_328 | 1.000 | 1.000 | 0.147 | 1.000 | **0.003** | 0.443 |  | 1.000 | 1.000 | 0.551 | 1.000 | 0.079 | 0.525 |  | 1.000 | 1.000 | **0.006** | 1.000 | 0.257 | 1.000 |
| OTU_329 | **< 0.001** | **< 0.001** | **< 0.001** | 1.000 | 1.000 | 1.000 |  | **< 0.001** | **< 0.001** | **0.011** | 1.000 | 1.000 | 1.000 |  | **0.027** | **0.004** | 0.087 | 1.000 | 1.000 | 1.000 |
| OTU_3290 | 1.000 | 1.000 | 1.000 | 0.688 | 1.000 | 1.000 |  | 1.000 | 1.000 | 1.000 | 1.000 | 1.000 | 1.000 |  | 1.000 | 1.000 | 1.000 | 1.000 | 1.000 | 1.000 |
| OTU_3294 | 0.400 | 1.000 | 1.000 | 1.000 | 0.195 | 1.000 |  | 0.400 | 1.000 | 1.000 | 0.997 | 0.529 | 1.000 |  | 1.000 | 1.000 | 0.188 | 1.000 | 1.000 | 1.000 |
| OTU_33 | **0.049** | 0.171 | **< 0.001** | 1.000 | 0.825 | 0.246 |  | **0.049** | 0.067 | **< 0.001** | 1.000 | 1.000 | 0.332 |  | **< 0.001** | **0.006** | **< 0.001** | 1.000 | 1.000 | 1.000 |
| OTU_330 | 0.755 | **< 0.001** | **< 0.001** | **0.024** | **0.005** | 1.000 |  | 0.755 | **< 0.001** | **< 0.001** | **< 0.001** | **< 0.001** | 1.000 |  | 0.227 | **< 0.001** | **< 0.001** | **< 0.001** | **0.038** | 1.000 |
| OTU_3308 | 1.000 | 1.000 | **< 0.001** | 0.890 | **< 0.001** | 0.121 |  | 1.000 | 1.000 | **0.035** | 0.662 | **0.005** | 1.000 |  | 1.000 | 1.000 | **0.002** | 1.000 | **0.003** | 0.205 |
| OTU_331 | 1.000 | **< 0.001** | **0.041** | **< 0.001** | 0.168 | 0.104 |  | 1.000 | **< 0.001** | **0.003** | **< 0.001** | **< 0.001** | 1.000 |  | 0.709 | **< 0.001** | **0.002** | **< 0.001** | 1.000 | 0.074 |
| OTU_3314 | **0.002** | **< 0.001** | **< 0.001** | **0.027** | 1.000 | 1.000 |  | **0.002** | **< 0.001** | **0.003** | 1.000 | 1.000 | 1.000 |  | 1.000 | **< 0.001** | **< 0.001** | 0.117 | 0.129 | 1.000 |
| OTU_3318 | 1.000 | 0.177 | 1.000 | 0.890 | 0.906 | **0.010** |  | 1.000 | 1.000 | 0.139 | 1.000 | 0.523 | 1.000 |  | 1.000 | 1.000 | 0.283 | 1.000 | 0.395 | 1.000 |
| OTU_3319 | 1.000 | 1.000 | 0.158 | 1.000 | 1.000 | 1.000 |  | 1.000 | **< 0.001** | **< 0.001** | 0.185 | **< 0.001** | 0.583 |  | 1.000 | 1.000 | 0.238 | 1.000 | **0.012** | 1.000 |
| OTU_332 | 1.000 | 1.000 | 1.000 | 1.000 | 1.000 | 1.000 |  | 1.000 | 1.000 | 1.000 | 1.000 | 1.000 | 1.000 |  | 1.000 | 1.000 | 0.207 | 1.000 | 0.220 | 1.000 |
| OTU_333 | 1.000 | **< 0.001** | 0.626 | **< 0.001** | 1.000 | **0.027** |  | 1.000 | **< 0.001** | 0.169 | **< 0.001** | **0.022** | **0.025** |  | 1.000 | **< 0.001** | 0.238 | 0.157 | 1.000 | 0.774 |
| OTU_3331 | 1.000 | 1.000 | 1.000 | 1.000 | 1.000 | 1.000 |  | 1.000 | 1.000 | 1.000 | 1.000 | 0.795 | 1.000 |  | 1.000 | 1.000 | 1.000 | 1.000 | 1.000 | 1.000 |
| OTU_3335 | 1.000 | **0.016** | 1.000 | **< 0.001** | 1.000 | **< 0.001** |  | 1.000 | 1.000 | 1.000 | 1.000 | 0.075 | 0.231 |  | 1.000 | **< 0.001** | 0.276 | **< 0.001** | 1.000 | 0.065 |
| OTU_3339 | 1.000 | 1.000 | 0.819 | 1.000 | 0.375 | 1.000 |  | 1.000 | 0.316 | 0.502 | 0.439 | 0.586 | 1.000 |  | 1.000 | **0.028** | 0.436 | 0.221 | 1.000 | 1.000 |
| OTU_334 | 0.801 | 1.000 | 1.000 | 1.000 | **0.003** | 0.352 |  | 0.801 | 1.000 | 0.091 | 1.000 | **0.001** | 0.223 |  | 1.000 | 1.000 | 1.000 | 1.000 | 1.000 | 1.000 |
| OTU_3344 | 1.000 | **0.028** | 1.000 | **0.025** | 1.000 | 1.000 |  | 1.000 | 0.092 | 1.000 | 0.096 | 1.000 | 1.000 |  | 1.000 | 0.107 | 1.000 | 0.809 | 1.000 | 1.000 |
| OTU_335 | 1.000 | **< 0.001** | **< 0.001** | **0.004** | **0.001** | 1.000 |  | 1.000 | **< 0.001** | **< 0.001** | **0.005** | **0.026** | 1.000 |  | 1.000 | **< 0.001** | **< 0.001** | **< 0.001** | **0.008** | 1.000 |
| OTU_3350 | 1.000 | 0.157 | **0.007** | 1.000 | 0.943 | 1.000 |  | 1.000 | **0.002** | 0.060 | 0.941 | 1.000 | 1.000 |  | 0.412 | **< 0.001** | **< 0.001** | 0.809 | 1.000 | 1.000 |
| OTU_3359 | 1.000 | 1.000 | 1.000 | 1.000 | 0.350 | 1.000 |  | 1.000 | **0.024** | 0.057 | **< 0.001** | **< 0.001** | 1.000 |  | 1.000 | 1.000 | 0.125 | 0.220 | **0.015** | 1.000 |
| OTU_336 | 1.000 | **< 0.001** | **< 0.001** | **< 0.001** | **0.005** | 0.105 |  | 1.000 | **< 0.001** | 0.315 | **< 0.001** | 1.000 | 0.069 |  | 1.000 | **< 0.001** | 0.284 | **0.033** | 1.000 | 1.000 |
| OTU_3361 | 1.000 | 0.838 | 1.000 | 1.000 | 1.000 | 1.000 |  | 1.000 | 1.000 | 1.000 | 1.000 | 1.000 | 1.000 |  | 1.000 | 1.000 | 1.000 | 1.000 | 1.000 | 1.000 |
| OTU_337 | 1.000 | 1.000 | 1.000 | 1.000 | 0.229 | 1.000 |  | 1.000 | 0.638 | **< 0.001** | 1.000 | **0.010** | 0.284 |  | 1.000 | 1.000 | **0.008** | 1.000 | 0.900 | 1.000 |
| OTU_3374 | 1.000 | **0.002** | **< 0.001** | **< 0.001** | **< 0.001** | 1.000 |  | 1.000 | **< 0.001** | **< 0.001** | **< 0.001** | **< 0.001** | 1.000 |  | 1.000 | **< 0.001** | **< 0.001** | **< 0.001** | **0.002** | 1.000 |
| OTU_338 | 0.139 | **0.001** | **0.008** | 1.000 | 1.000 | 1.000 |  | 0.139 | **0.001** | **0.009** | 1.000 | 1.000 | 1.000 |  | 1.000 | **< 0.001** | **0.002** | **0.024** | 0.439 | 1.000 |
| OTU_339 | 1.000 | 1.000 | 0.065 | 0.060 | **< 0.001** | 1.000 |  | 1.000 | **< 0.001** | **< 0.001** | 0.519 | **0.006** | 1.000 |  | 1.000 | 1.000 | **0.013** | 1.000 | **< 0.001** | **< 0.001** |
| OTU_3396 | 1.000 | 1.000 | 0.132 | 1.000 | **0.038** | 0.235 |  | 1.000 | **0.003** | **< 0.001** | 1.000 | 0.822 | 1.000 |  | 0.960 | 1.000 | **< 0.001** | 1.000 | 1.000 | 0.880 |
| OTU_34 | 1.000 | 1.000 | **< 0.001** | 0.217 | **< 0.001** | **< 0.001** |  | 1.000 | 1.000 | **< 0.001** | 1.000 | **< 0.001** | **< 0.001** |  | 1.000 | 1.000 | **< 0.001** | 1.000 | **< 0.001** | **< 0.001** |
| OTU_340 | **< 0.001** | **< 0.001** | **< 0.001** | 1.000 | 1.000 | 1.000 |  | **< 0.001** | **< 0.001** | **< 0.001** | 0.226 | 1.000 | 1.000 |  | **0.019** | **< 0.001** | **0.002** | 1.000 | 1.000 | 1.000 |
| OTU_3403 | 0.428 | **< 0.001** | **0.003** | **< 0.001** | 1.000 | **0.036** |  | 0.428 | **< 0.001** | **0.032** | **0.005** | 1.000 | 0.050 |  | 0.596 | **< 0.001** | **0.011** | **0.005** | 1.000 | 0.159 |
| OTU_3405 | 0.264 | 1.000 | 1.000 | **0.014** | **0.023** | 1.000 |  | 0.264 | 0.906 | 1.000 | **0.022** | **0.022** | 1.000 |  | 1.000 | 0.186 | 0.290 | 0.157 | 0.194 | 1.000 |
| OTU_341 | 1.000 | **< 0.001** | 0.209 | 0.242 | 1.000 | 1.000 |  | 1.000 | 0.094 | 1.000 | 1.000 | 1.000 | 1.000 |  | 1.000 | **0.009** | 0.193 | 1.000 | 1.000 | 1.000 |
| OTU_342 | 1.000 | 1.000 | 0.717 | 1.000 | 1.000 | 1.000 |  | 1.000 | 1.000 | **0.021** | 1.000 | 1.000 | 1.000 |  | 1.000 | 1.000 | 1.000 | 1.000 | 1.000 | 1.000 |
| OTU_3427 | **< 0.001** | **< 0.001** | **0.010** | **< 0.001** | **0.005** | **< 0.001** |  | **< 0.001** | **< 0.001** | **< 0.001** | **< 0.001** | 1.000 | **< 0.001** |  | **< 0.001** | **< 0.001** | **0.002** | **< 0.001** | **0.012** | **< 0.001** |
| OTU_343 | 1.000 | 0.155 | **0.020** | 1.000 | 1.000 | 1.000 |  | 1.000 | 1.000 | 1.000 | 1.000 | 1.000 | 1.000 |  | 1.000 | 1.000 | 1.000 | 1.000 | 1.000 | 1.000 |
| OTU_3437 | 1.000 | **0.023** | **< 0.001** | 0.247 | **0.005** | 1.000 |  | 1.000 | 1.000 | 1.000 | 1.000 | 1.000 | 1.000 |  | 1.000 | **0.003** | **< 0.001** | 0.736 | 0.062 | 1.000 |
| OTU_344 | 1.000 | **0.004** | 0.421 | **0.005** | 0.384 | 1.000 |  | 1.000 | **< 0.001** | **0.004** | **< 0.001** | **< 0.001** | 1.000 |  | 1.000 | **0.006** | 0.159 | 0.125 | 1.000 | 1.000 |
| OTU_345 | 1.000 | 1.000 | 0.722 | 1.000 | 0.244 | 1.000 |  | 1.000 | **< 0.001** | **< 0.001** | 1.000 | 0.127 | 1.000 |  | 1.000 | 1.000 | 1.000 | 1.000 | 1.000 | 1.000 |
| OTU_346 | 0.201 | **< 0.001** | **< 0.001** | 0.161 | **0.020** | 1.000 |  | 0.201 | **< 0.001** | **< 0.001** | 0.617 | 0.088 | 1.000 |  | 1.000 | **0.025** | **0.005** | 0.105 | **0.017** | 1.000 |
| OTU_3460 | 1.000 | **< 0.001** | **0.003** | **< 0.001** | **0.001** | 0.657 |  | 1.000 | **< 0.001** | **< 0.001** | **0.003** | 1.000 | 0.745 |  | **0.008** | **< 0.001** | **< 0.001** | 0.109 | 1.000 | 1.000 |
| OTU_3469 | 1.000 | 0.054 | **0.045** | 0.312 | 0.192 | 1.000 |  | 1.000 | 1.000 | 1.000 | 1.000 | 0.224 | 1.000 |  | 1.000 | 0.451 | **0.047** | 1.000 | 1.000 | 1.000 |
| OTU_347 | 1.000 | 1.000 | 1.000 | 0.592 | 0.496 | 1.000 |  | 1.000 | 1.000 | 1.000 | 1.000 | 1.000 | 1.000 |  | 1.000 | 1.000 | 1.000 | 1.000 | 1.000 | 1.000 |
| OTU_348 | 0.953 | **0.047** | **< 0.001** | 1.000 | 1.000 | 1.000 |  | 0.953 | **0.005** | **< 0.001** | 1.000 | 0.548 | 1.000 |  | 1.000 | 0.372 | **0.013** | 1.000 | 1.000 | 1.000 |
| OTU_349 | 1.000 | **< 0.001** | **< 0.001** | **0.015** | **0.022** | 1.000 |  | 1.000 | **< 0.001** | **< 0.001** | **< 0.001** | 0.092 | 1.000 |  | 0.259 | **< 0.001** | **< 0.001** | 0.724 | 1.000 | 1.000 |
| OTU_35 | 1.000 | **< 0.001** | **< 0.001** | **< 0.001** | **< 0.001** | 0.216 |  | 1.000 | **< 0.001** | **< 0.001** | **< 0.001** | **< 0.001** | 0.151 |  | 0.620 | **< 0.001** | **< 0.001** | **< 0.001** | **< 0.001** | 1.000 |
| OTU_350 | 1.000 | 1.000 | 0.346 | 1.000 | 1.000 | 1.000 |  | 1.000 | 0.833 | 0.207 | 1.000 | 1.000 | 1.000 |  | 1.000 | 0.961 | 0.776 | 1.000 | 1.000 | 1.000 |
| OTU_3501 | 1.000 | 1.000 | 0.578 | 1.000 | 1.000 | 0.665 |  | 1.000 | 1.000 | **0.001** | 1.000 | 0.946 | 0.066 |  | 1.000 | 0.721 | 1.000 | 1.000 | 1.000 | 1.000 |
| OTU_351 | 1.000 | 1.000 | **0.018** | 1.000 | **0.017** | 1.000 |  | 1.000 | 0.064 | **< 0.001** | 0.107 | **< 0.001** | 1.000 |  | 1.000 | 0.229 | **0.023** | 0.194 | **0.014** | 1.000 |
| OTU_3512 | 1.000 | **< 0.001** | 0.148 | **< 0.001** | 0.083 | 1.000 |  | 1.000 | **< 0.001** | **0.008** | **0.012** | 0.394 | 1.000 |  | 1.000 | **0.006** | 1.000 | **0.001** | 1.000 | 0.221 |
| OTU_3518 | 0.376 | **< 0.001** | 0.141 | 0.268 | 1.000 | 0.409 |  | 0.376 | **< 0.001** | 0.069 | **< 0.001** | 0.081 | **0.007** |  | 1.000 | **< 0.001** | 0.217 | 0.058 | 1.000 | 0.292 |
| OTU_352 | 1.000 | 1.000 | **< 0.001** | 1.000 | **< 0.001** | **< 0.001** |  | 1.000 | 1.000 | **< 0.001** | 1.000 | **< 0.001** | **< 0.001** |  | 1.000 | 1.000 | **< 0.001** | 1.000 | **< 0.001** | **< 0.001** |
| OTU_3521 | 1.000 | 1.000 | **0.015** | 1.000 | 0.535 | 0.816 |  | 1.000 | 1.000 | 0.854 | 0.131 | **0.005** | 1.000 |  | 1.000 | 0.174 | **0.002** | 1.000 | **0.025** | 1.000 |
| OTU_353 | **0.029** | **< 0.001** | **< 0.001** | 1.000 | 0.395 | 1.000 |  | **0.029** | **< 0.001** | **< 0.001** | 1.000 | **0.007** | 1.000 |  | 0.575 | **< 0.001** | **< 0.001** | 1.000 | **0.002** | 0.829 |
| OTU_3530 | 1.000 | 1.000 | **0.003** | 1.000 | **< 0.001** | **< 0.001** |  | 1.000 | 1.000 | 0.277 | 1.000 | **0.038** | 0.103 |  | 0.754 | 1.000 | 0.240 | 0.165 | 1.000 | 0.121 |
| OTU_354 | 1.000 | 1.000 | **< 0.001** | 1.000 | **0.012** | **0.008** |  | 1.000 | 1.000 | **< 0.001** | 1.000 | **0.004** | 0.179 |  | 1.000 | 1.000 | 0.143 | 1.000 | 0.506 | 1.000 |
| OTU_3559 | 0.112 | 1.000 | 1.000 | 1.000 | 1.000 | 1.000 |  | 0.112 | 0.568 | 1.000 | 1.000 | 1.000 | 1.000 |  | 1.000 | 1.000 | 1.000 | 1.000 | 1.000 | 1.000 |
| OTU_356 | 1.000 | 0.256 | 0.118 | 1.000 | 1.000 | 1.000 |  | 1.000 | 0.110 | 0.181 | 1.000 | 1.000 | 1.000 |  | 0.154 | **< 0.001** | **< 0.001** | 1.000 | 1.000 | 1.000 |
| OTU_3569 | 0.646 | **< 0.001** | **< 0.001** | 1.000 | 0.075 | 1.000 |  | 0.646 | **< 0.001** | **< 0.001** | **< 0.001** | **< 0.001** | 1.000 |  | 1.000 | **< 0.001** | **0.009** | 0.060 | 1.000 | 1.000 |
| OTU_357 | 1.000 | 1.000 | 1.000 | 1.000 | 0.294 | 0.133 |  | 1.000 | 0.535 | 1.000 | 0.136 | 1.000 | **0.013** |  | 1.000 | **0.033** | 1.000 | 0.871 | 1.000 | 0.333 |
| OTU_3577 | **< 0.001** | **< 0.001** | **< 0.001** | 1.000 | 1.000 | 1.000 |  | **< 0.001** | **< 0.001** | **< 0.001** | 1.000 | 1.000 | 1.000 |  | **< 0.001** | **< 0.001** | **< 0.001** | 1.000 | 1.000 | 1.000 |
| OTU_3579 | 1.000 | 1.000 | 1.000 | 1.000 | 1.000 | 1.000 |  | 1.000 | 1.000 | 0.747 | 1.000 | 1.000 | 1.000 |  | 1.000 | 1.000 | 0.372 | 1.000 | 1.000 | 1.000 |
| OTU_358 | 1.000 | **< 0.001** | 1.000 | **< 0.001** | 1.000 | **< 0.001** |  | 1.000 | 1.000 | **< 0.001** | 1.000 | **< 0.001** | **< 0.001** |  | 1.000 | **0.025** | 1.000 | 0.064 | 1.000 | **0.006** |
| OTU_3589 | 1.000 | **0.039** | 1.000 | 0.846 | 1.000 | 1.000 |  | 1.000 | **0.016** | 1.000 | 0.891 | 1.000 | 0.849 |  | 1.000 | 0.964 | 0.540 | 1.000 | 1.000 | 1.000 |
| OTU_359 | 1.000 | **< 0.001** | 1.000 | 0.507 | 1.000 | 0.429 |  | 1.000 | **0.016** | 0.915 | 1.000 | 1.000 | 1.000 |  | 1.000 | 0.195 | 1.000 | 1.000 | 1.000 | 1.000 |
| OTU_3594 | 1.000 | 1.000 | 1.000 | 0.826 | 0.128 | 1.000 |  | 1.000 | 1.000 | 0.275 | 0.060 | **< 0.001** | 1.000 |  | 1.000 | 1.000 | 1.000 | 1.000 | 1.000 | 1.000 |
| OTU_3599 | **< 0.001** | **< 0.001** | 1.000 | **0.006** | **< 0.001** | **< 0.001** |  | **< 0.001** | **< 0.001** | 1.000 | **< 0.001** | **< 0.001** | **< 0.001** |  | **< 0.001** | **< 0.001** | 1.000 | **< 0.001** | **< 0.001** | **< 0.001** |
| OTU_36 | **< 0.001** | **< 0.001** | 1.000 | **0.004** | **< 0.001** | **< 0.001** |  | **< 0.001** | **< 0.001** | 1.000 | 0.167 | **0.022** | **< 0.001** |  | 0.490 | **< 0.001** | 1.000 | 1.000 | 0.554 | **0.007** |
| OTU_360 | 1.000 | 1.000 | 1.000 | 1.000 | 1.000 | 1.000 |  | 1.000 | 1.000 | 1.000 | 0.299 | **0.045** | 1.000 |  | 1.000 | 1.000 | 1.000 | 1.000 | 1.000 | 1.000 |
| OTU_361 | 1.000 | 1.000 | 1.000 | 1.000 | 1.000 | 1.000 |  | 1.000 | 1.000 | 1.000 | 1.000 | 1.000 | 1.000 |  | 1.000 | 1.000 | 1.000 | 1.000 | 1.000 | 1.000 |
| OTU_362 | 1.000 | 1.000 | 1.000 | 1.000 | 0.889 | 0.716 |  | 1.000 | 1.000 | 1.000 | 1.000 | 1.000 | 1.000 |  | 1.000 | 1.000 | 1.000 | 1.000 | 0.055 | 1.000 |
| OTU_3625 | 1.000 | 1.000 | 1.000 | 1.000 | 1.000 | 1.000 |  | 1.000 | **0.004** | 1.000 | 1.000 | **< 0.001** | **0.002** |  | 1.000 | 1.000 | 1.000 | 1.000 | **0.021** | 0.085 |
| OTU_3626 | 1.000 | 1.000 | 1.000 | 1.000 | 1.000 | 1.000 |  | 1.000 | 1.000 | 1.000 | 1.000 | 1.000 | 1.000 |  | 0.624 | **< 0.001** | **0.001** | 0.362 | 1.000 | 1.000 |
| OTU_363 | 1.000 | **0.031** | **< 0.001** | 1.000 | **0.038** | 1.000 |  | 1.000 | 1.000 | 0.082 | 1.000 | **0.013** | 1.000 |  | 1.000 | **0.018** | **0.023** | 1.000 | 1.000 | 1.000 |
| OTU_364 | 1.000 | 0.572 | 0.056 | 1.000 | 1.000 | 1.000 |  | 1.000 | **0.018** | **0.014** | **0.028** | **0.017** | 1.000 |  | 1.000 | **0.006** | **< 0.001** | 1.000 | 0.625 | 1.000 |
| OTU_3645 | 1.000 | 0.481 | 1.000 | **0.010** | 1.000 | 1.000 |  | 1.000 | 1.000 | 1.000 | 1.000 | 1.000 | 1.000 |  | 1.000 | **0.002** | **0.003** | 0.097 | 0.085 | 1.000 |
| OTU_365 | 0.834 | 0.565 | 1.000 | 1.000 | 1.000 | 1.000 |  | 0.834 | 1.000 | 1.000 | 1.000 | 1.000 | 1.000 |  | 1.000 | 1.000 | 1.000 | 1.000 | 1.000 | 1.000 |
| OTU_366 | 1.000 | 1.000 | **< 0.001** | 1.000 | **< 0.001** | **< 0.001** |  | 1.000 | 1.000 | **< 0.001** | 1.000 | **< 0.001** | **< 0.001** |  | 1.000 | 1.000 | **< 0.001** | 1.000 | **0.001** | **< 0.001** |
| OTU_367 | 1.000 | **0.046** | **< 0.001** | 1.000 | **< 0.001** | 0.152 |  | 1.000 | **< 0.001** | **< 0.001** | 1.000 | **< 0.001** | 0.143 |  | 1.000 | **0.005** | **< 0.001** | **0.022** | **< 0.001** | 0.804 |
| OTU_368 | 1.000 | 1.000 | **< 0.001** | 1.000 | **< 0.001** | **0.035** |  | 1.000 | **< 0.001** | **< 0.001** | 1.000 | **0.013** | 1.000 |  | 1.000 | 1.000 | 1.000 | 1.000 | 1.000 | 0.352 |
| OTU_3686 | 1.000 | 0.871 | 0.084 | 1.000 | 0.998 | 1.000 |  | 1.000 | 1.000 | **0.019** | 1.000 | 0.610 | 1.000 |  | 1.000 | 1.000 | 0.366 | 1.000 | 1.000 | 1.000 |
| OTU_3688 | 1.000 | **0.049** | **0.021** | 0.291 | 0.092 | 1.000 |  | 1.000 | **< 0.001** | **0.013** | 0.139 | 1.000 | 1.000 |  | 1.000 | **0.005** | 0.388 | 1.000 | 1.000 | 1.000 |
| OTU_369 | 1.000 | **< 0.001** | 0.657 | **< 0.001** | 1.000 | **0.018** |  | 1.000 | **< 0.001** | 0.184 | **0.011** | 1.000 | 0.392 |  | 1.000 | **< 0.001** | 0.253 | **0.021** | 1.000 | 0.095 |
| OTU_3690 | 1.000 | 1.000 | 1.000 | 0.155 | 1.000 | 1.000 |  | 1.000 | **0.016** | 0.229 | 0.155 | 1.000 | 1.000 |  | 0.819 | 1.000 | 1.000 | 1.000 | 1.000 | 1.000 |
| OTU_3699 | 0.941 | **0.022** | **0.020** | 1.000 | 1.000 | 1.000 |  | 0.941 | 0.057 | 0.374 | 1.000 | 1.000 | 1.000 |  | 1.000 | 1.000 | 0.190 | 1.000 | 1.000 | 1.000 |
| OTU_37 | 0.130 | 1.000 | **< 0.001** | **< 0.001** | **< 0.001** | **< 0.001** |  | 0.130 | 1.000 | **< 0.001** | 0.270 | **< 0.001** | **< 0.001** |  | 1.000 | **0.009** | **< 0.001** | **0.033** | **< 0.001** | **< 0.001** |
| OTU_370 | 1.000 | 0.967 | 0.736 | 1.000 | 1.000 | 1.000 |  | 1.000 | 0.138 | 0.799 | 0.077 | 0.387 | 1.000 |  | 1.000 | **0.034** | 0.243 | 0.530 | 1.000 | 1.000 |
| OTU_371 | 1.000 | 1.000 | 1.000 | 1.000 | 1.000 | 1.000 |  | 1.000 | 1.000 | 0.087 | 1.000 | 1.000 | 1.000 |  | 1.000 | **0.034** | 1.000 | 1.000 | 0.733 | 0.084 |
| OTU_3715 | 1.000 | 1.000 | 1.000 | 1.000 | 1.000 | 1.000 |  | 1.000 | 0.358 | 0.579 | 0.454 | 0.625 | 1.000 |  | 0.417 | **0.006** | 0.087 | 1.000 | 1.000 | 1.000 |
| OTU_372 | **0.031** | 0.316 | **0.015** | 1.000 | 1.000 | 1.000 |  | **0.031** | 0.202 | **0.020** | 1.000 | 1.000 | 1.000 |  | 1.000 | 0.369 | **0.047** | 0.471 | **0.045** | 1.000 |
| OTU_373 | 1.000 | **0.001** | **0.004** | **< 0.001** | **< 0.001** | 1.000 |  | 1.000 | **0.002** | **< 0.001** | **0.012** | **< 0.001** | 1.000 |  | 1.000 | **0.005** | **< 0.001** | **< 0.001** | **< 0.001** | 1.000 |
| OTU_3731 | 0.151 | **0.007** | **0.002** | 1.000 | 1.000 | 1.000 |  | 0.151 | **< 0.001** | **0.002** | 1.000 | 1.000 | 1.000 |  | 0.443 | **< 0.001** | **< 0.001** | 1.000 | 1.000 | 1.000 |
| OTU_374 | 1.000 | 1.000 | 1.000 | 1.000 | 1.000 | 0.891 |  | 1.000 | 0.136 | 1.000 | 1.000 | 1.000 | 1.000 |  | 1.000 | 1.000 | 1.000 | 0.127 | 1.000 | 1.000 |
| OTU_3740 | 1.000 | 1.000 | 1.000 | 1.000 | 0.164 | 0.337 |  | 1.000 | 0.306 | **0.002** | 0.430 | **0.003** | 1.000 |  | 1.000 | 1.000 | 1.000 | 1.000 | 0.180 | 1.000 |
| OTU_375 | 1.000 | **< 0.001** | **0.001** | 0.070 | 0.671 | 1.000 |  | 1.000 | **< 0.001** | **< 0.001** | **0.023** | 0.133 | 1.000 |  | 1.000 | **< 0.001** | **0.013** | 0.715 | 1.000 | 1.000 |
| OTU_3757 | 1.000 | 1.000 | **< 0.001** | 0.211 | **< 0.001** | **< 0.001** |  | 1.000 | 1.000 | **< 0.001** | 1.000 | **< 0.001** | **< 0.001** |  | 1.000 | 1.000 | **< 0.001** | 1.000 | **< 0.001** | **< 0.001** |
| OTU_3758 | 1.000 | 1.000 | 1.000 | 1.000 | 1.000 | 1.000 |  | 1.000 | 1.000 | 1.000 | 1.000 | 1.000 | 1.000 |  | 1.000 | 1.000 | 1.000 | 1.000 | 1.000 | 1.000 |
| OTU_376 | 0.416 | **0.002** | 0.051 | 1.000 | 1.000 | 1.000 |  | 0.416 | 0.271 | 0.438 | 1.000 | 1.000 | 1.000 |  | 1.000 | 0.067 | 0.064 | 0.820 | 0.624 | 1.000 |
| OTU_377 | 0.684 | **0.008** | **0.026** | 1.000 | 1.000 | 1.000 |  | 0.684 | 0.760 | 1.000 | 0.829 | 1.000 | 1.000 |  | 1.000 | 0.066 | **< 0.001** | 0.060 | **< 0.001** | 1.000 |
| OTU_378 | 1.000 | 1.000 | 1.000 | 1.000 | 1.000 | 0.816 |  | 1.000 | 1.000 | 1.000 | 1.000 | 1.000 | 1.000 |  | 1.000 | 1.000 | 1.000 | 1.000 | 1.000 | 1.000 |
| OTU_3785 | 1.000 | **< 0.001** | **< 0.001** | 0.138 | **< 0.001** | 1.000 |  | 1.000 | **< 0.001** | **< 0.001** | 1.000 | 1.000 | 1.000 |  | 0.580 | **0.023** | **< 0.001** | 1.000 | **0.032** | 0.511 |
| OTU_379 | 1.000 | 1.000 | **0.009** | 1.000 | **< 0.001** | **0.041** |  | 1.000 | 0.115 | **< 0.001** | 1.000 | **0.027** | 1.000 |  | 1.000 | 1.000 | 1.000 | 1.000 | 1.000 | 1.000 |
| OTU_3799 | 1.000 | 1.000 | 1.000 | 1.000 | 1.000 | 1.000 |  | 1.000 | 0.229 | 0.393 | 1.000 | 1.000 | 1.000 |  | 0.634 | 0.430 | 1.000 | 1.000 | 1.000 | 1.000 |
| OTU_38 | 1.000 | **< 0.001** | 0.055 | 0.544 | 1.000 | 1.000 |  | 1.000 | 1.000 | 0.457 | 1.000 | 1.000 | 1.000 |  | 0.374 | **< 0.001** | 0.283 | 1.000 | 1.000 | 1.000 |
| OTU_380 | 1.000 | 0.281 | 0.444 | 0.159 | 0.200 | 1.000 |  | 1.000 | 0.060 | **< 0.001** | 1.000 | 0.290 | 1.000 |  | 1.000 | 0.081 | 1.000 | 1.000 | 1.000 | 1.000 |
| OTU_3800 | 1.000 | 0.102 | 1.000 | 0.826 | 1.000 | 1.000 |  | 1.000 | **0.018** | 0.500 | 1.000 | 1.000 | 1.000 |  | 1.000 | 0.716 | 0.518 | 0.403 | 0.225 | 1.000 |
| OTU_3808 | 0.253 | 0.099 | **0.014** | 1.000 | 1.000 | 1.000 |  | 0.253 | 0.600 | **0.032** | 1.000 | 1.000 | 1.000 |  | **0.028** | 0.198 | **0.023** | 1.000 | 1.000 | 1.000 |
| OTU_381 | **0.014** | **< 0.001** | **< 0.001** | 0.204 | 0.562 | 1.000 |  | **0.014** | **< 0.001** | **< 0.001** | 0.111 | **< 0.001** | 1.000 |  | 1.000 | **0.007** | **< 0.001** | 0.779 | **< 0.001** | 0.317 |
| OTU_3810 | 1.000 | 1.000 | 0.561 | 1.000 | 1.000 | 1.000 |  | 1.000 | 1.000 | 0.055 | 1.000 | 1.000 | 1.000 |  | 0.723 | 1.000 | 0.293 | 1.000 | 1.000 | 1.000 |
| OTU_3812 | 1.000 | 0.614 | 1.000 | **0.008** | 1.000 | **0.002** |  | 1.000 | 0.896 | 1.000 | 0.066 | 1.000 | **0.023** |  | 1.000 | 1.000 | 1.000 | 0.821 | 1.000 | 1.000 |
| OTU_3814 | 1.000 | 1.000 | 0.310 | 1.000 | 0.889 | 1.000 |  | 1.000 | 1.000 | 0.089 | 1.000 | 0.282 | 1.000 |  | 0.090 | 0.629 | 1.000 | 1.000 | 1.000 | 1.000 |
| OTU_382 | 1.000 | 0.058 | **0.003** | 1.000 | 0.952 | 1.000 |  | 1.000 | **< 0.001** | **< 0.001** | 1.000 | **0.015** | 1.000 |  | 1.000 | 1.000 | 0.251 | 0.702 | 0.065 | 1.000 |
| OTU_3820 | 1.000 | 0.782 | 1.000 | 1.000 | 1.000 | 1.000 |  | 1.000 | **0.003** | 0.306 | **0.003** | 0.285 | 1.000 |  | 1.000 | **0.007** | 0.670 | 0.564 | 1.000 | 1.000 |
| OTU_383 | 1.000 | **< 0.001** | 0.542 | **0.001** | 1.000 | 0.171 |  | 1.000 | **< 0.001** | **< 0.001** | **< 0.001** | 1.000 | 0.057 |  | 0.575 | **< 0.001** | **0.008** | 0.249 | 1.000 | 1.000 |
| OTU_3834 | 1.000 | 1.000 | 1.000 | 1.000 | 1.000 | 1.000 |  | 1.000 | 1.000 | 1.000 | 1.000 | 1.000 | 1.000 |  | 1.000 | 1.000 | 1.000 | 1.000 | 1.000 | 1.000 |
| OTU_384 | **0.048** | **< 0.001** | **< 0.001** | 1.000 | 1.000 | 1.000 |  | **0.048** | **< 0.001** | **< 0.001** | **< 0.001** | **0.006** | 1.000 |  | 1.000 | **< 0.001** | **< 0.001** | **0.001** | **< 0.001** | 1.000 |
| OTU_3847 | **< 0.001** | **< 0.001** | **0.016** | **0.003** | 1.000 | **< 0.001** |  | **< 0.001** | **< 0.001** | 1.000 | **< 0.001** | 1.000 | **< 0.001** |  | 0.311 | **< 0.001** | 1.000 | **0.024** | 1.000 | **< 0.001** |
| OTU_3848 | 1.000 | 1.000 | 1.000 | 1.000 | 1.000 | 1.000 |  | 1.000 | 0.309 | 1.000 | 0.572 | 1.000 | 1.000 |  | 1.000 | 1.000 | 1.000 | 1.000 | 1.000 | 1.000 |
| OTU_385 | 1.000 | 1.000 | **< 0.001** | 0.330 | **< 0.001** | **0.029** |  | 1.000 | 0.960 | **< 0.001** | 0.486 | **< 0.001** | **0.044** |  | 1.000 | 1.000 | **< 0.001** | 1.000 | **< 0.001** | **< 0.001** |
| OTU_386 | 1.000 | **0.017** | **0.013** | 1.000 | 1.000 | 1.000 |  | 1.000 | **0.023** | **0.030** | 0.782 | 0.848 | 1.000 |  | 1.000 | **0.038** | **0.032** | 1.000 | 1.000 | 1.000 |
| OTU_3874 | 1.000 | 1.000 | 1.000 | 1.000 | 1.000 | 1.000 |  | 1.000 | 0.220 | 0.113 | 1.000 | 1.000 | 1.000 |  | 1.000 | 1.000 | 1.000 | 1.000 | 1.000 | 1.000 |
| OTU_3876 | 1.000 | 1.000 | 1.000 | 0.917 | 0.063 | 1.000 |  | 1.000 | 1.000 | 1.000 | 1.000 | 1.000 | 1.000 |  | 1.000 | 1.000 | 1.000 | 1.000 | 1.000 | 1.000 |
| OTU_388 | 1.000 | 1.000 | 1.000 | 1.000 | 1.000 | 1.000 |  | 1.000 | 0.728 | 0.133 | 1.000 | 1.000 | 1.000 |  | 1.000 | 1.000 | 1.000 | 1.000 | 1.000 | 1.000 |
| OTU_3880 | 1.000 | 1.000 | 1.000 | 1.000 | 1.000 | 1.000 |  | 1.000 | 1.000 | 1.000 | 0.050 | 0.441 | 1.000 |  | 1.000 | 1.000 | 1.000 | 1.000 | 1.000 | 1.000 |
| OTU_389 | 1.000 | 1.000 | 1.000 | 1.000 | 0.604 | 0.985 |  | 1.000 | 1.000 | **0.018** | 1.000 | 0.108 | 1.000 |  | 1.000 | 1.000 | 0.144 | 1.000 | 0.137 | 1.000 |
| OTU_39 | **0.026** | **< 0.001** | **0.018** | **< 0.001** | 1.000 | **< 0.001** |  | **0.026** | **< 0.001** | **< 0.001** | **< 0.001** | 1.000 | **< 0.001** |  | 0.133 | **< 0.001** | 0.153 | **< 0.001** | 1.000 | **< 0.001** |
| OTU_390 | 1.000 | 1.000 | 1.000 | 1.000 | 1.000 | 1.000 |  | 1.000 | 0.079 | **0.043** | 1.000 | 1.000 | 1.000 |  | 1.000 | 1.000 | 1.000 | 1.000 | 1.000 | 1.000 |
| OTU_3904 | 0.063 | **0.004** | 0.876 | 1.000 | 1.000 | 1.000 |  | 0.063 | **0.016** | 1.000 | 1.000 | 1.000 | 0.289 |  | 0.960 | **0.029** | 0.742 | 1.000 | 1.000 | 1.000 |
| OTU_391 | 1.000 | 1.000 | **0.048** | 1.000 | **< 0.001** | **< 0.001** |  | 1.000 | 1.000 | **< 0.001** | 1.000 | **< 0.001** | **< 0.001** |  | 1.000 | 1.000 | 1.000 | 1.000 | 1.000 | 1.000 |
| OTU_3916 | 0.924 | 1.000 | 1.000 | 1.000 | 0.289 | 1.000 |  | 0.924 | 1.000 | 0.051 | 1.000 | 0.063 | 0.106 |  | 1.000 | 1.000 | 1.000 | 1.000 | 1.000 | 1.000 |
| OTU_392 | 1.000 | 0.410 | 0.272 | 0.378 | 0.194 | 1.000 |  | 1.000 | 1.000 | 1.000 | 0.606 | 0.783 | 1.000 |  | 1.000 | 0.320 | **0.014** | 1.000 | 1.000 | 1.000 |
| OTU_3926 | 1.000 | 1.000 | 1.000 | 1.000 | 1.000 | 1.000 |  | 1.000 | 0.371 | 1.000 | 1.000 | 1.000 | 1.000 |  | 1.000 | 1.000 | 1.000 | 1.000 | 1.000 | 1.000 |
| OTU_393 | 1.000 | 1.000 | 0.251 | 1.000 | 0.363 | 1.000 |  | 1.000 | 0.213 | 0.596 | 0.097 | 0.235 | 1.000 |  | 1.000 | 1.000 | 1.000 | 0.207 | **0.015** | 1.000 |
| OTU_3939 | 1.000 | 1.000 | 0.641 | 1.000 | 1.000 | 1.000 |  | 1.000 | 1.000 | 1.000 | 1.000 | 1.000 | 1.000 |  | 1.000 | 1.000 | 1.000 | 1.000 | 1.000 | 1.000 |
| OTU_394 | 1.000 | 1.000 | 1.000 | 1.000 | 0.130 | 1.000 |  | 1.000 | 1.000 | 1.000 | 1.000 | 1.000 | 0.970 |  | 1.000 | 1.000 | 1.000 | 1.000 | 0.159 | 0.162 |
| OTU_3943 | 1.000 | 1.000 | 1.000 | 1.000 | 1.000 | 1.000 |  | 1.000 | 1.000 | **0.035** | 1.000 | 1.000 | 1.000 |  | 1.000 | 1.000 | 1.000 | 1.000 | 1.000 | 1.000 |
| OTU_3944 | 0.080 | 0.528 | 1.000 | 1.000 | **0.004** | 0.138 |  | 0.080 | 1.000 | 1.000 | **0.009** | **< 0.001** | 1.000 |  | **< 0.001** | 1.000 | 1.000 | **< 0.001** | **< 0.001** | 1.000 |
| OTU_395 | 1.000 | 1.000 | **0.032** | 1.000 | 1.000 | 1.000 |  | 1.000 | 0.704 | **0.046** | 1.000 | 0.732 | 1.000 |  | 1.000 | 1.000 | 1.000 | 1.000 | 1.000 | 1.000 |
| OTU_396 | 1.000 | 1.000 | 1.000 | 1.000 | 1.000 | 1.000 |  | 1.000 | 1.000 | 1.000 | 1.000 | 1.000 | 1.000 |  | 1.000 | 1.000 | 1.000 | 1.000 | 1.000 | 1.000 |
| OTU_3960 | 1.000 | 1.000 | 0.321 | 1.000 | 0.717 | 1.000 |  | 1.000 | 0.415 | 0.244 | 1.000 | 1.000 | 1.000 |  | 1.000 | 1.000 | 1.000 | 1.000 | 1.000 | 1.000 |
| OTU_397 | 1.000 | 1.000 | 1.000 | 0.312 | 0.150 | 1.000 |  | 1.000 | 1.000 | 0.733 | 1.000 | 0.717 | 1.000 |  | 1.000 | 1.000 | 1.000 | 1.000 | 0.067 | 1.000 |
| OTU_398 | 1.000 | **< 0.001** | **0.006** | **< 0.001** | **< 0.001** | 1.000 |  | 1.000 | 1.000 | **0.012** | **0.020** | **< 0.001** | 1.000 |  | 0.063 | **< 0.001** | **< 0.001** | **0.005** | 1.000 | 0.727 |
| OTU_399 | 1.000 | **0.007** | **0.003** | 0.127 | **0.047** | 1.000 |  | 1.000 | **< 0.001** | **< 0.001** | **0.022** | **< 0.001** | 1.000 |  | 1.000 | **0.030** | **0.004** | 1.000 | 1.000 | 1.000 |
| OTU_3998 | 0.177 | **< 0.001** | **< 0.001** | **< 0.001** | **< 0.001** | 1.000 |  | 0.177 | **< 0.001** | **< 0.001** | **< 0.001** | **< 0.001** | 1.000 |  | **< 0.001** | **< 0.001** | **< 0.001** | **0.033** | 0.117 | 1.000 |
| OTU_4 | 1.000 | **< 0.001** | **0.027** | **< 0.001** | 1.000 | 0.166 |  | 1.000 | **< 0.001** | **< 0.001** | **< 0.001** | **< 0.001** | 1.000 |  | 0.945 | **< 0.001** | **< 0.001** | **0.026** | 0.888 | 1.000 |
| OTU_40 | **< 0.001** | **< 0.001** | **< 0.001** | 1.000 | 1.000 | 1.000 |  | **< 0.001** | **< 0.001** | **< 0.001** | 1.000 | 1.000 | 1.000 |  | 0.960 | **< 0.001** | **< 0.001** | 0.693 | 0.058 | 1.000 |
| OTU_400 | 1.000 | 1.000 | 1.000 | 1.000 | 1.000 | 1.000 |  | 1.000 | 1.000 | 1.000 | 1.000 | 1.000 | 1.000 |  | 1.000 | 1.000 | 1.000 | 1.000 | 1.000 | 1.000 |
| OTU_4007 | 0.122 | 1.000 | **0.008** | 1.000 | 1.000 | 1.000 |  | 0.122 | 1.000 | 1.000 | 1.000 | 1.000 | 1.000 |  | 1.000 | 1.000 | 1.000 | 1.000 | 1.000 | 1.000 |
| OTU_401 | 1.000 | 0.244 | 0.479 | 1.000 | 1.000 | 1.000 |  | 1.000 | **0.032** | 0.094 | 0.653 | 1.000 | 1.000 |  | 1.000 | 0.057 | 0.068 | **0.019** | **0.015** | 1.000 |
| OTU_4015 | 1.000 | **< 0.001** | **0.004** | **< 0.001** | 1.000 | 0.248 |  | 1.000 | **< 0.001** | **< 0.001** | **0.004** | 0.377 | 1.000 |  | 0.085 | **< 0.001** | **< 0.001** | 0.111 | 0.781 | 1.000 |
| OTU_4017 | 1.000 | 0.053 | 0.290 | **< 0.001** | **0.002** | 1.000 |  | 1.000 | **< 0.001** | **< 0.001** | 1.000 | 1.000 | 1.000 |  | 1.000 | 1.000 | **0.013** | 1.000 | 0.469 | 1.000 |
| OTU_402 | **0.006** | **< 0.001** | **< 0.001** | **0.042** | 0.503 | 1.000 |  | **0.006** | **< 0.001** | **< 0.001** | **0.015** | 0.343 | 1.000 |  | **0.022** | **< 0.001** | **< 0.001** | 0.065 | 1.000 | 1.000 |
| OTU_404 | **0.009** | **< 0.001** | **< 0.001** | 0.308 | 1.000 | 1.000 |  | **0.009** | **< 0.001** | **0.006** | 1.000 | 1.000 | 1.000 |  | **0.004** | **< 0.001** | **0.002** | 1.000 | 1.000 | 1.000 |
| OTU_4040 | 1.000 | 1.000 | 1.000 | 1.000 | 1.000 | 1.000 |  | 1.000 | 0.174 | 0.564 | 1.000 | 1.000 | 1.000 |  | 1.000 | 1.000 | 1.000 | 1.000 | 1.000 | 1.000 |
| OTU_4044 | 1.000 | 0.302 | 1.000 | 1.000 | 1.000 | 1.000 |  | 1.000 | **0.003** | 0.090 | 1.000 | 1.000 | 1.000 |  | 1.000 | **0.013** | 0.557 | 1.000 | 1.000 | 1.000 |
| OTU_4053 | 1.000 | 1.000 | 0.177 | 1.000 | **0.017** | 1.000 |  | 1.000 | 1.000 | **0.002** | 1.000 | 0.323 | 0.404 |  | 1.000 | 0.769 | **0.009** | 1.000 | 0.104 | 1.000 |
| OTU_406 | 1.000 | 1.000 | **< 0.001** | 1.000 | **< 0.001** | **0.020** |  | 1.000 | 1.000 | **< 0.001** | 1.000 | 0.122 | **0.019** |  | 1.000 | 1.000 | **< 0.001** | 1.000 | **< 0.001** | **< 0.001** |
| OTU_407 | 1.000 | 0.305 | 0.684 | 1.000 | 1.000 | 1.000 |  | 1.000 | 0.373 | 1.000 | **< 0.001** | **< 0.001** | 1.000 |  | 1.000 | 0.257 | 1.000 | 0.628 | 1.000 | 1.000 |
| OTU_408 | 1.000 | 0.912 | 1.000 | 1.000 | 1.000 | 1.000 |  | 1.000 | **0.008** | 1.000 | 1.000 | 1.000 | 0.745 |  | 1.000 | 1.000 | 1.000 | 1.000 | 1.000 | 1.000 |
| OTU_409 | 1.000 | 0.061 | 0.055 | 1.000 | 1.000 | 1.000 |  | 1.000 | **< 0.001** | **< 0.001** | **< 0.001** | **< 0.001** | 1.000 |  | 1.000 | 0.216 | 1.000 | 1.000 | 1.000 | 1.000 |
| OTU_4094 | 1.000 | 0.157 | 1.000 | 1.000 | 1.000 | 1.000 |  | 1.000 | 1.000 | 1.000 | 1.000 | 1.000 | 1.000 |  | 1.000 | 0.121 | 0.208 | **0.011** | **0.014** | 1.000 |
| OTU_4096 | 1.000 | 1.000 | **0.005** | 1.000 | 1.000 | 1.000 |  | 1.000 | 1.000 | 0.621 | 1.000 | 1.000 | 1.000 |  | 1.000 | 1.000 | **0.007** | 1.000 | 1.000 | 0.334 |
| OTU_4099 | 1.000 | 0.590 | 1.000 | 1.000 | 1.000 | 0.257 |  | 1.000 | **0.043** | 1.000 | 1.000 | 0.050 | **0.033** |  | 1.000 | 0.361 | 1.000 | 1.000 | 1.000 | 1.000 |
| OTU_41 | 1.000 | **< 0.001** | **< 0.001** | **0.007** | **< 0.001** | 1.000 |  | 1.000 | **< 0.001** | **< 0.001** | **< 0.001** | **< 0.001** | 1.000 |  | 1.000 | **0.022** | **0.017** | **0.003** | **0.001** | 1.000 |
| OTU_410 | 1.000 | 1.000 | 1.000 | 1.000 | 1.000 | 1.000 |  | 1.000 | **0.049** | 1.000 | 1.000 | 0.313 | 1.000 |  | 1.000 | 1.000 | 1.000 | 1.000 | 1.000 | 1.000 |
| OTU_4104 | 1.000 | **< 0.001** | 1.000 | **< 0.001** | 0.231 | **< 0.001** |  | 1.000 | **0.011** | 0.099 | **< 0.001** | 1.000 | **< 0.001** |  | 1.000 | **0.005** | 1.000 | 0.114 | 1.000 | **0.004** |
| OTU_4106 | 1.000 | 1.000 | 1.000 | 1.000 | 1.000 | 1.000 |  | 1.000 | 0.447 | 1.000 | 1.000 | 1.000 | 1.000 |  | 1.000 | 1.000 | 0.890 | 1.000 | 1.000 | 1.000 |
| OTU_411 | 0.267 | **< 0.001** | **< 0.001** | 0.186 | 0.356 | 1.000 |  | 0.267 | **< 0.001** | **< 0.001** | **< 0.001** | **0.001** | 0.341 |  | 1.000 | **0.038** | **0.037** | 0.500 | 0.391 | 1.000 |
| OTU_4116 | 1.000 | 1.000 | **< 0.001** | 1.000 | **< 0.001** | **< 0.001** |  | 1.000 | 1.000 | **< 0.001** | 1.000 | **< 0.001** | **< 0.001** |  | 1.000 | 1.000 | **< 0.001** | 1.000 | **< 0.001** | **< 0.001** |
| OTU_4122 | 1.000 | **< 0.001** | **< 0.001** | **< 0.001** | **< 0.001** | 1.000 |  | 1.000 | **< 0.001** | **< 0.001** | **< 0.001** | **< 0.001** | 1.000 |  | 1.000 | **< 0.001** | **< 0.001** | **0.004** | **< 0.001** | 1.000 |
| OTU_413 | 1.000 | 1.000 | 1.000 | 0.284 | 1.000 | 1.000 |  | 1.000 | 1.000 | 1.000 | 1.000 | 1.000 | 1.000 |  | 1.000 | 0.205 | 1.000 | 1.000 | 1.000 | 1.000 |
| OTU_4130 | 1.000 | 1.000 | 1.000 | 1.000 | 1.000 | 1.000 |  | 1.000 | 0.953 | 1.000 | 1.000 | 1.000 | 1.000 |  | 0.310 | 1.000 | 1.000 | 1.000 | 0.905 | 1.000 |
| OTU_414 | 1.000 | **< 0.001** | **< 0.001** | **< 0.001** | **< 0.001** | 0.540 |  | 1.000 | **< 0.001** | **< 0.001** | 0.091 | **< 0.001** | 0.190 |  | 1.000 | **< 0.001** | **< 0.001** | **< 0.001** | **< 0.001** | 1.000 |
| OTU_415 | 1.000 | 0.063 | 1.000 | 0.560 | 1.000 | 1.000 |  | 1.000 | 0.871 | 1.000 | 1.000 | 1.000 | 1.000 |  | 1.000 | 1.000 | 1.000 | 1.000 | 1.000 | 1.000 |
| OTU_4154 | 1.000 | **0.013** | 1.000 | 0.360 | 1.000 | 0.295 |  | 1.000 | **0.010** | 0.273 | 1.000 | 1.000 | 1.000 |  | 1.000 | 0.923 | 1.000 | 1.000 | 1.000 | 1.000 |
| OTU_416 | 1.000 | 1.000 | 0.853 | 1.000 | 0.342 | 1.000 |  | 1.000 | **0.002** | **< 0.001** | 0.903 | 0.064 | 1.000 |  | 0.916 | 1.000 | 1.000 | 1.000 | 0.404 | 1.000 |
| OTU_417 | 1.000 | 0.871 | **< 0.001** | 0.483 | **< 0.001** | **< 0.001** |  | 1.000 | 0.064 | **< 0.001** | **0.008** | **< 0.001** | **< 0.001** |  | 1.000 | **< 0.001** | **< 0.001** | **0.036** | **< 0.001** | **0.003** |
| OTU_418 | 1.000 | 1.000 | 0.052 | 1.000 | 1.000 | 1.000 |  | 1.000 | **0.021** | **0.002** | **< 0.001** | **< 0.001** | 1.000 |  | 1.000 | 0.273 | **0.041** | 0.715 | 0.084 | 1.000 |
| OTU_4184 | 1.000 | 1.000 | 1.000 | 1.000 | 1.000 | 1.000 |  | 1.000 | 0.125 | 0.120 | 0.181 | 0.138 | 1.000 |  | **< 0.001** | **< 0.001** | **< 0.001** | **0.030** | 1.000 | 0.131 |
| OTU_419 | 1.000 | **< 0.001** | **< 0.001** | 0.059 | **0.002** | 1.000 |  | 1.000 | 1.000 | 0.252 | **0.002** | **< 0.001** | 1.000 |  | 1.000 | **< 0.001** | **< 0.001** | 0.362 | **0.006** | 1.000 |
| OTU_4195 | 1.000 | 1.000 | 1.000 | 1.000 | 1.000 | 1.000 |  | 1.000 | 1.000 | 1.000 | 1.000 | 1.000 | 1.000 |  | 1.000 | 1.000 | 1.000 | 1.000 | 1.000 | 1.000 |
| OTU_42 | 1.000 | **< 0.001** | **< 0.001** | **< 0.001** | **< 0.001** | 1.000 |  | 1.000 | **0.004** | **< 0.001** | **< 0.001** | **< 0.001** | 0.534 |  | 1.000 | **< 0.001** | **< 0.001** | **< 0.001** | **< 0.001** | 1.000 |
| OTU_420 | 1.000 | 1.000 | 0.170 | 1.000 | **< 0.001** | **0.029** |  | 1.000 | 1.000 | 0.683 | 0.755 | **< 0.001** | **0.027** |  | 1.000 | 1.000 | 1.000 | 1.000 | 0.545 | 1.000 |
| OTU_421 | 1.000 | **0.003** | 1.000 | 0.541 | 1.000 | 0.108 |  | 1.000 | **0.009** | 1.000 | 1.000 | 1.000 | 0.871 |  | 1.000 | **0.044** | 0.232 | 1.000 | 1.000 | 1.000 |
| OTU_422 | 1.000 | 1.000 | **0.026** | 1.000 | 0.083 | 0.055 |  | 1.000 | 1.000 | **0.037** | 1.000 | **0.015** | 0.642 |  | 1.000 | 0.088 | **0.010** | 1.000 | 1.000 | 1.000 |
| OTU_4224 | 1.000 | 1.000 | 0.064 | 1.000 | 0.076 | 0.950 |  | 1.000 | 1.000 | 0.055 | 1.000 | 0.365 | 0.088 |  | 1.000 | 1.000 | 0.152 | 1.000 | 0.539 | 1.000 |
| OTU_423 | 1.000 | 1.000 | 0.168 | 1.000 | 1.000 | 0.249 |  | 1.000 | 1.000 | 1.000 | 0.683 | 0.097 | 1.000 |  | 1.000 | 1.000 | 0.277 | 1.000 | 0.777 | 1.000 |
| OTU_4233 | 1.000 | 1.000 | 1.000 | 1.000 | 1.000 | 1.000 |  | 1.000 | 1.000 | 1.000 | 1.000 | 1.000 | 1.000 |  | 1.000 | 1.000 | 1.000 | 0.393 | 1.000 | 1.000 |
| OTU_424 | **0.003** | 1.000 | 1.000 | 0.127 | **0.002** | 1.000 |  | **0.003** | 1.000 | 0.161 | 1.000 | **< 0.001** | 0.296 |  | 1.000 | 1.000 | 1.000 | 1.000 | 1.000 | 1.000 |
| OTU_4248 | 1.000 | **< 0.001** | **< 0.001** | **< 0.001** | **< 0.001** | 1.000 |  | 1.000 | **< 0.001** | **< 0.001** | **< 0.001** | **< 0.001** | 1.000 |  | 1.000 | **< 0.001** | **< 0.001** | **< 0.001** | **< 0.001** | **< 0.001** |
| OTU_4249 | 1.000 | 1.000 | 1.000 | 1.000 | 1.000 | 1.000 |  | 1.000 | 0.381 | 1.000 | 0.281 | 1.000 | **0.027** |  | 1.000 | **0.034** | 1.000 | 1.000 | 1.000 | 0.511 |
| OTU_425 | 1.000 | **< 0.001** | **< 0.001** | 0.247 | **< 0.001** | 1.000 |  | 1.000 | **< 0.001** | **< 0.001** | 1.000 | **0.001** | 0.340 |  | 0.117 | **0.015** | **< 0.001** | 1.000 | **< 0.001** | **0.003** |
| OTU_426 | **0.004** | **0.047** | **0.011** | 1.000 | 1.000 | 1.000 |  | **0.004** | 1.000 | 1.000 | 0.194 | 0.322 | 1.000 |  | **0.005** | 0.180 | 0.119 | 1.000 | 1.000 | 1.000 |
| OTU_427 | 1.000 | 1.000 | 1.000 | 1.000 | **0.003** | 0.343 |  | 1.000 | 1.000 | 0.446 | 1.000 | 0.218 | 1.000 |  | 1.000 | 1.000 | **0.025** | 1.000 | **0.044** | 0.950 |
| OTU_4274 | 1.000 | 1.000 | 1.000 | 1.000 | 0.067 | 1.000 |  | 1.000 | 1.000 | 0.084 | 1.000 | **0.004** | 1.000 |  | 1.000 | 1.000 | 0.057 | 0.867 | **0.027** | 1.000 |
| OTU_428 | 1.000 | 1.000 | **0.005** | 1.000 | **0.031** | 0.909 |  | 1.000 | 1.000 | 0.617 | 1.000 | **0.008** | 0.650 |  | 0.294 | 1.000 | 1.000 | 1.000 | **< 0.001** | 0.516 |
| OTU_429 | 1.000 | **< 0.001** | **< 0.001** | **< 0.001** | **< 0.001** | 1.000 |  | 1.000 | **0.001** | **< 0.001** | 0.126 | **0.003** | 1.000 |  | 1.000 | **0.019** | **< 0.001** | 0.100 | **0.003** | 1.000 |
| OTU_43 | 1.000 | **0.003** | **0.003** | 1.000 | 1.000 | 1.000 |  | 1.000 | **0.041** | 0.101 | 1.000 | 1.000 | 1.000 |  | 1.000 | **< 0.001** | **0.002** | 0.731 | 1.000 | 1.000 |
| OTU_430 | 1.000 | 1.000 | 1.000 | 1.000 | 1.000 | 1.000 |  | 1.000 | 1.000 | 1.000 | 0.070 | **0.046** | 1.000 |  | 1.000 | 1.000 | 1.000 | 1.000 | 1.000 | 1.000 |
| OTU_431 | 0.396 | **0.004** | **< 0.001** | 1.000 | **< 0.001** | 0.230 |  | 0.396 | **0.022** | **< 0.001** | 0.111 | **< 0.001** | 1.000 |  | 1.000 | **0.003** | **0.001** | 0.798 | 0.420 | 1.000 |
| OTU_4311 | 0.293 | 0.338 | 1.000 | 1.000 | 1.000 | 1.000 |  | 0.293 | 0.984 | 1.000 | 1.000 | 1.000 | 1.000 |  | 1.000 | 1.000 | 1.000 | 1.000 | 1.000 | 1.000 |
| OTU_432 | **< 0.001** | **< 0.001** | **< 0.001** | 1.000 | 1.000 | 1.000 |  | **< 0.001** | **< 0.001** | **< 0.001** | 1.000 | 1.000 | 1.000 |  | 0.063 | **< 0.001** | **< 0.001** | 0.636 | 1.000 | 1.000 |
| OTU_4325 | **< 0.001** | **< 0.001** | 0.740 | 1.000 | **< 0.001** | 0.101 |  | **< 0.001** | 1.000 | 1.000 | 0.257 | **< 0.001** | 0.140 |  | **0.002** | 0.106 | 1.000 | 1.000 | **< 0.001** | 0.070 |
| OTU_4337 | **< 0.001** | **0.006** | **0.002** | 1.000 | 1.000 | 1.000 |  | **< 0.001** | 0.226 | 1.000 | 1.000 | 1.000 | 0.857 |  | 1.000 | 0.094 | 1.000 | 1.000 | 1.000 | 0.880 |
| OTU_434 | 1.000 | 1.000 | 1.000 | 1.000 | 1.000 | 1.000 |  | 1.000 | 1.000 | 1.000 | 1.000 | 1.000 | 1.000 |  | 1.000 | 1.000 | 1.000 | 1.000 | 1.000 | 1.000 |
| OTU_435 | 1.000 | 1.000 | 1.000 | 1.000 | 1.000 | 1.000 |  | 1.000 | 0.124 | 1.000 | 1.000 | 0.718 | 1.000 |  | 1.000 | 1.000 | 1.000 | 1.000 | 1.000 | 1.000 |
| OTU_4354 | 1.000 | 1.000 | 0.073 | 0.051 | 1.000 | **0.002** |  | 1.000 | 1.000 | **< 0.001** | 1.000 | **< 0.001** | **< 0.001** |  | 1.000 | 1.000 | **< 0.001** | 1.000 | **< 0.001** | **< 0.001** |
| OTU_4356 | 1.000 | 0.749 | 0.792 | 0.829 | 0.723 | 1.000 |  | 1.000 | **0.046** | 0.308 | 1.000 | 1.000 | 1.000 |  | 1.000 | **0.028** | **0.037** | 1.000 | 1.000 | 1.000 |
| OTU_4357 | 1.000 | 1.000 | 0.139 | 1.000 | **0.005** | 1.000 |  | 1.000 | 1.000 | 1.000 | 1.000 | **0.002** | 1.000 |  | 1.000 | 1.000 | 1.000 | 1.000 | 1.000 | 1.000 |
| OTU_436 | **0.011** | **< 0.001** | **< 0.001** | 1.000 | 1.000 | 1.000 |  | **0.011** | **0.013** | **< 0.001** | 1.000 | 1.000 | 1.000 |  | 1.000 | **0.006** | **< 0.001** | 1.000 | **0.041** | 1.000 |
| OTU_4360 | 1.000 | 1.000 | 0.414 | 1.000 | 0.739 | 1.000 |  | 1.000 | 1.000 | 0.217 | 0.724 | **0.011** | 1.000 |  | 1.000 | 1.000 | 1.000 | 1.000 | 1.000 | 1.000 |
| OTU_437 | **< 0.001** | **< 0.001** | 1.000 | 1.000 | **< 0.001** | **< 0.001** |  | **< 0.001** | 1.000 | **0.003** | 1.000 | **< 0.001** | **< 0.001** |  | **< 0.001** | **< 0.001** | 0.914 | 1.000 | **< 0.001** | **< 0.001** |
| OTU_4387 | 1.000 | 1.000 | 1.000 | 1.000 | 1.000 | 1.000 |  | 1.000 | 1.000 | 1.000 | 1.000 | 1.000 | 1.000 |  | 1.000 | 0.790 | 1.000 | 1.000 | 1.000 | 1.000 |
| OTU_439 | 1.000 | 1.000 | 1.000 | 1.000 | 1.000 | 1.000 |  | 1.000 | 1.000 | 0.935 | 1.000 | 1.000 | 1.000 |  | 1.000 | 1.000 | 1.000 | 1.000 | 1.000 | 1.000 |
| OTU_4390 | 1.000 | 1.000 | 0.078 | 1.000 | **0.002** | **0.026** |  | 1.000 | 1.000 | 1.000 | 1.000 | 0.092 | 1.000 |  | 1.000 | 1.000 | **0.003** | 1.000 | **< 0.001** | 0.110 |
| OTU_4396 | 1.000 | 1.000 | 1.000 | 1.000 | 1.000 | 1.000 |  | 1.000 | 1.000 | 1.000 | 1.000 | 1.000 | 1.000 |  | 1.000 | 0.191 | **0.041** | 1.000 | 1.000 | 1.000 |
| OTU_4398 | 1.000 | 1.000 | 1.000 | 1.000 | 1.000 | 1.000 |  | 1.000 | **0.019** | 0.331 | 1.000 | 1.000 | 1.000 |  | 1.000 | 1.000 | 1.000 | 1.000 | 1.000 | 1.000 |
| OTU_44 | 1.000 | 0.670 | 1.000 | 0.169 | 1.000 | 0.108 |  | 1.000 | 1.000 | 1.000 | 1.000 | 1.000 | 1.000 |  | 0.526 | 1.000 | 1.000 | **< 0.001** | **0.006** | 1.000 |
| OTU_440 | **< 0.001** | **< 0.001** | **0.007** | 1.000 | 1.000 | 1.000 |  | **< 0.001** | **0.007** | **0.018** | 0.584 | 1.000 | 1.000 |  | 1.000 | 0.334 | 0.226 | 1.000 | 1.000 | 1.000 |
| OTU_4401 | 0.141 | 1.000 | 1.000 | 1.000 | 0.051 | 1.000 |  | 0.141 | 1.000 | 0.349 | 1.000 | 0.407 | 0.642 |  | 1.000 | 1.000 | 0.071 | 1.000 | 0.079 | 1.000 |
| OTU_4404 | 1.000 | 0.123 | **0.018** | 0.482 | 0.059 | 1.000 |  | 1.000 | **< 0.001** | **< 0.001** | 0.273 | **0.036** | 1.000 |  | 1.000 | **0.028** | **0.001** | 1.000 | 0.268 | 1.000 |
| OTU_4406 | 1.000 | 1.000 | 1.000 | 1.000 | 1.000 | 1.000 |  | 1.000 | 1.000 | 1.000 | 1.000 | 1.000 | 1.000 |  | 1.000 | 1.000 | 1.000 | 1.000 | 0.705 | 0.107 |
| OTU_441 | 1.000 | 0.919 | 1.000 | 1.000 | 1.000 | 1.000 |  | 1.000 | **0.028** | 1.000 | 0.652 | 1.000 | 0.349 |  | 1.000 | 1.000 | 1.000 | 1.000 | 1.000 | 1.000 |
| OTU_4415 | **0.003** | **0.001** | **0.001** | 1.000 | 1.000 | 1.000 |  | **0.003** | **0.007** | **0.002** | 1.000 | 1.000 | 1.000 |  | 0.380 | 0.189 | 0.123 | 1.000 | 1.000 | 1.000 |
| OTU_442 | 1.000 | 1.000 | **0.005** | 1.000 | **0.005** | 0.337 |  | 1.000 | **< 0.001** | **< 0.001** | **0.005** | **< 0.001** | 1.000 |  | 1.000 | 1.000 | **0.023** | **0.030** | **< 0.001** | 1.000 |
| OTU_4422 | 0.144 | **0.004** | **< 0.001** | 1.000 | 1.000 | 1.000 |  | 0.144 | **0.002** | **< 0.001** | 0.289 | **< 0.001** | 1.000 |  | 0.620 | **0.029** | **< 0.001** | 1.000 | 0.220 | 1.000 |
| OTU_443 | 1.000 | **0.003** | **0.003** | 0.416 | 0.270 | 1.000 |  | 1.000 | **< 0.001** | **0.002** | 1.000 | 1.000 | 1.000 |  | 1.000 | **0.011** | 0.171 | 0.119 | 0.931 | 1.000 |
| OTU_4434 | 1.000 | 1.000 | **< 0.001** | 1.000 | **< 0.001** | **0.026** |  | 1.000 | 1.000 | 0.054 | 1.000 | 0.064 | 0.107 |  | 1.000 | 1.000 | 1.000 | 1.000 | 1.000 | 1.000 |
| OTU_444 | 1.000 | 0.052 | 0.313 | 1.000 | 1.000 | 1.000 |  | 1.000 | 1.000 | 1.000 | 1.000 | 1.000 | 1.000 |  | 1.000 | 1.000 | 1.000 | 0.727 | 0.300 | 1.000 |
| OTU_4440 | **< 0.001** | **< 0.001** | **< 0.001** | **< 0.001** | **< 0.001** | 0.537 |  | **< 0.001** | **< 0.001** | **< 0.001** | **0.007** | **0.024** | 1.000 |  | **< 0.001** | **< 0.001** | **< 0.001** | **< 0.001** | **< 0.001** | 1.000 |
| OTU_4445 | 1.000 | 0.314 | 1.000 | 1.000 | 1.000 | 1.000 |  | 1.000 | 1.000 | 1.000 | 1.000 | 1.000 | 1.000 |  | 1.000 | 1.000 | 1.000 | 1.000 | 1.000 | 1.000 |
| OTU_445 | 1.000 | 0.992 | 1.000 | 1.000 | 1.000 | 1.000 |  | 1.000 | 1.000 | 1.000 | 1.000 | 1.000 | 1.000 |  | 1.000 | 1.000 | 1.000 | 1.000 | **0.008** | **0.022** |
| OTU_446 | 0.924 | **< 0.001** | 1.000 | 0.619 | 1.000 | 0.080 |  | 0.924 | **0.032** | 0.145 | 0.362 | 1.000 | 1.000 |  | 1.000 | **< 0.001** | 0.066 | **< 0.001** | 1.000 | 0.077 |
| OTU_4460 | **0.033** | 0.602 | 0.108 | 1.000 | 1.000 | 1.000 |  | **0.033** | 0.915 | 1.000 | 1.000 | 1.000 | 1.000 |  | 1.000 | 1.000 | 1.000 | 1.000 | 1.000 | 1.000 |
| OTU_447 | 1.000 | 1.000 | 0.083 | 1.000 | 0.135 | 1.000 |  | 1.000 | **0.021** | **0.004** | 0.164 | **0.035** | 1.000 |  | 1.000 | 0.139 | 0.339 | 1.000 | 1.000 | 1.000 |
| OTU_448 | 1.000 | 1.000 | 1.000 | 1.000 | 1.000 | 1.000 |  | 1.000 | 0.715 | 0.295 | 1.000 | 1.000 | 1.000 |  | 1.000 | 1.000 | 0.468 | 1.000 | 0.378 | 1.000 |
| OTU_449 | 1.000 | 1.000 | 1.000 | 1.000 | 1.000 | 1.000 |  | 1.000 | 0.230 | 1.000 | 0.829 | 1.000 | 1.000 |  | 1.000 | 0.662 | 1.000 | 1.000 | 1.000 | 1.000 |
| OTU_4492 | 1.000 | 0.073 | **0.001** | **0.003** | **< 0.001** | 1.000 |  | 1.000 | **< 0.001** | **< 0.001** | **< 0.001** | **< 0.001** | 1.000 |  | 1.000 | 0.683 | **0.025** | 1.000 | 0.122 | 1.000 |
| OTU_45 | **< 0.001** | **< 0.001** | **< 0.001** | **0.010** | 1.000 | 0.272 |  | **< 0.001** | **< 0.001** | **< 0.001** | 0.254 | 1.000 | 0.827 |  | **< 0.001** | **< 0.001** | **< 0.001** | **0.015** | 1.000 | 0.159 |
| OTU_450 | 1.000 | 1.000 | 1.000 | 1.000 | 1.000 | 1.000 |  | 1.000 | 1.000 | 0.577 | 0.340 | **< 0.001** | 0.770 |  | 0.311 | 0.430 | **0.012** | 1.000 | 1.000 | 1.000 |
| OTU_4500 | 1.000 | **0.001** | 0.592 | **< 0.001** | **0.010** | 1.000 |  | 1.000 | **< 0.001** | **< 0.001** | **< 0.001** | **< 0.001** | 1.000 |  | 1.000 | **< 0.001** | **0.028** | **< 0.001** | **0.006** | 1.000 |
| OTU_451 | 1.000 | 0.857 | 1.000 | 0.378 | 1.000 | 0.756 |  | 1.000 | 1.000 | 1.000 | 1.000 | 1.000 | 1.000 |  | 1.000 | 1.000 | 1.000 | 1.000 | 1.000 | 1.000 |
| OTU_4516 | 1.000 | 0.721 | 0.065 | 1.000 | 1.000 | 1.000 |  | 1.000 | 1.000 | 1.000 | 1.000 | 1.000 | 1.000 |  | **0.034** | 0.261 | **0.047** | 1.000 | 1.000 | 1.000 |
| OTU_452 | 1.000 | 1.000 | 1.000 | 1.000 | 1.000 | 1.000 |  | 1.000 | 1.000 | 0.225 | 0.138 | 1.000 | 1.000 |  | 1.000 | **0.011** | 1.000 | 1.000 | 1.000 | 1.000 |
| OTU_453 | 0.381 | **< 0.001** | **< 0.001** | **0.011** | 0.338 | 1.000 |  | 0.381 | **< 0.001** | **< 0.001** | **0.002** | **0.006** | 1.000 |  | 1.000 | **< 0.001** | **0.008** | **0.001** | 0.065 | 1.000 |
| OTU_454 | 1.000 | 1.000 | 1.000 | 1.000 | 0.905 | 1.000 |  | 1.000 | 1.000 | 1.000 | 1.000 | 1.000 | 1.000 |  | 1.000 | **< 0.001** | **< 0.001** | **0.015** | **< 0.001** | 1.000 |
| OTU_4548 | 1.000 | 1.000 | **0.002** | 0.340 | **< 0.001** | 0.687 |  | 1.000 | 0.953 | **< 0.001** | 1.000 | **< 0.001** | **0.003** |  | 1.000 | 1.000 | **< 0.001** | 1.000 | **< 0.001** | **< 0.001** |
| OTU_455 | 1.000 | 0.166 | 0.228 | 0.378 | 0.170 | **< 0.001** |  | 1.000 | 0.481 | 0.714 | 1.000 | 0.199 | **< 0.001** |  | 1.000 | 0.073 | 0.977 | **0.025** | 1.000 | **< 0.001** |
| OTU_4558 | 0.639 | 1.000 | **< 0.001** | 1.000 | 0.113 | **0.050** |  | 0.639 | 0.254 | **< 0.001** | 1.000 | **0.017** | **< 0.001** |  | 1.000 | 1.000 | 1.000 | 1.000 | 1.000 | 1.000 |
| OTU_456 | 1.000 | 0.433 | 0.442 | 1.000 | 1.000 | 1.000 |  | 1.000 | **< 0.001** | **< 0.001** | 1.000 | 1.000 | 1.000 |  | 1.000 | 0.377 | **0.015** | 1.000 | 0.888 | 1.000 |
| OTU_4564 | 0.073 | **0.044** | 1.000 | 1.000 | 0.074 | 0.151 |  | 0.073 | 1.000 | 1.000 | 1.000 | 1.000 | 1.000 |  | 1.000 | 1.000 | 1.000 | 1.000 | 1.000 | 1.000 |
| OTU_457 | 1.000 | 1.000 | **< 0.001** | 1.000 | **0.001** | **< 0.001** |  | 1.000 | 1.000 | **< 0.001** | 1.000 | **< 0.001** | **< 0.001** |  | 1.000 | 1.000 | **0.002** | 1.000 | 0.094 | **< 0.001** |
| OTU_4570 | 1.000 | 1.000 | 0.198 | 1.000 | 1.000 | 0.239 |  | 1.000 | 1.000 | 1.000 | 1.000 | 1.000 | 1.000 |  | 1.000 | 1.000 | 1.000 | 1.000 | 1.000 | 1.000 |
| OTU_458 | 1.000 | 1.000 | 1.000 | 1.000 | 1.000 | 1.000 |  | 1.000 | **< 0.001** | 0.127 | **< 0.001** | 0.458 | 0.380 |  | 1.000 | **0.010** | 0.436 | 1.000 | 1.000 | 1.000 |
| OTU_4581 | 1.000 | 1.000 | 1.000 | 1.000 | 1.000 | 1.000 |  | 1.000 | 1.000 | 1.000 | 1.000 | 1.000 | 1.000 |  | 1.000 | 1.000 | 1.000 | 1.000 | 1.000 | 1.000 |
| OTU_459 | 0.235 | 0.469 | 0.887 | 1.000 | 1.000 | 1.000 |  | 0.235 | 0.872 | 1.000 | 1.000 | 1.000 | 1.000 |  | 1.000 | **0.006** | 1.000 | 1.000 | 1.000 | 0.076 |
| OTU_4597 | 1.000 | 1.000 | 0.186 | 1.000 | 0.404 | 1.000 |  | 1.000 | 1.000 | 1.000 | 1.000 | 0.075 | 1.000 |  | 1.000 | 1.000 | 1.000 | 1.000 | 1.000 | 1.000 |
| OTU_46 | **< 0.001** | **< 0.001** | **< 0.001** | 1.000 | 1.000 | 1.000 |  | **< 0.001** | **< 0.001** | **< 0.001** | 0.310 | 1.000 | 1.000 |  | **< 0.001** | **< 0.001** | **< 0.001** | 0.189 | 1.000 | 1.000 |
| OTU_460 | 1.000 | 1.000 | 0.259 | 1.000 | 0.063 | 1.000 |  | 1.000 | **0.025** | **< 0.001** | 1.000 | 1.000 | 1.000 |  | 1.000 | 1.000 | 0.349 | 1.000 | 0.627 | 1.000 |
| OTU_4600 | 1.000 | 0.743 | 1.000 | 1.000 | 1.000 | 1.000 |  | 1.000 | 0.376 | 0.648 | 1.000 | 1.000 | 1.000 |  | 1.000 | 0.105 | 1.000 | 1.000 | 1.000 | 1.000 |
| OTU_461 | 1.000 | 1.000 | 1.000 | 1.000 | 1.000 | 1.000 |  | 1.000 | **0.007** | 1.000 | 1.000 | 0.991 | 0.677 |  | 1.000 | 1.000 | 0.212 | 1.000 | 0.204 | 0.085 |
| OTU_462 | 1.000 | **0.004** | 1.000 | 1.000 | 0.325 | **0.002** |  | 1.000 | 0.833 | 0.435 | 1.000 | 0.228 | **0.002** |  | 1.000 | 0.618 | 1.000 | 1.000 | 1.000 | 0.375 |
| OTU_463 | **0.036** | **0.020** | **0.005** | 1.000 | 1.000 | 1.000 |  | **0.036** | 1.000 | 1.000 | 1.000 | 1.000 | 1.000 |  | 1.000 | **0.002** | **0.008** | 0.177 | 0.404 | 1.000 |
| OTU_4630 | 1.000 | 1.000 | 1.000 | 1.000 | 0.284 | 0.950 |  | 1.000 | 1.000 | 1.000 | 1.000 | 1.000 | 1.000 |  | 1.000 | 1.000 | 0.654 | 1.000 | 1.000 | 1.000 |
| OTU_4631 | 1.000 | 0.118 | 1.000 | 0.112 | 1.000 | 1.000 |  | 1.000 | **< 0.001** | **< 0.001** | 0.264 | 0.114 | 1.000 |  | 0.181 | **0.002** | **0.032** | 1.000 | 1.000 | 1.000 |
| OTU_464 | 1.000 | 1.000 | 0.740 | 1.000 | 0.088 | 1.000 |  | 1.000 | 0.355 | 0.643 | **0.021** | **0.033** | 1.000 |  | 1.000 | 0.165 | 0.457 | 0.178 | 0.388 | 1.000 |
| OTU_4643 | 1.000 | 1.000 | 1.000 | 0.146 | 1.000 | 1.000 |  | 1.000 | 1.000 | 0.797 | 1.000 | 1.000 | 1.000 |  | 1.000 | 1.000 | 1.000 | 1.000 | 1.000 | 1.000 |
| OTU_465 | 1.000 | **< 0.001** | **< 0.001** | **< 0.001** | **0.002** | 1.000 |  | 1.000 | **< 0.001** | **< 0.001** | **0.009** | **0.005** | 1.000 |  | 0.728 | **< 0.001** | **< 0.001** | **< 0.001** | **0.004** | 1.000 |
| OTU_4657 | 1.000 | 1.000 | 1.000 | 1.000 | 1.000 | 1.000 |  | 1.000 | 1.000 | 1.000 | 1.000 | 1.000 | 1.000 |  | 1.000 | 0.617 | 0.940 | 1.000 | 1.000 | 1.000 |
| OTU_466 | 1.000 | **< 0.001** | 0.509 | **< 0.001** | 1.000 | 0.424 |  | 1.000 | 0.069 | 1.000 | **< 0.001** | **0.003** | 1.000 |  | 1.000 | **0.002** | 0.075 | 0.347 | 1.000 | 1.000 |
| OTU_467 | 1.000 | 1.000 | 0.350 | 1.000 | **< 0.001** | **0.001** |  | 1.000 | 0.090 | 1.000 | 1.000 | 0.055 | 0.275 |  | 0.119 | 0.093 | 1.000 | 1.000 | **0.002** | **0.010** |
| OTU_4670 | 1.000 | 1.000 | 0.051 | 1.000 | 1.000 | 1.000 |  | 1.000 | 0.135 | 0.085 | 1.000 | 1.000 | 1.000 |  | 1.000 | 0.490 | 0.173 | 1.000 | 1.000 | 1.000 |
| OTU_4676 | 1.000 | **0.037** | 1.000 | 1.000 | 1.000 | **0.046** |  | 1.000 | 0.234 | 1.000 | 0.332 | 1.000 | 0.197 |  | 1.000 | **0.003** | 1.000 | 0.883 | 1.000 | 0.517 |
| OTU_468 | 1.000 | **0.003** | **0.004** | 0.175 | 0.154 | 1.000 |  | 1.000 | 0.141 | 0.166 | 0.612 | 0.612 | 1.000 |  | 1.000 | 0.139 | 0.183 | **0.003** | **0.003** | 1.000 |
| OTU_4680 | 0.361 | 1.000 | 1.000 | 0.130 | 0.150 | 1.000 |  | 0.361 | **< 0.001** | **< 0.001** | 1.000 | **0.013** | 1.000 |  | 1.000 | 1.000 | 1.000 | 1.000 | 1.000 | 1.000 |
| OTU_4687 | 1.000 | **< 0.001** | **< 0.001** | **0.007** | **< 0.001** | 0.816 |  | 1.000 | **< 0.001** | **< 0.001** | **< 0.001** | **< 0.001** | 1.000 |  | 1.000 | **0.004** | **< 0.001** | 1.000 | **< 0.001** | **0.014** |
| OTU_469 | 1.000 | **0.004** | 0.307 | 0.101 | 1.000 | 1.000 |  | 1.000 | **< 0.001** | **< 0.001** | 0.292 | 0.238 | 1.000 |  | 1.000 | 0.056 | 0.100 | 1.000 | 1.000 | 1.000 |
| OTU_47 | **< 0.001** | **< 0.001** | **< 0.001** | 0.096 | **< 0.001** | **< 0.001** |  | **< 0.001** | **< 0.001** | **< 0.001** | **< 0.001** | **< 0.001** | 0.584 |  | **< 0.001** | **< 0.001** | **< 0.001** | **< 0.001** | **< 0.001** | 1.000 |
| OTU_470 | 1.000 | 1.000 | 1.000 | 1.000 | 1.000 | 1.000 |  | 1.000 | 1.000 | 1.000 | 1.000 | 1.000 | 1.000 |  | 1.000 | 1.000 | 1.000 | 1.000 | 1.000 | 1.000 |
| OTU_4700 | 1.000 | 0.325 | 0.155 | **0.010** | **0.003** | 1.000 |  | 1.000 | 1.000 | 1.000 | 0.925 | **0.048** | 1.000 |  | 1.000 | 1.000 | 0.370 | 1.000 | **0.001** | 1.000 |
| OTU_4709 | **< 0.001** | **0.009** | 0.502 | 1.000 | 0.152 | 1.000 |  | **< 0.001** | 1.000 | 1.000 | 1.000 | 1.000 | 1.000 |  | 0.195 | **0.010** | 0.178 | 1.000 | 1.000 | 1.000 |
| OTU_471 | 1.000 | 0.389 | 1.000 | 1.000 | 0.978 | 0.241 |  | 1.000 | 1.000 | 1.000 | 1.000 | **0.028** | 1.000 |  | 1.000 | 1.000 | 1.000 | 1.000 | 1.000 | 0.413 |
| OTU_472 | **0.011** | 0.652 | **< 0.001** | 1.000 | **< 0.001** | **< 0.001** |  | **0.011** | 0.673 | **< 0.001** | 1.000 | **< 0.001** | **< 0.001** |  | 1.000 | 1.000 | **0.026** | 1.000 | **< 0.001** | 0.064 |
| OTU_4723 | 1.000 | 1.000 | 0.992 | 1.000 | 1.000 | 1.000 |  | 1.000 | 1.000 | 1.000 | **0.006** | 0.106 | 1.000 |  | 1.000 | 1.000 | 1.000 | 1.000 | 0.510 | 0.191 |
| OTU_4729 | 1.000 | **0.004** | **< 0.001** | 0.247 | **0.006** | 1.000 |  | 1.000 | **0.026** | **0.004** | **< 0.001** | **< 0.001** | 1.000 |  | 1.000 | 1.000 | 1.000 | 1.000 | 1.000 | 1.000 |
| OTU_473 | 0.222 | **0.002** | 1.000 | 1.000 | 1.000 | 0.336 |  | 0.222 | **0.008** | 0.149 | 0.129 | 1.000 | 1.000 |  | 1.000 | **0.003** | 1.000 | 1.000 | 1.000 | 1.000 |
| OTU_474 | 1.000 | 1.000 | 0.088 | 1.000 | **0.023** | 0.334 |  | 1.000 | 0.873 | 1.000 | 1.000 | 1.000 | 1.000 |  | 1.000 | 1.000 | 1.000 | 1.000 | 1.000 | 1.000 |
| OTU_4741 | 1.000 | 1.000 | 1.000 | 1.000 | 1.000 | 1.000 |  | 1.000 | 1.000 | 1.000 | 1.000 | 1.000 | 1.000 |  | 1.000 | 0.498 | 1.000 | 1.000 | 1.000 | 1.000 |
| OTU_4743 | 1.000 | 1.000 | 1.000 | 1.000 | 1.000 | 1.000 |  | 1.000 | 1.000 | 0.194 | 1.000 | 1.000 | 1.000 |  | 1.000 | 1.000 | 1.000 | 1.000 | 0.505 | 1.000 |
| OTU_475 | 1.000 | 1.000 | **< 0.001** | 1.000 | **< 0.001** | **< 0.001** |  | 1.000 | 1.000 | 1.000 | 0.238 | **< 0.001** | 0.051 |  | 1.000 | 1.000 | **< 0.001** | 1.000 | **< 0.001** | **0.012** |
| OTU_4750 | 1.000 | 0.924 | 1.000 | 1.000 | 1.000 | 1.000 |  | 1.000 | **0.047** | 0.506 | 1.000 | 1.000 | 1.000 |  | 1.000 | 0.445 | 1.000 | 1.000 | 1.000 | 1.000 |
| OTU_4751 | 1.000 | 1.000 | 0.809 | 0.330 | 0.064 | 1.000 |  | 1.000 | 1.000 | **< 0.001** | 1.000 | 0.055 | 0.578 |  | 1.000 | 1.000 | 1.000 | 1.000 | 1.000 | 1.000 |
| OTU_4758 | 1.000 | 1.000 | 1.000 | 1.000 | 0.091 | 1.000 |  | 1.000 | 1.000 | **0.016** | 1.000 | **< 0.001** | **0.021** |  | 1.000 | 1.000 | 1.000 | 1.000 | 1.000 | 1.000 |
| OTU_476 | 1.000 | 1.000 | 1.000 | 1.000 | 0.471 | 1.000 |  | 1.000 | 0.616 | 1.000 | 1.000 | 1.000 | 0.827 |  | 1.000 | 1.000 | 1.000 | 1.000 | 0.391 | 1.000 |
| OTU_477 | 0.542 | **0.038** | 0.094 | 1.000 | 1.000 | 1.000 |  | 0.542 | **0.032** | **0.004** | 1.000 | 1.000 | 1.000 |  | 1.000 | 1.000 | 1.000 | 1.000 | 1.000 | 1.000 |
| OTU_478 | 0.445 | 1.000 | **0.003** | 0.353 | **< 0.001** | **0.007** |  | 0.445 | 1.000 | **< 0.001** | 1.000 | **< 0.001** | **0.008** |  | 0.805 | 1.000 | 1.000 | 1.000 | **0.032** | 0.457 |
| OTU_4784 | 1.000 | 1.000 | 0.215 | 1.000 | 0.441 | 0.985 |  | 1.000 | 0.224 | **0.005** | 1.000 | 1.000 | 1.000 |  | 1.000 | 1.000 | 0.991 | 1.000 | 1.000 | 1.000 |
| OTU_479 | 0.577 | 0.952 | 1.000 | 1.000 | 1.000 | 1.000 |  | 0.577 | **0.030** | 0.085 | 1.000 | 1.000 | 1.000 |  | 1.000 | 0.868 | 1.000 | 1.000 | 1.000 | 1.000 |
| OTU_4797 | 1.000 | 1.000 | 1.000 | 1.000 | 1.000 | 1.000 |  | 1.000 | 1.000 | 0.603 | 1.000 | 1.000 | 1.000 |  | 1.000 | 1.000 | 0.213 | 1.000 | 1.000 | 1.000 |
| OTU_48 | 1.000 | **0.001** | **0.008** | 1.000 | 1.000 | 1.000 |  | 1.000 | **< 0.001** | **< 0.001** | 1.000 | 0.690 | 1.000 |  | 0.608 | **< 0.001** | **0.011** | 1.000 | 1.000 | 1.000 |
| OTU_480 | 0.513 | 0.142 | **0.023** | **< 0.001** | **< 0.001** | 1.000 |  | 0.513 | **< 0.001** | **< 0.001** | **< 0.001** | **< 0.001** | 1.000 |  | 1.000 | **< 0.001** | 0.230 | **< 0.001** | 0.204 | 1.000 |
| OTU_4806 | 0.054 | 0.211 | 1.000 | 1.000 | 1.000 | 1.000 |  | 0.054 | 1.000 | 1.000 | 1.000 | 1.000 | 1.000 |  | 0.259 | 1.000 | 1.000 | 1.000 | 0.300 | 1.000 |
| OTU_4808 | 1.000 | 1.000 | 1.000 | 1.000 | 1.000 | 1.000 |  | 1.000 | 0.075 | 1.000 | **< 0.001** | **0.007** | 1.000 |  | 1.000 | 1.000 | 1.000 | 0.946 | 1.000 | 1.000 |
| OTU_481 | 1.000 | 1.000 | 1.000 | 1.000 | 1.000 | 1.000 |  | 1.000 | 1.000 | 1.000 | 1.000 | 1.000 | 1.000 |  | 1.000 | 0.774 | 1.000 | 1.000 | 1.000 | 1.000 |
| OTU_482 | 1.000 | 1.000 | 1.000 | 1.000 | 1.000 | 0.954 |  | 1.000 | 1.000 | **0.032** | **0.039** | 1.000 | 0.879 |  | 1.000 | 1.000 | 1.000 | **0.032** | 1.000 | 1.000 |
| OTU_483 | 1.000 | 1.000 | 1.000 | 1.000 | 1.000 | 0.539 |  | 1.000 | 1.000 | 0.642 | 1.000 | 1.000 | 1.000 |  | 1.000 | 1.000 | 1.000 | 1.000 | 1.000 | 1.000 |
| OTU_4832 | 1.000 | 1.000 | 1.000 | 1.000 | 1.000 | 1.000 |  | 1.000 | 1.000 | 0.180 | 1.000 | 0.137 | 0.564 |  | 1.000 | 1.000 | 0.173 | 1.000 | 1.000 | 1.000 |
| OTU_4833 | 1.000 | 0.174 | 0.316 | 1.000 | 1.000 | 1.000 |  | 1.000 | **0.028** | **< 0.001** | 0.739 | **0.047** | 1.000 |  | 1.000 | 1.000 | 1.000 | 1.000 | 1.000 | 1.000 |
| OTU_484 | 1.000 | 0.205 | **0.016** | 0.074 | **0.003** | 1.000 |  | 1.000 | **< 0.001** | **< 0.001** | 0.232 | **0.031** | 1.000 |  | 1.000 | **< 0.001** | **0.010** | **0.020** | 0.105 | 1.000 |
| OTU_4847 | 1.000 | 0.445 | 1.000 | 1.000 | 1.000 | 1.000 |  | 1.000 | 0.728 | 1.000 | 0.616 | 1.000 | 1.000 |  | 1.000 | 1.000 | 1.000 | 1.000 | 1.000 | 0.884 |
| OTU_485 | 1.000 | 1.000 | 1.000 | 1.000 | 1.000 | 1.000 |  | 1.000 | 0.132 | **0.020** | 1.000 | 1.000 | 1.000 |  | 1.000 | 1.000 | 1.000 | 1.000 | 1.000 | 1.000 |
| OTU_486 | 1.000 | 1.000 | 0.740 | 1.000 | 1.000 | 1.000 |  | 1.000 | 0.058 | 1.000 | 1.000 | 1.000 | 1.000 |  | 1.000 | 0.741 | 1.000 | 1.000 | 1.000 | 1.000 |
| OTU_4862 | 0.327 | 1.000 | 1.000 | 1.000 | 0.132 | 1.000 |  | 0.327 | 0.441 | 1.000 | 1.000 | 0.493 | 0.373 |  | 0.946 | 1.000 | 1.000 | 1.000 | 1.000 | 1.000 |
| OTU_488 | 1.000 | 0.273 | 0.952 | 1.000 | 1.000 | 1.000 |  | 1.000 | **< 0.001** | **0.010** | 1.000 | 1.000 | 1.000 |  | 1.000 | **0.014** | **0.022** | 1.000 | 1.000 | 1.000 |
| OTU_4880 | 1.000 | 1.000 | 1.000 | 1.000 | 1.000 | 1.000 |  | 1.000 | **0.004** | 0.094 | **< 0.001** | **0.004** | 1.000 |  | 1.000 | 0.389 | 1.000 | 0.410 | 1.000 | 1.000 |
| OTU_489 | 1.000 | **0.001** | 0.792 | **0.033** | 1.000 | 0.753 |  | 1.000 | **< 0.001** | **0.006** | **< 0.001** | **< 0.001** | 1.000 |  | 1.000 | **< 0.001** | **< 0.001** | **0.028** | **0.032** | 1.000 |
| OTU_4894 | 1.000 | 0.212 | 1.000 | 0.142 | 1.000 | 1.000 |  | 1.000 | **< 0.001** | 1.000 | **< 0.001** | 1.000 | **< 0.001** |  | 1.000 | 1.000 | 1.000 | 1.000 | 0.925 | **0.033** |
| OTU_49 | 1.000 | **< 0.001** | 1.000 | 0.078 | 1.000 | **0.023** |  | 1.000 | 1.000 | 1.000 | 0.283 | 1.000 | 0.094 |  | 1.000 | **0.013** | 1.000 | 1.000 | 1.000 | 0.941 |
| OTU_490 | 1.000 | **0.050** | 0.527 | 1.000 | 1.000 | 1.000 |  | 1.000 | 1.000 | 1.000 | 0.769 | 1.000 | 1.000 |  | 1.000 | 0.152 | 0.241 | 1.000 | 1.000 | 1.000 |
| OTU_4908 | **0.006** | **< 0.001** | **< 0.001** | 1.000 | 0.169 | 1.000 |  | **0.006** | **< 0.001** | **< 0.001** | 1.000 | **0.027** | 1.000 |  | 1.000 | **0.027** | **0.026** | 1.000 | 1.000 | 1.000 |
| OTU_491 | 1.000 | 1.000 | 1.000 | 1.000 | 1.000 | 1.000 |  | 1.000 | 1.000 | 0.673 | 1.000 | 1.000 | 0.620 |  | 1.000 | 1.000 | 1.000 | 1.000 | 1.000 | 1.000 |
| OTU_492 | 1.000 | **0.003** | **0.011** | **< 0.001** | **< 0.001** | 1.000 |  | 1.000 | 0.154 | 1.000 | 0.080 | 0.567 | 1.000 |  | 1.000 | 0.056 | **0.002** | 0.111 | **0.003** | 1.000 |
| OTU_493 | 1.000 | 1.000 | 1.000 | 1.000 | 1.000 | 1.000 |  | 1.000 | **0.032** | 0.071 | 0.194 | 0.311 | 1.000 |  | 1.000 | 0.460 | 1.000 | 1.000 | 1.000 | 1.000 |
| OTU_494 | 1.000 | 1.000 | 1.000 | 1.000 | 1.000 | 1.000 |  | 1.000 | 0.060 | 0.392 | **0.005** | **0.030** | 1.000 |  | 1.000 | 1.000 | 1.000 | 1.000 | 1.000 | 1.000 |
| OTU_4940 | 0.666 | 0.158 | 1.000 | 1.000 | 1.000 | 1.000 |  | 0.666 | 1.000 | 1.000 | 1.000 | 1.000 | 1.000 |  | 1.000 | 1.000 | 1.000 | 1.000 | 1.000 | 1.000 |
| OTU_495 | 1.000 | **< 0.001** | **0.043** | **< 0.001** | 1.000 | **0.004** |  | 1.000 | **< 0.001** | 0.154 | **< 0.001** | 0.227 | **0.009** |  | 0.943 | **< 0.001** | 1.000 | **< 0.001** | **0.044** | **0.017** |
| OTU_496 | **0.007** | **0.002** | **< 0.001** | 1.000 | 0.739 | 1.000 |  | **0.007** | 1.000 | **0.002** | 1.000 | **0.017** | 0.879 |  | 1.000 | 1.000 | 0.143 | 1.000 | 0.620 | 1.000 |
| OTU_497 | 1.000 | 1.000 | **0.025** | 1.000 | 0.350 | **0.013** |  | 1.000 | 1.000 | **0.004** | 1.000 | **0.006** | **< 0.001** |  | 1.000 | 1.000 | 0.191 | 1.000 | **0.027** | **0.043** |
| OTU_4972 | 1.000 | 0.216 | 1.000 | 1.000 | 1.000 | 1.000 |  | 1.000 | **0.007** | 1.000 | 0.555 | 1.000 | 1.000 |  | 1.000 | 1.000 | 0.845 | 1.000 | 1.000 | 1.000 |
| OTU_498 | 1.000 | 0.929 | 0.346 | 1.000 | 0.192 | **< 0.001** |  | 1.000 | 0.507 | **< 0.001** | 1.000 | 0.106 | 1.000 |  | 1.000 | 1.000 | 1.000 | 1.000 | 1.000 | 1.000 |
| OTU_4986 | 0.568 | 0.503 | **0.047** | 1.000 | 1.000 | 1.000 |  | 0.568 | **0.005** | 0.150 | 1.000 | 1.000 | 1.000 |  | **< 0.001** | **< 0.001** | **< 0.001** | 1.000 | 1.000 | 1.000 |
| OTU_4988 | 1.000 | 0.679 | 1.000 | 0.062 | 0.747 | 1.000 |  | 1.000 | 0.799 | 0.366 | 1.000 | 1.000 | 1.000 |  | 1.000 | 1.000 | 0.537 | 1.000 | 0.593 | 1.000 |
| OTU_499 | 0.416 | 1.000 | 0.117 | 1.000 | **< 0.001** | **0.002** |  | 0.416 | 0.670 | **< 0.001** | 1.000 | **< 0.001** | **< 0.001** |  | 1.000 | 1.000 | **0.003** | 1.000 | **< 0.001** | **< 0.001** |
| OTU_4990 | 1.000 | 1.000 | 1.000 | 1.000 | 0.836 | 1.000 |  | 1.000 | 1.000 | 1.000 | 1.000 | 1.000 | 1.000 |  | 1.000 | 1.000 | 1.000 | 1.000 | 1.000 | 1.000 |
| OTU_5 | 0.070 | **< 0.001** | **< 0.001** | 0.610 | 0.751 | 1.000 |  | 0.070 | **< 0.001** | **< 0.001** | 0.270 | 0.072 | 1.000 |  | 1.000 | **< 0.001** | **< 0.001** | **0.007** | 0.062 | 1.000 |
| OTU_500 | 1.000 | 1.000 | **0.019** | 1.000 | 1.000 | **0.033** |  | 1.000 | 1.000 | **0.033** | 0.686 | 1.000 | 0.110 |  | 1.000 | 1.000 | 0.653 | 1.000 | 1.000 | 1.000 |
| OTU_501 | 1.000 | 1.000 | **< 0.001** | 0.376 | **< 0.001** | **0.017** |  | 1.000 | 0.277 | **< 0.001** | 1.000 | **< 0.001** | **0.004** |  | 1.000 | **< 0.001** | **< 0.001** | **0.021** | **< 0.001** | **0.007** |
| OTU_502 | 1.000 | 1.000 | 1.000 | 0.106 | 0.252 | 1.000 |  | 1.000 | 0.168 | 1.000 | 1.000 | 0.515 | 1.000 |  | **0.025** | 1.000 | 1.000 | 1.000 | 0.080 | 1.000 |
| OTU_5023 | 0.846 | 0.078 | **< 0.001** | 1.000 | **0.031** | 0.402 |  | 0.846 | **0.016** | **0.004** | 0.373 | 0.109 | 1.000 |  | 1.000 | 1.000 | 0.506 | 1.000 | 1.000 | 1.000 |
| OTU_503 | 1.000 | 1.000 | 0.122 | 1.000 | 0.195 | 1.000 |  | 1.000 | **0.001** | **< 0.001** | 1.000 | 1.000 | 1.000 |  | 1.000 | 1.000 | 1.000 | 1.000 | 1.000 | 1.000 |
| OTU_504 | 0.208 | **< 0.001** | **< 0.001** | 0.062 | 0.203 | 1.000 |  | 0.208 | 0.398 | 0.169 | 0.302 | 0.099 | 1.000 |  | 1.000 | 1.000 | **0.002** | 1.000 | 0.059 | 0.281 |
| OTU_505 | 1.000 | 1.000 | 1.000 | 1.000 | 1.000 | 1.000 |  | 1.000 | 1.000 | 1.000 | 1.000 | 1.000 | 1.000 |  | 1.000 | 1.000 | 0.540 | 1.000 | 1.000 | 1.000 |
| OTU_5052 | 1.000 | 1.000 | 1.000 | 1.000 | 1.000 | 1.000 |  | 1.000 | 0.863 | 1.000 | 0.449 | 1.000 | 1.000 |  | 1.000 | 1.000 | 0.992 | 1.000 | 0.144 | 1.000 |
| OTU_5053 | 1.000 | 1.000 | 1.000 | 1.000 | 1.000 | 1.000 |  | 1.000 | **0.002** | **0.030** | 1.000 | 1.000 | 1.000 |  | 1.000 | 0.278 | 0.201 | 1.000 | 1.000 | 1.000 |
| OTU_5056 | 1.000 | 1.000 | 1.000 | 1.000 | 1.000 | 1.000 |  | 1.000 | 1.000 | 1.000 | 1.000 | 1.000 | 1.000 |  | 1.000 | 1.000 | 0.180 | 1.000 | 1.000 | 1.000 |
| OTU_506 | 1.000 | 1.000 | 1.000 | 1.000 | 1.000 | 1.000 |  | 1.000 | 0.110 | **0.008** | 0.348 | **0.028** | 1.000 |  | 1.000 | 1.000 | 1.000 | 1.000 | 1.000 | 1.000 |
| OTU_5066 | 1.000 | **< 0.001** | **< 0.001** | **0.037** | **0.005** | 1.000 |  | 1.000 | **< 0.001** | **< 0.001** | 0.093 | **0.009** | 1.000 |  | **0.004** | **< 0.001** | **< 0.001** | 1.000 | 1.000 | 1.000 |
| OTU_507 | **< 0.001** | **< 0.001** | **< 0.001** | **< 0.001** | **< 0.001** | 1.000 |  | **< 0.001** | **< 0.001** | **< 0.001** | **< 0.001** | **< 0.001** | 1.000 |  | 0.163 | **< 0.001** | **< 0.001** | **< 0.001** | **< 0.001** | 1.000 |
| OTU_5075 | 0.445 | 0.432 | 0.390 | 1.000 | 1.000 | 1.000 |  | 0.445 | 0.218 | 1.000 | 1.000 | 1.000 | 1.000 |  | 1.000 | 1.000 | 1.000 | 1.000 | 1.000 | 1.000 |
| OTU_508 | 0.292 | **0.039** | **0.001** | **< 0.001** | **< 0.001** | 1.000 |  | 0.292 | **0.012** | **< 0.001** | **0.006** | **< 0.001** | 1.000 |  | 1.000 | **0.010** | **< 0.001** | 0.108 | **< 0.001** | 1.000 |
| OTU_509 | 1.000 | 0.480 | 0.050 | 0.901 | 0.083 | 1.000 |  | 1.000 | 1.000 | 0.097 | 1.000 | 0.081 | 1.000 |  | 1.000 | 1.000 | 0.193 | 1.000 | 0.113 | 1.000 |
| OTU_5091 | 1.000 | 0.704 | 0.091 | 1.000 | 0.114 | 1.000 |  | 1.000 | 1.000 | 1.000 | 0.256 | 0.170 | 1.000 |  | 1.000 | **0.032** | 0.431 | 0.896 | 1.000 | 1.000 |
| OTU_51 | 0.546 | **< 0.001** | **< 0.001** | **< 0.001** | 0.188 | **0.009** |  | 0.546 | **< 0.001** | **< 0.001** | **< 0.001** | **< 0.001** | **0.021** |  | 0.608 | **< 0.001** | **< 0.001** | **< 0.001** | **0.002** | 1.000 |
| OTU_510 | 1.000 | 0.258 | 1.000 | 1.000 | 1.000 | 1.000 |  | 1.000 | 1.000 | 1.000 | 1.000 | 0.454 | 1.000 |  | 1.000 | 1.000 | 1.000 | 1.000 | 1.000 | 1.000 |
| OTU_5102 | 1.000 | 1.000 | 1.000 | 1.000 | 1.000 | 1.000 |  | 1.000 | 1.000 | 1.000 | 1.000 | 1.000 | 1.000 |  | 1.000 | 1.000 | 1.000 | 1.000 | 1.000 | 1.000 |
| OTU_5103 | 1.000 | 1.000 | **0.004** | 0.770 | **< 0.001** | 0.537 |  | 1.000 | 0.226 | **< 0.001** | 0.318 | **< 0.001** | 1.000 |  | 1.000 | 1.000 | 0.479 | 1.000 | 1.000 | 1.000 |
| OTU_5109 | 1.000 | 0.333 | 1.000 | 0.976 | 1.000 | 1.000 |  | 1.000 | 1.000 | 1.000 | 1.000 | 1.000 | 1.000 |  | 1.000 | 1.000 | 1.000 | 1.000 | 1.000 | 1.000 |
| OTU_511 | 1.000 | 1.000 | 1.000 | 1.000 | 1.000 | 1.000 |  | 1.000 | 1.000 | 0.120 | 1.000 | **0.016** | 1.000 |  | 1.000 | 0.289 | 0.106 | 1.000 | 1.000 | 1.000 |
| OTU_5117 | 1.000 | 0.569 | 1.000 | 1.000 | 1.000 | 1.000 |  | 1.000 | **0.033** | 0.089 | 1.000 | 1.000 | 1.000 |  | 1.000 | 1.000 | 1.000 | 1.000 | 1.000 | 1.000 |
| OTU_512 | 1.000 | 1.000 | **0.011** | 1.000 | **0.002** | 0.166 |  | 1.000 | 1.000 | **< 0.001** | 1.000 | **0.005** | **0.027** |  | 1.000 | 1.000 | **< 0.001** | 1.000 | 0.060 | **0.014** |
| OTU_5127 | 1.000 | 0.116 | 0.103 | 0.623 | 0.438 | 1.000 |  | 1.000 | 1.000 | 0.448 | 1.000 | 0.766 | 1.000 |  | 1.000 | 1.000 | 1.000 | 1.000 | 1.000 | 1.000 |
| OTU_513 | 1.000 | 1.000 | 1.000 | 1.000 | 1.000 | 1.000 |  | 1.000 | 1.000 | 0.105 | 1.000 | 1.000 | 1.000 |  | 0.855 | 0.482 | 1.000 | 1.000 | 1.000 | 1.000 |
| OTU_5145 | 1.000 | 1.000 | 0.454 | 0.749 | 0.069 | 1.000 |  | 1.000 | 1.000 | 0.980 | 1.000 | 1.000 | 1.000 |  | 1.000 | 1.000 | 1.000 | 1.000 | 1.000 | 1.000 |
| OTU_515 | 1.000 | 1.000 | **0.002** | 1.000 | **0.016** | **0.032** |  | 1.000 | 1.000 | **< 0.001** | 1.000 | **< 0.001** | **< 0.001** |  | 1.000 | 1.000 | **< 0.001** | 1.000 | **< 0.001** | **0.024** |
| OTU_5156 | 1.000 | 1.000 | 1.000 | 1.000 | 1.000 | 1.000 |  | 1.000 | **0.046** | 1.000 | **0.005** | 0.212 | 1.000 |  | 1.000 | 1.000 | 1.000 | 1.000 | 1.000 | 1.000 |
| OTU_516 | 1.000 | 1.000 | 1.000 | 1.000 | 1.000 | 1.000 |  | 1.000 | 1.000 | 1.000 | 0.097 | **< 0.001** | 1.000 |  | 1.000 | 1.000 | 1.000 | 1.000 | 1.000 | 1.000 |
| OTU_517 | 1.000 | 0.098 | 1.000 | 1.000 | 1.000 | 1.000 |  | 1.000 | **< 0.001** | 0.262 | 1.000 | 1.000 | 1.000 |  | 1.000 | 1.000 | 1.000 | 1.000 | 1.000 | 1.000 |
| OTU_5175 | 1.000 | **< 0.001** | 0.064 | **< 0.001** | 0.447 | **< 0.001** |  | 1.000 | **0.004** | 1.000 | **< 0.001** | 1.000 | **0.027** |  | 1.000 | **< 0.001** | **0.002** | **< 0.001** | 0.092 | 1.000 |
| OTU_518 | 1.000 | 1.000 | 1.000 | 1.000 | 0.105 | 1.000 |  | 1.000 | 1.000 | **0.009** | 0.093 | **< 0.001** | 1.000 |  | 1.000 | **0.011** | 0.147 | **0.021** | 0.186 | 1.000 |
| OTU_5181 | 0.566 | **< 0.001** | **< 0.001** | 1.000 | 0.054 | 1.000 |  | 0.566 | 1.000 | **0.009** | 1.000 | 0.121 | 1.000 |  | 0.170 | **< 0.001** | **< 0.001** | 0.836 | 0.318 | 1.000 |
| OTU_519 | **< 0.001** | 0.097 | 1.000 | 1.000 | **< 0.001** | 0.087 |  | **< 0.001** | **< 0.001** | 1.000 | 1.000 | **< 0.001** | **< 0.001** |  | **0.024** | **0.007** | 1.000 | 1.000 | **0.007** | **0.012** |
| OTU_5196 | 1.000 | 1.000 | 0.058 | 1.000 | **0.047** | 0.866 |  | 1.000 | 1.000 | 0.369 | 1.000 | 0.456 | 0.869 |  | 1.000 | 1.000 | 0.500 | 1.000 | 0.722 | 1.000 |
| OTU_5199 | 0.416 | 0.270 | 0.235 | 1.000 | 1.000 | 1.000 |  | 0.416 | 1.000 | 1.000 | 1.000 | 1.000 | 1.000 |  | 1.000 | 1.000 | 1.000 | 1.000 | 1.000 | 1.000 |
| OTU_52 | **< 0.001** | 0.272 | 0.116 | 1.000 | **< 0.001** | **< 0.001** |  | **< 0.001** | 1.000 | **< 0.001** | **0.014** | **< 0.001** | **< 0.001** |  | 1.000 | 1.000 | 0.339 | 0.764 | 0.202 | 1.000 |
| OTU_520 | 1.000 | 1.000 | 0.155 | 1.000 | **0.025** | 1.000 |  | 1.000 | 1.000 | 1.000 | 1.000 | **0.006** | **0.046** |  | 1.000 | 1.000 | 0.535 | 0.628 | **0.004** | 1.000 |
| OTU_521 | 1.000 | 1.000 | **0.006** | 1.000 | 1.000 | **0.032** |  | 1.000 | 1.000 | 1.000 | 0.495 | 1.000 | 1.000 |  | 1.000 | 1.000 | 0.339 | 1.000 | 0.403 | 1.000 |
| OTU_5219 | 0.815 | **0.015** | **0.007** | 1.000 | 1.000 | 1.000 |  | 0.815 | **< 0.001** | **< 0.001** | 1.000 | 0.834 | 1.000 |  | 0.362 | **0.005** | 0.109 | 1.000 | 1.000 | 1.000 |
| OTU_522 | 1.000 | **< 0.001** | **< 0.001** | **0.005** | **< 0.001** | 1.000 |  | 1.000 | **0.014** | **0.008** | 0.057 | **0.027** | 1.000 |  | 0.383 | **< 0.001** | **< 0.001** | 1.000 | 0.336 | 1.000 |
| OTU_5220 | 1.000 | **0.006** | **0.016** | **0.038** | 0.070 | 1.000 |  | 1.000 | 1.000 | 1.000 | 1.000 | 1.000 | 1.000 |  | **0.004** | **< 0.001** | **< 0.001** | 1.000 | 1.000 | 1.000 |
| OTU_5224 | 1.000 | 1.000 | 0.461 | 1.000 | 0.080 | 1.000 |  | 1.000 | **0.025** | **0.002** | 0.299 | **0.027** | 1.000 |  | 1.000 | **0.009** | **< 0.001** | 1.000 | 0.090 | 1.000 |
| OTU_5228 | 1.000 | 0.336 | **< 0.001** | **0.026** | **< 0.001** | **< 0.001** |  | 1.000 | 0.218 | **< 0.001** | **0.005** | **< 0.001** | **< 0.001** |  | **0.025** | **< 0.001** | **< 0.001** | 0.189 | **0.008** | 1.000 |
| OTU_523 | 0.385 | 1.000 | 1.000 | 1.000 | 1.000 | 1.000 |  | 0.385 | 1.000 | 1.000 | 1.000 | 1.000 | 1.000 |  | 1.000 | 1.000 | 1.000 | 1.000 | 1.000 | 1.000 |
| OTU_5231 | 1.000 | 1.000 | 1.000 | 1.000 | 1.000 | 1.000 |  | 1.000 | 1.000 | 1.000 | 1.000 | 1.000 | 1.000 |  | 0.152 | 1.000 | 0.753 | 1.000 | 1.000 | 1.000 |
| OTU_524 | 1.000 | **0.006** | 0.265 | 1.000 | 1.000 | 1.000 |  | 1.000 | **< 0.001** | **0.032** | 1.000 | 1.000 | 1.000 |  | 1.000 | **0.003** | **0.005** | 0.380 | 0.435 | 1.000 |
| OTU_5244 | 1.000 | 0.126 | 1.000 | 0.054 | 1.000 | 1.000 |  | 1.000 | **0.002** | **0.007** | **0.044** | 0.090 | 1.000 |  | 1.000 | 1.000 | 0.822 | 1.000 | 0.594 | 1.000 |
| OTU_525 | 1.000 | 1.000 | **0.013** | 1.000 | 0.062 | 1.000 |  | 1.000 | **0.008** | **< 0.001** | 1.000 | 0.194 | 1.000 |  | 1.000 | 0.174 | **0.014** | 1.000 | 1.000 | 1.000 |
| OTU_526 | 0.755 | 0.067 | 0.253 | 1.000 | 1.000 | 1.000 |  | 0.755 | 1.000 | 0.839 | **< 0.001** | **< 0.001** | 1.000 |  | 1.000 | 1.000 | 1.000 | 0.429 | 1.000 | 1.000 |
| OTU_5263 | 1.000 | 0.508 | 0.050 | 1.000 | 1.000 | 1.000 |  | 1.000 | 1.000 | 1.000 | 0.124 | 0.053 | 1.000 |  | 1.000 | 1.000 | **0.002** | 1.000 | **0.034** | 0.337 |
| OTU_527 | 1.000 | 1.000 | 1.000 | 1.000 | 1.000 | 1.000 |  | 1.000 | 1.000 | 1.000 | 1.000 | 1.000 | 1.000 |  | 1.000 | 0.127 | 0.991 | 1.000 | 1.000 | 1.000 |
| OTU_528 | 1.000 | 1.000 | **0.049** | 0.059 | **< 0.001** | 1.000 |  | 1.000 | **< 0.001** | **0.017** | **0.021** | 0.728 | 1.000 |  | 1.000 | 0.256 | 1.000 | 1.000 | 1.000 | 1.000 |
| OTU_5282 | 1.000 | 0.183 | **0.026** | 1.000 | 0.294 | 1.000 |  | 1.000 | **0.003** | **0.002** | 0.631 | 0.347 | 1.000 |  | 1.000 | 1.000 | 1.000 | 1.000 | 0.950 | 1.000 |
| OTU_529 | 1.000 | 1.000 | 1.000 | 1.000 | 1.000 | 1.000 |  | 1.000 | 0.769 | **0.026** | 1.000 | 1.000 | 1.000 |  | 1.000 | 1.000 | 1.000 | 1.000 | 1.000 | 1.000 |
| OTU_53 | **0.001** | 0.568 | **< 0.001** | 1.000 | **< 0.001** | **< 0.001** |  | **0.001** | 0.827 | **< 0.001** | 1.000 | **< 0.001** | **< 0.001** |  | 1.000 | 1.000 | **< 0.001** | 1.000 | **< 0.001** | **< 0.001** |
| OTU_530 | 1.000 | 1.000 | **0.040** | 0.801 | **0.001** | 1.000 |  | 1.000 | **< 0.001** | **< 0.001** | 0.925 | **0.038** | 1.000 |  | 1.000 | 0.997 | **0.002** | 1.000 | 1.000 | 1.000 |
| OTU_531 | 1.000 | 0.054 | 0.073 | **0.041** | **0.040** | 1.000 |  | 1.000 | 1.000 | 1.000 | 0.580 | 1.000 | 1.000 |  | 1.000 | 0.107 | **0.010** | 1.000 | 0.950 | 1.000 |
| OTU_5312 | 1.000 | 0.827 | **0.044** | 0.457 | **0.014** | 1.000 |  | 1.000 | 1.000 | 1.000 | 1.000 | 0.182 | 1.000 |  | 0.756 | 0.086 | **0.001** | 1.000 | 1.000 | 1.000 |
| OTU_532 | 0.105 | **< 0.001** | 1.000 | 1.000 | 1.000 | 0.201 |  | 0.105 | **< 0.001** | **< 0.001** | 0.192 | 1.000 | 1.000 |  | **0.005** | **< 0.001** | **< 0.001** | 0.135 | 1.000 | 1.000 |
| OTU_533 | **< 0.001** | **< 0.001** | **< 0.001** | 0.211 | 1.000 | 1.000 |  | **< 0.001** | **< 0.001** | **< 0.001** | 1.000 | 1.000 | 1.000 |  | **0.002** | **< 0.001** | **< 0.001** | 0.643 | **0.009** | 1.000 |
| OTU_5330 | 1.000 | 1.000 | 0.145 | 1.000 | 1.000 | 1.000 |  | 1.000 | 0.420 | **0.010** | 0.473 | **0.011** | 1.000 |  | 1.000 | 1.000 | 0.207 | 1.000 | 1.000 | 1.000 |
| OTU_5337 | 0.704 | 1.000 | 0.641 | 0.259 | **< 0.001** | 1.000 |  | 0.704 | 1.000 | 1.000 | 1.000 | 0.593 | 0.175 |  | 1.000 | 1.000 | 0.902 | 1.000 | 1.000 | 1.000 |
| OTU_534 | 1.000 | **0.015** | **0.031** | **0.007** | **0.010** | 1.000 |  | 1.000 | **0.004** | **< 0.001** | **0.007** | **< 0.001** | 1.000 |  | 0.341 | 1.000 | 1.000 | 1.000 | 0.189 | 1.000 |
| OTU_535 | 1.000 | 1.000 | 0.568 | 1.000 | 1.000 | 1.000 |  | 1.000 | 0.341 | **0.032** | 1.000 | 1.000 | 1.000 |  | 1.000 | **0.011** | **0.008** | 1.000 | 1.000 | 1.000 |
| OTU_5352 | 1.000 | 1.000 | **< 0.001** | 1.000 | **< 0.001** | **0.003** |  | 1.000 | 1.000 | **< 0.001** | 1.000 | **< 0.001** | 0.066 |  | 1.000 | **0.002** | **< 0.001** | 0.252 | **< 0.001** | 1.000 |
| OTU_536 | 1.000 | 0.368 | 1.000 | 0.125 | 1.000 | 0.271 |  | 1.000 | 0.381 | 1.000 | 1.000 | 1.000 | 1.000 |  | 1.000 | 0.653 | 1.000 | 0.359 | 1.000 | 0.299 |
| OTU_537 | **0.015** | 1.000 | 0.141 | **0.002** | **< 0.001** | 0.806 |  | **0.015** | 0.183 | **0.009** | **0.012** | **< 0.001** | 1.000 |  | 1.000 | 0.372 | **0.005** | 1.000 | 1.000 | 1.000 |
| OTU_5370 | 1.000 | 1.000 | 1.000 | 1.000 | 0.244 | 1.000 |  | 1.000 | 0.172 | 1.000 | 0.283 | 1.000 | 1.000 |  | 1.000 | 0.140 | 1.000 | 0.208 | 1.000 | 1.000 |
| OTU_538 | 1.000 | 1.000 | **0.017** | 0.864 | **< 0.001** | 1.000 |  | 1.000 | 1.000 | 1.000 | 1.000 | 0.099 | 1.000 |  | 1.000 | 1.000 | **< 0.001** | 1.000 | **0.008** | 0.079 |
| OTU_539 | 1.000 | **< 0.001** | 1.000 | **< 0.001** | 0.662 | **< 0.001** |  | 1.000 | **< 0.001** | 1.000 | **< 0.001** | 1.000 | **< 0.001** |  | 1.000 | **< 0.001** | 1.000 | **< 0.001** | 1.000 | **< 0.001** |
| OTU_5391 | 1.000 | 1.000 | 1.000 | 1.000 | **0.039** | 1.000 |  | 1.000 | 1.000 | 0.160 | 0.425 | **0.002** | 1.000 |  | 1.000 | 1.000 | 1.000 | 1.000 | 1.000 | 1.000 |
| OTU_5398 | 1.000 | 1.000 | 1.000 | 1.000 | 1.000 | 1.000 |  | 1.000 | **0.003** | 0.830 | 0.093 | 1.000 | 1.000 |  | 1.000 | 0.255 | 1.000 | 1.000 | 1.000 | 1.000 |
| OTU_54 | **0.035** | **< 0.001** | **< 0.001** | **0.021** | 0.540 | 1.000 |  | **0.035** | **< 0.001** | **< 0.001** | 0.073 | **0.027** | 1.000 |  | 0.641 | **0.003** | **< 0.001** | 1.000 | 1.000 | 1.000 |
| OTU_541 | 1.000 | 1.000 | 1.000 | 1.000 | 1.000 | 1.000 |  | 1.000 | 0.068 | 0.061 | 0.103 | 0.073 | 1.000 |  | 1.000 | 1.000 | 1.000 | 1.000 | 1.000 | 1.000 |
| OTU_5417 | 1.000 | 1.000 | 1.000 | 1.000 | 1.000 | 1.000 |  | 1.000 | 1.000 | 1.000 | 1.000 | 1.000 | 1.000 |  | 1.000 | 1.000 | 1.000 | 1.000 | 0.955 | 1.000 |
| OTU_542 | 1.000 | 0.259 | 0.572 | 1.000 | 1.000 | 1.000 |  | 1.000 | 1.000 | 1.000 | 1.000 | 1.000 | 1.000 |  | 1.000 | **0.005** | **0.014** | 1.000 | 1.000 | 1.000 |
| OTU_543 | 1.000 | 0.059 | **0.006** | **0.006** | **< 0.001** | 1.000 |  | 1.000 | 0.337 | 0.085 | **0.001** | **< 0.001** | 1.000 |  | 1.000 | 1.000 | **0.035** | 1.000 | **0.003** | 0.708 |
| OTU_5431 | 1.000 | 1.000 | 1.000 | 1.000 | 1.000 | 1.000 |  | 1.000 | **< 0.001** | **< 0.001** | 0.477 | 0.765 | 1.000 |  | 1.000 | 1.000 | 1.000 | 1.000 | 1.000 | 1.000 |
| OTU_5439 | 1.000 | **< 0.001** | 0.151 | **0.002** | 0.970 | 1.000 |  | 1.000 | **< 0.001** | **0.022** | 0.155 | 1.000 | 1.000 |  | 0.821 | **0.012** | 0.253 | 1.000 | 1.000 | 1.000 |
| OTU_544 | 1.000 | 1.000 | 0.759 | 1.000 | 1.000 | 1.000 |  | 1.000 | 0.221 | **0.019** | 1.000 | 1.000 | 1.000 |  | 1.000 | 1.000 | 1.000 | 1.000 | 1.000 | 1.000 |
| OTU_545 | 0.156 | 0.078 | 0.330 | 1.000 | 1.000 | 1.000 |  | 0.156 | **0.036** | 1.000 | 1.000 | 1.000 | 1.000 |  | 1.000 | 1.000 | 1.000 | 1.000 | 1.000 | 1.000 |
| OTU_5450 | 1.000 | 1.000 | 1.000 | 1.000 | 1.000 | 1.000 |  | 1.000 | 1.000 | 0.910 | 0.494 | 0.909 | 1.000 |  | 1.000 | 1.000 | 1.000 | 1.000 | 1.000 | 1.000 |
| OTU_5451 | **0.002** | **< 0.001** | **0.005** | 0.672 | 1.000 | 0.253 |  | **0.002** | 0.073 | 0.135 | 1.000 | 1.000 | 1.000 |  | 0.380 | 0.189 | 0.089 | 1.000 | 1.000 | 1.000 |
| OTU_5457 | 1.000 | 1.000 | 0.968 | 1.000 | 0.230 | 1.000 |  | 1.000 | 0.741 | 0.318 | 1.000 | 1.000 | 1.000 |  | 1.000 | 1.000 | 0.258 | 1.000 | 1.000 | 1.000 |
| OTU_546 | **0.006** | **0.029** | 1.000 | 1.000 | **0.021** | 0.286 |  | **0.006** | 0.120 | 1.000 | 1.000 | 0.104 | 0.476 |  | 1.000 | **0.039** | 1.000 | 1.000 | 1.000 | 0.944 |
| OTU_5464 | 1.000 | 1.000 | 1.000 | 1.000 | 1.000 | 1.000 |  | 1.000 | 1.000 | 1.000 | 1.000 | 0.946 | 1.000 |  | 1.000 | 1.000 | 1.000 | 1.000 | 1.000 | 1.000 |
| OTU_5469 | **0.006** | **0.028** | 0.528 | 1.000 | 1.000 | 1.000 |  | **0.006** | **< 0.001** | 0.746 | 0.122 | 1.000 | **0.022** |  | 0.195 | **< 0.001** | 1.000 | 1.000 | 1.000 | 0.109 |
| OTU_547 | 1.000 | 1.000 | 1.000 | 1.000 | 1.000 | 1.000 |  | 1.000 | 1.000 | 1.000 | 1.000 | 1.000 | 1.000 |  | 1.000 | 1.000 | 1.000 | 1.000 | 1.000 | 1.000 |
| OTU_5475 | 1.000 | **0.016** | 1.000 | 0.396 | 1.000 | 1.000 |  | 1.000 | 1.000 | 1.000 | 1.000 | 1.000 | 1.000 |  | 1.000 | 1.000 | 1.000 | 1.000 | 1.000 | 1.000 |
| OTU_548 | 1.000 | 1.000 | 0.968 | 1.000 | 1.000 | 1.000 |  | 1.000 | 1.000 | 1.000 | 1.000 | 1.000 | 1.000 |  | 1.000 | 1.000 | 1.000 | 0.519 | 1.000 | 1.000 |
| OTU_5485 | 0.666 | **0.029** | 0.071 | 1.000 | 1.000 | 1.000 |  | 0.666 | 1.000 | 0.997 | 1.000 | 1.000 | 1.000 |  | 1.000 | 1.000 | 1.000 | 1.000 | 1.000 | 1.000 |
| OTU_549 | 0.518 | **< 0.001** | **0.004** | **0.003** | 1.000 | 0.516 |  | 0.518 | **< 0.001** | **0.006** | **< 0.001** | **0.008** | 0.273 |  | 1.000 | **0.016** | 1.000 | 0.570 | 1.000 | 1.000 |
| OTU_5492 | 1.000 | 1.000 | 1.000 | 1.000 | 1.000 | 1.000 |  | 1.000 | 0.069 | 1.000 | 1.000 | 1.000 | 1.000 |  | 1.000 | 0.111 | 1.000 | 0.723 | 1.000 | 1.000 |
| OTU_5496 | 1.000 | 0.426 | 1.000 | 1.000 | 1.000 | 1.000 |  | 1.000 | 1.000 | 1.000 | 1.000 | 1.000 | 1.000 |  | 1.000 | 1.000 | 1.000 | 1.000 | 1.000 | 1.000 |
| OTU_55 | 0.089 | 0.381 | **< 0.001** | **< 0.001** | **< 0.001** | **< 0.001** |  | 0.089 | **0.012** | **< 0.001** | **< 0.001** | **< 0.001** | **< 0.001** |  | 1.000 | **0.002** | **< 0.001** | **< 0.001** | **< 0.001** | **< 0.001** |
| OTU_550 | 1.000 | 1.000 | **0.009** | 1.000 | **0.006** | **0.034** |  | 1.000 | 1.000 | 0.097 | 1.000 | 0.213 | **0.011** |  | 1.000 | 1.000 | **< 0.001** | 1.000 | **< 0.001** | **0.028** |
| OTU_5503 | 1.000 | 1.000 | 0.168 | 1.000 | **0.009** | 1.000 |  | 1.000 | 1.000 | **0.038** | 1.000 | **0.041** | 1.000 |  | 1.000 | 1.000 | 0.194 | 1.000 | **0.006** | 1.000 |
| OTU_551 | 1.000 | **< 0.001** | 1.000 | **< 0.001** | 1.000 | **< 0.001** |  | 1.000 | **< 0.001** | **< 0.001** | **< 0.001** | **0.003** | **0.004** |  | 1.000 | **< 0.001** | 1.000 | **< 0.001** | 1.000 | **0.002** |
| OTU_5513 | 1.000 | 1.000 | 1.000 | 0.736 | 1.000 | 1.000 |  | 1.000 | 1.000 | 0.476 | 1.000 | 0.320 | 1.000 |  | 1.000 | 1.000 | 1.000 | 1.000 | 1.000 | 1.000 |
| OTU_552 | 1.000 | 0.075 | 1.000 | 0.467 | 1.000 | 1.000 |  | 1.000 | **0.009** | 1.000 | 1.000 | **0.006** | 0.058 |  | 1.000 | 0.099 | 1.000 | 1.000 | 1.000 | 0.287 |
| OTU_553 | 1.000 | 0.171 | 0.469 | 1.000 | **0.012** | **< 0.001** |  | 1.000 | 1.000 | 0.827 | 1.000 | **0.005** | 0.076 |  | 1.000 | 1.000 | 0.632 | 1.000 | 0.393 | 0.398 |
| OTU_5532 | 0.556 | 1.000 | 1.000 | 1.000 | 1.000 | 1.000 |  | 0.556 | 1.000 | 1.000 | 1.000 | 1.000 | 1.000 |  | 1.000 | 0.816 | **0.003** | 0.188 | **< 0.001** | 1.000 |
| OTU_554 | 0.729 | **0.032** | 0.069 | 1.000 | 1.000 | 1.000 |  | 0.729 | **< 0.001** | **< 0.001** | **0.041** | **0.044** | 1.000 |  | 1.000 | 0.289 | 0.226 | 1.000 | 1.000 | 1.000 |
| OTU_5547 | 1.000 | **0.002** | **0.005** | 0.715 | 0.912 | 1.000 |  | 1.000 | **< 0.001** | **< 0.001** | 0.090 | 0.083 | 1.000 |  | 0.619 | **< 0.001** | **0.001** | 0.445 | 1.000 | 1.000 |
| OTU_5549 | 1.000 | 1.000 | 1.000 | 1.000 | 1.000 | 1.000 |  | 1.000 | 1.000 | 1.000 | 1.000 | 1.000 | 1.000 |  | 1.000 | 1.000 | 0.405 | 1.000 | 1.000 | 1.000 |
| OTU_555 | 1.000 | 1.000 | 0.176 | 1.000 | 1.000 | 0.370 |  | 1.000 | **0.004** | **< 0.001** | **0.007** | **< 0.001** | 1.000 |  | 1.000 | 1.000 | 0.173 | **0.035** | **0.002** | 1.000 |
| OTU_5559 | **< 0.001** | **< 0.001** | 0.185 | 1.000 | 0.940 | **0.043** |  | **< 0.001** | **< 0.001** | **0.017** | 1.000 | 1.000 | 0.365 |  | **0.027** | **< 0.001** | 0.233 | 1.000 | 1.000 | 0.210 |
| OTU_556 | 0.725 | 1.000 | 1.000 | 0.097 | 1.000 | 0.958 |  | 0.725 | 0.773 | 1.000 | 0.912 | 1.000 | 1.000 |  | 1.000 | 1.000 | 1.000 | 1.000 | 1.000 | 1.000 |
| OTU_5562 | 1.000 | 1.000 | 1.000 | 1.000 | 1.000 | 1.000 |  | 1.000 | 1.000 | 0.060 | 1.000 | 0.403 | 1.000 |  | 1.000 | 1.000 | 1.000 | 1.000 | 1.000 | 1.000 |
| OTU_5566 | 1.000 | 1.000 | 1.000 | 1.000 | 1.000 | 1.000 |  | 1.000 | 1.000 | 1.000 | 1.000 | 1.000 | 1.000 |  | 1.000 | 1.000 | 1.000 | 1.000 | 1.000 | 1.000 |
| OTU_557 | **< 0.001** | **< 0.001** | **0.029** | 1.000 | 1.000 | 1.000 |  | **< 0.001** | 1.000 | 1.000 | **0.003** | **< 0.001** | 1.000 |  | 1.000 | 1.000 | 1.000 | 1.000 | 1.000 | 1.000 |
| OTU_558 | 0.666 | **0.007** | **0.002** | 1.000 | 1.000 | 1.000 |  | 0.666 | **0.004** | **0.029** | 1.000 | 1.000 | 1.000 |  | 1.000 | **0.003** | **< 0.001** | 0.651 | 0.117 | 1.000 |
| OTU_559 | 1.000 | 1.000 | 1.000 | 1.000 | 1.000 | 1.000 |  | 1.000 | **0.008** | 0.611 | 1.000 | 1.000 | 1.000 |  | 1.000 | 0.951 | 1.000 | 1.000 | 1.000 | 1.000 |
| OTU_5597 | 1.000 | 1.000 | 1.000 | 1.000 | 1.000 | 1.000 |  | 1.000 | 1.000 | 1.000 | 1.000 | 1.000 | 1.000 |  | 1.000 | 1.000 | 1.000 | 1.000 | 0.970 | 1.000 |
| OTU_56 | 1.000 | **0.020** | **< 0.001** | **0.003** | **< 0.001** | **< 0.001** |  | 1.000 | 1.000 | **< 0.001** | 0.329 | **< 0.001** | **< 0.001** |  | 1.000 | 1.000 | **< 0.001** | 1.000 | **< 0.001** | **< 0.001** |
| OTU_560 | 1.000 | 1.000 | **0.003** | 1.000 | **0.038** | **0.005** |  | 1.000 | 1.000 | **0.002** | 1.000 | 0.407 | **0.012** |  | 1.000 | 1.000 | 1.000 | 1.000 | 1.000 | 0.058 |
| OTU_561 | 0.134 | 0.659 | 1.000 | 1.000 | 0.312 | 1.000 |  | 0.134 | 1.000 | **0.030** | 1.000 | 1.000 | 1.000 |  | 1.000 | 1.000 | 1.000 | 1.000 | 1.000 | 1.000 |
| OTU_562 | 1.000 | 1.000 | 1.000 | 0.152 | 0.077 | 1.000 |  | 1.000 | 0.142 | **0.027** | 1.000 | 0.459 | 1.000 |  | 1.000 | 0.209 | 0.358 | 1.000 | 1.000 | 1.000 |
| OTU_5626 | 1.000 | 0.958 | 1.000 | 1.000 | 1.000 | 1.000 |  | 1.000 | 1.000 | 1.000 | 1.000 | 1.000 | 1.000 |  | 1.000 | 0.891 | 0.111 | 1.000 | 0.555 | 1.000 |
| OTU_563 | 1.000 | 0.687 | **0.027** | 1.000 | 1.000 | 1.000 |  | 1.000 | **0.024** | 0.247 | 0.302 | 1.000 | 1.000 |  | 1.000 | 0.557 | 0.877 | 1.000 | 1.000 | 1.000 |
| OTU_5631 | 1.000 | **< 0.001** | **< 0.001** | 0.140 | **< 0.001** | 1.000 |  | 1.000 | **< 0.001** | **< 0.001** | 0.175 | **< 0.001** | 1.000 |  | 1.000 | **< 0.001** | **< 0.001** | 0.087 | **0.005** | 1.000 |
| OTU_5632 | 1.000 | 1.000 | 1.000 | 1.000 | 1.000 | 1.000 |  | 1.000 | 1.000 | 1.000 | 1.000 | 1.000 | 1.000 |  | 1.000 | 1.000 | 1.000 | 1.000 | 1.000 | 1.000 |
| OTU_564 | 1.000 | 1.000 | **< 0.001** | 1.000 | **< 0.001** | **< 0.001** |  | 1.000 | 1.000 | 1.000 | 0.175 | **0.041** | 1.000 |  | 1.000 | 1.000 | **< 0.001** | 1.000 | **< 0.001** | **< 0.001** |
| OTU_5642 | **< 0.001** | **< 0.001** | **< 0.001** | **< 0.001** | **0.011** | 1.000 |  | **< 0.001** | **< 0.001** | **< 0.001** | 0.182 | **0.009** | 1.000 |  | **0.007** | **< 0.001** | **< 0.001** | **< 0.001** | **< 0.001** | 1.000 |
| OTU_5644 | 1.000 | 1.000 | 1.000 | 1.000 | 1.000 | 1.000 |  | 1.000 | 1.000 | 1.000 | 0.448 | 0.063 | 1.000 |  | 1.000 | 1.000 | 0.508 | 1.000 | 0.228 | 1.000 |
| OTU_565 | 1.000 | 1.000 | 1.000 | 1.000 | 1.000 | 1.000 |  | 1.000 | 1.000 | 0.171 | 1.000 | 1.000 | 1.000 |  | 1.000 | 1.000 | 1.000 | 1.000 | 1.000 | 1.000 |
| OTU_5650 | 1.000 | 1.000 | 1.000 | 1.000 | 1.000 | 1.000 |  | 1.000 | 1.000 | 1.000 | 1.000 | 1.000 | 1.000 |  | 1.000 | 1.000 | 1.000 | 1.000 | 1.000 | 1.000 |
| OTU_5653 | 1.000 | **< 0.001** | 1.000 | **0.007** | 1.000 | 0.279 |  | 1.000 | 0.415 | 1.000 | **0.045** | 1.000 | 1.000 |  | 0.056 | **< 0.001** | **0.001** | 1.000 | 1.000 | 1.000 |
| OTU_5656 | 1.000 | 1.000 | 1.000 | 1.000 | 1.000 | 1.000 |  | 1.000 | **0.006** | **< 0.001** | 1.000 | 1.000 | 1.000 |  | 1.000 | 1.000 | 1.000 | 1.000 | 1.000 | 1.000 |
| OTU_566 | 1.000 | 0.655 | **0.045** | 1.000 | 1.000 | 1.000 |  | 1.000 | 1.000 | 1.000 | 1.000 | 1.000 | 1.000 |  | 1.000 | 0.273 | **0.024** | 1.000 | 0.535 | 1.000 |
| OTU_5661 | 1.000 | **0.007** | 1.000 | **0.005** | 1.000 | **0.007** |  | 1.000 | **< 0.001** | 0.161 | **< 0.001** | 0.188 | **0.019** |  | 1.000 | 0.051 | 1.000 | 0.078 | 1.000 | 0.067 |
| OTU_5663 | 1.000 | 1.000 | 1.000 | 1.000 | 1.000 | 1.000 |  | 1.000 | 1.000 | 1.000 | 1.000 | 1.000 | 1.000 |  | 1.000 | 1.000 | 1.000 | 1.000 | 1.000 | 1.000 |
| OTU_567 | 0.399 | **< 0.001** | 1.000 | 0.175 | 1.000 | **0.023** |  | 0.399 | **0.001** | 1.000 | **0.002** | 1.000 | **0.024** |  | 1.000 | 0.052 | 1.000 | 0.066 | 1.000 | 0.068 |
| OTU_5671 | 0.787 | 1.000 | 0.059 | 1.000 | 1.000 | 1.000 |  | 0.787 | 1.000 | 0.222 | 1.000 | 1.000 | 1.000 |  | 1.000 | 1.000 | 1.000 | 0.710 | 0.422 | 1.000 |
| OTU_568 | 1.000 | 1.000 | 0.122 | 1.000 | **0.012** | **0.001** |  | 1.000 | 1.000 | **< 0.001** | 1.000 | **0.013** | **0.037** |  | 1.000 | 1.000 | **0.008** | 1.000 | **0.002** | 0.084 |
| OTU_5681 | 0.256 | 1.000 | 1.000 | 0.242 | **0.013** | 1.000 |  | 0.256 | 0.207 | **0.040** | 1.000 | 1.000 | 1.000 |  | 1.000 | 1.000 | 1.000 | 1.000 | 1.000 | 1.000 |
| OTU_5689 | 1.000 | 1.000 | 1.000 | 1.000 | 1.000 | 1.000 |  | 1.000 | 1.000 | 1.000 | 1.000 | 1.000 | 1.000 |  | 1.000 | 1.000 | 1.000 | 1.000 | 1.000 | 1.000 |
| OTU_569 | 1.000 | **0.049** | 0.313 | 1.000 | 1.000 | 1.000 |  | 1.000 | 0.113 | 0.653 | 1.000 | 1.000 | 1.000 |  | 1.000 | 1.000 | 1.000 | 1.000 | 1.000 | 1.000 |
| OTU_57 | **< 0.001** | **< 0.001** | **< 0.001** | **< 0.001** | 1.000 | **< 0.001** |  | **< 0.001** | **< 0.001** | **< 0.001** | **< 0.001** | 1.000 | **< 0.001** |  | **< 0.001** | **< 0.001** | **< 0.001** | **< 0.001** | 1.000 | **< 0.001** |
| OTU_570 | 1.000 | 1.000 | 0.629 | **0.047** | **0.007** | 1.000 |  | 1.000 | 0.210 | 1.000 | 1.000 | 1.000 | 1.000 |  | 1.000 | 1.000 | 1.000 | 1.000 | 0.835 | 1.000 |
| OTU_5704 | 1.000 | 1.000 | 1.000 | 1.000 | 1.000 | 1.000 |  | 1.000 | **< 0.001** | **< 0.001** | 0.068 | 1.000 | 1.000 |  | 1.000 | **< 0.001** | **< 0.001** | **0.019** | 0.373 | 1.000 |
| OTU_571 | 1.000 | 1.000 | 1.000 | 1.000 | 1.000 | 1.000 |  | 1.000 | 1.000 | 0.409 | 0.905 | 0.079 | 1.000 |  | 1.000 | 1.000 | 1.000 | 1.000 | 1.000 | 1.000 |
| OTU_572 | 1.000 | 1.000 | 1.000 | 0.453 | 1.000 | 1.000 |  | 1.000 | 1.000 | 1.000 | 1.000 | 1.000 | 1.000 |  | 1.000 | 1.000 | 1.000 | 1.000 | 1.000 | 1.000 |
| OTU_5724 | **0.012** | 1.000 | **< 0.001** | **0.001** | **< 0.001** | **< 0.001** |  | **0.012** | 0.231 | **< 0.001** | **0.002** | **< 0.001** | **0.018** |  | 0.422 | 1.000 | **< 0.001** | 0.145 | **< 0.001** | **< 0.001** |
| OTU_573 | 1.000 | 0.594 | 1.000 | 1.000 | 1.000 | 1.000 |  | 1.000 | 0.249 | 1.000 | 0.905 | 1.000 | 1.000 |  | 1.000 | 1.000 | 1.000 | 1.000 | 1.000 | 1.000 |
| OTU_5730 | 0.419 | 1.000 | 0.376 | 1.000 | 1.000 | 1.000 |  | 0.419 | **< 0.001** | **< 0.001** | 1.000 | 1.000 | 1.000 |  | 0.210 | 0.366 | 1.000 | 1.000 | 1.000 | 1.000 |
| OTU_5738 | 0.264 | **< 0.001** | 1.000 | 1.000 | 1.000 | 0.064 |  | 0.264 | 0.234 | 1.000 | 1.000 | 1.000 | 0.130 |  | 1.000 | 0.178 | 1.000 | 1.000 | 1.000 | 0.638 |
| OTU_574 | 1.000 | 0.172 | 1.000 | 0.453 | 1.000 | 0.295 |  | 1.000 | 1.000 | 1.000 | 1.000 | 1.000 | 1.000 |  | 1.000 | 1.000 | 1.000 | 1.000 | 1.000 | 1.000 |
| OTU_575 | 1.000 | 1.000 | 1.000 | 0.085 | 1.000 | 1.000 |  | 1.000 | 1.000 | 0.566 | 1.000 | 1.000 | 1.000 |  | 1.000 | 1.000 | 1.000 | 1.000 | 1.000 | 1.000 |
| OTU_5754 | 1.000 | 1.000 | **0.004** | 1.000 | 0.078 | 0.087 |  | 1.000 | 1.000 | 1.000 | 1.000 | 1.000 | 1.000 |  | 1.000 | 1.000 | **0.015** | 1.000 | 0.404 | 1.000 |
| OTU_576 | 1.000 | 1.000 | 0.283 | 1.000 | 1.000 | 1.000 |  | 1.000 | **0.003** | **< 0.001** | 1.000 | 0.997 | 1.000 |  | 1.000 | 1.000 | 1.000 | 1.000 | 1.000 | 1.000 |
| OTU_5761 | 1.000 | 1.000 | 1.000 | 1.000 | 1.000 | 1.000 |  | 1.000 | 1.000 | 1.000 | 1.000 | 1.000 | 1.000 |  | 1.000 | 1.000 | 1.000 | 1.000 | 1.000 | 1.000 |
| OTU_577 | 1.000 | 1.000 | 1.000 | 1.000 | 1.000 | 1.000 |  | 1.000 | 1.000 | 1.000 | 0.330 | **0.034** | 1.000 |  | 1.000 | 1.000 | 1.000 | 1.000 | 1.000 | 1.000 |
| OTU_5772 | 0.080 | 0.053 | **0.045** | 1.000 | 1.000 | 1.000 |  | 0.080 | **0.005** | **< 0.001** | 1.000 | 1.000 | 1.000 |  | **0.031** | 0.123 | **0.007** | 1.000 | 1.000 | 1.000 |
| OTU_578 | 0.516 | 0.368 | 0.313 | 1.000 | 1.000 | 1.000 |  | 0.516 | 0.110 | **0.003** | 1.000 | 0.093 | 1.000 |  | 1.000 | 1.000 | 1.000 | 1.000 | 1.000 | 1.000 |
| OTU_5781 | **< 0.001** | **< 0.001** | **0.004** | 1.000 | 1.000 | 1.000 |  | **< 0.001** | 1.000 | 1.000 | 1.000 | 1.000 | 1.000 |  | **0.045** | **0.022** | **0.010** | 1.000 | 1.000 | 1.000 |
| OTU_5787 | 1.000 | 0.078 | 1.000 | 1.000 | 1.000 | 1.000 |  | 1.000 | 0.711 | 0.128 | 1.000 | 1.000 | 1.000 |  | 1.000 | 0.052 | 1.000 | 1.000 | 1.000 | 1.000 |
| OTU_5788 | 1.000 | 1.000 | 1.000 | 1.000 | 1.000 | 1.000 |  | 1.000 | 1.000 | 1.000 | 1.000 | 1.000 | 1.000 |  | 1.000 | 1.000 | 1.000 | 1.000 | 1.000 | 1.000 |
| OTU_579 | 1.000 | 1.000 | 1.000 | 1.000 | 0.141 | 1.000 |  | 1.000 | 1.000 | 1.000 | 1.000 | 0.051 | 1.000 |  | 1.000 | 1.000 | 1.000 | 1.000 | 1.000 | 1.000 |
| OTU_58 | **< 0.001** | **< 0.001** | **< 0.001** | **0.042** | 1.000 | **< 0.001** |  | **< 0.001** | **< 0.001** | **< 0.001** | **0.001** | 1.000 | **< 0.001** |  | **< 0.001** | **< 0.001** | **< 0.001** | **< 0.001** | 1.000 | **< 0.001** |
| OTU_580 | 0.098 | **< 0.001** | **< 0.001** | 1.000 | **0.037** | 1.000 |  | 0.098 | **< 0.001** | **< 0.001** | **0.012** | 1.000 | 0.134 |  | 1.000 | **< 0.001** | **< 0.001** | 0.119 | 0.531 | 1.000 |
| OTU_581 | 1.000 | 0.100 | **0.025** | **0.018** | **0.002** | 1.000 |  | 1.000 | **0.038** | **< 0.001** | 0.052 | **< 0.001** | 1.000 |  | 0.352 | **< 0.001** | **< 0.001** | 1.000 | 0.576 | 1.000 |
| OTU_5814 | 1.000 | 1.000 | 0.346 | 1.000 | 1.000 | 1.000 |  | 1.000 | 1.000 | 1.000 | 1.000 | 1.000 | 1.000 |  | 1.000 | 0.991 | 1.000 | 1.000 | 1.000 | 1.000 |
| OTU_582 | 1.000 | 1.000 | **< 0.001** | 1.000 | **< 0.001** | **< 0.001** |  | 1.000 | 1.000 | **0.010** | 1.000 | 1.000 | **0.041** |  | 1.000 | 1.000 | **0.002** | 1.000 | **0.003** | **0.001** |
| OTU_583 | 1.000 | **< 0.001** | **0.026** | **0.026** | 1.000 | 1.000 |  | 1.000 | **< 0.001** | 0.109 | **< 0.001** | 0.127 | 0.725 |  | 1.000 | **0.019** | 1.000 | 0.174 | 1.000 | 1.000 |
| OTU_584 | 1.000 | 1.000 | 1.000 | 0.093 | **0.047** | 1.000 |  | 1.000 | 1.000 | 1.000 | **0.022** | **0.045** | 1.000 |  | 1.000 | 1.000 | 1.000 | 1.000 | 1.000 | 1.000 |
| OTU_585 | 1.000 | **0.007** | **< 0.001** | 1.000 | 0.089 | 1.000 |  | 1.000 | **0.007** | **0.003** | 1.000 | 1.000 | 1.000 |  | 0.065 | 0.084 | **0.024** | 1.000 | 1.000 | 1.000 |
| OTU_586 | 1.000 | **0.015** | **0.006** | **0.027** | **0.007** | 1.000 |  | 1.000 | **< 0.001** | **< 0.001** | **0.011** | **< 0.001** | 1.000 |  | 1.000 | 0.471 | **< 0.001** | 1.000 | **< 0.001** | 0.168 |
| OTU_587 | 1.000 | **0.008** | **0.030** | **0.003** | **0.008** | 1.000 |  | 1.000 | **< 0.001** | **< 0.001** | **0.036** | **< 0.001** | 1.000 |  | 1.000 | 1.000 | 1.000 | 1.000 | 0.532 | 1.000 |
| OTU_588 | 1.000 | 1.000 | 1.000 | 1.000 | 1.000 | 1.000 |  | 1.000 | 1.000 | 1.000 | 1.000 | 1.000 | 1.000 |  | 1.000 | 1.000 | 1.000 | 1.000 | 0.174 | 1.000 |
| OTU_5885 | 1.000 | **< 0.001** | **< 0.001** | **< 0.001** | **< 0.001** | 1.000 |  | 1.000 | **< 0.001** | **< 0.001** | **0.002** | **0.017** | 1.000 |  | 1.000 | **0.001** | **< 0.001** | 0.702 | **0.006** | 1.000 |
| OTU_5886 | 1.000 | 0.659 | 1.000 | 1.000 | 1.000 | 1.000 |  | 1.000 | **0.003** | 1.000 | **0.022** | 1.000 | **0.013** |  | 0.960 | 1.000 | 1.000 | 1.000 | **0.016** | 0.651 |
| OTU_5889 | 1.000 | 1.000 | 0.197 | 1.000 | 0.306 | 1.000 |  | 1.000 | 0.141 | 1.000 | 1.000 | **0.004** | 0.473 |  | 1.000 | 1.000 | 0.245 | 1.000 | 0.391 | 0.153 |
| OTU_59 | 0.340 | 1.000 | **< 0.001** | **< 0.001** | **< 0.001** | **< 0.001** |  | 0.340 | 0.355 | **< 0.001** | **0.002** | **< 0.001** | **< 0.001** |  | 1.000 | **0.002** | **< 0.001** | **< 0.001** | **< 0.001** | **0.008** |
| OTU_590 | 1.000 | 1.000 | 1.000 | 1.000 | 0.554 | 1.000 |  | 1.000 | **0.004** | 0.149 | 1.000 | 1.000 | 1.000 |  | 0.561 | **0.003** | **0.010** | 1.000 | 1.000 | 1.000 |
| OTU_5902 | 1.000 | 1.000 | 0.383 | 1.000 | **0.010** | 1.000 |  | 1.000 | 1.000 | 0.116 | 1.000 | **0.030** | 1.000 |  | 1.000 | 0.373 | **0.001** | 1.000 | **0.006** | 1.000 |
| OTU_5908 | 1.000 | **< 0.001** | 0.116 | **< 0.001** | **< 0.001** | 1.000 |  | 1.000 | **< 0.001** | **< 0.001** | **< 0.001** | **< 0.001** | 0.080 |  | 1.000 | **< 0.001** | 0.067 | **< 0.001** | **< 0.001** | 1.000 |
| OTU_5909 | 0.622 | 1.000 | 1.000 | **0.007** | 1.000 | 1.000 |  | 0.622 | 0.762 | 0.807 | 0.166 | 0.140 | 1.000 |  | 1.000 | 1.000 | 1.000 | 1.000 | 1.000 | 1.000 |
| OTU_591 | 1.000 | 0.850 | 0.915 | 1.000 | 1.000 | 1.000 |  | 1.000 | 1.000 | 1.000 | 1.000 | 1.000 | 1.000 |  | 1.000 | 0.654 | 0.250 | 1.000 | 1.000 | 1.000 |
| OTU_5914 | 0.074 | **0.003** | **0.003** | 1.000 | 1.000 | 1.000 |  | 0.074 | 1.000 | 1.000 | 0.991 | 1.000 | 1.000 |  | 1.000 | 1.000 | 1.000 | 1.000 | 1.000 | 1.000 |
| OTU_592 | 1.000 | 1.000 | 1.000 | 0.384 | 1.000 | 1.000 |  | 1.000 | 1.000 | 1.000 | 1.000 | 1.000 | 1.000 |  | 1.000 | 1.000 | 1.000 | 1.000 | 1.000 | 1.000 |
| OTU_593 | 1.000 | **< 0.001** | **0.008** | **< 0.001** | **< 0.001** | 1.000 |  | 1.000 | **< 0.001** | **< 0.001** | **< 0.001** | **0.003** | 0.063 |  | 1.000 | **< 0.001** | 1.000 | **< 0.001** | 1.000 | **0.014** |
| OTU_594 | 1.000 | 1.000 | 0.461 | 1.000 | **0.001** | 0.055 |  | 1.000 | 1.000 | **0.008** | 1.000 | **0.031** | **< 0.001** |  | 1.000 | 1.000 | 0.113 | 1.000 | 0.622 | 0.894 |
| OTU_595 | 1.000 | 0.183 | **< 0.001** | 0.079 | **< 0.001** | 1.000 |  | 1.000 | 0.773 | 0.310 | 1.000 | 1.000 | 1.000 |  | 0.723 | 0.074 | **< 0.001** | 1.000 | 0.312 | 1.000 |
| OTU_5952 | **0.002** | **< 0.001** | **< 0.001** | 0.291 | 1.000 | 1.000 |  | **0.002** | **< 0.001** | **0.018** | **< 0.001** | 1.000 | **0.030** |  | 1.000 | **< 0.001** | **< 0.001** | **0.002** | 0.135 | 1.000 |
| OTU_5956 | 0.756 | **< 0.001** | **0.007** | 0.119 | 1.000 | 1.000 |  | 0.756 | **< 0.001** | **0.011** | **0.046** | 1.000 | 0.396 |  | 1.000 | **0.024** | 1.000 | 1.000 | 1.000 | 1.000 |
| OTU_596 | 1.000 | 1.000 | 1.000 | 1.000 | 1.000 | 1.000 |  | 1.000 | 1.000 | 1.000 | 1.000 | 1.000 | 1.000 |  | 1.000 | 1.000 | 1.000 | 1.000 | 1.000 | 1.000 |
| OTU_597 | 1.000 | 1.000 | **0.008** | 1.000 | 0.140 | 0.369 |  | 1.000 | 1.000 | 0.685 | 1.000 | 0.057 | 1.000 |  | 1.000 | 1.000 | 0.131 | 1.000 | 1.000 | 1.000 |
| OTU_598 | 1.000 | **0.033** | 0.954 | **0.002** | 0.076 | 1.000 |  | 1.000 | 0.746 | 1.000 | **0.031** | 1.000 | 0.567 |  | 1.000 | **0.007** | 1.000 | 0.121 | 1.000 | 0.944 |
| OTU_5987 | 0.292 | 1.000 | 0.786 | 1.000 | 1.000 | 1.000 |  | 0.292 | 0.069 | 0.063 | 1.000 | 1.000 | 1.000 |  | 0.803 | **0.035** | 0.079 | 1.000 | 1.000 | 1.000 |
| OTU_599 | 0.419 | **0.011** | 0.052 | 1.000 | 1.000 | 1.000 |  | 0.419 | 0.106 | 0.586 | 0.631 | 1.000 | 1.000 |  | 1.000 | 0.330 | 1.000 | 1.000 | 1.000 | 1.000 |
| OTU_5996 | 1.000 | 0.374 | 1.000 | **0.049** | 0.250 | 1.000 |  | 1.000 | 1.000 | 1.000 | 1.000 | 1.000 | 1.000 |  | 1.000 | 0.912 | 0.533 | 1.000 | 1.000 | 1.000 |
| OTU_6 | 1.000 | **< 0.001** | **0.004** | **< 0.001** | **0.002** | 0.644 |  | 1.000 | **< 0.001** | **< 0.001** | **< 0.001** | **0.001** | 0.313 |  | 0.202 | **< 0.001** | **< 0.001** | **< 0.001** | 1.000 | 0.089 |
| OTU_60 | **0.014** | **< 0.001** | **< 0.001** | **0.002** | **< 0.001** | 1.000 |  | **0.014** | **< 0.001** | **< 0.001** | 0.654 | 0.056 | 1.000 |  | **< 0.001** | **< 0.001** | **< 0.001** | **0.007** | 0.145 | 1.000 |
| OTU_600 | 0.700 | 0.084 | 0.201 | 1.000 | 1.000 | 1.000 |  | 0.700 | 0.326 | 1.000 | 1.000 | 0.147 | 1.000 |  | 1.000 | 0.167 | 1.000 | 1.000 | 1.000 | 1.000 |
| OTU_6009 | 1.000 | 0.160 | 0.158 | 1.000 | 1.000 | 1.000 |  | 1.000 | **< 0.001** | **< 0.001** | 1.000 | 1.000 | 1.000 |  | 1.000 | 0.108 | 0.737 | 1.000 | 1.000 | 1.000 |
| OTU_601 | 1.000 | 0.151 | 1.000 | 0.457 | 0.890 | **0.003** |  | 1.000 | 0.055 | **0.003** | 1.000 | 1.000 | 1.000 |  | 1.000 | 1.000 | 1.000 | 1.000 | 1.000 | 1.000 |
| OTU_6012 | 1.000 | 1.000 | 1.000 | 1.000 | 1.000 | 1.000 |  | 1.000 | 1.000 | 1.000 | 1.000 | 1.000 | 1.000 |  | 1.000 | 1.000 | 1.000 | 1.000 | 1.000 | 1.000 |
| OTU_602 | 1.000 | 1.000 | 0.264 | 1.000 | 0.747 | 1.000 |  | 1.000 | 1.000 | 1.000 | 1.000 | 1.000 | 1.000 |  | 1.000 | 1.000 | 0.340 | 1.000 | 0.185 | 1.000 |
| OTU_603 | 1.000 | 1.000 | 0.226 | 1.000 | 1.000 | 1.000 |  | 1.000 | 1.000 | 1.000 | 1.000 | 1.000 | 0.217 |  | 1.000 | 1.000 | 1.000 | 1.000 | 1.000 | 1.000 |
| OTU_6030 | 1.000 | 1.000 | 1.000 | 1.000 | 0.086 | 1.000 |  | 1.000 | 0.777 | 1.000 | 1.000 | **0.023** | 1.000 |  | 1.000 | 1.000 | 1.000 | 1.000 | 1.000 | 1.000 |
| OTU_604 | 1.000 | **0.016** | **0.003** | 0.233 | **0.035** | 1.000 |  | 1.000 | **0.014** | **0.016** | 0.071 | 0.063 | 1.000 |  | **< 0.001** | **< 0.001** | **< 0.001** | 1.000 | 1.000 | 1.000 |
| OTU_605 | 1.000 | 1.000 | 1.000 | 1.000 | 1.000 | 1.000 |  | 1.000 | 1.000 | 1.000 | 1.000 | 1.000 | 1.000 |  | 1.000 | 1.000 | 1.000 | 1.000 | 1.000 | 1.000 |
| OTU_606 | **0.014** | 0.989 | 1.000 | 1.000 | 0.052 | 1.000 |  | **0.014** | **0.010** | 1.000 | 0.486 | 1.000 | 1.000 |  | 1.000 | **0.039** | 1.000 | 0.750 | 1.000 | 0.225 |
| OTU_6061 | 0.451 | **0.049** | 1.000 | 1.000 | 1.000 | 1.000 |  | 0.451 | **0.020** | **0.033** | 1.000 | 0.394 | 1.000 |  | 1.000 | 1.000 | 1.000 | 1.000 | 1.000 | 1.000 |
| OTU_6063 | 0.288 | **< 0.001** | **< 0.001** | **0.012** | 0.281 | 1.000 |  | 0.288 | **< 0.001** | **< 0.001** | **< 0.001** | **0.040** | 1.000 |  | **0.025** | **< 0.001** | **< 0.001** | 0.780 | 0.092 | 1.000 |
| OTU_607 | 1.000 | 1.000 | 0.561 | 1.000 | 1.000 | 1.000 |  | 1.000 | **< 0.001** | **< 0.001** | 1.000 | 1.000 | 1.000 |  | 1.000 | 1.000 | 1.000 | 1.000 | 0.420 | 1.000 |
| OTU_608 | 1.000 | 0.547 | 1.000 | 1.000 | 1.000 | 1.000 |  | 1.000 | 0.217 | 1.000 | 1.000 | 1.000 | 1.000 |  | 0.380 | **0.002** | **0.017** | 1.000 | 1.000 | 1.000 |
| OTU_6082 | 1.000 | 0.716 | 1.000 | 1.000 | 1.000 | 1.000 |  | 1.000 | 0.226 | 0.788 | 0.260 | 0.731 | 1.000 |  | 1.000 | **0.016** | 0.506 | 0.439 | 1.000 | 1.000 |
| OTU_609 | **0.014** | **< 0.001** | **< 0.001** | 1.000 | 1.000 | 1.000 |  | **0.014** | **< 0.001** | **0.025** | **0.044** | 1.000 | 0.078 |  | 0.672 | **0.004** | 0.101 | 1.000 | 1.000 | 1.000 |
| OTU_6098 | 0.107 | 0.063 | 0.288 | 1.000 | 1.000 | 1.000 |  | 0.107 | 1.000 | 1.000 | 1.000 | 1.000 | 1.000 |  | 1.000 | 1.000 | 1.000 | 1.000 | 1.000 | 1.000 |
| OTU_61 | **< 0.001** | **< 0.001** | **< 0.001** | 0.063 | 1.000 | 1.000 |  | **< 0.001** | **< 0.001** | **< 0.001** | **0.002** | 1.000 | 0.066 |  | **< 0.001** | **< 0.001** | **0.002** | 0.731 | 1.000 | 0.074 |
| OTU_610 | 1.000 | 1.000 | 1.000 | 1.000 | 1.000 | 1.000 |  | 1.000 | **0.019** | 0.350 | 1.000 | 1.000 | 1.000 |  | 1.000 | 1.000 | 1.000 | 1.000 | 1.000 | 1.000 |
| OTU_6105 | 1.000 | 0.578 | 1.000 | 1.000 | 1.000 | 0.970 |  | 1.000 | 0.698 | 1.000 | 1.000 | 0.427 | 1.000 |  | 1.000 | 1.000 | 1.000 | 1.000 | 1.000 | 1.000 |
| OTU_611 | 0.507 | 1.000 | 1.000 | 1.000 | 1.000 | 1.000 |  | 0.507 | **0.005** | **0.012** | 1.000 | 1.000 | 1.000 |  | **0.020** | 0.282 | 1.000 | 1.000 | 0.110 | 1.000 |
| OTU_6117 | 1.000 | 1.000 | 1.000 | 0.689 | 1.000 | 1.000 |  | 1.000 | **0.037** | **0.032** | **0.011** | **0.007** | 1.000 |  | 1.000 | 0.119 | 1.000 | **0.003** | **0.034** | 1.000 |
| OTU_6119 | 1.000 | 1.000 | 1.000 | 1.000 | 0.410 | 0.306 |  | 1.000 | 1.000 | 1.000 | 1.000 | 0.054 | 1.000 |  | 1.000 | 1.000 | 1.000 | 1.000 | 0.993 | 1.000 |
| OTU_612 | 0.173 | **< 0.001** | **0.007** | 1.000 | 1.000 | 1.000 |  | 0.173 | **0.005** | **< 0.001** | 1.000 | 1.000 | 1.000 |  | **0.001** | **< 0.001** | **< 0.001** | 1.000 | 1.000 | 1.000 |
| OTU_6127 | 0.382 | 1.000 | 1.000 | 1.000 | 0.117 | 1.000 |  | 0.382 | 0.255 | 1.000 | 0.359 | 1.000 | 1.000 |  | 1.000 | 1.000 | 1.000 | 0.780 | 1.000 | 1.000 |
| OTU_613 | **0.017** | **0.002** | **< 0.001** | 1.000 | 1.000 | 1.000 |  | **0.017** | **0.004** | **0.011** | 0.097 | 0.170 | 1.000 |  | 1.000 | **0.003** | **< 0.001** | 0.242 | 0.080 | 1.000 |
| OTU_6130 | 0.558 | 0.217 | **0.005** | 1.000 | 1.000 | 1.000 |  | 0.558 | **0.018** | 0.055 | 1.000 | 1.000 | 1.000 |  | 1.000 | 0.427 | 0.506 | 1.000 | 1.000 | 1.000 |
| OTU_6131 | 0.107 | 0.177 | 0.290 | 1.000 | 1.000 | 1.000 |  | 0.107 | 0.227 | 0.571 | 1.000 | 1.000 | 1.000 |  | 1.000 | 1.000 | 1.000 | 1.000 | 1.000 | 1.000 |
| OTU_614 | 0.084 | **0.002** | 0.392 | 1.000 | 1.000 | 1.000 |  | 0.084 | 1.000 | 1.000 | 1.000 | 0.658 | 0.490 |  | 1.000 | 0.479 | 1.000 | 1.000 | 1.000 | 1.000 |
| OTU_6145 | 1.000 | 1.000 | 1.000 | 1.000 | 1.000 | 1.000 |  | 1.000 | 0.131 | 1.000 | 1.000 | 1.000 | 1.000 |  | 1.000 | 1.000 | 1.000 | 1.000 | 1.000 | 1.000 |
| OTU_615 | 1.000 | 1.000 | 1.000 | 1.000 | 1.000 | 1.000 |  | 1.000 | 1.000 | 1.000 | 1.000 | 1.000 | 1.000 |  | 1.000 | 1.000 | 1.000 | 1.000 | 1.000 | 1.000 |
| OTU_616 | **< 0.001** | **0.017** | **0.004** | 0.457 | 0.965 | 1.000 |  | **< 0.001** | 0.137 | 0.959 | 0.838 | 0.093 | 1.000 |  | 0.426 | 0.071 | 0.517 | 1.000 | 1.000 | 1.000 |
| OTU_6166 | **< 0.001** | **< 0.001** | **< 0.001** | **0.003** | 0.090 | 1.000 |  | **< 0.001** | **< 0.001** | **< 0.001** | **0.042** | **0.011** | 1.000 |  | 0.347 | **< 0.001** | **< 0.001** | **0.021** | **0.004** | 1.000 |
| OTU_617 | 0.650 | **< 0.001** | **< 0.001** | 0.418 | 0.531 | 1.000 |  | 0.650 | **< 0.001** | **0.002** | 0.295 | 1.000 | 1.000 |  | 0.117 | **< 0.001** | **0.003** | 0.564 | 1.000 | 1.000 |
| OTU_6173 | 1.000 | **0.004** | **0.030** | 0.124 | 0.549 | 1.000 |  | 1.000 | **< 0.001** | **< 0.001** | 1.000 | 1.000 | 1.000 |  | 1.000 | 0.092 | 0.052 | 1.000 | 0.990 | 1.000 |
| OTU_618 | 1.000 | 1.000 | **< 0.001** | 0.785 | **< 0.001** | **0.010** |  | 1.000 | 1.000 | **< 0.001** | 1.000 | 1.000 | **0.021** |  | 1.000 | 1.000 | **< 0.001** | 1.000 | **0.027** | **0.047** |
| OTU_6184 | **0.014** | 0.067 | 0.817 | 1.000 | 1.000 | 1.000 |  | **0.014** | 1.000 | 0.484 | 1.000 | 1.000 | 1.000 |  | 1.000 | 1.000 | 1.000 | 1.000 | 1.000 | 1.000 |
| OTU_6185 | 1.000 | 0.858 | 1.000 | **0.013** | **0.019** | 1.000 |  | 1.000 | 1.000 | 1.000 | **0.026** | 0.284 | 1.000 |  | 1.000 | **0.011** | 1.000 | 0.319 | 1.000 | 1.000 |
| OTU_619 | 1.000 | 1.000 | 1.000 | 1.000 | 0.285 | 1.000 |  | 1.000 | 1.000 | 1.000 | 1.000 | 0.299 | 1.000 |  | 1.000 | **0.004** | 1.000 | 0.220 | 1.000 | **0.047** |
| OTU_62 | 1.000 | 1.000 | 1.000 | 1.000 | 1.000 | 1.000 |  | 1.000 | 0.230 | 0.261 | 1.000 | 1.000 | 1.000 |  | 1.000 | 1.000 | 1.000 | 1.000 | 1.000 | 1.000 |
| OTU_620 | 1.000 | 0.702 | **< 0.001** | **0.005** | **< 0.001** | **0.017** |  | 1.000 | 0.287 | 0.233 | 1.000 | **< 0.001** | **< 0.001** |  | 1.000 | 0.053 | 1.000 | 1.000 | 1.000 | 0.109 |
| OTU_6207 | 1.000 | 1.000 | 1.000 | 1.000 | 1.000 | 1.000 |  | 1.000 | 1.000 | 1.000 | 1.000 | 1.000 | 1.000 |  | 1.000 | 1.000 | 1.000 | 0.081 | 0.203 | 1.000 |
| OTU_621 | 1.000 | 1.000 | **0.046** | 1.000 | **0.037** | 1.000 |  | 1.000 | 0.271 | **< 0.001** | 1.000 | 0.150 | 1.000 |  | 1.000 | 0.690 | 0.058 | 1.000 | 0.077 | 1.000 |
| OTU_622 | **0.034** | **0.032** | 0.050 | 1.000 | 1.000 | 1.000 |  | **0.034** | **0.003** | **0.042** | 1.000 | 1.000 | 1.000 |  | 1.000 | **0.002** | 1.000 | 1.000 | 1.000 | 0.385 |
| OTU_6222 | 1.000 | 1.000 | **0.003** | 1.000 | 0.109 | **0.036** |  | 1.000 | 1.000 | 0.182 | 1.000 | 0.079 | **0.004** |  | 1.000 | 1.000 | **< 0.001** | 1.000 | **< 0.001** | **< 0.001** |
| OTU_623 | 1.000 | 1.000 | **0.007** | **0.022** | **< 0.001** | 1.000 |  | 1.000 | **0.002** | **< 0.001** | 1.000 | **0.011** | 0.950 |  | 1.000 | **0.008** | **0.001** | 0.150 | **0.020** | 1.000 |
| OTU_6235 | 0.622 | **0.029** | **0.002** | 1.000 | 1.000 | 1.000 |  | 0.622 | **0.012** | 0.265 | 1.000 | 1.000 | 1.000 |  | **< 0.001** | **< 0.001** | **0.001** | 0.637 | 1.000 | 0.239 |
| OTU_6239 | 1.000 | 1.000 | 0.105 | 1.000 | 0.582 | 0.105 |  | 1.000 | 1.000 | 0.239 | 1.000 | **0.027** | 1.000 |  | 1.000 | 1.000 | 0.187 | 1.000 | 1.000 | 1.000 |
| OTU_624 | 1.000 | 1.000 | 1.000 | 1.000 | 1.000 | 1.000 |  | 1.000 | 1.000 | 1.000 | 1.000 | 1.000 | 1.000 |  | 1.000 | 1.000 | 1.000 | 1.000 | 1.000 | 1.000 |
| OTU_6240 | 1.000 | 1.000 | 1.000 | 1.000 | **0.034** | 1.000 |  | 1.000 | 1.000 | 1.000 | 0.123 | 0.313 | 1.000 |  | 1.000 | 1.000 | 0.496 | 0.506 | **0.005** | 1.000 |
| OTU_6244 | 1.000 | 0.708 | 1.000 | 1.000 | 1.000 | 0.810 |  | 1.000 | 1.000 | 1.000 | 1.000 | 1.000 | 1.000 |  | 0.960 | 1.000 | 0.384 | 1.000 | **< 0.001** | **0.002** |
| OTU_625 | 1.000 | **< 0.001** | **< 0.001** | **< 0.001** | **< 0.001** | 0.457 |  | 1.000 | **< 0.001** | **< 0.001** | **< 0.001** | **< 0.001** | 1.000 |  | 1.000 | **< 0.001** | **< 0.001** | **< 0.001** | **< 0.001** | 1.000 |
| OTU_6250 | 1.000 | **< 0.001** | **< 0.001** | 0.101 | **0.022** | 1.000 |  | 1.000 | 1.000 | **< 0.001** | 0.411 | **< 0.001** | 0.554 |  | 1.000 | **< 0.001** | **< 0.001** | **< 0.001** | **< 0.001** | 1.000 |
| OTU_626 | **0.003** | 0.214 | 1.000 | 1.000 | **0.010** | 1.000 |  | **0.003** | **0.013** | 1.000 | 1.000 | 0.484 | 0.149 |  | 0.169 | 1.000 | 1.000 | 1.000 | **0.002** | 0.613 |
| OTU_6263 | 1.000 | 0.207 | 1.000 | 1.000 | 1.000 | 1.000 |  | 1.000 | 1.000 | 1.000 | 1.000 | 1.000 | 1.000 |  | 1.000 | 1.000 | 1.000 | 1.000 | 1.000 | 1.000 |
| OTU_6269 | 1.000 | 1.000 | 1.000 | 1.000 | 1.000 | 1.000 |  | 1.000 | **0.018** | **0.009** | 0.495 | 0.224 | 1.000 |  | 1.000 | 0.191 | **0.025** | 1.000 | 0.442 | 1.000 |
| OTU_627 | 1.000 | 1.000 | 1.000 | 1.000 | 1.000 | 1.000 |  | 1.000 | 1.000 | 1.000 | 1.000 | 1.000 | 1.000 |  | 1.000 | 1.000 | 1.000 | 1.000 | 1.000 | 1.000 |
| OTU_628 | 1.000 | 0.058 | 0.957 | 0.632 | 1.000 | 1.000 |  | 1.000 | 0.910 | 1.000 | **< 0.001** | **< 0.001** | 1.000 |  | 1.000 | 0.192 | 1.000 | **0.025** | 0.332 | 1.000 |
| OTU_6285 | 1.000 | 1.000 | 1.000 | 1.000 | 1.000 | 1.000 |  | 1.000 | 1.000 | 1.000 | 1.000 | 0.662 | 1.000 |  | 1.000 | 0.663 | **0.005** | 1.000 | 0.227 | 1.000 |
| OTU_629 | 1.000 | 0.282 | 1.000 | 1.000 | 1.000 | 1.000 |  | 1.000 | **< 0.001** | **< 0.001** | 1.000 | 1.000 | 1.000 |  | 1.000 | **0.015** | 0.243 | **0.034** | 0.400 | 1.000 |
| OTU_63 | 1.000 | **< 0.001** | **< 0.001** | **< 0.001** | **< 0.001** | **0.007** |  | 1.000 | 0.289 | **< 0.001** | **< 0.001** | **< 0.001** | **< 0.001** |  | 0.780 | **< 0.001** | **< 0.001** | **< 0.001** | **< 0.001** | 1.000 |
| OTU_630 | 1.000 | 1.000 | **< 0.001** | 1.000 | **0.045** | 0.110 |  | 1.000 | 1.000 | **< 0.001** | 1.000 | 0.052 | **0.045** |  | 1.000 | 1.000 | **< 0.001** | 1.000 | **0.004** | 0.058 |
| OTU_6306 | 1.000 | 0.153 | 0.162 | 1.000 | 1.000 | 1.000 |  | 1.000 | 0.107 | 0.544 | 1.000 | 1.000 | 1.000 |  | 0.976 | **0.023** | **0.019** | 1.000 | 1.000 | 1.000 |
| OTU_631 | 1.000 | 0.107 | 0.198 | 0.198 | 0.270 | 1.000 |  | 1.000 | **< 0.001** | **< 0.001** | 0.070 | 0.167 | 1.000 |  | 1.000 | 0.087 | 0.506 | **0.003** | **0.014** | 1.000 |
| OTU_632 | 1.000 | 1.000 | 1.000 | 1.000 | 1.000 | 1.000 |  | 1.000 | 1.000 | 1.000 | 1.000 | 1.000 | 1.000 |  | 1.000 | 1.000 | 1.000 | 1.000 | 1.000 | 1.000 |
| OTU_6324 | **0.018** | **< 0.001** | 0.157 | **< 0.001** | 1.000 | **< 0.001** |  | **0.018** | **< 0.001** | 1.000 | **< 0.001** | 1.000 | **< 0.001** |  | 1.000 | **< 0.001** | 1.000 | **< 0.001** | 1.000 | **< 0.001** |
| OTU_633 | 0.054 | **< 0.001** | **< 0.001** | 1.000 | **0.032** | 1.000 |  | 0.054 | **< 0.001** | **< 0.001** | 1.000 | 1.000 | 1.000 |  | 1.000 | 0.515 | 1.000 | 1.000 | 1.000 | 1.000 |
| OTU_634 | 1.000 | 1.000 | 1.000 | 1.000 | 1.000 | 1.000 |  | 1.000 | 1.000 | 1.000 | 1.000 | 1.000 | 1.000 |  | 1.000 | 1.000 | 1.000 | 1.000 | 1.000 | 1.000 |
| OTU_635 | 1.000 | 1.000 | 1.000 | 1.000 | 1.000 | 1.000 |  | 1.000 | 1.000 | 1.000 | 1.000 | 1.000 | 1.000 |  | 1.000 | 1.000 | 1.000 | 1.000 | 0.602 | 1.000 |
| OTU_6355 | 1.000 | 1.000 | 1.000 | 1.000 | 1.000 | 1.000 |  | 1.000 | 1.000 | 1.000 | 1.000 | 1.000 | 1.000 |  | 1.000 | 1.000 | 1.000 | 1.000 | 1.000 | 1.000 |
| OTU_6359 | 0.905 | 0.659 | 1.000 | 1.000 | 1.000 | 1.000 |  | 0.905 | 0.081 | 0.102 | 1.000 | 1.000 | 1.000 |  | 1.000 | 1.000 | 1.000 | 1.000 | 1.000 | 1.000 |
| OTU_636 | 1.000 | 0.871 | **< 0.001** | 0.796 | **< 0.001** | 0.105 |  | 1.000 | 1.000 | **0.047** | 1.000 | 0.056 | 0.729 |  | 1.000 | 1.000 | 0.593 | 1.000 | 0.581 | 0.145 |
| OTU_6369 | 1.000 | 1.000 | 0.917 | 1.000 | 1.000 | 1.000 |  | 1.000 | 1.000 | 1.000 | 1.000 | 1.000 | 1.000 |  | 1.000 | 0.198 | 1.000 | 1.000 | 1.000 | 1.000 |
| OTU_637 | 0.254 | 1.000 | 1.000 | 0.236 | **0.033** | 1.000 |  | 0.254 | 1.000 | 1.000 | 1.000 | 1.000 | 1.000 |  | 1.000 | 1.000 | 1.000 | 1.000 | 1.000 | 1.000 |
| OTU_6379 | 1.000 | 0.052 | 1.000 | 1.000 | 1.000 | 1.000 |  | 1.000 | **0.016** | **0.003** | 1.000 | 1.000 | 1.000 |  | **0.013** | **0.022** | **0.007** | 1.000 | 1.000 | 1.000 |
| OTU_638 | 1.000 | 1.000 | **0.009** | 1.000 | **0.044** | **0.013** |  | 1.000 | 1.000 | **< 0.001** | 1.000 | **0.004** | **0.003** |  | 1.000 | 1.000 | **0.027** | 1.000 | 0.221 | **0.030** |
| OTU_6387 | 1.000 | **0.008** | 1.000 | 1.000 | 1.000 | 1.000 |  | 1.000 | **< 0.001** | 0.118 | **0.002** | 1.000 | 0.830 |  | 0.132 | **< 0.001** | **0.017** | 1.000 | 1.000 | 1.000 |
| OTU_639 | 0.166 | **0.019** | 0.152 | 1.000 | 1.000 | 1.000 |  | 0.166 | **0.019** | 1.000 | 0.065 | 1.000 | 1.000 |  | 1.000 | 1.000 | 0.708 | 1.000 | 1.000 | 1.000 |
| OTU_6398 | 1.000 | 1.000 | 1.000 | 1.000 | 0.773 | 1.000 |  | 1.000 | 1.000 | 0.877 | 1.000 | 1.000 | 1.000 |  | 1.000 | 1.000 | 1.000 | 1.000 | 1.000 | 0.334 |
| OTU_64 | 0.105 | 1.000 | **0.007** | 1.000 | **< 0.001** | **< 0.001** |  | 0.105 | 1.000 | 1.000 | 1.000 | 1.000 | 0.357 |  | 1.000 | 1.000 | **< 0.001** | 1.000 | 0.069 | **< 0.001** |
| OTU_640 | 1.000 | 1.000 | 1.000 | 1.000 | 1.000 | 1.000 |  | 1.000 | 1.000 | 1.000 | 1.000 | 1.000 | 1.000 |  | 1.000 | 1.000 | 1.000 | 1.000 | 1.000 | 1.000 |
| OTU_6405 | 1.000 | 1.000 | 0.310 | 1.000 | 0.054 | 0.594 |  | 1.000 | 1.000 | 1.000 | 1.000 | 1.000 | 1.000 |  | 1.000 | 1.000 | 0.364 | 1.000 | 0.404 | 1.000 |
| OTU_641 | **0.003** | **0.004** | 1.000 | 1.000 | **0.007** | **0.040** |  | **0.003** | 1.000 | **0.007** | 1.000 | **0.009** | **0.008** |  | 1.000 | 1.000 | 1.000 | 1.000 | 0.292 | 1.000 |
| OTU_6412 | 1.000 | 1.000 | 1.000 | 1.000 | 1.000 | 1.000 |  | 1.000 | 1.000 | 1.000 | 1.000 | 1.000 | 1.000 |  | 1.000 | **0.014** | **< 0.001** | 0.151 | **< 0.001** | 1.000 |
| OTU_6417 | 1.000 | **0.015** | 0.407 | 0.702 | 1.000 | 1.000 |  | 1.000 | 1.000 | 0.097 | 1.000 | 0.162 | 1.000 |  | 1.000 | **0.050** | 0.115 | 1.000 | 1.000 | 1.000 |
| OTU_6418 | 1.000 | 1.000 | 1.000 | 1.000 | 1.000 | 0.687 |  | 1.000 | 1.000 | 0.944 | 1.000 | 1.000 | 1.000 |  | 1.000 | 1.000 | 1.000 | 1.000 | 1.000 | 1.000 |
| OTU_642 | 1.000 | 1.000 | 1.000 | 1.000 | 1.000 | 1.000 |  | 1.000 | **< 0.001** | **0.006** | 1.000 | 1.000 | 1.000 |  | 1.000 | 0.214 | 1.000 | 1.000 | 1.000 | 1.000 |
| OTU_6420 | 0.091 | 0.146 | 0.528 | 1.000 | **< 0.001** | **< 0.001** |  | 0.091 | **0.030** | 1.000 | 1.000 | **< 0.001** | **< 0.001** |  | 1.000 | 1.000 | **< 0.001** | 1.000 | **< 0.001** | **0.011** |
| OTU_6427 | 1.000 | 1.000 | 0.624 | 1.000 | 1.000 | 1.000 |  | 1.000 | 1.000 | 1.000 | 0.098 | 1.000 | 1.000 |  | 1.000 | 1.000 | 1.000 | 1.000 | 1.000 | 1.000 |
| OTU_643 | **0.042** | **< 0.001** | **< 0.001** | **< 0.001** | **0.046** | 1.000 |  | **0.042** | **< 0.001** | **< 0.001** | 0.275 | 0.374 | 1.000 |  | 0.152 | **< 0.001** | **< 0.001** | **0.012** | 0.170 | 1.000 |
| OTU_6434 | 1.000 | 1.000 | 1.000 | 1.000 | 1.000 | 1.000 |  | 1.000 | 1.000 | 1.000 | 1.000 | 1.000 | 1.000 |  | 1.000 | 0.242 | 0.161 | 1.000 | 1.000 | 1.000 |
| OTU_6437 | 1.000 | 1.000 | 1.000 | 1.000 | 1.000 | 1.000 |  | 1.000 | 1.000 | 0.247 | 1.000 | 1.000 | 1.000 |  | 1.000 | 0.138 | 0.089 | 1.000 | 1.000 | 1.000 |
| OTU_6438 | 1.000 | **0.028** | 1.000 | **0.007** | 0.473 | 1.000 |  | 1.000 | 0.937 | 1.000 | **0.040** | 1.000 | 1.000 |  | 1.000 | 1.000 | 1.000 | 1.000 | 1.000 | 1.000 |
| OTU_644 | 0.331 | **< 0.001** | **< 0.001** | 1.000 | 1.000 | 1.000 |  | 0.331 | **< 0.001** | **0.006** | 0.825 | 1.000 | 1.000 |  | 0.365 | 0.181 | **0.042** | 1.000 | 1.000 | 1.000 |
| OTU_6447 | **0.021** | 0.114 | **0.003** | 1.000 | 1.000 | 1.000 |  | **0.021** | 1.000 | 1.000 | 1.000 | 1.000 | 1.000 |  | 1.000 | 1.000 | 1.000 | 1.000 | 1.000 | 1.000 |
| OTU_645 | 1.000 | 1.000 | 1.000 | 1.000 | 1.000 | 1.000 |  | 1.000 | 0.224 | **0.031** | 1.000 | 1.000 | 1.000 |  | 1.000 | 1.000 | 1.000 | 1.000 | 1.000 | 1.000 |
| OTU_646 | 0.646 | **0.042** | 0.369 | **< 0.001** | **< 0.001** | 1.000 |  | 0.646 | **< 0.001** | **< 0.001** | **0.029** | **0.009** | 1.000 |  | 1.000 | 0.066 | 0.088 | 0.690 | 0.675 | 1.000 |
| OTU_647 | 0.815 | 1.000 | **0.003** | **0.002** | **< 0.001** | 1.000 |  | 0.815 | 0.497 | **0.001** | 0.662 | **0.001** | 1.000 |  | 1.000 | **0.006** | **< 0.001** | 1.000 | **0.012** | 1.000 |
| OTU_648 | 1.000 | 1.000 | 1.000 | 0.881 | 0.744 | 1.000 |  | 1.000 | 1.000 | 0.350 | 1.000 | 1.000 | 1.000 |  | 1.000 | 1.000 | 1.000 | 1.000 | 1.000 | 1.000 |
| OTU_649 | 1.000 | **< 0.001** | **0.014** | **< 0.001** | **0.004** | 0.087 |  | 1.000 | **< 0.001** | **0.002** | **< 0.001** | 0.529 | **0.014** |  | 1.000 | **< 0.001** | 0.344 | **< 0.001** | 1.000 | **0.011** |
| OTU_65 | 0.220 | **< 0.001** | **0.009** | 0.417 | 1.000 | 1.000 |  | 0.220 | **< 0.001** | **< 0.001** | 0.657 | 1.000 | 1.000 |  | **0.010** | **< 0.001** | **< 0.001** | 0.305 | 0.770 | 1.000 |
| OTU_650 | 1.000 | 1.000 | 1.000 | 1.000 | 1.000 | 1.000 |  | 1.000 | 1.000 | 1.000 | 1.000 | 1.000 | 1.000 |  | 1.000 | 1.000 | 1.000 | 1.000 | 1.000 | 1.000 |
| OTU_6501 | **0.049** | **0.034** | **0.028** | 1.000 | 1.000 | 1.000 |  | **0.049** | 1.000 | 1.000 | 1.000 | 1.000 | 1.000 |  | **0.019** | **< 0.001** | **< 0.001** | **< 0.001** | **< 0.001** | 1.000 |
| OTU_651 | 1.000 | **< 0.001** | **0.002** | 0.074 | 0.103 | 1.000 |  | 1.000 | **< 0.001** | **< 0.001** | 0.075 | **< 0.001** | 1.000 |  | 0.125 | **< 0.001** | **< 0.001** | 0.103 | **0.016** | 1.000 |
| OTU_652 | 1.000 | 1.000 | 1.000 | 1.000 | 1.000 | 1.000 |  | 1.000 | **0.001** | 0.077 | 1.000 | 1.000 | 1.000 |  | 1.000 | **0.009** | **0.012** | 0.968 | 0.931 | 1.000 |
| OTU_6527 | 1.000 | 1.000 | 1.000 | 1.000 | 1.000 | 1.000 |  | 1.000 | 0.538 | 1.000 | 1.000 | 1.000 | 1.000 |  | 1.000 | 0.666 | 1.000 | 1.000 | 1.000 | 1.000 |
| OTU_653 | 1.000 | 1.000 | 1.000 | 1.000 | 1.000 | 1.000 |  | 1.000 | 1.000 | 0.433 | 1.000 | 1.000 | 1.000 |  | 1.000 | 1.000 | 1.000 | 1.000 | 1.000 | 1.000 |
| OTU_6532 | 0.151 | **< 0.001** | **0.030** | 1.000 | 1.000 | 1.000 |  | 0.151 | 1.000 | 0.366 | 1.000 | 1.000 | 1.000 |  | 1.000 | **0.005** | 0.518 | 0.458 | 1.000 | 1.000 |
| OTU_654 | 1.000 | 1.000 | 1.000 | 1.000 | 1.000 | 1.000 |  | 1.000 | 1.000 | 1.000 | 1.000 | 1.000 | 1.000 |  | 1.000 | 1.000 | 1.000 | 1.000 | 1.000 | 1.000 |
| OTU_6544 | 1.000 | 1.000 | 1.000 | 1.000 | 1.000 | 1.000 |  | 1.000 | 1.000 | 1.000 | 1.000 | 0.160 | 1.000 |  | 0.575 | 0.070 | 0.114 | 1.000 | 1.000 | 1.000 |
| OTU_6547 | 0.396 | **< 0.001** | **0.002** | 0.247 | 1.000 | 1.000 |  | 0.396 | **< 0.001** | **< 0.001** | **0.005** | 0.121 | 1.000 |  | 0.524 | **< 0.001** | **< 0.001** | 0.461 | 1.000 | 1.000 |
| OTU_655 | 0.743 | 0.305 | 0.541 | **< 0.001** | **< 0.001** | 1.000 |  | 0.743 | 1.000 | 1.000 | **0.013** | **0.003** | 1.000 |  | 1.000 | 0.488 | 0.114 | 1.000 | 1.000 | 1.000 |
| OTU_656 | 1.000 | 1.000 | 0.340 | 1.000 | 1.000 | 1.000 |  | 1.000 | 1.000 | 0.285 | 1.000 | 1.000 | 1.000 |  | 1.000 | 1.000 | 1.000 | 1.000 | 1.000 | 1.000 |
| OTU_6560 | 1.000 | 1.000 | 1.000 | 1.000 | 1.000 | 1.000 |  | 1.000 | 1.000 | 1.000 | 0.662 | 0.850 | 1.000 |  | 1.000 | 1.000 | 1.000 | 1.000 | 1.000 | 1.000 |
| OTU_657 | 0.723 | **< 0.001** | **< 0.001** | **0.014** | 0.307 | 1.000 |  | 0.723 | **0.015** | 1.000 | **0.024** | 1.000 | 1.000 |  | 1.000 | 1.000 | 1.000 | 1.000 | 1.000 | 1.000 |
| OTU_658 | 1.000 | 1.000 | **< 0.001** | 1.000 | **< 0.001** | **< 0.001** |  | 1.000 | **< 0.001** | **< 0.001** | 0.103 | **< 0.001** | 0.231 |  | 1.000 | 1.000 | **< 0.001** | 1.000 | **< 0.001** | **0.022** |
| OTU_659 | 1.000 | 1.000 | 1.000 | 1.000 | 1.000 | 1.000 |  | 1.000 | 1.000 | 1.000 | 1.000 | 1.000 | 1.000 |  | 1.000 | 1.000 | 1.000 | 1.000 | 1.000 | 1.000 |
| OTU_66 | 1.000 | 1.000 | 1.000 | 1.000 | 1.000 | 1.000 |  | 1.000 | **< 0.001** | **< 0.001** | 1.000 | 1.000 | 1.000 |  | 1.000 | 0.093 | 1.000 | 1.000 | 1.000 | 1.000 |
| OTU_660 | 0.151 | **< 0.001** | 0.694 | 1.000 | 1.000 | 0.552 |  | 0.151 | **< 0.001** | **< 0.001** | **< 0.001** | 0.088 | 0.125 |  | 1.000 | **0.002** | 0.317 | 0.623 | 1.000 | 1.000 |
| OTU_6603 | 1.000 | 1.000 | 1.000 | 1.000 | 1.000 | 1.000 |  | 1.000 | 0.198 | **0.018** | 1.000 | 1.000 | 1.000 |  | 1.000 | 1.000 | 1.000 | 1.000 | 1.000 | 1.000 |
| OTU_6604 | 1.000 | **0.004** | **0.001** | **0.008** | **0.001** | 1.000 |  | 1.000 | **0.026** | **< 0.001** | 1.000 | 1.000 | 1.000 |  | 1.000 | 1.000 | 1.000 | 1.000 | 1.000 | 1.000 |
| OTU_661 | 1.000 | 1.000 | 1.000 | 1.000 | 1.000 | 1.000 |  | 1.000 | **< 0.001** | **< 0.001** | 1.000 | 1.000 | 1.000 |  | 1.000 | 0.188 | 1.000 | 0.329 | 1.000 | **0.009** |
| OTU_6618 | 1.000 | **0.017** | 1.000 | 1.000 | 1.000 | 1.000 |  | 1.000 | **0.018** | 1.000 | 1.000 | 1.000 | 1.000 |  | 1.000 | 1.000 | 1.000 | 1.000 | 0.475 | 1.000 |
| OTU_662 | 0.177 | **< 0.001** | **< 0.001** | 0.353 | **< 0.001** | 0.977 |  | 0.177 | **< 0.001** | **< 0.001** | 0.497 | **0.046** | 1.000 |  | 1.000 | **< 0.001** | **< 0.001** | 0.162 | **< 0.001** | 0.358 |
| OTU_6623 | 0.505 | 0.333 | 1.000 | 1.000 | 1.000 | 1.000 |  | 0.505 | 1.000 | 0.285 | 1.000 | 1.000 | 1.000 |  | 1.000 | 0.191 | 0.140 | 1.000 | 1.000 | 1.000 |
| OTU_663 | 1.000 | 0.870 | 1.000 | 1.000 | 1.000 | 1.000 |  | 1.000 | **< 0.001** | **< 0.001** | 0.269 | 0.092 | 1.000 |  | 0.619 | **0.048** | **0.004** | 1.000 | 1.000 | 1.000 |
| OTU_6634 | 1.000 | 1.000 | **0.021** | 1.000 | 0.927 | 1.000 |  | 1.000 | 0.080 | 0.095 | 1.000 | 1.000 | 1.000 |  | 1.000 | 0.822 | **0.024** | 1.000 | 0.678 | 1.000 |
| OTU_664 | **< 0.001** | **< 0.001** | **< 0.001** | **< 0.001** | **< 0.001** | 1.000 |  | **< 0.001** | **< 0.001** | **< 0.001** | **< 0.001** | **< 0.001** | 0.584 |  | **< 0.001** | **< 0.001** | **< 0.001** | **< 0.001** | **< 0.001** | 1.000 |
| OTU_665 | 1.000 | 1.000 | **< 0.001** | 1.000 | **< 0.001** | **< 0.001** |  | 1.000 | 0.207 | 1.000 | 1.000 | **0.023** | **0.002** |  | 1.000 | 1.000 | **0.002** | 1.000 | **0.004** | **0.001** |
| OTU_6656 | 1.000 | 1.000 | 1.000 | 1.000 | 0.243 | 1.000 |  | 1.000 | 0.473 | **0.019** | **0.007** | **< 0.001** | 1.000 |  | 1.000 | 1.000 | 1.000 | 1.000 | 1.000 | 1.000 |
| OTU_666 | 1.000 | 1.000 | 1.000 | 1.000 | 1.000 | 1.000 |  | 1.000 | 1.000 | 0.449 | 1.000 | 1.000 | 1.000 |  | 1.000 | 1.000 | 1.000 | 1.000 | 1.000 | 1.000 |
| OTU_667 | 1.000 | 1.000 | **0.009** | 0.138 | **< 0.001** | 0.342 |  | 1.000 | 1.000 | 0.353 | 0.060 | **< 0.001** | 1.000 |  | 1.000 | 1.000 | 0.178 | 1.000 | 1.000 | 1.000 |
| OTU_6674 | 1.000 | 1.000 | **0.004** | 1.000 | **0.005** | 0.832 |  | 1.000 | 1.000 | 1.000 | 1.000 | 1.000 | 1.000 |  | 1.000 | 1.000 | **0.006** | 1.000 | 0.906 | **0.033** |
| OTU_668 | 1.000 | 1.000 | 1.000 | 1.000 | 1.000 | 1.000 |  | 1.000 | 1.000 | 1.000 | 1.000 | 1.000 | 1.000 |  | 1.000 | 1.000 | 1.000 | 1.000 | 1.000 | 1.000 |
| OTU_6680 | 1.000 | 1.000 | 1.000 | 1.000 | 1.000 | 1.000 |  | 1.000 | 1.000 | 1.000 | 1.000 | 1.000 | 1.000 |  | 1.000 | 1.000 | 0.116 | 1.000 | 0.074 | 0.315 |
| OTU_6688 | 0.344 | 0.081 | **0.006** | 1.000 | 1.000 | 1.000 |  | 0.344 | 1.000 | 1.000 | 1.000 | 1.000 | 1.000 |  | 1.000 | **0.024** | **0.029** | 1.000 | 1.000 | 1.000 |
| OTU_669 | 1.000 | **< 0.001** | **0.046** | **< 0.001** | 1.000 | 0.222 |  | 1.000 | **< 0.001** | 0.994 | 1.000 | 1.000 | **0.027** |  | 1.000 | **< 0.001** | **0.008** | **0.022** | 1.000 | 1.000 |
| OTU_67 | **0.004** | **< 0.001** | 0.385 | 1.000 | 1.000 | **0.015** |  | **0.004** | **< 0.001** | 1.000 | 1.000 | **< 0.001** | **< 0.001** |  | **< 0.001** | **< 0.001** | **< 0.001** | **0.024** | 1.000 | 0.052 |
| OTU_670 | 1.000 | **0.006** | 0.792 | 0.228 | 1.000 | 1.000 |  | 1.000 | 0.362 | 1.000 | 0.822 | 1.000 | 1.000 |  | 1.000 | 0.064 | 0.084 | 1.000 | 1.000 | 1.000 |
| OTU_671 | 1.000 | 0.786 | 1.000 | 1.000 | 1.000 | 0.100 |  | 1.000 | 1.000 | 0.219 | 1.000 | 0.093 | 1.000 |  | 1.000 | 1.000 | 1.000 | 1.000 | 1.000 | 0.066 |
| OTU_6719 | 1.000 | 1.000 | 0.113 | 1.000 | 0.086 | 1.000 |  | 1.000 | **0.033** | 0.056 | 0.460 | 0.632 | 1.000 |  | 0.230 | **0.001** | **< 0.001** | 1.000 | 1.000 | 1.000 |
| OTU_672 | **0.035** | **< 0.001** | **< 0.001** | **0.006** | **0.010** | 1.000 |  | **0.035** | **< 0.001** | **< 0.001** | **0.002** | 1.000 | 0.291 |  | **0.010** | **< 0.001** | **< 0.001** | **< 0.001** | **0.009** | 1.000 |
| OTU_6724 | 1.000 | 1.000 | 1.000 | 1.000 | 1.000 | 1.000 |  | 1.000 | 1.000 | 1.000 | 1.000 | 1.000 | 1.000 |  | 1.000 | 1.000 | 1.000 | 1.000 | 1.000 | 1.000 |
| OTU_673 | 0.992 | 1.000 | 1.000 | **0.028** | **0.034** | 1.000 |  | 0.992 | **0.021** | **< 0.001** | **< 0.001** | **< 0.001** | 1.000 |  | 1.000 | 0.092 | **< 0.001** | 1.000 | **0.013** | 0.358 |
| OTU_6739 | 0.080 | **< 0.001** | **< 0.001** | 1.000 | 1.000 | 1.000 |  | 0.080 | **0.032** | 0.395 | 1.000 | 1.000 | 1.000 |  | 1.000 | 0.943 | 1.000 | 1.000 | 1.000 | 1.000 |
| OTU_674 | 1.000 | 1.000 | 0.244 | 1.000 | **< 0.001** | **< 0.001** |  | 1.000 | 0.111 | **< 0.001** | 0.162 | **< 0.001** | 1.000 |  | 1.000 | 1.000 | 0.302 | 1.000 | 1.000 | 1.000 |
| OTU_6743 | 1.000 | 1.000 | 1.000 | 1.000 | 1.000 | 1.000 |  | 1.000 | **< 0.001** | **0.002** | 1.000 | 1.000 | 1.000 |  | 1.000 | 0.145 | 0.443 | 1.000 | 1.000 | 1.000 |
| OTU_675 | **< 0.001** | **< 0.001** | **< 0.001** | 1.000 | 1.000 | 1.000 |  | **< 0.001** | **< 0.001** | **0.007** | 1.000 | 1.000 | 1.000 |  | **0.004** | 0.178 | **0.007** | 1.000 | 1.000 | 1.000 |
| OTU_6759 | 1.000 | 0.526 | 0.274 | 1.000 | 1.000 | 1.000 |  | 1.000 | 0.964 | 1.000 | 1.000 | 1.000 | 1.000 |  | 1.000 | 1.000 | 1.000 | 1.000 | 1.000 | 1.000 |
| OTU_676 | 1.000 | 1.000 | 0.849 | 1.000 | 0.074 | 1.000 |  | 1.000 | 1.000 | 0.237 | 1.000 | **0.023** | **0.008** |  | 1.000 | 1.000 | 1.000 | 1.000 | 1.000 | 1.000 |
| OTU_6763 | **0.016** | **< 0.001** | **< 0.001** | **< 0.001** | **< 0.001** | 1.000 |  | **0.016** | **< 0.001** | **< 0.001** | **0.028** | **0.031** | 1.000 |  | 0.411 | **< 0.001** | **< 0.001** | **0.011** | **0.009** | 1.000 |
| OTU_6765 | 0.376 | **< 0.001** | **< 0.001** | 0.292 | 0.192 | 1.000 |  | 0.376 | 0.060 | **0.003** | 1.000 | 1.000 | 1.000 |  | 0.856 | 0.486 | 0.338 | 1.000 | 1.000 | 1.000 |
| OTU_677 | 1.000 | 1.000 | **0.001** | 1.000 | **0.022** | **0.015** |  | 1.000 | **0.007** | **< 0.001** | **< 0.001** | **< 0.001** | 1.000 |  | 1.000 | 0.848 | 0.057 | 1.000 | 0.068 | 1.000 |
| OTU_6771 | 0.396 | **0.023** | 0.265 | 1.000 | 1.000 | 1.000 |  | 0.396 | 0.336 | **0.036** | 0.461 | **0.044** | 1.000 |  | 1.000 | 1.000 | 1.000 | **0.021** | **0.015** | 1.000 |
| OTU_6773 | 1.000 | **0.042** | **0.037** | 0.164 | 0.109 | 1.000 |  | 1.000 | **0.012** | 0.714 | 1.000 | 1.000 | 1.000 |  | 1.000 | 0.263 | 1.000 | 1.000 | 1.000 | 1.000 |
| OTU_678 | 1.000 | 1.000 | 1.000 | 1.000 | 0.464 | 1.000 |  | 1.000 | 1.000 | 1.000 | 0.291 | 1.000 | 1.000 |  | 1.000 | 1.000 | 1.000 | 1.000 | 1.000 | 1.000 |
| OTU_6786 | 1.000 | 1.000 | 1.000 | 1.000 | 1.000 | 1.000 |  | 1.000 | 1.000 | 0.565 | 1.000 | 1.000 | 1.000 |  | 1.000 | 1.000 | 1.000 | 1.000 | 1.000 | 1.000 |
| OTU_679 | **< 0.001** | 0.180 | 1.000 | 0.492 | **0.023** | 1.000 |  | **< 0.001** | 1.000 | 0.588 | 1.000 | 0.765 | 1.000 |  | 0.805 | 0.359 | 1.000 | 1.000 | 0.635 | 0.913 |
| OTU_6791 | 1.000 | 1.000 | 0.581 | 1.000 | **0.023** | 1.000 |  | 1.000 | 1.000 | 0.232 | 1.000 | 1.000 | 1.000 |  | 1.000 | 1.000 | 1.000 | 1.000 | 1.000 | 1.000 |
| OTU_6792 | 0.060 | **0.003** | **< 0.001** | 1.000 | 0.962 | 1.000 |  | 0.060 | **0.024** | 0.071 | **0.003** | **0.007** | 1.000 |  | 1.000 | 0.076 | **< 0.001** | **0.049** | **< 0.001** | 1.000 |
| OTU_6799 | 1.000 | 1.000 | 0.679 | 1.000 | 1.000 | 1.000 |  | 1.000 | 1.000 | 1.000 | 1.000 | 1.000 | 1.000 |  | 1.000 | 1.000 | 1.000 | 1.000 | 1.000 | 1.000 |
| OTU_68 | **< 0.001** | **< 0.001** | **< 0.001** | 1.000 | 1.000 | 1.000 |  | **< 0.001** | **< 0.001** | **0.019** | **< 0.001** | 1.000 | 0.093 |  | **0.002** | **< 0.001** | 0.073 | 1.000 | 1.000 | 0.084 |
| OTU_680 | 1.000 | 1.000 | 1.000 | 1.000 | 1.000 | 1.000 |  | 1.000 | **0.014** | **0.009** | 1.000 | 1.000 | 1.000 |  | 1.000 | 1.000 | 0.167 | 1.000 | 1.000 | 1.000 |
| OTU_6803 | 1.000 | 0.573 | **0.004** | 0.531 | **0.002** | 1.000 |  | 1.000 | 1.000 | **0.024** | 1.000 | **0.003** | **0.039** |  | 0.916 | 0.242 | **< 0.001** | 1.000 | 0.145 | 0.456 |
| OTU_6809 | 1.000 | 1.000 | 1.000 | 1.000 | 1.000 | 1.000 |  | 1.000 | 1.000 | 1.000 | 0.591 | 1.000 | 1.000 |  | 1.000 | 1.000 | 1.000 | 1.000 | 1.000 | 1.000 |
| OTU_681 | 1.000 | 1.000 | 0.480 | 1.000 | 1.000 | 1.000 |  | 1.000 | 1.000 | **0.025** | 1.000 | 0.150 | 0.341 |  | 1.000 | 1.000 | 1.000 | 1.000 | 1.000 | 1.000 |
| OTU_6812 | 0.074 | **0.042** | 0.182 | 1.000 | 1.000 | 1.000 |  | 0.074 | 0.179 | 1.000 | 1.000 | 1.000 | 1.000 |  | 0.198 | **< 0.001** | **0.008** | 1.000 | 1.000 | 1.000 |
| OTU_682 | 1.000 | **0.006** | 1.000 | **< 0.001** | 0.590 | 0.113 |  | 1.000 | **< 0.001** | 1.000 | 0.119 | 1.000 | 0.108 |  | 1.000 | 0.166 | 1.000 | 1.000 | 1.000 | 1.000 |
| OTU_6821 | 1.000 | 1.000 | 1.000 | 1.000 | 1.000 | 1.000 |  | 1.000 | 0.598 | 0.207 | 1.000 | 1.000 | 1.000 |  | 1.000 | 1.000 | 1.000 | 1.000 | 1.000 | 1.000 |
| OTU_6829 | 1.000 | **0.025** | 0.060 | 1.000 | 1.000 | 1.000 |  | 1.000 | 0.422 | 0.988 | 1.000 | 1.000 | 1.000 |  | 1.000 | 0.066 | 0.076 | 1.000 | 1.000 | 1.000 |
| OTU_683 | 1.000 | 1.000 | 1.000 | 1.000 | 0.227 | 0.340 |  | 1.000 | 1.000 | 0.667 | 1.000 | 0.527 | 1.000 |  | 1.000 | 1.000 | 1.000 | 1.000 | 1.000 | 1.000 |
| OTU_684 | 1.000 | 0.255 | **0.005** | 1.000 | **< 0.001** | **< 0.001** |  | 1.000 | 0.568 | **< 0.001** | 0.150 | **0.006** | **< 0.001** |  | 1.000 | 1.000 | **< 0.001** | 1.000 | **< 0.001** | **< 0.001** |
| OTU_6840 | 1.000 | 1.000 | 1.000 | 1.000 | 1.000 | 1.000 |  | 1.000 | 1.000 | 1.000 | 1.000 | 1.000 | 1.000 |  | 0.619 | 1.000 | 1.000 | 1.000 | 1.000 | 1.000 |
| OTU_6841 | **< 0.001** | **< 0.001** | **0.036** | **0.024** | **0.006** | **< 0.001** |  | **< 0.001** | **< 0.001** | 1.000 | **0.014** | **0.018** | **< 0.001** |  | **< 0.001** | **< 0.001** | 0.054 | **0.005** | 0.403 | **< 0.001** |
| OTU_6847 | 1.000 | 1.000 | 1.000 | 1.000 | 0.088 | 0.446 |  | 1.000 | 1.000 | 1.000 | 1.000 | 1.000 | 1.000 |  | 1.000 | 1.000 | 0.159 | 1.000 | **0.015** | 0.559 |
| OTU_685 | 1.000 | 1.000 | 1.000 | 1.000 | 1.000 | 1.000 |  | 1.000 | 0.447 | 0.911 | 0.601 | 1.000 | 1.000 |  | 1.000 | 1.000 | 1.000 | 1.000 | 1.000 | 1.000 |
| OTU_686 | 1.000 | 1.000 | 1.000 | 0.232 | 1.000 | 1.000 |  | 1.000 | 1.000 | 1.000 | 1.000 | 1.000 | 1.000 |  | 1.000 | 1.000 | 1.000 | 1.000 | 1.000 | 1.000 |
| OTU_6860 | 1.000 | 0.111 | 1.000 | 1.000 | 1.000 | 1.000 |  | 1.000 | 1.000 | 1.000 | 1.000 | 1.000 | 1.000 |  | 1.000 | 1.000 | 1.000 | 1.000 | 1.000 | 1.000 |
| OTU_6866 | 1.000 | **< 0.001** | 0.593 | **0.038** | 1.000 | 0.772 |  | 1.000 | **< 0.001** | 1.000 | **0.022** | 1.000 | 0.177 |  | 1.000 | **0.004** | 1.000 | **0.012** | 1.000 | 1.000 |
| OTU_687 | 1.000 | 1.000 | 0.183 | 1.000 | 0.058 | 1.000 |  | 1.000 | 1.000 | 1.000 | 1.000 | 0.739 | 1.000 |  | 1.000 | 1.000 | 0.089 | 1.000 | 0.403 | 1.000 |
| OTU_6876 | 1.000 | 1.000 | 1.000 | 1.000 | 0.752 | 1.000 |  | 1.000 | 1.000 | 1.000 | 1.000 | 1.000 | 1.000 |  | 1.000 | 1.000 | 1.000 | 1.000 | 1.000 | 1.000 |
| OTU_6877 | 1.000 | 1.000 | 1.000 | 1.000 | 1.000 | 1.000 |  | 1.000 | 1.000 | 1.000 | 1.000 | 1.000 | 1.000 |  | 1.000 | 1.000 | 1.000 | 1.000 | 1.000 | 1.000 |
| OTU_688 | **0.020** | 1.000 | 1.000 | 0.505 | 0.851 | 1.000 |  | **0.020** | 0.251 | 1.000 | 1.000 | 1.000 | 1.000 |  | 1.000 | 1.000 | 1.000 | 1.000 | 1.000 | 1.000 |
| OTU_6885 | 1.000 | 1.000 | 1.000 | 1.000 | 1.000 | 1.000 |  | 1.000 | 0.864 | 0.066 | 1.000 | 1.000 | 1.000 |  | 1.000 | 1.000 | 1.000 | 1.000 | 1.000 | 1.000 |
| OTU_689 | 1.000 | 1.000 | 0.065 | 1.000 | 0.063 | **0.009** |  | 1.000 | 1.000 | **0.012** | 1.000 | **0.015** | **< 0.001** |  | 1.000 | 1.000 | 1.000 | 1.000 | 0.342 | 0.081 |
| OTU_6890 | **0.010** | **< 0.001** | 0.065 | 1.000 | 1.000 | 1.000 |  | **0.010** | **0.004** | **0.011** | 1.000 | 1.000 | 1.000 |  | 0.324 | **0.006** | **0.036** | 1.000 | 1.000 | 1.000 |
| OTU_6895 | 1.000 | 1.000 | 1.000 | 1.000 | 1.000 | 1.000 |  | 1.000 | **< 0.001** | **< 0.001** | **0.002** | **< 0.001** | 1.000 |  | 1.000 | 1.000 | 1.000 | 1.000 | 1.000 | 1.000 |
| OTU_69 | 1.000 | 1.000 | **0.004** | 1.000 | **0.006** | **0.033** |  | 1.000 | **< 0.001** | **< 0.001** | 1.000 | **0.012** | 0.207 |  | 1.000 | 0.675 | **0.005** | 1.000 | 0.230 | 1.000 |
| OTU_690 | **0.015** | **0.009** | 0.065 | 1.000 | 1.000 | 1.000 |  | **0.015** | **< 0.001** | **0.002** | 1.000 | 0.475 | 1.000 |  | **0.005** | **0.002** | **0.030** | 1.000 | 1.000 | 1.000 |
| OTU_6908 | 1.000 | 1.000 | **0.044** | 0.592 | **< 0.001** | 1.000 |  | 1.000 | **0.034** | **< 0.001** | **0.020** | **< 0.001** | 0.051 |  | 1.000 | 0.198 | **0.012** | **0.030** | **< 0.001** | 1.000 |
| OTU_691 | 0.181 | 1.000 | 1.000 | 1.000 | **0.010** | 1.000 |  | 0.181 | 1.000 | 0.653 | 0.093 | **< 0.001** | 1.000 |  | 1.000 | 1.000 | 1.000 | 0.835 | **0.003** | 1.000 |
| OTU_6916 | 1.000 | **0.026** | 1.000 | 1.000 | 1.000 | 1.000 |  | 1.000 | 0.457 | 1.000 | 0.279 | 1.000 | 1.000 |  | 1.000 | 0.289 | 1.000 | 0.888 | 1.000 | 1.000 |
| OTU_692 | 1.000 | 1.000 | 1.000 | 1.000 | 0.937 | 1.000 |  | 1.000 | 1.000 | 1.000 | 1.000 | 1.000 | 1.000 |  | 1.000 | 1.000 | 1.000 | 1.000 | 1.000 | 1.000 |
| OTU_693 | 1.000 | 1.000 | 1.000 | 1.000 | 1.000 | 1.000 |  | 1.000 | **0.044** | 0.285 | 1.000 | 1.000 | 1.000 |  | 1.000 | 0.546 | 0.575 | 1.000 | 1.000 | 1.000 |
| OTU_694 | 1.000 | 1.000 | 1.000 | 1.000 | 0.578 | 0.418 |  | 1.000 | 1.000 | 1.000 | 1.000 | 0.190 | 1.000 |  | 1.000 | 1.000 | 1.000 | 1.000 | 1.000 | 1.000 |
| OTU_695 | 1.000 | 1.000 | 1.000 | 1.000 | 1.000 | 1.000 |  | 1.000 | 1.000 | 1.000 | 1.000 | 1.000 | 1.000 |  | 1.000 | 1.000 | 1.000 | 1.000 | 1.000 | 1.000 |
| OTU_6951 | 1.000 | **0.029** | 1.000 | 1.000 | 1.000 | 0.868 |  | 1.000 | 0.487 | 1.000 | 1.000 | 1.000 | 1.000 |  | 1.000 | 1.000 | 1.000 | 0.731 | 1.000 | 0.706 |
| OTU_696 | 1.000 | 0.071 | 1.000 | 0.829 | 0.731 | **0.003** |  | 1.000 | 1.000 | 0.068 | 1.000 | **0.001** | 0.149 |  | 1.000 | 1.000 | 1.000 | 1.000 | **0.049** | 0.280 |
| OTU_6966 | 1.000 | 1.000 | 1.000 | 1.000 | 1.000 | 1.000 |  | 1.000 | 1.000 | 0.225 | 1.000 | 1.000 | 1.000 |  | 1.000 | 0.372 | **0.045** | 1.000 | 1.000 | 1.000 |
| OTU_6968 | 1.000 | 1.000 | 1.000 | 1.000 | 1.000 | 1.000 |  | 1.000 | **0.001** | **0.017** | 1.000 | 1.000 | 1.000 |  | 1.000 | 1.000 | 1.000 | 1.000 | 1.000 | 1.000 |
| OTU_697 | 1.000 | **0.033** | 1.000 | 1.000 | 1.000 | 1.000 |  | 1.000 | 1.000 | 0.477 | **0.045** | 1.000 | **0.020** |  | 1.000 | **0.007** | 1.000 | 0.690 | 1.000 | 0.137 |
| OTU_6972 | 1.000 | 1.000 | 1.000 | 1.000 | 1.000 | 1.000 |  | 1.000 | 1.000 | 1.000 | 1.000 | 0.160 | 1.000 |  | **< 0.001** | **< 0.001** | **< 0.001** | 0.500 | 0.621 | 1.000 |
| OTU_698 | 1.000 | **0.001** | **0.001** | **< 0.001** | **< 0.001** | 1.000 |  | 1.000 | **< 0.001** | **< 0.001** | **< 0.001** | **< 0.001** | 1.000 |  | 1.000 | **< 0.001** | **0.002** | **0.021** | 0.117 | 1.000 |
| OTU_6981 | 1.000 | 1.000 | 1.000 | 1.000 | 0.470 | 1.000 |  | 1.000 | 1.000 | 1.000 | 1.000 | 1.000 | 1.000 |  | 0.981 | 0.549 | 0.131 | 1.000 | 1.000 | 1.000 |
| OTU_6985 | 1.000 | 1.000 | 1.000 | 0.330 | 0.199 | 1.000 |  | 1.000 | 1.000 | 1.000 | 1.000 | 1.000 | 1.000 |  | 1.000 | **0.041** | 0.092 | 0.238 | 0.377 | 1.000 |
| OTU_6988 | 1.000 | 1.000 | 1.000 | 1.000 | 1.000 | 1.000 |  | 1.000 | 0.110 | **0.003** | 1.000 | 1.000 | 1.000 |  | 1.000 | 1.000 | 1.000 | 1.000 | 1.000 | 1.000 |
| OTU_699 | 1.000 | 1.000 | 0.215 | 1.000 | **0.016** | **0.003** |  | 1.000 | 0.280 | 0.056 | 1.000 | **0.005** | **< 0.001** |  | 1.000 | 1.000 | 0.449 | 1.000 | 1.000 | 0.240 |
| OTU_7 | **< 0.001** | **< 0.001** | **< 0.001** | 1.000 | 1.000 | 1.000 |  | **< 0.001** | **< 0.001** | **< 0.001** | 0.884 | **0.005** | 1.000 |  | **< 0.001** | **< 0.001** | **< 0.001** | 1.000 | 1.000 | 1.000 |
| OTU_70 | 0.563 | **< 0.001** | **< 0.001** | **< 0.001** | **< 0.001** | **< 0.001** |  | 0.563 | **< 0.001** | **< 0.001** | **< 0.001** | **< 0.001** | **< 0.001** |  | **< 0.001** | **< 0.001** | **< 0.001** | **< 0.001** | **< 0.001** | 0.093 |
| OTU_700 | 1.000 | 1.000 | 0.059 | 0.689 | **0.004** | 1.000 |  | 1.000 | **0.010** | **< 0.001** | 0.163 | **0.001** | 1.000 |  | 0.575 | **< 0.001** | **< 0.001** | 0.743 | 1.000 | 1.000 |
| OTU_701 | **0.049** | 0.172 | 0.113 | 1.000 | 1.000 | 1.000 |  | **0.049** | 0.435 | 0.362 | 0.066 | **0.040** | 1.000 |  | **0.045** | **0.006** | 0.090 | 1.000 | 1.000 | 1.000 |
| OTU_7013 | 1.000 | 1.000 | 0.207 | 1.000 | 1.000 | 1.000 |  | 1.000 | **0.004** | **< 0.001** | 1.000 | 0.214 | 1.000 |  | 1.000 | **0.009** | **< 0.001** | 0.085 | **< 0.001** | 1.000 |
| OTU_7017 | 0.288 | 1.000 | 0.128 | **< 0.001** | **< 0.001** | 1.000 |  | 0.288 | 1.000 | 1.000 | 0.688 | 1.000 | 1.000 |  | 1.000 | **0.002** | 1.000 | 0.149 | 1.000 | 1.000 |
| OTU_702 | 1.000 | **< 0.001** | **0.005** | **< 0.001** | **< 0.001** | 1.000 |  | 1.000 | **< 0.001** | **< 0.001** | **< 0.001** | **0.018** | 0.432 |  | 1.000 | **0.002** | **0.004** | **< 0.001** | **< 0.001** | 1.000 |
| OTU_703 | 1.000 | 1.000 | 0.855 | 0.632 | 0.190 | 1.000 |  | 1.000 | 1.000 | **0.041** | 1.000 | **0.029** | 1.000 |  | 1.000 | 1.000 | 1.000 | 1.000 | 1.000 | 1.000 |
| OTU_704 | **0.001** | **< 0.001** | **< 0.001** | 1.000 | 1.000 | 1.000 |  | **0.001** | **< 0.001** | **< 0.001** | 1.000 | 1.000 | 0.472 |  | 0.943 | **< 0.001** | 0.102 | **0.016** | 1.000 | 0.070 |
| OTU_705 | 1.000 | 0.310 | 1.000 | 1.000 | 1.000 | 1.000 |  | 1.000 | 1.000 | 1.000 | 1.000 | 1.000 | 1.000 |  | 1.000 | 1.000 | 0.066 | 1.000 | 0.093 | 0.662 |
| OTU_706 | **< 0.001** | **< 0.001** | **< 0.001** | 0.848 | 1.000 | 0.083 |  | **< 0.001** | **< 0.001** | 0.168 | **0.044** | 1.000 | **0.002** |  | **0.003** | **< 0.001** | **0.003** | 0.471 | 1.000 | 0.179 |
| OTU_707 | 1.000 | 0.075 | 1.000 | 1.000 | 1.000 | 0.866 |  | 1.000 | 1.000 | 1.000 | 1.000 | 1.000 | 1.000 |  | 1.000 | 1.000 | 1.000 | 1.000 | 1.000 | 1.000 |
| OTU_708 | 1.000 | **< 0.001** | 0.956 | **< 0.001** | 0.166 | 0.244 |  | 1.000 | **< 0.001** | 0.667 | **0.002** | 1.000 | 0.287 |  | 0.584 | **< 0.001** | 0.351 | **0.015** | 1.000 | **0.010** |
| OTU_709 | 1.000 | 0.152 | 1.000 | 1.000 | 1.000 | 1.000 |  | 1.000 | 1.000 | 1.000 | 1.000 | 1.000 | 1.000 |  | 1.000 | 1.000 | 1.000 | 1.000 | 1.000 | 1.000 |
| OTU_71 | **< 0.001** | **< 0.001** | **< 0.001** | **0.007** | **0.038** | 1.000 |  | **< 0.001** | **< 0.001** | **< 0.001** | **< 0.001** | **0.011** | 1.000 |  | 0.075 | **< 0.001** | **< 0.001** | **0.001** | **< 0.001** | 1.000 |
| OTU_710 | 1.000 | 1.000 | 0.072 | 1.000 | 1.000 | 1.000 |  | 1.000 | **0.022** | 0.180 | 1.000 | 1.000 | 1.000 |  | 1.000 | 1.000 | 1.000 | 1.000 | 0.956 | 1.000 |
| OTU_711 | 1.000 | 1.000 | 1.000 | 1.000 | 1.000 | 1.000 |  | 1.000 | **0.004** | **0.003** | 1.000 | 1.000 | 1.000 |  | 1.000 | 1.000 | 1.000 | 0.831 | 1.000 | 1.000 |
| OTU_712 | 1.000 | 1.000 | 1.000 | 1.000 | 1.000 | 1.000 |  | 1.000 | 1.000 | 0.362 | 1.000 | 1.000 | 1.000 |  | 1.000 | 1.000 | 1.000 | 1.000 | 1.000 | 1.000 |
| OTU_713 | 0.830 | **0.019** | 1.000 | 1.000 | 1.000 | 1.000 |  | 0.830 | 0.343 | 1.000 | 1.000 | 1.000 | 1.000 |  | 1.000 | 1.000 | 1.000 | 1.000 | 1.000 | 1.000 |
| OTU_714 | 1.000 | 1.000 | 1.000 | 1.000 | 1.000 | 1.000 |  | 1.000 | 1.000 | 0.746 | 1.000 | 1.000 | 1.000 |  | 1.000 | 1.000 | 1.000 | 1.000 | 0.201 | 1.000 |
| OTU_715 | **< 0.001** | **< 0.001** | 0.228 | 1.000 | **0.011** | 0.249 |  | **< 0.001** | **< 0.001** | **0.039** | 1.000 | 0.227 | 0.088 |  | **< 0.001** | **< 0.001** | 0.075 | 1.000 | **0.039** | 1.000 |
| OTU_716 | 1.000 | 1.000 | 0.237 | 1.000 | 1.000 | 1.000 |  | 1.000 | 1.000 | 0.078 | 1.000 | 1.000 | 1.000 |  | 1.000 | 0.371 | 0.312 | 1.000 | 1.000 | 1.000 |
| OTU_717 | 1.000 | 0.907 | 1.000 | 0.997 | 1.000 | 1.000 |  | 1.000 | 1.000 | 1.000 | 0.106 | 0.109 | 1.000 |  | 1.000 | 1.000 | 1.000 | 1.000 | 1.000 | 1.000 |
| OTU_719 | 1.000 | **< 0.001** | 0.628 | 0.055 | 1.000 | 0.074 |  | 1.000 | 1.000 | 0.849 | 1.000 | 1.000 | 1.000 |  | 0.805 | **0.016** | 0.469 | 1.000 | 1.000 | 1.000 |
| OTU_72 | 1.000 | 1.000 | 1.000 | 1.000 | 1.000 | 1.000 |  | 1.000 | 0.154 | 1.000 | 1.000 | 1.000 | 1.000 |  | 1.000 | 1.000 | 1.000 | 1.000 | 1.000 | 1.000 |
| OTU_720 | 1.000 | 1.000 | 1.000 | 1.000 | 1.000 | 1.000 |  | 1.000 | 0.630 | 1.000 | 1.000 | 1.000 | 1.000 |  | 0.085 | **0.036** | 1.000 | 1.000 | 1.000 | 1.000 |
| OTU_721 | 1.000 | 0.899 | **0.004** | 0.819 | **0.002** | 1.000 |  | 1.000 | 1.000 | **0.027** | 0.717 | **0.002** | 1.000 |  | 1.000 | 0.412 | **0.005** | 1.000 | **0.021** | 1.000 |
| OTU_722 | 1.000 | 1.000 | 1.000 | 1.000 | 1.000 | 1.000 |  | 1.000 | 1.000 | 1.000 | 1.000 | 0.299 | 1.000 |  | 1.000 | 1.000 | 0.351 | 1.000 | 1.000 | 1.000 |
| OTU_723 | 1.000 | 1.000 | 1.000 | 1.000 | 1.000 | 1.000 |  | 1.000 | 0.448 | 1.000 | 0.279 | 1.000 | 1.000 |  | 1.000 | 1.000 | 1.000 | 1.000 | 0.827 | 1.000 |
| OTU_724 | 1.000 | 1.000 | 0.454 | 1.000 | 1.000 | 1.000 |  | 1.000 | **< 0.001** | **< 0.001** | **< 0.001** | **0.002** | 1.000 |  | 1.000 | 0.481 | 0.052 | 1.000 | 1.000 | 1.000 |
| OTU_725 | 1.000 | 0.166 | **0.028** | 1.000 | 0.514 | 1.000 |  | 1.000 | **0.028** | 0.169 | 0.268 | 1.000 | 1.000 |  | 0.187 | **0.005** | 0.100 | 1.000 | 1.000 | 1.000 |
| OTU_726 | 1.000 | 1.000 | **< 0.001** | 1.000 | **< 0.001** | **< 0.001** |  | 1.000 | 1.000 | 1.000 | 1.000 | 1.000 | 1.000 |  | 1.000 | 1.000 | **< 0.001** | 1.000 | **< 0.001** | **< 0.001** |
| OTU_727 | 1.000 | 1.000 | 0.211 | 1.000 | 0.651 | 1.000 |  | 1.000 | 0.167 | **0.010** | 1.000 | 1.000 | 1.000 |  | 1.000 | 0.118 | **0.010** | 1.000 | 1.000 | 1.000 |
| OTU_728 | 1.000 | 1.000 | 0.065 | 1.000 | **0.031** | 1.000 |  | 1.000 | 1.000 | 1.000 | 1.000 | 1.000 | 1.000 |  | 1.000 | 1.000 | 0.431 | 1.000 | 1.000 | 1.000 |
| OTU_729 | 1.000 | **0.013** | 1.000 | **0.032** | 1.000 | **0.005** |  | 1.000 | 1.000 | 0.948 | 1.000 | 1.000 | 1.000 |  | 1.000 | **0.010** | 1.000 | 0.367 | 1.000 | **0.003** |
| OTU_73 | 1.000 | **< 0.001** | **< 0.001** | **< 0.001** | **< 0.001** | 0.426 |  | 1.000 | **< 0.001** | **< 0.001** | **< 0.001** | **< 0.001** | 1.000 |  | 1.000 | **< 0.001** | **< 0.001** | **< 0.001** | **< 0.001** | 0.290 |
| OTU_730 | 1.000 | 1.000 | 0.597 | 1.000 | 1.000 | 1.000 |  | 1.000 | 1.000 | 1.000 | 1.000 | 1.000 | 1.000 |  | 1.000 | 1.000 | 1.000 | 1.000 | 1.000 | 1.000 |
| OTU_731 | 0.091 | 0.305 | **0.026** | 1.000 | 1.000 | 1.000 |  | 0.091 | 0.507 | 1.000 | 0.060 | 0.799 | 1.000 |  | 1.000 | 0.073 | **0.019** | 1.000 | 1.000 | 1.000 |
| OTU_733 | 1.000 | 1.000 | **< 0.001** | 1.000 | **< 0.001** | **< 0.001** |  | 1.000 | 0.373 | **< 0.001** | 1.000 | **< 0.001** | 0.105 |  | 1.000 | 1.000 | **< 0.001** | 1.000 | **< 0.001** | **< 0.001** |
| OTU_734 | 1.000 | **< 0.001** | 0.655 | 0.224 | 1.000 | 0.997 |  | 1.000 | **< 0.001** | **< 0.001** | 0.273 | 1.000 | 1.000 |  | 0.943 | **0.002** | 0.385 | 1.000 | 1.000 | 1.000 |
| OTU_735 | 1.000 | 0.607 | 0.203 | **0.012** | **0.002** | 1.000 |  | 1.000 | **< 0.001** | **< 0.001** | 0.868 | 0.088 | 1.000 |  | 1.000 | **< 0.001** | **< 0.001** | 0.666 | 0.566 | 1.000 |
| OTU_736 | **< 0.001** | 0.190 | 1.000 | **0.029** | **< 0.001** | 0.213 |  | **< 0.001** | **0.010** | 0.266 | 1.000 | **0.035** | 1.000 |  | **0.028** | 1.000 | 1.000 | **0.014** | **< 0.001** | 1.000 |
| OTU_737 | 0.122 | **0.003** | **< 0.001** | 1.000 | 0.537 | 1.000 |  | 0.122 | **< 0.001** | **< 0.001** | 1.000 | 1.000 | 1.000 |  | **< 0.001** | **< 0.001** | **< 0.001** | 1.000 | 1.000 | 1.000 |
| OTU_738 | 1.000 | 1.000 | 0.812 | 1.000 | **0.027** | 0.866 |  | 1.000 | 1.000 | 0.233 | 1.000 | 1.000 | 1.000 |  | 1.000 | 1.000 | 1.000 | 1.000 | 1.000 | 1.000 |
| OTU_739 | **< 0.001** | 0.085 | 0.052 | 1.000 | 1.000 | 1.000 |  | **< 0.001** | 0.147 | 0.217 | 1.000 | 1.000 | 1.000 |  | 0.437 | 0.386 | 0.165 | 1.000 | 1.000 | 1.000 |
| OTU_74 | **0.011** | **< 0.001** | **< 0.001** | 0.986 | 0.074 | 1.000 |  | **0.011** | **< 0.001** | **< 0.001** | 0.188 | **0.008** | 1.000 |  | 0.238 | **< 0.001** | **< 0.001** | 0.532 | 0.430 | 1.000 |
| OTU_740 | 1.000 | 0.758 | 0.107 | 1.000 | 1.000 | 1.000 |  | 1.000 | **0.025** | **0.028** | **0.042** | **0.035** | 1.000 |  | 1.000 | 0.810 | 1.000 | 1.000 | 1.000 | 1.000 |
| OTU_742 | 1.000 | 1.000 | 1.000 | 1.000 | 1.000 | 1.000 |  | 1.000 | 0.215 | 1.000 | 1.000 | 1.000 | 1.000 |  | 1.000 | 1.000 | 1.000 | 1.000 | 1.000 | 1.000 |
| OTU_743 | 1.000 | 1.000 | 1.000 | 1.000 | 1.000 | 1.000 |  | 1.000 | **0.021** | 1.000 | 1.000 | 0.766 | 0.140 |  | 1.000 | **0.011** | 1.000 | 0.855 | 1.000 | 0.075 |
| OTU_744 | 1.000 | 1.000 | 1.000 | 0.785 | 1.000 | 1.000 |  | 1.000 | 1.000 | 1.000 | 0.165 | 0.518 | 1.000 |  | 1.000 | 1.000 | 1.000 | 1.000 | 1.000 | 1.000 |
| OTU_745 | 1.000 | 1.000 | 1.000 | 1.000 | 1.000 | 1.000 |  | 1.000 | 1.000 | 0.265 | 1.000 | 1.000 | 0.111 |  | 1.000 | 0.274 | 1.000 | 1.000 | 0.071 | **0.035** |
| OTU_746 | 1.000 | 1.000 | **0.017** | 1.000 | **< 0.001** | 0.114 |  | 1.000 | 1.000 | 0.431 | 1.000 | **0.001** | 0.857 |  | 1.000 | 1.000 | **0.003** | 1.000 | **< 0.001** | **0.037** |
| OTU_747 | 1.000 | 1.000 | 1.000 | 1.000 | 0.937 | 1.000 |  | 1.000 | 1.000 | 1.000 | 1.000 | 1.000 | 1.000 |  | 1.000 | 1.000 | 1.000 | 1.000 | 1.000 | 1.000 |
| OTU_748 | 1.000 | 1.000 | 0.616 | 1.000 | 1.000 | 1.000 |  | 1.000 | 0.130 | 0.052 | 0.187 | 0.063 | 1.000 |  | 1.000 | 1.000 | 1.000 | 1.000 | 1.000 | 1.000 |
| OTU_749 | 1.000 | 1.000 | 1.000 | 1.000 | 1.000 | 1.000 |  | 1.000 | 0.726 | 0.214 | 0.950 | 0.247 | 1.000 |  | 0.480 | 0.342 | 1.000 | 1.000 | 1.000 | 1.000 |
| OTU_75 | 1.000 | 0.268 | 1.000 | 0.664 | 1.000 | 1.000 |  | 1.000 | **0.001** | **< 0.001** | 0.185 | 0.123 | 1.000 |  | 1.000 | **0.002** | **0.009** | 1.000 | 1.000 | 1.000 |
| OTU_750 | 1.000 | 1.000 | 1.000 | 1.000 | 1.000 | 1.000 |  | 1.000 | 1.000 | 1.000 | 1.000 | 1.000 | 1.000 |  | 1.000 | 1.000 | 1.000 | 1.000 | 1.000 | 1.000 |
| OTU_751 | 1.000 | **< 0.001** | **0.002** | 0.090 | 0.689 | 1.000 |  | 1.000 | 0.065 | **0.008** | 1.000 | 1.000 | 1.000 |  | 1.000 | 0.172 | 0.483 | 0.286 | 0.592 | 1.000 |
| OTU_752 | 1.000 | 1.000 | 1.000 | 1.000 | 1.000 | 1.000 |  | 1.000 | **< 0.001** | **< 0.001** | 0.122 | **0.036** | 1.000 |  | 1.000 | 1.000 | 0.284 | 1.000 | 1.000 | 1.000 |
| OTU_753 | 0.411 | 0.617 | 1.000 | 1.000 | 0.990 | 1.000 |  | 0.411 | 1.000 | 0.380 | 0.417 | 1.000 | 1.000 |  | 0.135 | **< 0.001** | 0.197 | 1.000 | 1.000 | 1.000 |
| OTU_754 | 1.000 | 1.000 | 0.601 | 1.000 | 1.000 | 1.000 |  | 1.000 | 0.126 | 1.000 | 0.183 | 1.000 | 1.000 |  | 1.000 | 0.089 | 1.000 | 0.329 | 1.000 | 1.000 |
| OTU_755 | 1.000 | 1.000 | **0.046** | 1.000 | **0.035** | 1.000 |  | 1.000 | 1.000 | 1.000 | 1.000 | 1.000 | 1.000 |  | 1.000 | 0.510 | 0.820 | 1.000 | 1.000 | 1.000 |
| OTU_757 | 0.205 | **< 0.001** | 0.102 | 1.000 | 1.000 | 1.000 |  | 0.205 | 1.000 | 1.000 | 1.000 | 0.089 | 1.000 |  | 1.000 | 0.140 | 1.000 | 1.000 | 1.000 | 0.468 |
| OTU_758 | 1.000 | 0.312 | 0.483 | **0.050** | 0.061 | 1.000 |  | 1.000 | 1.000 | 1.000 | 1.000 | 1.000 | 1.000 |  | 0.658 | 1.000 | 1.000 | 0.454 | 0.257 | 1.000 |
| OTU_759 | 1.000 | 1.000 | 1.000 | 1.000 | 1.000 | 1.000 |  | 1.000 | 1.000 | 0.395 | 1.000 | 1.000 | 1.000 |  | 1.000 | 1.000 | 0.586 | 1.000 | 1.000 | 1.000 |
| OTU_76 | 0.053 | **< 0.001** | 1.000 | **0.004** | **0.015** | **< 0.001** |  | 0.053 | **< 0.001** | 1.000 | 0.106 | **< 0.001** | **< 0.001** |  | **0.013** | **< 0.001** | 1.000 | 0.270 | **0.014** | **< 0.001** |
| OTU_760 | 1.000 | 1.000 | 1.000 | 1.000 | 1.000 | 1.000 |  | 1.000 | 1.000 | 1.000 | 1.000 | 0.785 | 1.000 |  | 1.000 | 1.000 | 1.000 | 1.000 | 1.000 | 1.000 |
| OTU_761 | 0.596 | 0.093 | **< 0.001** | **< 0.001** | **< 0.001** | 1.000 |  | 0.596 | 1.000 | 1.000 | 1.000 | 1.000 | 1.000 |  | 1.000 | **0.022** | 0.083 | 0.249 | 0.621 | 1.000 |
| OTU_762 | 1.000 | 1.000 | 1.000 | 1.000 | 0.973 | 1.000 |  | 1.000 | 0.413 | 0.127 | 0.560 | 0.148 | 1.000 |  | 1.000 | 0.389 | 0.953 | 0.809 | 1.000 | 1.000 |
| OTU_763 | 1.000 | 1.000 | **0.020** | 1.000 | 0.106 | 0.111 |  | 1.000 | 1.000 | 0.098 | **0.006** | **< 0.001** | 1.000 |  | 1.000 | 0.587 | **0.002** | 1.000 | **0.004** | 1.000 |
| OTU_764 | 1.000 | 0.456 | 1.000 | 1.000 | 1.000 | 1.000 |  | 1.000 | **0.005** | 0.053 | 1.000 | 0.259 | 1.000 |  | 1.000 | 1.000 | 1.000 | 1.000 | 1.000 | 1.000 |
| OTU_765 | 1.000 | **0.001** | **0.007** | **0.047** | 0.112 | 1.000 |  | 1.000 | **0.002** | 0.084 | **< 0.001** | **0.001** | 1.000 |  | 1.000 | **0.015** | 1.000 | 0.101 | 1.000 | 0.529 |
| OTU_766 | 1.000 | 1.000 | 1.000 | 0.650 | 1.000 | 0.370 |  | 1.000 | 1.000 | **0.021** | 1.000 | 0.058 | **0.024** |  | 1.000 | 1.000 | 1.000 | 1.000 | 0.778 | 0.210 |
| OTU_767 | 1.000 | 1.000 | 1.000 | 1.000 | 1.000 | 1.000 |  | 1.000 | 1.000 | 1.000 | 1.000 | 1.000 | 1.000 |  | 1.000 | 1.000 | 1.000 | 1.000 | 1.000 | 1.000 |
| OTU_768 | **0.002** | **< 0.001** | **0.001** | 1.000 | 1.000 | 1.000 |  | **0.002** | **< 0.001** | **0.002** | 0.153 | 1.000 | 0.321 |  | 0.358 | **0.002** | **0.010** | 1.000 | 1.000 | 1.000 |
| OTU_769 | 1.000 | 1.000 | 1.000 | 1.000 | 1.000 | 1.000 |  | 1.000 | 1.000 | 1.000 | 1.000 | 1.000 | 1.000 |  | 1.000 | 1.000 | 1.000 | 1.000 | 1.000 | 1.000 |
| OTU_77 | 1.000 | 1.000 | 0.165 | 1.000 | **0.007** | 0.764 |  | 1.000 | 1.000 | 0.999 | 1.000 | 0.149 | 1.000 |  | 1.000 | 1.000 | 0.742 | 1.000 | 0.618 | 1.000 |
| OTU_770 | 1.000 | 1.000 | 0.722 | 1.000 | **0.015** | 1.000 |  | 1.000 | **0.004** | **< 0.001** | 1.000 | 0.555 | 1.000 |  | 1.000 | 1.000 | 0.586 | 1.000 | 0.344 | 0.726 |
| OTU_771 | 1.000 | 1.000 | 1.000 | 1.000 | 1.000 | 0.665 |  | 1.000 | 0.676 | **0.025** | 1.000 | 1.000 | 1.000 |  | 1.000 | 1.000 | 1.000 | 1.000 | 1.000 | 1.000 |
| OTU_772 | **< 0.001** | **< 0.001** | **< 0.001** | 1.000 | 1.000 | 1.000 |  | **< 0.001** | **< 0.001** | **< 0.001** | 0.468 | 1.000 | 1.000 |  | **0.049** | **< 0.001** | **< 0.001** | 1.000 | 1.000 | 1.000 |
| OTU_773 | 1.000 | 1.000 | 1.000 | 1.000 | 1.000 | 1.000 |  | 1.000 | 1.000 | 1.000 | 1.000 | 1.000 | 1.000 |  | 1.000 | 1.000 | 1.000 | 1.000 | 1.000 | 1.000 |
| OTU_774 | 0.654 | 1.000 | 1.000 | 0.908 | 0.102 | 1.000 |  | 0.654 | **0.005** | **0.001** | 1.000 | 1.000 | 1.000 |  | 1.000 | 1.000 | 1.000 | 0.060 | 0.130 | 1.000 |
| OTU_775 | 1.000 | 1.000 | 1.000 | 1.000 | 1.000 | 1.000 |  | 1.000 | 1.000 | 1.000 | 1.000 | 1.000 | 1.000 |  | 1.000 | 1.000 | 1.000 | 1.000 | 1.000 | 1.000 |
| OTU_776 | 1.000 | 1.000 | 0.469 | 1.000 | 0.082 | **0.031** |  | 1.000 | 1.000 | 1.000 | 1.000 | 0.123 | 0.175 |  | 1.000 | 0.974 | 1.000 | 1.000 | 0.523 | 0.088 |
| OTU_777 | 1.000 | 0.989 | 0.146 | 1.000 | 1.000 | 1.000 |  | 1.000 | 1.000 | 0.172 | 1.000 | **0.007** | 1.000 |  | 1.000 | 1.000 | 0.213 | 1.000 | 1.000 | 1.000 |
| OTU_778 | 1.000 | 1.000 | 0.167 | 1.000 | 0.262 | 1.000 |  | 1.000 | 1.000 | 0.060 | 1.000 | 0.073 | 0.944 |  | 1.000 | 1.000 | **< 0.001** | 1.000 | 0.345 | **0.013** |
| OTU_779 | 1.000 | 0.751 | 1.000 | 1.000 | 1.000 | 1.000 |  | 1.000 | 1.000 | 1.000 | 1.000 | 1.000 | 1.000 |  | 1.000 | **0.029** | 0.268 | **0.037** | 0.273 | 1.000 |
| OTU_78 | 1.000 | **< 0.001** | **< 0.001** | **< 0.001** | **< 0.001** | 0.342 |  | 1.000 | **< 0.001** | **< 0.001** | **< 0.001** | **< 0.001** | 0.080 |  | 0.058 | **< 0.001** | **< 0.001** | **< 0.001** | 0.059 | 1.000 |
| OTU_780 | **0.017** | **< 0.001** | **< 0.001** | **< 0.001** | **< 0.001** | 1.000 |  | **0.017** | **< 0.001** | **< 0.001** | **0.031** | 0.056 | 1.000 |  | 0.187 | **< 0.001** | **< 0.001** | **< 0.001** | 0.203 | 1.000 |
| OTU_781 | 1.000 | 1.000 | 1.000 | 1.000 | 1.000 | 1.000 |  | 1.000 | 1.000 | 1.000 | 1.000 | 1.000 | 1.000 |  | 1.000 | 1.000 | 1.000 | 1.000 | 1.000 | 1.000 |
| OTU_782 | 1.000 | 1.000 | 0.158 | 1.000 | 0.130 | 0.779 |  | 1.000 | 1.000 | 1.000 | 1.000 | 1.000 | 1.000 |  | 1.000 | 1.000 | 1.000 | 1.000 | 0.894 | 1.000 |
| OTU_783 | 1.000 | 1.000 | 1.000 | 0.942 | 1.000 | 1.000 |  | 1.000 | 1.000 | 1.000 | 1.000 | 1.000 | 1.000 |  | 1.000 | 1.000 | 1.000 | 1.000 | 1.000 | 1.000 |
| OTU_784 | 1.000 | 1.000 | 1.000 | 1.000 | 1.000 | 1.000 |  | 1.000 | **0.003** | **< 0.001** | 1.000 | 0.781 | 1.000 |  | 1.000 | 1.000 | 1.000 | 1.000 | 1.000 | 1.000 |
| OTU_785 | 1.000 | 1.000 | 1.000 | 1.000 | 1.000 | 1.000 |  | 1.000 | 1.000 | 1.000 | 1.000 | 1.000 | 1.000 |  | 1.000 | **0.031** | 1.000 | **0.022** | 1.000 | **0.012** |
| OTU_786 | 1.000 | 0.210 | **0.004** | 1.000 | **0.018** | 1.000 |  | 1.000 | 0.279 | **0.003** | 1.000 | 0.158 | 1.000 |  | 1.000 | 0.136 | **0.024** | 1.000 | 0.598 | 1.000 |
| OTU_787 | 0.074 | 0.142 | 0.438 | 1.000 | 1.000 | 1.000 |  | 0.074 | 1.000 | 1.000 | 1.000 | 1.000 | 1.000 |  | 1.000 | 1.000 | 1.000 | 1.000 | 0.073 | 1.000 |
| OTU_788 | 1.000 | 1.000 | 1.000 | 1.000 | 1.000 | 1.000 |  | 1.000 | 1.000 | 0.928 | 1.000 | 1.000 | 1.000 |  | 1.000 | 1.000 | 1.000 | 1.000 | 1.000 | 1.000 |
| OTU_789 | 1.000 | 1.000 | 0.076 | 1.000 | 0.095 | 0.338 |  | 1.000 | 1.000 | **0.014** | 1.000 | **0.034** | **0.020** |  | 1.000 | 1.000 | 1.000 | 1.000 | 1.000 | 0.306 |
| OTU_79 | 1.000 | 0.434 | **< 0.001** | **0.003** | **< 0.001** | **< 0.001** |  | 1.000 | **< 0.001** | **< 0.001** | 1.000 | **< 0.001** | **0.002** |  | 1.000 | 0.076 | **< 0.001** | 1.000 | **< 0.001** | **0.001** |
| OTU_790 | 1.000 | 1.000 | **0.041** | 1.000 | **< 0.001** | 0.181 |  | 1.000 | **0.047** | **< 0.001** | 1.000 | 0.749 | 1.000 |  | 1.000 | 1.000 | **0.017** | 1.000 | 0.850 | 1.000 |
| OTU_791 | 1.000 | **0.004** | 1.000 | **< 0.001** | 1.000 | **0.017** |  | 1.000 | **< 0.001** | 1.000 | 1.000 | 0.231 | **0.012** |  | 1.000 | **0.006** | 1.000 | **< 0.001** | 1.000 | **0.004** |
| OTU_792 | 1.000 | 1.000 | 1.000 | 1.000 | 1.000 | 0.844 |  | 1.000 | 0.569 | 0.413 | 1.000 | 1.000 | 1.000 |  | 1.000 | 1.000 | 1.000 | 1.000 | 1.000 | 1.000 |
| OTU_793 | 1.000 | 1.000 | 1.000 | 0.826 | 1.000 | 0.074 |  | 1.000 | 1.000 | 1.000 | 1.000 | 1.000 | 1.000 |  | 1.000 | 1.000 | 1.000 | 1.000 | 1.000 | 1.000 |
| OTU_794 | 1.000 | 1.000 | **0.022** | 1.000 | 0.074 | 1.000 |  | 1.000 | 0.540 | 0.950 | 0.985 | 1.000 | 1.000 |  | 1.000 | 0.109 | **0.010** | 1.000 | 0.420 | 1.000 |
| OTU_795 | 1.000 | 1.000 | 1.000 | 1.000 | 1.000 | 1.000 |  | 1.000 | 1.000 | 1.000 | 1.000 | 0.793 | 1.000 |  | 1.000 | 1.000 | 1.000 | 1.000 | 1.000 | 1.000 |
| OTU_796 | 1.000 | 1.000 | 0.387 | 1.000 | 1.000 | 1.000 |  | 1.000 | 0.457 | 0.085 | 0.617 | 0.102 | 1.000 |  | 1.000 | 1.000 | 1.000 | 1.000 | 1.000 | 1.000 |
| OTU_797 | 1.000 | 0.387 | 1.000 | 0.916 | 1.000 | 0.419 |  | 1.000 | 1.000 | 1.000 | 1.000 | 1.000 | 1.000 |  | 1.000 | 1.000 | 1.000 | 1.000 | 1.000 | 1.000 |
| OTU_798 | 0.178 | **< 0.001** | **< 0.001** | 1.000 | 1.000 | 1.000 |  | 0.178 | **< 0.001** | **< 0.001** | 1.000 | 0.096 | 1.000 |  | **0.010** | **< 0.001** | **< 0.001** | 1.000 | 1.000 | 1.000 |
| OTU_799 | 1.000 | 1.000 | 1.000 | 1.000 | 1.000 | 1.000 |  | 1.000 | 1.000 | 1.000 | 1.000 | 1.000 | 1.000 |  | 1.000 | 0.474 | 0.666 | 1.000 | 1.000 | 1.000 |
| OTU_8 | **< 0.001** | **< 0.001** | **0.002** | **< 0.001** | 1.000 | **< 0.001** |  | **< 0.001** | **< 0.001** | **< 0.001** | **< 0.001** | 1.000 | **< 0.001** |  | **< 0.001** | **< 0.001** | **< 0.001** | **< 0.001** | 1.000 | **< 0.001** |
| OTU_80 | **0.001** | **< 0.001** | 1.000 | 1.000 | **0.025** | **0.017** |  | **0.001** | 0.218 | 1.000 | 1.000 | **0.044** | 0.056 |  | 1.000 | 1.000 | 1.000 | 1.000 | 1.000 | 1.000 |
| OTU_800 | 1.000 | **0.015** | 1.000 | **< 0.001** | 0.196 | 1.000 |  | 1.000 | **< 0.001** | **0.012** | **< 0.001** | **0.003** | 1.000 |  | 1.000 | 1.000 | 1.000 | 0.168 | 1.000 | 1.000 |
| OTU_801 | 1.000 | 1.000 | 1.000 | 1.000 | 1.000 | 1.000 |  | 1.000 | 1.000 | 1.000 | 1.000 | 1.000 | 1.000 |  | 1.000 | 1.000 | 1.000 | 1.000 | 1.000 | 1.000 |
| OTU_802 | 1.000 | 1.000 | 0.162 | 1.000 | 1.000 | 1.000 |  | 1.000 | 1.000 | **0.002** | 1.000 | 1.000 | 0.823 |  | 1.000 | 1.000 | 1.000 | 1.000 | 1.000 | 1.000 |
| OTU_803 | 1.000 | 1.000 | 1.000 | 1.000 | 1.000 | 1.000 |  | 1.000 | 1.000 | **0.028** | 1.000 | 1.000 | 1.000 |  | 1.000 | 1.000 | 1.000 | 1.000 | 1.000 | 1.000 |
| OTU_804 | 1.000 | 1.000 | 0.621 | 1.000 | 0.608 | 1.000 |  | 1.000 | 1.000 | 1.000 | 1.000 | 0.759 | 1.000 |  | 1.000 | 1.000 | 1.000 | 1.000 | 1.000 | 1.000 |
| OTU_805 | 0.546 | **0.012** | **0.005** | 1.000 | 1.000 | 1.000 |  | 0.546 | 0.060 | 0.062 | 0.094 | 0.076 | 1.000 |  | 0.596 | **0.031** | **0.018** | 1.000 | 1.000 | 1.000 |
| OTU_806 | 1.000 | 1.000 | 1.000 | 1.000 | 1.000 | 1.000 |  | 1.000 | 0.545 | 1.000 | 1.000 | 1.000 | 1.000 |  | 1.000 | 1.000 | 1.000 | 1.000 | 1.000 | 1.000 |
| OTU_807 | 1.000 | 1.000 | 0.479 | 1.000 | **0.050** | **0.043** |  | 1.000 | 0.657 | 1.000 | 1.000 | 0.226 | 0.365 |  | 1.000 | 1.000 | 1.000 | 1.000 | 1.000 | 1.000 |
| OTU_808 | 1.000 | 1.000 | 1.000 | 1.000 | 1.000 | 1.000 |  | 1.000 | **0.044** | 1.000 | 0.739 | 1.000 | **0.011** |  | 1.000 | 0.895 | 1.000 | 0.730 | 1.000 | 1.000 |
| OTU_809 | 1.000 | **< 0.001** | **< 0.001** | **< 0.001** | **< 0.001** | **< 0.001** |  | 1.000 | **< 0.001** | **< 0.001** | **< 0.001** | **< 0.001** | **< 0.001** |  | 1.000 | **< 0.001** | **< 0.001** | **< 0.001** | **< 0.001** | **< 0.001** |
| OTU_81 | **< 0.001** | **< 0.001** | **0.001** | 1.000 | 1.000 | 1.000 |  | **< 0.001** | **< 0.001** | **< 0.001** | 0.871 | 1.000 | 1.000 |  | 1.000 | **0.050** | 1.000 | 1.000 | 1.000 | 1.000 |
| OTU_810 | 0.225 | 1.000 | 1.000 | **0.016** | **0.001** | 1.000 |  | 0.225 | 0.415 | 0.389 | **0.003** | **0.002** | 1.000 |  | 1.000 | 1.000 | 0.212 | 1.000 | 0.222 | 1.000 |
| OTU_811 | 1.000 | 1.000 | 0.058 | 1.000 | 1.000 | **0.035** |  | 1.000 | 1.000 | **< 0.001** | 1.000 | **< 0.001** | **0.018** |  | 1.000 | 1.000 | 1.000 | 1.000 | 1.000 | 1.000 |
| OTU_812 | 1.000 | 1.000 | 0.874 | 0.916 | 0.096 | 1.000 |  | 1.000 | 0.092 | 0.074 | 1.000 | 1.000 | 1.000 |  | 1.000 | 1.000 | 0.186 | 1.000 | 1.000 | 1.000 |
| OTU_813 | 0.387 | 1.000 | 1.000 | 1.000 | **0.044** | 1.000 |  | 0.387 | 1.000 | 1.000 | 1.000 | 1.000 | 1.000 |  | 1.000 | 1.000 | 1.000 | 1.000 | 1.000 | 1.000 |
| OTU_815 | 1.000 | **0.031** | 1.000 | **0.020** | 1.000 | 1.000 |  | 1.000 | **< 0.001** | 1.000 | **< 0.001** | 1.000 | **< 0.001** |  | 1.000 | **< 0.001** | 1.000 | **0.020** | 1.000 | **< 0.001** |
| OTU_816 | 1.000 | 1.000 | 1.000 | 1.000 | 1.000 | 1.000 |  | 1.000 | 1.000 | 1.000 | 1.000 | 1.000 | 1.000 |  | 1.000 | 1.000 | 1.000 | 1.000 | 1.000 | 1.000 |
| OTU_817 | 1.000 | 0.407 | 1.000 | 0.258 | 1.000 | 1.000 |  | 1.000 | 1.000 | 1.000 | 1.000 | 1.000 | 1.000 |  | 1.000 | 1.000 | 1.000 | 1.000 | 1.000 | 1.000 |
| OTU_818 | 1.000 | 1.000 | 0.056 | 1.000 | 0.127 | 1.000 |  | 1.000 | 0.287 | 0.262 | 1.000 | 1.000 | 1.000 |  | 1.000 | 0.730 | 0.364 | 1.000 | 1.000 | 1.000 |
| OTU_819 | 1.000 | 1.000 | 1.000 | 1.000 | 1.000 | 1.000 |  | 1.000 | 1.000 | 0.122 | 1.000 | 1.000 | 1.000 |  | 1.000 | 1.000 | 1.000 | 1.000 | 1.000 | 1.000 |
| OTU_82 | 0.610 | **< 0.001** | 1.000 | 1.000 | 1.000 | 0.306 |  | 0.610 | **< 0.001** | 0.773 | **0.030** | 1.000 | **0.019** |  | 1.000 | **< 0.001** | 1.000 | 1.000 | 1.000 | 0.239 |
| OTU_820 | 1.000 | 1.000 | 1.000 | 0.363 | 0.230 | 1.000 |  | 1.000 | 1.000 | 1.000 | 1.000 | 1.000 | 1.000 |  | 1.000 | 1.000 | 1.000 | 1.000 | 1.000 | 1.000 |
| OTU_821 | 1.000 | 1.000 | 1.000 | 0.239 | 1.000 | 1.000 |  | 1.000 | 0.054 | 0.255 | 1.000 | 1.000 | 1.000 |  | 1.000 | 1.000 | 1.000 | 1.000 | 1.000 | 1.000 |
| OTU_822 | **0.006** | **0.005** | 1.000 | 1.000 | **0.016** | **0.050** |  | **0.006** | **0.003** | 1.000 | 1.000 | **0.001** | **0.002** |  | 1.000 | 1.000 | 1.000 | 1.000 | 0.196 | 0.087 |
| OTU_823 | 1.000 | 1.000 | 1.000 | 1.000 | 1.000 | 1.000 |  | 1.000 | 1.000 | 0.736 | 1.000 | 1.000 | 1.000 |  | 1.000 | 1.000 | 1.000 | 1.000 | 1.000 | 1.000 |
| OTU_824 | 1.000 | 1.000 | 1.000 | 1.000 | 1.000 | 0.737 |  | 1.000 | 1.000 | 0.438 | 1.000 | 1.000 | 1.000 |  | 1.000 | 1.000 | 1.000 | 1.000 | 1.000 | 1.000 |
| OTU_825 | 0.074 | 0.066 | 1.000 | 1.000 | 1.000 | 1.000 |  | 0.074 | 1.000 | 1.000 | 1.000 | 1.000 | 1.000 |  | 1.000 | 1.000 | 1.000 | 1.000 | 1.000 | 1.000 |
| OTU_826 | 1.000 | 1.000 | 1.000 | 1.000 | 0.314 | 1.000 |  | 1.000 | 1.000 | 1.000 | 1.000 | 1.000 | 1.000 |  | 1.000 | 1.000 | 0.658 | 1.000 | 0.600 | 0.521 |
| OTU_827 | 1.000 | 1.000 | 1.000 | 1.000 | 1.000 | 1.000 |  | 1.000 | 1.000 | 1.000 | 1.000 | 1.000 | 1.000 |  | 1.000 | 1.000 | 1.000 | 1.000 | 1.000 | 1.000 |
| OTU_828 | 1.000 | 1.000 | 0.108 | 1.000 | 0.063 | 1.000 |  | 1.000 | **0.024** | **< 0.001** | 1.000 | 0.167 | 0.329 |  | 1.000 | 1.000 | 1.000 | 1.000 | 1.000 | 1.000 |
| OTU_829 | 1.000 | 1.000 | 1.000 | 0.372 | 0.225 | 1.000 |  | 1.000 | **0.007** | **0.004** | 1.000 | 1.000 | 1.000 |  | 1.000 | 1.000 | 1.000 | 1.000 | 1.000 | 1.000 |
| OTU_83 | **< 0.001** | **< 0.001** | 0.115 | 1.000 | 0.604 | 0.440 |  | **< 0.001** | 0.196 | 1.000 | 1.000 | 1.000 | 1.000 |  | **< 0.001** | **< 0.001** | 1.000 | 1.000 | **0.011** | **0.044** |
| OTU_830 | **0.014** | **0.023** | **0.002** | 1.000 | 1.000 | 1.000 |  | **0.014** | 0.124 | 0.220 | 1.000 | 1.000 | 1.000 |  | **0.031** | 1.000 | 1.000 | 1.000 | 0.160 | 1.000 |
| OTU_831 | 1.000 | 1.000 | **< 0.001** | 1.000 | **< 0.001** | **0.007** |  | 1.000 | **0.022** | **0.002** | **0.036** | **0.002** | 1.000 |  | 1.000 | 1.000 | **0.003** | 0.372 | **< 0.001** | 1.000 |
| OTU_833 | **0.027** | **< 0.001** | 0.197 | 1.000 | 1.000 | 0.149 |  | **0.027** | **0.009** | 1.000 | 1.000 | 1.000 | 0.516 |  | 1.000 | **0.006** | 0.083 | 1.000 | 1.000 | 1.000 |
| OTU_834 | 1.000 | 0.103 | 1.000 | 1.000 | 1.000 | 1.000 |  | 1.000 | 1.000 | 1.000 | 0.511 | 0.529 | 1.000 |  | 1.000 | 0.074 | 1.000 | 1.000 | 1.000 | 1.000 |
| OTU_836 | **< 0.001** | **< 0.001** | **0.003** | 1.000 | 1.000 | 0.670 |  | **< 0.001** | **< 0.001** | 0.137 | 1.000 | 1.000 | 1.000 |  | 0.079 | **< 0.001** | 0.138 | 0.264 | 1.000 | 0.061 |
| OTU_837 | 1.000 | **< 0.001** | **< 0.001** | **< 0.001** | 0.133 | 1.000 |  | 1.000 | **0.001** | **0.010** | 1.000 | 1.000 | 1.000 |  | 1.000 | 1.000 | 1.000 | 1.000 | 1.000 | 1.000 |
| OTU_838 | 1.000 | **0.005** | 0.062 | 0.457 | 1.000 | 1.000 |  | 1.000 | **< 0.001** | **0.001** | 0.646 | 1.000 | 1.000 |  | 0.286 | **0.042** | **0.014** | 1.000 | 1.000 | 1.000 |
| OTU_839 | 1.000 | 1.000 | 1.000 | 1.000 | 1.000 | 1.000 |  | 1.000 | 1.000 | 0.911 | 1.000 | 0.909 | 1.000 |  | 1.000 | 0.198 | 1.000 | 1.000 | 1.000 | 1.000 |
| OTU_84 | **0.008** | **< 0.001** | **< 0.001** | **0.002** | 1.000 | 1.000 |  | **0.008** | **< 0.001** | **0.003** | **< 0.001** | 1.000 | **0.001** |  | 0.430 | **< 0.001** | **< 0.001** | **< 0.001** | **0.021** | 0.944 |
| OTU_840 | 1.000 | 1.000 | 0.092 | 1.000 | **0.025** | 0.297 |  | 1.000 | 1.000 | 1.000 | 1.000 | 0.367 | 1.000 |  | 1.000 | 0.186 | **< 0.001** | 1.000 | 0.106 | 1.000 |
| OTU_841 | 1.000 | 1.000 | 1.000 | 1.000 | 0.924 | 1.000 |  | 1.000 | 0.809 | 1.000 | 1.000 | 1.000 | 1.000 |  | 1.000 | 1.000 | 1.000 | 1.000 | 0.682 | 1.000 |
| OTU_842 | 1.000 | 1.000 | 1.000 | 1.000 | 1.000 | 1.000 |  | 1.000 | **0.003** | 1.000 | 1.000 | 1.000 | 0.231 |  | 1.000 | 1.000 | 1.000 | 1.000 | 1.000 | 1.000 |
| OTU_843 | 1.000 | 1.000 | 1.000 | 1.000 | 1.000 | 1.000 |  | 1.000 | 1.000 | 0.369 | 1.000 | 0.063 | 1.000 |  | 1.000 | 1.000 | 0.374 | 1.000 | 1.000 | 1.000 |
| OTU_844 | 1.000 | 1.000 | 0.296 | 1.000 | 0.251 | 0.347 |  | 1.000 | 1.000 | 1.000 | 1.000 | 1.000 | 1.000 |  | 1.000 | 1.000 | **0.043** | 1.000 | **0.017** | 1.000 |
| OTU_845 | 1.000 | 1.000 | **0.003** | 1.000 | 0.140 | **0.041** |  | 1.000 | 0.600 | **< 0.001** | 0.466 | 1.000 | **0.005** |  | 1.000 | 1.000 | **0.002** | 1.000 | **0.048** | 0.116 |
| OTU_846 | **0.004** | **0.002** | 0.315 | 1.000 | 0.998 | 1.000 |  | **0.004** | 0.247 | 1.000 | 1.000 | 1.000 | 1.000 |  | 1.000 | **0.044** | 0.353 | 1.000 | 1.000 | 1.000 |
| OTU_847 | 1.000 | **0.011** | **0.010** | 1.000 | 1.000 | 1.000 |  | 1.000 | **< 0.001** | **< 0.001** | 1.000 | 1.000 | 1.000 |  | 0.164 | 0.408 | **0.048** | 1.000 | 1.000 | 1.000 |
| OTU_848 | 1.000 | 1.000 | 1.000 | 1.000 | 1.000 | 1.000 |  | 1.000 | **0.004** | 1.000 | 1.000 | **0.017** | **0.028** |  | 1.000 | 1.000 | 1.000 | 1.000 | 1.000 | 1.000 |
| OTU_849 | 1.000 | 1.000 | 1.000 | 1.000 | 1.000 | 1.000 |  | 1.000 | 1.000 | 1.000 | 1.000 | 1.000 | 1.000 |  | 1.000 | 1.000 | 1.000 | 1.000 | 1.000 | 1.000 |
| OTU_85 | 1.000 | **< 0.001** | 0.197 | **0.001** | 1.000 | 0.810 |  | 1.000 | **< 0.001** | **< 0.001** | **0.006** | 0.056 | 1.000 |  | 0.443 | **< 0.001** | **< 0.001** | **0.021** | 0.675 | 1.000 |
| OTU_850 | 1.000 | 1.000 | 1.000 | 1.000 | 1.000 | 1.000 |  | 1.000 | 0.149 | **< 0.001** | 1.000 | 1.000 | 1.000 |  | 1.000 | 1.000 | 0.873 | 1.000 | 1.000 | 1.000 |
| OTU_851 | 1.000 | 0.475 | **0.048** | 1.000 | 1.000 | 1.000 |  | 1.000 | **< 0.001** | **< 0.001** | 1.000 | 0.947 | 1.000 |  | 1.000 | 1.000 | 0.979 | 1.000 | 1.000 | 1.000 |
| OTU_852 | 1.000 | 1.000 | 1.000 | 1.000 | 1.000 | 1.000 |  | 1.000 | 1.000 | 0.628 | 1.000 | 0.079 | 1.000 |  | 1.000 | 1.000 | 1.000 | 1.000 | 0.404 | 1.000 |
| OTU_853 | 1.000 | **0.016** | 1.000 | 1.000 | 1.000 | **0.046** |  | 1.000 | 1.000 | 1.000 | 1.000 | 1.000 | 0.786 |  | 1.000 | 1.000 | 1.000 | 0.830 | 1.000 | 1.000 |
| OTU_854 | 1.000 | 1.000 | 1.000 | 1.000 | 1.000 | 1.000 |  | 1.000 | 1.000 | **0.023** | 1.000 | 1.000 | 1.000 |  | 1.000 | 1.000 | 1.000 | 1.000 | 1.000 | 1.000 |
| OTU_855 | 1.000 | 1.000 | 1.000 | 0.192 | **0.049** | 1.000 |  | 1.000 | 0.692 | **0.015** | 1.000 | 1.000 | 1.000 |  | 1.000 | 1.000 | 1.000 | 1.000 | 1.000 | 1.000 |
| OTU_856 | 1.000 | 1.000 | **< 0.001** | 1.000 | **< 0.001** | **< 0.001** |  | 1.000 | 1.000 | **< 0.001** | 1.000 | **< 0.001** | **< 0.001** |  | 1.000 | 1.000 | **< 0.001** | 1.000 | **< 0.001** | **< 0.001** |
| OTU_857 | 1.000 | 1.000 | 1.000 | 1.000 | 1.000 | 1.000 |  | 1.000 | 1.000 | 1.000 | 1.000 | 1.000 | 1.000 |  | 0.498 | **0.002** | 1.000 | 1.000 | 0.106 | **0.001** |
| OTU_859 | 1.000 | 0.442 | 0.641 | 1.000 | 1.000 | 1.000 |  | 1.000 | 0.115 | 0.386 | 1.000 | 1.000 | 1.000 |  | 0.619 | 0.101 | 0.431 | 1.000 | 1.000 | 1.000 |
| OTU_86 | **< 0.001** | **< 0.001** | **< 0.001** | 0.917 | 1.000 | 0.173 |  | **< 0.001** | **< 0.001** | **< 0.001** | **0.007** | 1.000 | **0.022** |  | **< 0.001** | **< 0.001** | **< 0.001** | **0.002** | 1.000 | **< 0.001** |
| OTU_860 | 1.000 | 1.000 | 1.000 | 1.000 | 0.697 | 1.000 |  | 1.000 | 0.620 | 0.107 | 0.822 | 0.125 | 1.000 |  | 1.000 | 0.156 | 1.000 | 0.066 | 1.000 | 1.000 |
| OTU_861 | 1.000 | **0.002** | 0.081 | 0.181 | 1.000 | 1.000 |  | 1.000 | 1.000 | 1.000 | 1.000 | 1.000 | 1.000 |  | 1.000 | 0.549 | 1.000 | 1.000 | 1.000 | 1.000 |
| OTU_862 | 0.220 | 0.452 | 1.000 | 1.000 | 0.778 | 1.000 |  | 0.220 | 1.000 | 1.000 | 1.000 | 1.000 | 1.000 |  | 1.000 | 1.000 | 1.000 | 1.000 | 1.000 | 1.000 |
| OTU_863 | 0.158 | **0.011** | **0.002** | 1.000 | 1.000 | 1.000 |  | 0.158 | 0.255 | 0.627 | 0.127 | 0.249 | 1.000 |  | 1.000 | **0.027** | **0.002** | 1.000 | 0.704 | 1.000 |
| OTU_864 | 1.000 | 1.000 | 1.000 | 1.000 | 0.357 | 1.000 |  | 1.000 | 1.000 | 1.000 | 1.000 | 1.000 | 1.000 |  | 0.150 | 1.000 | 1.000 | 1.000 | 0.545 | 1.000 |
| OTU_865 | **0.009** | **0.049** | **0.038** | 1.000 | 1.000 | 1.000 |  | **0.009** | 1.000 | 1.000 | 1.000 | 1.000 | 1.000 |  | 1.000 | 1.000 | 1.000 | 1.000 | 1.000 | 1.000 |
| OTU_866 | 1.000 | 1.000 | 1.000 | 1.000 | 1.000 | 1.000 |  | 1.000 | 1.000 | 1.000 | 1.000 | 1.000 | 1.000 |  | 1.000 | 1.000 | 1.000 | 1.000 | 1.000 | 1.000 |
| OTU_867 | 0.138 | 0.106 | 0.057 | 1.000 | 1.000 | 1.000 |  | 0.138 | 1.000 | 1.000 | 0.070 | 0.080 | 1.000 |  | 1.000 | 1.000 | 1.000 | 1.000 | 1.000 | 1.000 |
| OTU_868 | 1.000 | 1.000 | 1.000 | 1.000 | 1.000 | 1.000 |  | 1.000 | 1.000 | 1.000 | 1.000 | 1.000 | 1.000 |  | 1.000 | 1.000 | 1.000 | 1.000 | 1.000 | 1.000 |
| OTU_869 | 1.000 | 0.212 | **0.002** | 0.467 | **0.003** | 1.000 |  | 1.000 | **0.009** | 0.102 | **< 0.001** | **< 0.001** | 1.000 |  | 1.000 | **0.008** | **0.011** | **0.009** | **0.009** | 1.000 |
| OTU_87 | 1.000 | **0.002** | 1.000 | 0.124 | 1.000 | 0.123 |  | 1.000 | **0.030** | 1.000 | 1.000 | 1.000 | 0.137 |  | 1.000 | **< 0.001** | 1.000 | **0.033** | 1.000 | 0.630 |
| OTU_870 | 1.000 | 1.000 | 1.000 | 1.000 | 1.000 | 1.000 |  | 1.000 | 0.462 | 1.000 | 1.000 | 1.000 | 1.000 |  | 1.000 | 1.000 | 1.000 | 1.000 | 1.000 | 0.880 |
| OTU_871 | 1.000 | **0.014** | 1.000 | **< 0.001** | 0.956 | 0.192 |  | 1.000 | **< 0.001** | 0.077 | 0.140 | 1.000 | 0.574 |  | 1.000 | **0.008** | 1.000 | **0.033** | 1.000 | 0.208 |
| OTU_872 | 0.180 | 1.000 | 1.000 | 1.000 | 1.000 | 1.000 |  | 0.180 | 1.000 | 1.000 | 1.000 | 1.000 | 1.000 |  | 1.000 | 1.000 | 1.000 | 1.000 | 1.000 | 1.000 |
| OTU_873 | 1.000 | **0.030** | **0.012** | 0.498 | 0.174 | 1.000 |  | 1.000 | 1.000 | 0.980 | 1.000 | 1.000 | 1.000 |  | 1.000 | 0.173 | 1.000 | 0.888 | 1.000 | 1.000 |
| OTU_874 | 1.000 | **0.039** | 1.000 | **0.003** | 0.814 | 0.778 |  | 1.000 | **< 0.001** | 0.805 | 0.280 | 1.000 | **0.045** |  | 1.000 | **0.010** | 1.000 | 1.000 | 1.000 | 0.317 |
| OTU_875 | 1.000 | 1.000 | 1.000 | 0.890 | 1.000 | 1.000 |  | 1.000 | 1.000 | 1.000 | 1.000 | 1.000 | 1.000 |  | 1.000 | 1.000 | 0.718 | 1.000 | 0.578 | 1.000 |
| OTU_876 | 0.901 | **0.043** | 0.058 | 1.000 | 1.000 | 1.000 |  | 0.901 | 0.192 | 1.000 | 0.259 | 1.000 | 1.000 |  | 1.000 | 1.000 | 1.000 | 1.000 | 1.000 | 1.000 |
| OTU_877 | 1.000 | 1.000 | 1.000 | 1.000 | 1.000 | 1.000 |  | 1.000 | 0.135 | 1.000 | 1.000 | 1.000 | 1.000 |  | 1.000 | 1.000 | 1.000 | 1.000 | 1.000 | 1.000 |
| OTU_878 | 0.144 | 0.163 | 1.000 | 1.000 | 1.000 | 1.000 |  | 0.144 | 1.000 | 0.875 | 1.000 | **0.030** | **0.008** |  | 1.000 | 1.000 | 1.000 | 1.000 | 0.835 | 1.000 |
| OTU_879 | 1.000 | 1.000 | 1.000 | 0.411 | 0.098 | 1.000 |  | 1.000 | 1.000 | 1.000 | 1.000 | 1.000 | 1.000 |  | 1.000 | 1.000 | 1.000 | 0.445 | 0.344 | 1.000 |
| OTU_88 | **0.018** | **< 0.001** | **< 0.001** | **0.017** | 0.322 | 1.000 |  | **0.018** | **< 0.001** | **0.017** | **< 0.001** | 1.000 | **0.004** |  | **< 0.001** | **< 0.001** | **< 0.001** | **0.016** | 1.000 | 1.000 |
| OTU_880 | 1.000 | 0.483 | **0.022** | 1.000 | 0.572 | 1.000 |  | 1.000 | 1.000 | 0.130 | 0.174 | **0.015** | 1.000 |  | 1.000 | 0.106 | 0.083 | 1.000 | 1.000 | 1.000 |
| OTU_881 | 1.000 | 1.000 | 1.000 | 1.000 | 1.000 | 1.000 |  | 1.000 | 1.000 | 1.000 | 1.000 | 1.000 | 1.000 |  | 1.000 | 1.000 | 1.000 | 1.000 | 1.000 | 1.000 |
| OTU_882 | 1.000 | 0.478 | 0.435 | 1.000 | 1.000 | 1.000 |  | 1.000 | 1.000 | 1.000 | 1.000 | 1.000 | 1.000 |  | 1.000 | 1.000 | 1.000 | 1.000 | 1.000 | 1.000 |
| OTU_883 | 1.000 | 1.000 | 0.865 | 1.000 | **0.027** | 0.278 |  | 1.000 | 1.000 | **0.001** | 1.000 | **0.002** | 0.297 |  | 1.000 | 1.000 | 0.081 | 1.000 | 1.000 | 1.000 |
| OTU_884 | 1.000 | 1.000 | 1.000 | 1.000 | 1.000 | 1.000 |  | 1.000 | 0.136 | 1.000 | 1.000 | 1.000 | 0.316 |  | 1.000 | 1.000 | 1.000 | 1.000 | 1.000 | 1.000 |
| OTU_885 | 1.000 | **0.021** | **< 0.001** | **0.013** | **< 0.001** | 1.000 |  | 1.000 | **< 0.001** | **0.004** | **0.022** | 0.856 | 1.000 |  | 1.000 | 0.063 | **0.029** | 0.121 | **0.043** | 1.000 |
| OTU_887 | 1.000 | 1.000 | 1.000 | 1.000 | 1.000 | 1.000 |  | 1.000 | 1.000 | 1.000 | 1.000 | 1.000 | 1.000 |  | 1.000 | 1.000 | 0.855 | 1.000 | 1.000 | 1.000 |
| OTU_889 | 1.000 | 1.000 | 1.000 | 1.000 | 1.000 | 1.000 |  | 1.000 | 1.000 | 1.000 | 1.000 | 1.000 | 1.000 |  | 1.000 | 1.000 | 1.000 | 1.000 | 1.000 | 1.000 |
| OTU_89 | **< 0.001** | **< 0.001** | 0.257 | **< 0.001** | **< 0.001** | **< 0.001** |  | **< 0.001** | **< 0.001** | **< 0.001** | **< 0.001** | **< 0.001** | **< 0.001** |  | **< 0.001** | **< 0.001** | **< 0.001** | **0.005** | **< 0.001** | **< 0.001** |
| OTU_890 | 1.000 | 1.000 | 1.000 | 1.000 | 1.000 | 1.000 |  | 1.000 | 1.000 | 1.000 | 1.000 | 1.000 | 1.000 |  | 1.000 | 1.000 | 1.000 | 1.000 | 1.000 | 1.000 |
| OTU_891 | 1.000 | 1.000 | 0.107 | 1.000 | **0.035** | **0.013** |  | 1.000 | **< 0.001** | 0.788 | 1.000 | 0.395 | **0.002** |  | 1.000 | 1.000 | 1.000 | 1.000 | 0.404 | **0.028** |
| OTU_893 | 1.000 | 1.000 | 1.000 | 1.000 | 1.000 | 1.000 |  | 1.000 | 0.155 | 1.000 | 0.528 | 1.000 | 0.125 |  | 1.000 | 1.000 | 1.000 | 1.000 | 1.000 | 1.000 |
| OTU_894 | 1.000 | 0.399 | 1.000 | 1.000 | 1.000 | 1.000 |  | 1.000 | 1.000 | 1.000 | 1.000 | 1.000 | 1.000 |  | 1.000 | 0.263 | 0.189 | 1.000 | 1.000 | 1.000 |
| OTU_895 | 1.000 | 1.000 | 1.000 | 1.000 | 1.000 | 1.000 |  | 1.000 | 1.000 | 0.158 | 1.000 | 0.187 | 1.000 |  | 1.000 | 1.000 | 1.000 | 1.000 | 1.000 | 1.000 |
| OTU_896 | **0.001** | **< 0.001** | **< 0.001** | **< 0.001** | **< 0.001** | 1.000 |  | **0.001** | **< 0.001** | **< 0.001** | 0.323 | 0.192 | 1.000 |  | **< 0.001** | **< 0.001** | **< 0.001** | **< 0.001** | **< 0.001** | 1.000 |
| OTU_897 | 1.000 | **0.009** | 1.000 | 0.055 | 1.000 | 1.000 |  | 1.000 | **< 0.001** | **0.009** | **< 0.001** | **0.011** | 1.000 |  | 1.000 | **0.026** | 0.632 | 1.000 | 1.000 | 1.000 |
| OTU_898 | 1.000 | 1.000 | 0.145 | 0.094 | **< 0.001** | 1.000 |  | 1.000 | 1.000 | 1.000 | 0.556 | **0.002** | 1.000 |  | 1.000 | 0.160 | 0.405 | **0.028** | 0.060 | 1.000 |
| OTU_899 | 1.000 | 1.000 | 1.000 | 1.000 | 1.000 | 1.000 |  | 1.000 | 1.000 | 1.000 | 1.000 | 1.000 | 1.000 |  | 1.000 | 1.000 | 1.000 | 1.000 | 0.350 | 1.000 |
| OTU_9 | **0.001** | **< 0.001** | 1.000 | 0.219 | 0.379 | **< 0.001** |  | **0.001** | **< 0.001** | **< 0.001** | **< 0.001** | 1.000 | **< 0.001** |  | 0.154 | **< 0.001** | 1.000 | **< 0.001** | 1.000 | **< 0.001** |
| OTU_90 | 0.334 | **< 0.001** | **< 0.001** | **< 0.001** | **< 0.001** | 1.000 |  | 0.334 | **< 0.001** | **< 0.001** | **< 0.001** | **< 0.001** | 1.000 |  | 1.000 | **< 0.001** | **< 0.001** | **< 0.001** | 0.124 | 1.000 |
| OTU_900 | 1.000 | 0.316 | 1.000 | 1.000 | 1.000 | 1.000 |  | 1.000 | **< 0.001** | **< 0.001** | 0.913 | 0.459 | 1.000 |  | 1.000 | 1.000 | 1.000 | 1.000 | 1.000 | 1.000 |
| OTU_901 | 1.000 | 0.589 | 1.000 | 0.478 | 1.000 | 0.645 |  | 1.000 | 0.218 | 1.000 | 0.328 | 1.000 | 0.231 |  | 1.000 | 1.000 | 0.355 | 1.000 | 1.000 | 0.708 |
| OTU_902 | **0.038** | 0.797 | 1.000 | 1.000 | **< 0.001** | **0.050** |  | **0.038** | 0.243 | **< 0.001** | 0.340 | **< 0.001** | 0.086 |  | 1.000 | 1.000 | 0.995 | 1.000 | 0.217 | 0.087 |
| OTU_903 | 1.000 | 0.205 | **0.021** | 0.437 | **0.038** | 1.000 |  | 1.000 | 0.341 | 0.076 | **0.020** | **0.003** | 1.000 |  | 0.666 | 1.000 | 1.000 | 1.000 | 1.000 | 1.000 |
| OTU_904 | 1.000 | **0.020** | **< 0.001** | 0.628 | **0.015** | 1.000 |  | 1.000 | 0.436 | **0.037** | 1.000 | 0.393 | 1.000 |  | 1.000 | 0.152 | **0.001** | 1.000 | 0.137 | 1.000 |
| OTU_905 | 1.000 | 1.000 | 1.000 | 1.000 | 1.000 | 1.000 |  | 1.000 | 1.000 | 1.000 | 1.000 | 1.000 | 1.000 |  | 1.000 | 0.111 | 1.000 | 1.000 | 1.000 | 1.000 |
| OTU_907 | 0.138 | **0.013** | 1.000 | 1.000 | 1.000 | 0.737 |  | 0.138 | **0.003** | 0.484 | 1.000 | 0.728 | 1.000 |  | 0.805 | 0.427 | 1.000 | 1.000 | 1.000 | 1.000 |
| OTU_908 | 0.646 | 0.579 | 0.385 | 1.000 | 1.000 | 1.000 |  | 0.646 | **0.006** | 1.000 | 1.000 | 1.000 | 0.058 |  | 1.000 | 0.099 | 1.000 | 1.000 | 1.000 | 1.000 |
| OTU_909 | 1.000 | 0.244 | 1.000 | 1.000 | 1.000 | 1.000 |  | 1.000 | 1.000 | 1.000 | 1.000 | 1.000 | 1.000 |  | 1.000 | 1.000 | 1.000 | 1.000 | 1.000 | 1.000 |
| OTU_91 | **0.049** | **< 0.001** | 1.000 | 1.000 | 0.717 | **0.023** |  | **0.049** | **< 0.001** | 0.395 | 0.430 | 1.000 | **< 0.001** |  | 1.000 | **< 0.001** | 1.000 | **0.039** | 1.000 | **< 0.001** |
| OTU_910 | 1.000 | 0.228 | 0.238 | 1.000 | 1.000 | 1.000 |  | 1.000 | 1.000 | 1.000 | 1.000 | 1.000 | 1.000 |  | 1.000 | 1.000 | 1.000 | 1.000 | 1.000 | 1.000 |
| OTU_911 | 0.868 | 1.000 | 1.000 | 1.000 | 1.000 | 1.000 |  | 0.868 | 0.053 | 1.000 | 0.062 | 1.000 | 1.000 |  | 1.000 | **< 0.001** | **< 0.001** | 0.570 | **0.032** | 1.000 |
| OTU_912 | 1.000 | 0.152 | 0.134 | 0.131 | 0.088 | 1.000 |  | 1.000 | 0.180 | 1.000 | 1.000 | 1.000 | 1.000 |  | 1.000 | 1.000 | 0.586 | 0.157 | 0.062 | 1.000 |
| OTU_913 | 1.000 | 1.000 | 1.000 | 0.628 | 1.000 | 1.000 |  | 1.000 | 1.000 | 1.000 | 0.466 | 1.000 | 1.000 |  | 1.000 | 1.000 | 1.000 | 1.000 | 1.000 | 1.000 |
| OTU_914 | 1.000 | 0.311 | 1.000 | 1.000 | 1.000 | 1.000 |  | 1.000 | 1.000 | 1.000 | 1.000 | 1.000 | 1.000 |  | 1.000 | **0.009** | 1.000 | 0.140 | 1.000 | 0.084 |
| OTU_916 | 0.801 | **0.024** | 1.000 | 1.000 | 1.000 | 1.000 |  | 0.801 | **0.028** | 1.000 | 0.796 | 1.000 | 0.521 |  | 1.000 | 1.000 | 1.000 | 1.000 | 1.000 | 1.000 |
| OTU_917 | 1.000 | 1.000 | **0.038** | 1.000 | 1.000 | 1.000 |  | 1.000 | 1.000 | 0.578 | 1.000 | 1.000 | 1.000 |  | 1.000 | 1.000 | 0.314 | 1.000 | 1.000 | 1.000 |
| OTU_918 | 1.000 | 1.000 | **0.009** | 1.000 | 0.130 | 1.000 |  | 1.000 | 1.000 | **0.020** | 1.000 | 1.000 | 0.714 |  | 1.000 | 1.000 | 1.000 | 1.000 | 1.000 | 1.000 |
| OTU_92 | 1.000 | **0.043** | **< 0.001** | 0.058 | **< 0.001** | 0.065 |  | 1.000 | **< 0.001** | **< 0.001** | **< 0.001** | **< 0.001** | 1.000 |  | 0.210 | **< 0.001** | **< 0.001** | 0.759 | **0.003** | 1.000 |
| OTU_920 | 1.000 | 1.000 | 1.000 | 1.000 | 1.000 | 1.000 |  | 1.000 | 0.971 | 1.000 | 1.000 | 1.000 | 1.000 |  | 1.000 | 1.000 | 1.000 | 1.000 | 0.107 | 0.290 |
| OTU_921 | 0.508 | 1.000 | 1.000 | 1.000 | 1.000 | 1.000 |  | 0.508 | 1.000 | 1.000 | 1.000 | 1.000 | 1.000 |  | 1.000 | 1.000 | 1.000 | 1.000 | 1.000 | 1.000 |
| OTU_922 | 1.000 | 1.000 | 0.089 | 1.000 | **0.024** | 0.657 |  | 1.000 | 1.000 | 0.199 | 1.000 | 0.054 | 1.000 |  | 1.000 | 1.000 | **0.038** | 1.000 | 1.000 | 1.000 |
| OTU_924 | 0.344 | 0.345 | 0.141 | 1.000 | 1.000 | 1.000 |  | 0.344 | 1.000 | 1.000 | 1.000 | 1.000 | 1.000 |  | 1.000 | 1.000 | 1.000 | 1.000 | 1.000 | 1.000 |
| OTU_925 | 1.000 | 1.000 | 1.000 | 1.000 | 0.153 | 1.000 |  | 1.000 | 1.000 | 1.000 | 1.000 | 0.509 | 1.000 |  | 1.000 | 1.000 | 1.000 | 1.000 | 1.000 | 1.000 |
| OTU_926 | 1.000 | 1.000 | 1.000 | 1.000 | 0.591 | 1.000 |  | 1.000 | 1.000 | 0.141 | 1.000 | 1.000 | 1.000 |  | 1.000 | 1.000 | 1.000 | 1.000 | 1.000 | 1.000 |
| OTU_927 | 1.000 | 1.000 | 1.000 | 1.000 | 1.000 | 1.000 |  | 1.000 | 1.000 | 0.128 | 1.000 | 1.000 | 1.000 |  | 1.000 | 1.000 | 1.000 | 1.000 | 1.000 | 1.000 |
| OTU_928 | 0.469 | **< 0.001** | **< 0.001** | 1.000 | 0.723 | 1.000 |  | 0.469 | **0.013** | 0.170 | 1.000 | 1.000 | 1.000 |  | 1.000 | 0.174 | 1.000 | 1.000 | 1.000 | 1.000 |
| OTU_929 | 1.000 | 1.000 | 0.215 | 1.000 | 0.130 | 0.282 |  | 1.000 | 1.000 | 1.000 | 1.000 | 1.000 | 1.000 |  | 0.195 | **0.025** | **0.001** | 1.000 | 1.000 | 1.000 |
| OTU_93 | 1.000 | 1.000 | **< 0.001** | 1.000 | **< 0.001** | **< 0.001** |  | 1.000 | 1.000 | **< 0.001** | 1.000 | **< 0.001** | **< 0.001** |  | 1.000 | 1.000 | **< 0.001** | 1.000 | **< 0.001** | **< 0.001** |
| OTU_930 | 1.000 | 1.000 | 1.000 | 1.000 | 1.000 | 1.000 |  | 1.000 | 1.000 | 1.000 | 1.000 | 1.000 | 1.000 |  | 1.000 | 1.000 | 1.000 | 1.000 | 1.000 | 1.000 |
| OTU_931 | 1.000 | 1.000 | 1.000 | 1.000 | 0.083 | 0.379 |  | 1.000 | 1.000 | 1.000 | 1.000 | 1.000 | 1.000 |  | 1.000 | 1.000 | **0.015** | 1.000 | 0.388 | 0.760 |
| OTU_932 | 1.000 | 1.000 | **0.005** | 1.000 | 0.246 | 0.700 |  | 1.000 | 1.000 | 1.000 | 1.000 | 1.000 | 0.481 |  | 0.379 | 1.000 | 0.079 | 1.000 | 1.000 | 1.000 |
| OTU_933 | 1.000 | 0.811 | 1.000 | 1.000 | 1.000 | 1.000 |  | 1.000 | **< 0.001** | **0.003** | 1.000 | 1.000 | 1.000 |  | 1.000 | 0.974 | 1.000 | 1.000 | 1.000 | 1.000 |
| OTU_934 | 1.000 | 1.000 | 1.000 | 0.608 | 1.000 | 0.124 |  | 1.000 | 1.000 | 0.366 | 1.000 | 1.000 | 0.938 |  | 1.000 | 1.000 | 1.000 | 1.000 | 1.000 | 1.000 |
| OTU_935 | 1.000 | 1.000 | **0.008** | 1.000 | **0.006** | 0.185 |  | 1.000 | 1.000 | 1.000 | 1.000 | 1.000 | 1.000 |  | 1.000 | 1.000 | 0.295 | 1.000 | 1.000 | 1.000 |
| OTU_936 | 1.000 | 1.000 | 1.000 | 1.000 | 1.000 | 1.000 |  | 1.000 | 1.000 | **0.033** | 0.096 | 1.000 | 0.071 |  | 1.000 | 1.000 | 0.979 | 1.000 | 0.266 | 1.000 |
| OTU_938 | 1.000 | 1.000 | 1.000 | 1.000 | 0.198 | 1.000 |  | 1.000 | 1.000 | **0.044** | **0.007** | **< 0.001** | 1.000 |  | 1.000 | 1.000 | 1.000 | 1.000 | 1.000 | 1.000 |
| OTU_939 | 1.000 | 1.000 | **0.029** | 1.000 | **< 0.001** | **0.029** |  | 1.000 | 1.000 | **0.022** | 1.000 | 0.158 | **0.021** |  | 1.000 | 1.000 | **< 0.001** | 1.000 | **0.002** | **0.003** |
| OTU_94 | **0.001** | **< 0.001** | **< 0.001** | **0.005** | 1.000 | **0.019** |  | **0.001** | **< 0.001** | **< 0.001** | 0.127 | 1.000 | 0.313 |  | **0.011** | **< 0.001** | **< 0.001** | **0.003** | 1.000 | **0.032** |
| OTU_940 | 0.639 | 1.000 | 1.000 | 0.895 | 0.070 | 1.000 |  | 0.639 | 1.000 | 1.000 | 1.000 | 1.000 | 1.000 |  | 1.000 | 1.000 | 1.000 | 1.000 | 1.000 | 1.000 |
| OTU_941 | **< 0.001** | 1.000 | 1.000 | **0.014** | **0.001** | 1.000 |  | **< 0.001** | 1.000 | 1.000 | 1.000 | 1.000 | 1.000 |  | 1.000 | 0.188 | 1.000 | 1.000 | 1.000 | 1.000 |
| OTU_942 | 1.000 | 0.908 | 0.141 | 1.000 | 1.000 | 1.000 |  | 1.000 | 1.000 | 1.000 | 0.709 | 1.000 | 1.000 |  | 1.000 | **0.011** | 0.737 | 0.462 | 1.000 | 1.000 |
| OTU_943 | **0.001** | **< 0.001** | **0.005** | 1.000 | 1.000 | 1.000 |  | **0.001** | **< 0.001** | **< 0.001** | 0.150 | 1.000 | 1.000 |  | 1.000 | 0.116 | 1.000 | 1.000 | 1.000 | 1.000 |
| OTU_944 | 1.000 | 0.128 | 0.054 | 1.000 | 1.000 | 1.000 |  | 1.000 | 0.158 | 0.999 | 0.451 | 1.000 | 1.000 |  | 1.000 | 1.000 | 1.000 | 1.000 | 1.000 | 1.000 |
| OTU_945 | 1.000 | 1.000 | 1.000 | 1.000 | 1.000 | 0.295 |  | 1.000 | 0.317 | **0.002** | 0.441 | **0.003** | 1.000 |  | 1.000 | 1.000 | 1.000 | 1.000 | 1.000 | 0.748 |
| OTU_946 | 1.000 | 1.000 | 1.000 | 1.000 | 1.000 | 1.000 |  | 1.000 | 1.000 | 1.000 | 1.000 | 1.000 | 1.000 |  | 1.000 | 1.000 | 1.000 | 1.000 | 1.000 | 1.000 |
| OTU_947 | 1.000 | 1.000 | 1.000 | 1.000 | 1.000 | 1.000 |  | 1.000 | 0.394 | 1.000 | 1.000 | 1.000 | 1.000 |  | 1.000 | 1.000 | 1.000 | 1.000 | 1.000 | 1.000 |
| OTU_948 | 1.000 | 1.000 | 1.000 | 1.000 | 1.000 | 0.221 |  | 1.000 | 1.000 | **0.019** | 1.000 | **0.024** | 0.344 |  | 1.000 | 1.000 | 0.988 | 1.000 | 1.000 | 1.000 |
| OTU_949 | 1.000 | 1.000 | 1.000 | 1.000 | 0.438 | 1.000 |  | 1.000 | 1.000 | 1.000 | 1.000 | 1.000 | 1.000 |  | 1.000 | **0.025** | 1.000 | 0.161 | 1.000 | 0.334 |
| OTU_95 | **0.009** | 1.000 | **< 0.001** | **< 0.001** | **< 0.001** | 0.665 |  | **0.009** | 0.399 | **< 0.001** | **< 0.001** | **< 0.001** | 0.284 |  | 1.000 | **0.007** | **< 0.001** | **< 0.001** | **< 0.001** | 1.000 |
| OTU_950 | 0.079 | 1.000 | 1.000 | 1.000 | **0.006** | 1.000 |  | 0.079 | 1.000 | 0.186 | 1.000 | 0.218 | 1.000 |  | 1.000 | 1.000 | 1.000 | 1.000 | 1.000 | 1.000 |
| OTU_951 | 1.000 | 1.000 | 1.000 | 1.000 | 1.000 | 1.000 |  | 1.000 | 1.000 | 1.000 | 1.000 | 1.000 | 1.000 |  | 1.000 | 1.000 | 1.000 | 1.000 | 1.000 | 1.000 |
| OTU_952 | 1.000 | 0.919 | 0.106 | 1.000 | 0.262 | 1.000 |  | 1.000 | 0.145 | 0.148 | 1.000 | 1.000 | 1.000 |  | 1.000 | 1.000 | 1.000 | 0.209 | 1.000 | 1.000 |
| OTU_953 | **0.001** | **< 0.001** | 0.104 | 1.000 | 0.962 | 1.000 |  | **0.001** | **0.029** | 0.756 | 1.000 | 1.000 | 1.000 |  | 1.000 | 0.133 | 0.132 | 1.000 | 1.000 | 1.000 |
| OTU_955 | 1.000 | 1.000 | 0.197 | 1.000 | 0.739 | 1.000 |  | 1.000 | 0.067 | **0.010** | 0.724 | 0.123 | 1.000 |  | 1.000 | 1.000 | 1.000 | 1.000 | 1.000 | 1.000 |
| OTU_956 | 0.622 | 0.217 | 0.149 | 1.000 | 1.000 | 1.000 |  | 0.622 | 1.000 | 0.441 | 1.000 | 0.525 | 1.000 |  | 1.000 | 0.842 | 1.000 | 1.000 | 1.000 | 1.000 |
| OTU_957 | 1.000 | 1.000 | 1.000 | 1.000 | 1.000 | 1.000 |  | 1.000 | 1.000 | 1.000 | 1.000 | **0.040** | 1.000 |  | 1.000 | 1.000 | 1.000 | 1.000 | 1.000 | 1.000 |
| OTU_958 | 1.000 | 1.000 | 1.000 | 1.000 | 1.000 | 1.000 |  | 1.000 | 0.140 | 1.000 | 1.000 | 0.955 | 1.000 |  | 1.000 | 1.000 | 1.000 | 1.000 | 0.077 | 1.000 |
| OTU_959 | 0.901 | 0.617 | 1.000 | 1.000 | 1.000 | 1.000 |  | 0.901 | 0.073 | 1.000 | 0.605 | 1.000 | 1.000 |  | 1.000 | 1.000 | 1.000 | 1.000 | 1.000 | 1.000 |
| OTU_96 | 1.000 | **< 0.001** | **< 0.001** | **0.006** | **< 0.001** | 1.000 |  | 1.000 | **0.006** | **< 0.001** | 0.473 | **< 0.001** | 0.879 |  | 1.000 | **< 0.001** | **< 0.001** | 0.428 | **0.012** | 1.000 |
| OTU_960 | 1.000 | 1.000 | 1.000 | 1.000 | 1.000 | 1.000 |  | 1.000 | 0.640 | 0.609 | 1.000 | 1.000 | 1.000 |  | 1.000 | 1.000 | 1.000 | 1.000 | 1.000 | 1.000 |
| OTU_961 | 1.000 | **0.018** | 0.599 | 1.000 | 1.000 | 1.000 |  | 1.000 | 0.090 | 0.128 | 1.000 | 1.000 | 1.000 |  | 1.000 | 0.876 | 1.000 | 1.000 | 1.000 | 1.000 |
| OTU_962 | 1.000 | 0.236 | 0.202 | 0.576 | 0.395 | 1.000 |  | 1.000 | 1.000 | 0.643 | 1.000 | 1.000 | 1.000 |  | 1.000 | 1.000 | 1.000 | 1.000 | 1.000 | 1.000 |
| OTU_963 | 1.000 | 1.000 | 1.000 | 1.000 | 0.661 | 1.000 |  | 1.000 | **0.005** | 1.000 | 0.068 | 1.000 | **0.003** |  | 1.000 | 1.000 | 0.998 | 1.000 | 1.000 | 1.000 |
| OTU_964 | 1.000 | 1.000 | 1.000 | 1.000 | 1.000 | 1.000 |  | 1.000 | 1.000 | 1.000 | 1.000 | 1.000 | 1.000 |  | 1.000 | 1.000 | 1.000 | 1.000 | 1.000 | 1.000 |
| OTU_965 | 1.000 | 1.000 | 1.000 | 1.000 | 1.000 | 1.000 |  | 1.000 | 1.000 | 1.000 | 0.869 | 1.000 | 1.000 |  | 1.000 | 0.445 | 1.000 | 1.000 | 1.000 | 0.662 |
| OTU_966 | 1.000 | 0.930 | **0.025** | 1.000 | **0.034** | 1.000 |  | 1.000 | 1.000 | 1.000 | 1.000 | 1.000 | 1.000 |  | 1.000 | 1.000 | 0.176 | 1.000 | 0.627 | 1.000 |
| OTU_967 | 1.000 | 1.000 | 1.000 | 1.000 | 1.000 | 1.000 |  | 1.000 | 1.000 | 1.000 | 1.000 | 1.000 | 1.000 |  | 1.000 | 1.000 | 1.000 | 1.000 | 1.000 | 1.000 |
| OTU_968 | 1.000 | 0.477 | **< 0.001** | 1.000 | **0.005** | 0.572 |  | 1.000 | 1.000 | **0.001** | 1.000 | **0.001** | **0.030** |  | 1.000 | 1.000 | 0.222 | 1.000 | 0.219 | 0.478 |
| OTU_97 | **< 0.001** | **< 0.001** | 1.000 | 1.000 | **< 0.001** | **< 0.001** |  | **< 0.001** | **0.018** | 1.000 | 1.000 | **< 0.001** | **< 0.001** |  | 1.000 | 0.681 | **< 0.001** | 1.000 | **< 0.001** | **< 0.001** |
| OTU_971 | 1.000 | 1.000 | 1.000 | 1.000 | 1.000 | 1.000 |  | 1.000 | 1.000 | 1.000 | 1.000 | 0.458 | 1.000 |  | 1.000 | 1.000 | 1.000 | 1.000 | 1.000 | 1.000 |
| OTU_972 | 1.000 | 1.000 | 1.000 | 1.000 | 1.000 | 1.000 |  | 1.000 | 1.000 | 0.182 | 1.000 | **0.001** | 0.554 |  | 1.000 | 1.000 | 1.000 | 1.000 | 1.000 | 0.334 |
| OTU_973 | 1.000 | **0.005** | 0.162 | **0.004** | 0.103 | 1.000 |  | 1.000 | **< 0.001** | 0.994 | **< 0.001** | 1.000 | 0.080 |  | 1.000 | 1.000 | 1.000 | 1.000 | 1.000 | 1.000 |
| OTU_974 | 1.000 | 1.000 | 1.000 | 1.000 | 1.000 | 1.000 |  | 1.000 | 1.000 | 1.000 | 1.000 | 1.000 | 1.000 |  | 1.000 | 1.000 | 1.000 | 1.000 | 1.000 | 1.000 |
| OTU_975 | 1.000 | 0.127 | 1.000 | 1.000 | 1.000 | 1.000 |  | 1.000 | 1.000 | 1.000 | 1.000 | 1.000 | 1.000 |  | 1.000 | 0.192 | 0.101 | 1.000 | 0.796 | 1.000 |
| OTU_977 | 1.000 | 1.000 | 1.000 | 1.000 | 1.000 | 1.000 |  | 1.000 | 1.000 | 1.000 | 1.000 | 1.000 | 1.000 |  | 1.000 | 1.000 | 1.000 | 1.000 | 1.000 | 1.000 |
| OTU_978 | 1.000 | 1.000 | 1.000 | 1.000 | 1.000 | 1.000 |  | 1.000 | 0.115 | **0.002** | 1.000 | 0.256 | 1.000 |  | 1.000 | 0.517 | 0.145 | 1.000 | 1.000 | 1.000 |
| OTU_979 | 1.000 | 1.000 | 1.000 | 1.000 | 1.000 | 1.000 |  | 1.000 | 0.269 | 0.236 | 1.000 | 1.000 | 1.000 |  | 1.000 | 1.000 | 1.000 | 0.833 | 0.098 | 1.000 |
| OTU_98 | **< 0.001** | **< 0.001** | **< 0.001** | **0.003** | 0.129 | **< 0.001** |  | **< 0.001** | **< 0.001** | 1.000 | **0.045** | **< 0.001** | **< 0.001** |  | **< 0.001** | **< 0.001** | **< 0.001** | **< 0.001** | 1.000 | **< 0.001** |
| OTU_980 | 1.000 | 1.000 | 1.000 | 1.000 | 1.000 | 1.000 |  | 1.000 | 1.000 | 1.000 | 1.000 | 0.783 | 1.000 |  | 1.000 | 0.618 | 1.000 | 1.000 | 1.000 | 1.000 |
| OTU_981 | 1.000 | 1.000 | 1.000 | 1.000 | 1.000 | 1.000 |  | 1.000 | 1.000 | 1.000 | 1.000 | 1.000 | 1.000 |  | **0.004** | **< 0.001** | **< 0.001** | 1.000 | 0.607 | 1.000 |
| OTU_983 | 1.000 | **< 0.001** | **0.012** | **< 0.001** | 1.000 | 0.147 |  | 1.000 | **< 0.001** | **0.004** | **< 0.001** | 0.903 | 0.249 |  | 1.000 | **0.010** | 0.717 | 1.000 | 1.000 | 1.000 |
| OTU_984 | 0.070 | 1.000 | 1.000 | 1.000 | 1.000 | 1.000 |  | 0.070 | 1.000 | 1.000 | 1.000 | 1.000 | 1.000 |  | 1.000 | 1.000 | 1.000 | 1.000 | 0.303 | 1.000 |
| OTU_985 | 1.000 | 1.000 | 1.000 | 1.000 | 1.000 | 1.000 |  | 1.000 | **0.004** | **0.006** | **0.006** | **0.008** | 1.000 |  | 1.000 | 1.000 | 1.000 | 1.000 | 1.000 | 1.000 |
| OTU_987 | 0.687 | **0.001** | 0.710 | 1.000 | 1.000 | 1.000 |  | 0.687 | **< 0.001** | **0.013** | 1.000 | 1.000 | 1.000 |  | 0.369 | **< 0.001** | **0.018** | 0.189 | 1.000 | 1.000 |
| OTU_988 | 1.000 | 1.000 | 0.236 | 1.000 | 0.697 | 1.000 |  | 1.000 | 0.498 | 1.000 | 1.000 | 1.000 | 1.000 |  | 1.000 | 1.000 | 1.000 | 1.000 | 1.000 | 1.000 |
| OTU_989 | 1.000 | 1.000 | 1.000 | 1.000 | 1.000 | 1.000 |  | 1.000 | 1.000 | 1.000 | 1.000 | 1.000 | 1.000 |  | 1.000 | 1.000 | 1.000 | 1.000 | 1.000 | 1.000 |
| OTU_99 | **< 0.001** | **< 0.001** | **0.004** | 0.292 | **< 0.001** | 0.901 |  | **< 0.001** | **< 0.001** | 1.000 | 1.000 | **< 0.001** | **< 0.001** |  | 0.056 | **0.029** | 1.000 | 1.000 | **0.048** | 0.103 |
| OTU_991 | 1.000 | 1.000 | 0.141 | 1.000 | 1.000 | 1.000 |  | 1.000 | 0.179 | **0.032** | 0.783 | 0.148 | 1.000 |  | 1.000 | **0.044** | **0.005** | 1.000 | 1.000 | 1.000 |
| OTU_992 | 0.068 | **0.005** | 1.000 | 1.000 | 0.181 | **0.049** |  | 0.068 | **< 0.001** | 1.000 | 1.000 | **0.023** | **< 0.001** |  | **0.002** | **< 0.001** | 1.000 | 1.000 | **0.004** | **0.002** |
| OTU_993 | 1.000 | 1.000 | 1.000 | 1.000 | 1.000 | 1.000 |  | 1.000 | 1.000 | 1.000 | 1.000 | 0.294 | 1.000 |  | 1.000 | 1.000 | 1.000 | 1.000 | 0.257 | 1.000 |
| OTU_994 | 1.000 | 1.000 | **0.004** | 1.000 | 0.080 | **0.006** |  | 1.000 | 1.000 | 0.395 | 1.000 | 1.000 | 1.000 |  | 1.000 | 1.000 | **0.017** | 1.000 | **0.008** | **< 0.001** |
| OTU_996 | 1.000 | 1.000 | **0.004** | 1.000 | **0.003** | **0.002** |  | 1.000 | 1.000 | 1.000 | 0.178 | **0.009** | 1.000 |  | 1.000 | 1.000 | **0.022** | 1.000 | 0.201 | 0.055 |
| OTU_997 | 0.556 | 1.000 | 1.000 | 1.000 | 1.000 | 1.000 |  | 0.556 | 1.000 | 1.000 | 1.000 | 1.000 | 1.000 |  | 1.000 | 1.000 | 1.000 | 1.000 | 1.000 | 1.000 |
| OTU_998 | 0.195 | 1.000 | 1.000 | 1.000 | 0.160 | 1.000 |  | 0.195 | **0.008** | **0.002** | 1.000 | 1.000 | 1.000 |  | 1.000 | 1.000 | 1.000 | 1.000 | 1.000 | 1.000 |
| OTU_999 | 1.000 | **0.049** | **0.002** | 0.292 | **0.012** | 1.000 |  | 1.000 | 0.906 | 0.418 | **0.037** | **0.008** | 1.000 |  | 1.000 | 1.000 | 0.217 | 0.516 | **0.022** | 1.000 |

Significant values are given in bold.
