# Supplementary material for: Bacterial Preferences for Specific Soil Particle Size Fractions Revealed by Community Analyses
Source: Front Microbiol. 2018 Feb 23;9:149. doi: 10.3389/fmicb.2018.00149 (PMC5829042; doi:10.3389/fmicb.2018.00149)
Supplement: Supplementary file 8 [file Table8.DOCX]

Table S8 Significance values for phylum level of bacteria after Bonferroni-correction to account for multiple pair-wise comparisons between sand/POM, coarse silt, fine silt, and clay for all three replicates, i.e. UNF, NPK, and AM

| **Phylum** | **UNF** | | | | | |  | **NPK** | | | | | |  | **AM** | | | | | |
| --- | --- | --- | --- | --- | --- | --- | --- | --- | --- | --- | --- | --- | --- | --- | --- | --- | --- | --- | --- | --- |
|  | **Sand/POM - Coarse silt** | **Sand/POM - Fine silt** | **Sand/POM - Clay** | **Coarse silt - Fine silt** | **Coarse silt - Clay** | **Fine silt - Clay** |  | **Sand/POM - Coarse silt** | **Sand/POM - Fine silt** | **Sand/POM - Clay** | **Coarse silt - Fine silt** | **Coarse silt - Clay** | **Fine silt - Clay** |  | **Sand/POM - Coarse silt** | **Sand/POM - Fine silt** | **Sand/POM - Clay** | **Coarse silt - Fine silt** | **Coarse silt - Clay** | **Fine silt - Clay** |
| Acidobacteria | **0.031** | **0.031** | **0.003** | 0.697 | 1.000 | 1.000 |  | **< 0.001** | **< 0.001** | **< 0.001** | 0.138 | 0.556 | 1.000 |  | 1.000 | **0.006** | 1.000 | 0.112 | 1.000 | 0.409 |
| Actinobacteria | 1.000 | 1.000 | 1.000 | **< 0.001** | 1.000 | **< 0.001** |  | 1.000 | **< 0.001** | 1.000 | **0.004** | 1.000 | **0.009** |  | 1.000 | **< 0.001** | 1.000 | **0.006** | 1.000 | **0.007** |
| Armatimonadetes | **< 0.001** | **< 0.001** | 1.000 | 0.175 | **< 0.001** | 1.000 |  | **0.003** | 1.000 | 1.000 | 1.000 | **0.002** | 1.000 |  | 1.000 | 1.000 | **0.022** | 1.000 | **0.038** | 1.000 |
| Bacteroidetes | **< 0.001** | **< 0.001** | 0.227 | 1.000 | 0.351 | **< 0.001** |  | 0.492 | **< 0.001** | 1.000 | 0.120 | 1.000 | **0.012** |  | 0.053 | **< 0.001** | 0.081 | **0.037** | 1.000 | **0.012** |
| Candidate division  WPS-1 | 1.000 | 1.000 | 1.000 | 1.000 | 1.000 | 1.000 |  | 1.000 | 1.000 | 1.000 | 1.000 | 1.000 | 1.000 |  | 1.000 | 1.000 | 1.000 | 1.000 | 1.000 | 1.000 |
| Candidate division  WPS-2 | 1.000 | 1.000 | 1.000 | 1.000 | 0.152 | 1.000 |  | 1.000 | **< 0.001** | 1.000 | 0.578 | 1.000 | 0.082 |  | 1.000 | 1.000 | **0.041** | 1.000 | 0.351 | 0.126 |
| Chloroflexi | 1.000 | 1.000 | 0.772 | 1.000 | 1.000 | 1.000 |  | 1.000 | **0.006** | 0.918 | **0.025** | 1.000 | 1.000 |  | 1.000 | 1.000 | 1.000 | 1.000 | 1.000 | 1.000 |
| Cyanobacteria | **0.028** | **0.028** | 1.000 | 0.435 | 0.617 | **< 0.001** |  | **< 0.001** | **< 0.001** | **< 0.001** | **< 0.001** | 1.000 | **0.011** |  | **< 0.001** | **< 0.001** | **< 0.001** | **< 0.001** | 1.000 | **< 0.001** |
| Firmicutes | **< 0.001** | **< 0.001** | **< 0.001** | 0.435 | 1.000 | 1.000 |  | **< 0.001** | **< 0.001** | **< 0.001** | 1.000 | 0.705 | 1.000 |  | **< 0.001** | **< 0.001** | **< 0.001** | 1.000 | 1.000 | 1.000 |
| Gemmatimonadetes | **< 0.001** | **< 0.001** | **< 0.001** | **< 0.001** | **< 0.001** | **0.001** |  | **0.001** | 1.000 | **< 0.001** | **0.025** | **< 0.001** | **< 0.001** |  | 1.000 | **< 0.001** | **< 0.001** | **0.006** | **< 0.001** | **0.016** |
| Latescibacteria | 1.000 | 1.000 | 1.000 | 1.000 | 1.000 | 1.000 |  | 1.000 | 1.000 | 1.000 | 1.000 | 1.000 | 1.000 |  | 1.000 | 0.332 | 1.000 | 1.000 | 1.000 | 1.000 |
| Nitrospirae | 0.441 | 0.441 | **< 0.001** | **0.010** | **0.005** | 1.000 |  | **0.006** | **< 0.001** | **< 0.001** | **< 0.001** | **0.002** | 1.000 |  | 0.053 | **< 0.001** | 1.000 | **0.008** | 1.000 | **< 0.001** |
| Planctomycetes | 1.000 | 1.000 | 1.000 | 0.697 | 1.000 | **0.006** |  | 1.000 | 1.000 | **< 0.001** | 1.000 | **0.007** | **0.003** |  | 1.000 | **< 0.001** | 1.000 | **0.009** | 1.000 | **< 0.001** |
| Proteobacteria | 1.000 | 1.000 | **< 0.001** | 1.000 | **0.004** | **0.003** |  | 1.000 | 1.000 | 1.000 | 1.000 | 1.000 | 1.000 |  | 0.212 | **0.001** | **< 0.001** | 1.000 | **0.022** | 0.379 |
| Spirochaetes | **0.006** | **0.006** | **< 0.001** | 1.000 | 1.000 | 1.000 |  | 0.070 | 0.117 | **< 0.001** | 1.000 | 1.000 | 1.000 |  | 1.000 | **< 0.001** | **< 0.001** | 0.223 | 0.119 | 1.000 |
| Verrucomicrobia | 1.000 | 1.000 | 1.000 | 1.000 | 1.000 | 1.000 |  | 1.000 | 1.000 | 0.447 | 1.000 | 1.000 | 1.000 |  | 1.000 | 1.000 | 1.000 | 1.000 | 1.000 | 1.000 |
| Unclassified bacteria | **0.031** | **0.031** | 0.313 | 1.000 | 1.000 | 0.993 |  | 0.092 | **< 0.001** | **< 0.001** | 0.138 | 0.537 | 1.000 |  | 1.000 | 0.665 | 1.000 | 1.000 | 1.000 | 0.409 |

Significant values are given in bold.
