# Supplementary material for: Bacterial Preferences for Specific Soil Particle Size Fractions Revealed by Community Analyses
Source: Front Microbiol. 2018 Feb 23;9:149. doi: 10.3389/fmicb.2018.00149 (PMC5829042; doi:10.3389/fmicb.2018.00149)
Supplement: Supplementary file 9 [file Table9.DOCX]

Table S9 Significance values for class level of bacteria after Bonferroni-correction to account for multiple pair-wise comparisons between sand/POM, coarse silt, fine silt, and clay for all three replicates, i.e. UNF, NPK, and AM

| **Class** | **UNF** | | | | | |  | **NPK** | | | | | |  | **AM** | | | | | |
| --- | --- | --- | --- | --- | --- | --- | --- | --- | --- | --- | --- | --- | --- | --- | --- | --- | --- | --- | --- | --- |
|  | **Sand/POM - Coarse silt** | **Sand/POM - Fine silt** | **Sand/POM - Clay** | **Coarse silt - Fine silt** | **Coarse silt - Clay** | **Fine silt - Clay** |  | **Sand/POM - Coarse silt** | **Sand/POM - Fine silt** | **Sand/POM - Clay** | **Coarse silt - Fine silt** | **Coarse silt - Clay** | **Fine silt - Clay** |  | **Sand/POM - Coarse silt** | **Sand/POM - Fine silt** | **Sand/POM - Clay** | **Coarse silt - Fine silt** | **Coarse silt - Clay** | **Fine silt - Clay** |
| Acidobacteria Gp1 | 1.000 | 0.136 | 1.000 | 1.000 | 1.000 | 1.000 |  | 0.180 | **0.003** | 1.000 | 1.000 | 1.000 | 0.609 |  | 1.000 | 0.331 | 1.000 | 1.000 | 1.000 | 0.578 |
| Acidobacteria Gp2 | 1.000 | 0.053 | **< 0.001** | 1.000 | 0.311 | 1.000 |  | 1.000 | **< 0.001** | 0.080 | 0.671 | 1.000 | 1.000 |  | 0.386 | 1.000 | 0.554 | **< 0.001** | **< 0.001** | 1.000 |
| Acidobacteria Gp3 | 1.000 | **< 0.001** | **< 0.001** | **< 0.001** | **< 0.001** | 1.000 |  | 1.000 | **< 0.001** | **< 0.001** | **< 0.001** | **< 0.001** | 0.094 |  | 1.000 | **< 0.001** | 0.071 | **< 0.001** | **< 0.001** | 0.108 |
| Acidobacteria Gp4 | 1.000 | 1.000 | **0.002** | 1.000 | **< 0.001** | **0.030** |  | 1.000 | 1.000 | **0.049** | 1.000 | **0.032** | 0.211 |  | 1.000 | 1.000 | 1.000 | 1.000 | 0.554 | 0.108 |
| Acidobacteria Gp5 | 1.000 | 0.269 | 0.216 | 0.846 | 0.767 | 1.000 |  | 1.000 | 1.000 | 0.841 | 1.000 | 1.000 | 1.000 |  | 1.000 | 1.000 | 1.000 | 0.753 | 1.000 | 1.000 |
| Acidobacteria Gp6 | 1.000 | 1.000 | 1.000 | 1.000 | 1.000 | 1.000 |  | 1.000 | 1.000 | 1.000 | 1.000 | 1.000 | 1.000 |  | 0.488 | 1.000 | 0.093 | 1.000 | 1.000 | 1.000 |
| Acidobacteria Gp7 | 0.444 | 1.000 | **0.019** | 0.180 | 1.000 | **0.010** |  | 0.238 | 0.534 | **0.001** | 1.000 | 1.000 | 1.000 |  | 1.000 | 1.000 | 0.606 | 1.000 | 1.000 | 0.780 |
| Acidobacteria Gp10 | 0.091 | **< 0.001** | 1.000 | **0.003** | 1.000 | **< 0.001** |  | 1.000 | **< 0.001** | 1.000 | **< 0.001** | 1.000 | **< 0.001** |  | 0.056 | **< 0.001** | 1.000 | **0.005** | 1.000 | **< 0.001** |
| Acidobacteria Gp11 | 1.000 | **< 0.001** | **< 0.001** | **< 0.001** | **< 0.001** | 1.000 |  | 1.000 | 0.485 | 0.135 | 1.000 | 1.000 | 1.000 |  | 1.000 | 0.071 | 0.160 | **0.010** | **0.015** | 1.000 |
| Acidobacteria Gp13 | 1.000 | **< 0.001** | **< 0.001** | **< 0.001** | **< 0.001** | 1.000 |  | 0.518 | **< 0.001** | **< 0.001** | 0.053 | 1.000 | 1.000 |  | 1.000 | **0.037** | **0.003** | 0.137 | **0.007** | 1.000 |
| Acidobacteria Gp16 | 0.108 | **< 0.001** | **< 0.001** | 0.806 | 0.743 | 1.000 |  | **0.034** | **< 0.001** | **< 0.001** | 1.000 | 1.000 | 1.000 |  | 1.000 | **0.006** | **0.008** | 0.250 | 0.316 | 1.000 |
| Acidobacteria Gp17 | 0.129 | 1.000 | **< 0.001** | **0.003** | **< 0.001** | **< 0.001** |  | 0.726 | 0.068 | **< 0.001** | **< 0.001** | **< 0.001** | **< 0.001** |  | 1.000 | **0.005** | **< 0.001** | **0.005** | **< 0.001** | 0.089 |
| Acidobacteria Gp22 | 1.000 | 1.000 | 1.000 | 1.000 | 0.195 | 0.472 |  | 1.000 | 1.000 | 1.000 | 1.000 | 1.000 | 0.156 |  | 1.000 | 1.000 | 1.000 | 1.000 | 1.000 | 1.000 |
| Acidobacteria Gp25 | 1.000 | 1.000 | **< 0.001** | 1.000 | **0.013** | **< 0.001** |  | 0.726 | 1.000 | **< 0.001** | 1.000 | 0.153 | **0.008** |  | 1.000 | 1.000 | 1.000 | 1.000 | 1.000 | 1.000 |
| Actinobacteria | 1.000 | 0.088 | 1.000 | 0.098 | 1.000 | **0.008** |  | 1.000 | 0.125 | 1.000 | 0.353 | 1.000 | **0.004** |  | 1.000 | 0.105 | 1.000 | 0.137 | 1.000 | 1.000 |
| Alphaproteobacteria | 1.000 | 1.000 | 1.000 | 1.000 | 1.000 | 1.000 |  | 1.000 | 1.000 | 1.000 | 1.000 | 1.000 | 1.000 |  | 1.000 | 1.000 | 1.000 | 1.000 | 1.000 | 1.000 |
| Anaerolineae | 1.000 | 1.000 | **< 0.001** | 1.000 | **0.002** | 0.293 |  | 1.000 | 1.000 | **< 0.001** | 0.939 | 0.377 | **< 0.001** |  | 1.000 | 0.270 | **0.005** | **0.028** | **< 0.001** | 1.000 |
| Armatimonadetes gp4 | 1.000 | 1.000 | **< 0.001** | 0.558 | **< 0.001** | **0.031** |  | 1.000 | 1.000 | 0.133 | 1.000 | **0.026** | 1.000 |  | 1.000 | **0.005** | **0.002** | 0.076 | **0.016** | 1.000 |
| Armatimonadetes gp5 | 1.000 | 1.000 | 1.000 | 1.000 | 1.000 | 1.000 |  | 1.000 | 0.466 | 1.000 | 1.000 | 1.000 | 0.576 |  | 1.000 | 1.000 | 0.150 | 1.000 | 0.657 | 1.000 |
| Armatimonadia | **< 0.001** | **< 0.001** | **< 0.001** | 1.000 | 1.000 | 1.000 |  | **< 0.001** | **< 0.001** | **< 0.001** | **0.007** | 0.878 | 1.000 |  | 0.152 | **0.001** | 1.000 | 1.000 | 1.000 | 0.511 |
| Bacilli | **< 0.001** | **< 0.001** | **< 0.001** | 1.000 | 1.000 | 1.000 |  | **< 0.001** | **< 0.001** | **< 0.001** | 1.000 | 1.000 | 1.000 |  | **0.024** | **< 0.001** | **< 0.001** | 1.000 | 0.967 | 1.000 |
| Bacteroidia | 0.318 | 0.108 | **0.005** | 1.000 | 1.000 | 1.000 |  | **0.015** | 0.485 | 0.792 | 1.000 | 0.977 | 1.000 |  | 0.664 | 0.148 | 1.000 | 1.000 | 1.000 | 1.000 |
| Betaproteobacteria | 1.000 | 0.292 | **< 0.001** | 1.000 | **0.001** | 0.634 |  | 1.000 | **0.005** | **< 0.001** | 1.000 | **0.003** | 0.632 |  | 0.056 | **0.001** | **< 0.001** | 1.000 | 1.000 | 1.000 |
| Caldilineae | 0.511 | **< 0.001** | **< 0.001** | 0.263 | 0.244 | 1.000 |  | **0.004** | 1.000 | 1.000 | **< 0.001** | **< 0.001** | 1.000 |  | 1.000 | **0.021** | **0.002** | 0.111 | **0.009** | 1.000 |
| Chthonomonadetes | 1.000 | 1.000 | 0.311 | 1.000 | 0.259 | 1.000 |  | 0.949 | **0.003** | **0.005** | 1.000 | 1.000 | 1.000 |  | 1.000 | 1.000 | 0.793 | 1.000 | 1.000 | 0.855 |
| Clostridia | 0.076 | **< 0.001** | **< 0.001** | 1.000 | 1.000 | 1.000 |  | **< 0.001** | **< 0.001** | **< 0.001** | 1.000 | 1.000 | 1.000 |  | **0.002** | 0.072 | 0.071 | 1.000 | 1.000 | 1.000 |
| Cytophagia | 1.000 | **< 0.001** | 1.000 | 0.240 | 0.743 | **< 0.001** |  | 1.000 | **< 0.001** | 1.000 | **< 0.001** | 1.000 | **0.001** |  | 1.000 | 0.188 | **0.041** | 1.000 | **< 0.001** | **< 0.001** |
| Deltaproteobacteria | 1.000 | 1.000 | **< 0.001** | 0.719 | **< 0.001** | 0.082 |  | 1.000 | **< 0.001** | **< 0.001** | **0.016** | **< 0.001** | **0.010** |  | 1.000 | **< 0.001** | **< 0.001** | **0.025** | **< 0.001** | 1.000 |
| Erysipelotrichia | 0.070 | **< 0.001** | **0.002** | 0.290 | 1.000 | 1.000 |  | **0.009** | 1.000 | 1.000 | **< 0.001** | **0.003** | 1.000 |  | 0.130 | 1.000 | 1.000 | 0.188 | 0.338 | 1.000 |
| Flavobacteriia | **< 0.001** | **< 0.001** | **< 0.001** | **0.002** | 1.000 | **< 0.001** |  | **< 0.001** | **< 0.001** | **0.009** | 0.126 | 1.000 | **0.001** |  | **< 0.001** | **< 0.001** | 1.000 | 1.000 | **< 0.001** | **< 0.001** |
| Gammaproteobacteria | 1.000 | **< 0.001** | **0.002** | **< 0.001** | 1.000 | **0.002** |  | 0.663 | **< 0.001** | **< 0.001** | **< 0.001** | **0.025** | 0.094 |  | 0.152 | **< 0.001** | **< 0.001** | **< 0.001** | 0.969 | **0.020** |
| Gemmatimonadetes | **0.015** | 1.000 | **0.007** | **< 0.001** | **< 0.001** | 0.459 |  | 0.829 | 1.000 | **< 0.001** | **0.009** | **< 0.001** | **< 0.001** |  | 1.000 | **< 0.001** | **< 0.001** | **0.025** | **< 0.001** | 1.000 |
| Holophagae | 1.000 | 0.216 | 1.000 | 0.324 | 1.000 | **0.007** |  | 1.000 | 1.000 | 1.000 | 1.000 | 1.000 | 0.609 |  | 1.000 | 0.332 | 0.554 | 1.000 | 0.094 | **< 0.001** |
| Ktedonobacteria | 1.000 | 0.052 | 0.654 | 0.093 | 0.823 | 1.000 |  | **0.001** | **< 0.001** | **< 0.001** | 1.000 | 1.000 | 1.000 |  | 1.000 | 1.000 | 1.000 | 1.000 | 1.000 | 1.000 |
| Negativicutes | 1.000 | 1.000 | 1.000 | 1.000 | 1.000 | 1.000 |  | 1.000 | **0.009** | **0.024** | 0.212 | 0.675 | 1.000 |  | 1.000 | 1.000 | 1.000 | 0.171 | 1.000 | 1.000 |
| Nitrospira | 1.000 | **< 0.001** | **< 0.001** | 0.263 | **0.005** | 1.000 |  | 0.726 | **< 0.001** | **< 0.001** | **0.044** | 1.000 | 1.000 |  | 1.000 | **< 0.001** | 1.000 | 0.083 | 1.000 | **0.022** |
| Opitutae | 0.061 | **< 0.001** | **< 0.001** | 0.290 | 0.743 | 1.000 |  | 0.810 | **< 0.001** | **< 0.001** | **0.001** | **0.012** | 1.000 |  | **< 0.001** | **< 0.001** | **< 0.001** | 0.524 | 1.000 | 1.000 |
| Planctomycetia | 1.000 | 0.096 | 1.000 | 0.366 | 1.000 | **< 0.001** |  | 1.000 | 1.000 | 1.000 | 1.000 | 1.000 | 0.411 |  | 0.386 | **< 0.001** | 1.000 | 0.069 | 0.607 | **< 0.001** |
| Spartobacteria | 0.091 | 0.292 | **0.002** | 1.000 | 1.000 | 1.000 |  | 0.726 | 1.000 | 0.135 | 1.000 | 1.000 | 1.000 |  | 0.122 | 1.000 | **0.039** | 1.000 | 1.000 | 1.000 |
| Sphingobacteriia | **0.016** | **< 0.001** | 1.000 | 1.000 | 0.249 | **0.002** |  | 1.000 | **< 0.001** | 0.209 | 0.523 | 1.000 | 1.000 |  | 1.000 | **0.009** | 1.000 | 0.587 | 1.000 | **0.046** |
| Spirochaetia | 0.061 | 0.119 | **0.004** | 1.000 | 1.000 | 1.000 |  | 0.163 | 0.085 | **< 0.001** | 1.000 | 1.000 | 1.000 |  | 1.000 | **0.006** | **0.007** | 0.537 | 0.554 | 1.000 |
| Verrucomicrobia Subdivision3 | 1.000 | 0.449 | 0.109 | 1.000 | 1.000 | 1.000 |  | 1.000 | 1.000 | 1.000 | 1.000 | 1.000 | 1.000 |  | 1.000 | 1.000 | 0.815 | 1.000 | 1.000 | 1.000 |
| Verrucomicrobiae | 1.000 | 0.543 | 1.000 | 1.000 | 0.227 | 0.073 |  | 0.064 | **0.048** | 1.000 | 1.000 | 0.240 | 0.351 |  | 1.000 | 1.000 | 1.000 | 1.000 | 0.554 | 0.697 |
| Unclassified classes | 1.000 | 1.000 | 1.000 | 1.000 | 1.000 | 1.000 |  | 0.726 | 1.000 | 0.080 | 1.000 | 1.000 | 1.000 |  | 0.152 | 0.360 | 0.554 | 1.000 | 1.000 | 1.000 |

Significant values are given in bold.
