# Supplementary material for: Bacterial Preferences for Specific Soil Particle Size Fractions Revealed by Community Analyses
Source: Front Microbiol. 2018 Feb 23;9:149. doi: 10.3389/fmicb.2018.00149 (PMC5829042; doi:10.3389/fmicb.2018.00149)
Supplement: Supplementary file 10 [file Table10.DOCX]

Table S10 Significance values for order level of bacteria after Bonferroni-correction to account for multiple pair-wise comparisons between sand/POM, coarse silt, fine silt, and clay for all three replicates, i.e. UNF, NPK, and AM

| **Order** | **UNF** | | | | | |  | **NPK** | | | | | |  | **AM** | | | | | |
| --- | --- | --- | --- | --- | --- | --- | --- | --- | --- | --- | --- | --- | --- | --- | --- | --- | --- | --- | --- | --- |
|  | **Sand/POM - Coarse silt** | **Sand/POM - Fine silt** | **Sand/POM - Clay** | **Coarse silt - Fine silt** | **Coarse silt - Clay** | **Fine silt - Clay** |  | **Sand/POM - Coarse silt** | **Sand/POM - Fine silt** | **Sand/POM - Clay** | **Coarse silt - Fine silt** | **Coarse silt - Clay** | **Fine silt - Clay** |  | **Sand/POM - Coarse silt** | **Sand/POM - Fine silt** | **Sand/POM - Clay** | **Coarse silt - Fine silt** | **Coarse silt - Clay** | **Fine silt - Clay** |
| Acidimicrobiales | 1.000 | 1.000 | 1.000 | 1.000 | 1.000 | 1.000 |  | 1.000 | 1.000 | 1.000 | 1.000 | 1.000 | 1.000 |  | 1.000 | 1.000 | 1.000 | 1.000 | 1.000 | 1.000 |
| Actinomycetales | 0.696 | **< 0.001** | 0.791 | **< 0.001** | 1.000 | **< 0.001** |  | 1.000 | **< 0.001** | 1.000 | **< 0.001** | 1.000 | **< 0.001** |  | 1.000 | **< 0.001** | 1.000 | **< 0.001** | 1.000 | **0.015** |
| Alteromonadales | 0.168 | 1.000 | 1.000 | 1.000 | 1.000 | 1.000 |  | 1.000 | 1.000 | 1.000 | 1.000 | 0.578 | 1.000 |  | 1.000 | 1.000 | 1.000 | 1.000 | 1.000 | 1.000 |
| Anaerolineales | 1.000 | 1.000 | 0.054 | 1.000 | **0.043** | 0.447 |  | 1.000 | 0.954 | 0.269 | 0.665 | 1.000 | **< 0.001** |  | 1.000 | 1.000 | 0.051 | 0.066 | **< 0.001** | 1.000 |
| Armatimonadales | **< 0.001** | **< 0.001** | **< 0.001** | 1.000 | 1.000 | 1.000 |  | **0.009** | **< 0.001** | **< 0.001** | **0.016** | 1.000 | 1.000 |  | 0.649 | **0.006** | 1.000 | 1.000 | 1.000 | 0.687 |
| Bacillales | **< 0.001** | **< 0.001** | **< 0.001** | **0.038** | 0.492 | 1.000 |  | **< 0.001** | **< 0.001** | **< 0.001** | 0.962 | 0.730 | 1.000 |  | **< 0.001** | **< 0.001** | **< 0.001** | 1.000 | 1.000 | 1.000 |
| Bacteroidales | 0.265 | 0.075 | **0.008** | 1.000 | 1.000 | 1.000 |  | 0.062 | 1.000 | 1.000 | 1.000 | 0.720 | 1.000 |  | 0.413 | 0.118 | 1.000 | 1.000 | 1.000 | 1.000 |
| Bdellovibrionales | 1.000 | 1.000 | 1.000 | 1.000 | 1.000 | 1.000 |  | 0.142 | **0.015** | 0.834 | 1.000 | 1.000 | 1.000 |  | 1.000 | 1.000 | 1.000 | 1.000 | 1.000 | 1.000 |
| Burkholderiales | 1.000 | 0.096 | **< 0.001** | 1.000 | **< 0.001** | **0.005** |  | **0.019** | **< 0.001** | **< 0.001** | 1.000 | **< 0.001** | **0.042** |  | **< 0.001** | **< 0.001** | **< 0.001** | 1.000 | 0.260 | 1.000 |
| Caldilineales | 0.528 | **< 0.001** | **< 0.001** | 0.347 | 0.290 | 1.000 |  | **0.042** | 0.414 | 1.000 | **< 0.001** | **< 0.001** | 1.000 |  | 1.000 | **0.017** | **0.014** | 0.619 | 0.424 | 1.000 |
| Caulobacterales | **< 0.001** | **< 0.001** | **0.002** | 1.000 | 0.154 | **0.018** |  | **< 0.001** | **< 0.001** | 0.751 | 1.000 | 0.289 | **< 0.001** |  | **0.002** | **< 0.001** | **0.040** | 0.066 | 1.000 | **0.002** |
| Chromatiales | 1.000 | **0.016** | **0.019** | 0.959 | 0.854 | 1.000 |  | **0.002** | **< 0.001** | **< 0.001** | 1.000 | 1.000 | 1.000 |  | 0.066 | 0.262 | **0.040** | 1.000 | 1.000 | 1.000 |
| Chthonomonadales | 1.000 | 0.654 | 0.056 | 1.000 | 0.146 | 1.000 |  | 0.146 | **< 0.001** | **< 0.001** | 0.962 | 1.000 | 1.000 |  | 1.000 | 1.000 | 0.639 | 1.000 | 1.000 | 1.000 |
| Clostridiales | **0.013** | **< 0.001** | **< 0.001** | 1.000 | 0.132 | 1.000 |  | **< 0.001** | **< 0.001** | **< 0.001** | 1.000 | 1.000 | 1.000 |  | **< 0.001** | **0.001** | **0.014** | 1.000 | 1.000 | 1.000 |
| Cytophagales | 1.000 | **0.006** | 1.000 | 0.959 | 0.118 | **< 0.001** |  | 1.000 | **0.004** | 1.000 | **0.002** | 1.000 | **< 0.001** |  | 1.000 | 1.000 | **0.014** | 1.000 | **< 0.001** | **< 0.001** |
| Desulfuromonadales | 1.000 | **< 0.001** | **< 0.001** | **< 0.001** | **< 0.001** | 0.061 |  | 1.000 | **< 0.001** | **< 0.001** | **< 0.001** | **< 0.001** | 0.960 |  | 1.000 | **< 0.001** | **< 0.001** | **< 0.001** | **< 0.001** | 1.000 |
| Enterobacteriales | 1.000 | 0.137 | **< 0.001** | 1.000 | **0.012** | 0.447 |  | 1.000 | 0.062 | 0.059 | 0.665 | 0.578 | 1.000 |  | 1.000 | 1.000 | 1.000 | 1.000 | 1.000 | 1.000 |
| Erysipelotrichales | **0.021** | **< 0.001** | **< 0.001** | 0.095 | 1.000 | 1.000 |  | **0.027** | 0.389 | 1.000 | **< 0.001** | **< 0.001** | 1.000 |  | **0.004** | 1.000 | 1.000 | 0.170 | 0.054 | 1.000 |
| Flavobacteriales | **< 0.001** | **< 0.001** | **0.023** | **0.002** | 0.668 | **< 0.001** |  | **< 0.001** | **< 0.001** | 0.600 | 0.166 | 0.720 | **< 0.001** |  | **< 0.001** | **< 0.001** | 0.976 | 1.000 | **< 0.001** | **< 0.001** |
| Gaiellales | **< 0.001** | **< 0.001** | **< 0.001** | **0.015** | 1.000 | **0.028** |  | **< 0.001** | **< 0.001** | **< 0.001** | **0.016** | 1.000 | **0.001** |  | **0.010** | **< 0.001** | **0.008** | **0.002** | 1.000 | **0.003** |
| Gemmatimonadales | **< 0.001** | 1.000 | 0.486 | **< 0.001** | **< 0.001** | 0.056 |  | **< 0.001** | 1.000 | **< 0.001** | **0.016** | **< 0.001** | **< 0.001** |  | 1.000 | **< 0.001** | **< 0.001** | **< 0.001** | **< 0.001** | 0.103 |
| Holophagales | 1.000 | 1.000 | 1.000 | 0.959 | 1.000 | **0.017** |  | 1.000 | 1.000 | 1.000 | 1.000 | 1.000 | 0.910 |  | 1.000 | 0.922 | 0.482 | 1.000 | 0.477 | **< 0.001** |
| Hydrogenophilales | 1.000 | 0.850 | 1.000 | 1.000 | 1.000 | 1.000 |  | 1.000 | 1.000 | 1.000 | 1.000 | 1.000 | 1.000 |  | 0.501 | **0.030** | 0.051 | 1.000 | 1.000 | 1.000 |
| Ktedonobacterales | 1.000 | 0.086 | 0.791 | 0.055 | 0.364 | 1.000 |  | **0.004** | **< 0.001** | **< 0.001** | 1.000 | 1.000 | 1.000 |  | 1.000 | 1.000 | 1.000 | 1.000 | 1.000 | 1.000 |
| Legionellales | **< 0.001** | **0.009** | 1.000 | 1.000 | **< 0.001** | **0.001** |  | 1.000 | 1.000 | **0.028** | 1.000 | 0.324 | **< 0.001** |  | 1.000 | 1.000 | 1.000 | 1.000 | 1.000 | 0.122 |
| Methylophilales | 1.000 | 1.000 | 1.000 | 1.000 | 1.000 | 1.000 |  | **0.003** | **0.013** | **< 0.001** | 1.000 | 1.000 | 1.000 |  | 0.583 | **0.001** | **0.035** | 1.000 | 1.000 | 1.000 |
| Myxococcales | 0.093 | 0.141 | **< 0.001** | **< 0.001** | **< 0.001** | **< 0.001** |  | 1.000 | **0.017** | **< 0.001** | **< 0.001** | **< 0.001** | **< 0.001** |  | 1.000 | **< 0.001** | **< 0.001** | **< 0.001** | **< 0.001** | **< 0.001** |
| Neisseriales | 1.000 | **0.027** | 1.000 | 0.914 | 1.000 | 1.000 |  | 1.000 | 0.954 | 0.662 | 1.000 | 1.000 | 1.000 |  | 0.140 | 1.000 | 1.000 | 1.000 | 1.000 | 1.000 |
| Nitrosomonadales | **< 0.001** | **< 0.001** | **< 0.001** | **< 0.001** | **< 0.001** | **< 0.001** |  | **< 0.001** | **< 0.001** | **< 0.001** | **< 0.001** | **< 0.001** | **< 0.001** |  | **< 0.001** | **< 0.001** | **< 0.001** | **0.001** | **< 0.001** | **< 0.001** |
| Nitrospirales | 0.068 | **< 0.001** | **< 0.001** | **0.001** | **< 0.001** | 1.000 |  | **< 0.001** | **< 0.001** | **< 0.001** | **< 0.001** | **0.009** | 1.000 |  | **0.002** | **< 0.001** | 0.526 | **0.030** | 1.000 | **< 0.001** |
| Opitutales | 0.140 | **< 0.001** | **< 0.001** | 0.588 | 0.914 | 1.000 |  | 1.000 | **< 0.001** | **< 0.001** | **< 0.001** | **0.016** | 1.000 |  | **< 0.001** | **< 0.001** | **< 0.001** | 0.066 | 0.461 | 1.000 |
| Planctomycetales | 1.000 | 1.000 | **< 0.001** | 0.959 | **0.004** | **< 0.001** |  | 0.366 | 0.630 | **< 0.001** | 1.000 | **0.013** | **0.003** |  | 1.000 | **< 0.001** | 1.000 | **< 0.001** | 1.000 | **< 0.001** |
| Pseudomonadales | 0.390 | **< 0.001** | 0.901 | 1.000 | 1.000 | 0.475 |  | 1.000 | **< 0.001** | **< 0.001** | 0.125 | 0.113 | 1.000 |  | **< 0.001** | **< 0.001** | **< 0.001** | 1.000 | 1.000 | 1.000 |
| Rhizobiales | 1.000 | 0.220 | 1.000 | 0.411 | 1.000 | **0.038** |  | 0.904 | **0.013** | 1.000 | 1.000 | 1.000 | 1.000 |  | 1.000 | 1.000 | 0.137 | 1.000 | 0.076 | 0.100 |
| Rhodobacterales | 1.000 | 1.000 | 1.000 | 1.000 | 0.146 | 1.000 |  | **0.010** | 1.000 | 1.000 | 0.857 | 0.720 | 1.000 |  | 1.000 | **< 0.001** | 0.709 | **0.019** | 1.000 | 1.000 |
| Rhodocyclales | 0.265 | **0.003** | 1.000 | 1.000 | 1.000 | 0.753 |  | 1.000 | 0.880 | 1.000 | 0.278 | 1.000 | 0.458 |  | **0.012** | **< 0.001** | **0.008** | 1.000 | 1.000 | 1.000 |
| Rhodospirillales | 1.000 | **< 0.001** | **< 0.001** | **< 0.001** | **< 0.001** | **< 0.001** |  | 1.000 | **< 0.001** | **< 0.001** | **< 0.001** | **< 0.001** | **< 0.001** |  | **0.012** | **< 0.001** | 1.000 | **< 0.001** | **0.048** | **< 0.001** |
| Selenomonadales | 1.000 | 1.000 | 1.000 | 1.000 | 1.000 | 1.000 |  | 1.000 | 0.526 | 1.000 | 0.797 | 1.000 | 1.000 |  | 1.000 | 1.000 | 1.000 | 0.465 | 1.000 | 1.000 |
| Solirubrobacterales | **< 0.001** | **< 0.001** | **0.002** | **0.023** | 1.000 | **< 0.001** |  | **0.001** | **< 0.001** | 0.267 | 1.000 | 1.000 | **0.049** |  | 0.533 | **0.001** | 1.000 | 1.000 | 1.000 | 0.813 |
| Sphingobacteriales | **0.022** | **< 0.001** | 1.000 | 1.000 | **< 0.001** | **< 0.001** |  | 1.000 | 0.209 | 1.000 | 0.665 | 1.000 | 0.402 |  | 1.000 | **0.017** | 1.000 | **0.039** | 1.000 | **0.022** |
| Sphingomonadales | 0.421 | **< 0.001** | **< 0.001** | 1.000 | **< 0.001** | **< 0.001** |  | 1.000 | **< 0.001** | **< 0.001** | **< 0.001** | **< 0.001** | **< 0.001** |  | **0.030** | **< 0.001** | 1.000 | **< 0.001** | **< 0.001** | **< 0.001** |
| Spirochaetales | 0.168 | 0.679 | 0.060 | 1.000 | 1.000 | 1.000 |  | 0.663 | 0.846 | **0.007** | 1.000 | 1.000 | 1.000 |  | 1.000 | **0.043** | **0.040** | 0.905 | 0.630 | 1.000 |
| Verrucomicrobiales | 1.000 | 1.000 | 1.000 | 1.000 | 0.137 | 0.182 |  | 1.000 | 1.000 | 1.000 | 1.000 | 0.093 | 0.407 |  | 1.000 | 1.000 | 1.000 | 1.000 | 1.000 | 1.000 |
| Xanthomonadales | 0.231 | **< 0.001** | 1.000 | **< 0.001** | **0.011** | **< 0.001** |  | **0.024** | **< 0.001** | 1.000 | **< 0.001** | 0.057 | **< 0.001** |  | 0.061 | **< 0.001** | 1.000 | **< 0.001** | 0.056 | **< 0.001** |
| Unclassified orders | **< 0.001** | **< 0.001** | **0.002** | 1.000 | 1.000 | 1.000 |  | **< 0.001** | **< 0.001** | **0.032** | 1.000 | 1.000 | 1.000 |  | 1.000 | 1.000 | **0.014** | 1.000 | 0.056 | 0.146 |

Significant values are given in bold.
