# Supplementary material for: Bacterial Preferences for Specific Soil Particle Size Fractions Revealed by Community Analyses
Source: Front Microbiol. 2018 Feb 23;9:149. doi: 10.3389/fmicb.2018.00149 (PMC5829042; doi:10.3389/fmicb.2018.00149)
Supplement: Supplementary file 11 [file Table11.DOCX]

Table S11 Significance values for family level of bacteria after Bonferroni-correction to account for multiple pair-wise comparisons between sand/POM, coarse silt, fine silt, and clay for all three replicates, i.e. UNF, NPK, and AM

| **Family** | **UNF** | | | | | |  | **NPK** | | | | | |  | **AM** | | | | | |
| --- | --- | --- | --- | --- | --- | --- | --- | --- | --- | --- | --- | --- | --- | --- | --- | --- | --- | --- | --- | --- |
|  | **Sand/POM - Coarse silt** | **Sand/POM - Fine silt** | **Sand/POM - Clay** | **Coarse silt - Fine silt** | **Coarse silt - Clay** | **Fine silt - Clay** |  | **Sand/POM - Coarse silt** | **Sand/POM - Fine silt** | **Sand/POM - Clay** | **Coarse silt - Fine silt** | **Coarse silt - Clay** | **Fine silt - Clay** |  | **Sand/POM - Coarse silt** | **Sand/POM - Fine silt** | **Sand/POM - Clay** | **Coarse silt - Fine silt** | **Coarse silt - Clay** | **Fine silt - Clay** |
| Acetobacteraceae | 0.781 | **< 0.001** | **< 0.001** | **< 0.001** | **< 0.001** | 0.220 |  | 0.070 | **< 0.001** | **< 0.001** | **< 0.001** | **< 0.001** | 0.220 |  | 0.146 | **< 0.001** | **< 0.001** | **< 0.001** | **< 0.001** | 1.000 |
| Acidimicrobiaceae | 1.000 | 1.000 | 1.000 | 1.000 | 1.000 | 1.000 |  | 1.000 | 1.000 | 1.000 | 0.183 | 0.430 | 1.000 |  | 1.000 | 1.000 | 1.000 | 1.000 | 1.000 | 1.000 |
| Acidothermaceae | 1.000 | 1.000 | 1.000 | 1.000 | 1.000 | 1.000 |  | 1.000 | 1.000 | 1.000 | 1.000 | 1.000 | 1.000 |  | 0.789 | 1.000 | 1.000 | 0.203 | 0.825 | 1.000 |
| Actinospicaceae | 1.000 | 0.433 | 0.308 | 0.634 | 0.356 | 1.000 |  | 1.000 | 0.844 | 1.000 | 1.000 | 1.000 | 1.000 |  | 1.000 | 1.000 | 1.000 | 1.000 | 1.000 | 1.000 |
| Alcaligenaceae | 1.000 | 1.000 | 1.000 | 1.000 | 1.000 | 1.000 |  | 0.576 | 0.447 | 0.396 | 1.000 | 1.000 | 1.000 |  | 0.843 | 1.000 | 1.000 | 1.000 | 1.000 | 1.000 |
| Alicyclobacillaceae | **< 0.001** | **< 0.001** | **< 0.001** | 1.000 | 0.188 | 1.000 |  | **< 0.001** | **< 0.001** | **< 0.001** | 1.000 | 1.000 | 1.000 |  | **< 0.001** | **< 0.001** | 0.184 | 1.000 | 1.000 | 1.000 |
| Alteromonadaceae | 0.350 | 1.000 | 1.000 | 1.000 | 1.000 | 1.000 |  | 1.000 | 1.000 | 1.000 | 1.000 | 0.173 | 1.000 |  | 1.000 | 1.000 | 1.000 | 1.000 | 1.000 | 1.000 |
| Anaerolineaceae | 1.000 | 1.000 | 0.114 | 1.000 | 0.294 | **0.029** |  | 1.000 | 1.000 | 0.594 | 0.368 | 1.000 | **0.029** |  | 1.000 | 1.000 | 0.459 | 0.334 | **0.010** | 1.000 |
| Armatimonadaceae | **< 0.001** | **< 0.001** | **< 0.001** | 1.000 | 1.000 | 0.494 |  | **< 0.001** | **< 0.001** | **< 0.001** | **0.038** | 1.000 | 0.494 |  | 0.750 | **0.014** | 1.000 | 1.000 | 1.000 | 0.561 |
| Bacillaceae 1 | **0.004** | **< 0.001** | **< 0.001** | 0.174 | **0.014** | 1.000 |  | **< 0.001** | **< 0.001** | **< 0.001** | 0.139 | **< 0.001** | 1.000 |  | 0.200 | **0.006** | **< 0.001** | 1.000 | 0.512 | 1.000 |
| Bdellovibrionaceae | 1.000 | 1.000 | 1.000 | 1.000 | 1.000 | 1.000 |  | 0.068 | **0.012** | 0.532 | 1.000 | 1.000 | 1.000 |  | 1.000 | 1.000 | 1.000 | 1.000 | 1.000 | 1.000 |
| Bradyrhizobiaceae | 0.590 | 1.000 | **< 0.001** | 1.000 | **0.030** | 0.579 |  | 1.000 | 1.000 | 1.000 | 0.915 | 1.000 | 0.579 |  | 1.000 | 1.000 | **< 0.001** | 1.000 | **0.023** | **0.020** |
| Brucellaceae | 1.000 | 1.000 | 0.469 | 1.000 | 1.000 | 1.000 |  | **0.013** | **< 0.001** | **< 0.001** | 0.139 | **< 0.001** | 1.000 |  | 1.000 | 1.000 | 0.284 | 0.683 | **0.019** | 1.000 |
| Burkholderiaceae | **0.002** | 1.000 | **0.004** | **0.043** | 1.000 | 0.854 |  | 1.000 | **0.005** | 1.000 | **0.033** | 1.000 | 0.854 |  | 1.000 | 0.544 | 1.000 | 0.996 | 1.000 | 1.000 |
| Caldilineaceae | 1.000 | **< 0.001** | **< 0.001** | 0.541 | **0.035** | 1.000 |  | **0.010** | 0.770 | 0.532 | **< 0.001** | **< 0.001** | 1.000 |  | 1.000 | **0.006** | **< 0.001** | 0.334 | **0.046** | 1.000 |
| Catenulisporaceae | 1.000 | 1.000 | 1.000 | 1.000 | 1.000 | 0.453 |  | 1.000 | 1.000 | 1.000 | 1.000 | 1.000 | 0.453 |  | 1.000 | 1.000 | 0.091 | 1.000 | 0.102 | 0.422 |
| Caulobacteraceae | **< 0.001** | **< 0.001** | **0.019** | 1.000 | **0.002** | **< 0.001** |  | **< 0.001** | **< 0.001** | 1.000 | 1.000 | **< 0.001** | **< 0.001** |  | **0.003** | **< 0.001** | 1.000 | 0.445 | 0.496 | **< 0.001** |
| Cellulomonadaceae | 1.000 | 0.848 | **< 0.001** | 0.070 | **< 0.001** | 1.000 |  | 1.000 | 0.303 | 0.435 | **0.038** | **0.043** | 1.000 |  | 1.000 | 0.160 | 0.129 | **0.006** | **0.004** | 1.000 |
| Chitinophagaceae | **0.005** | **< 0.001** | 1.000 | 1.000 | **< 0.001** | **0.006** |  | 1.000 | **0.027** | 1.000 | 1.000 | 0.256 | **0.006** |  | 1.000 | 0.603 | 1.000 | 0.549 | 1.000 | **0.007** |
| Chthonomonadaceae | 1.000 | 1.000 | **0.047** | 1.000 | **0.027** | 1.000 |  | 0.841 | **< 0.001** | **< 0.001** | 0.649 | 0.075 | 1.000 |  | 1.000 | 1.000 | 0.119 | 1.000 | 1.000 | 1.000 |
| Clostridiaceae 1 | 1.000 | 1.000 | **< 0.001** | 1.000 | **< 0.001** | **0.018** |  | **0.001** | **0.004** | **< 0.001** | 1.000 | **0.045** | **0.018** |  | **0.005** | 0.072 | **< 0.001** | 1.000 | 1.000 | 1.000 |
| Comamonadaceae | 0.110 | **< 0.001** | **< 0.001** | **< 0.001** | **< 0.001** | 1.000 |  | **< 0.001** | **< 0.001** | **< 0.001** | **0.012** | 0.078 | 1.000 |  | **< 0.001** | **< 0.001** | **< 0.001** | 0.127 | 1.000 | 1.000 |
| Conexibacteraceae | 1.000 | **0.003** | **< 0.001** | 0.545 | 0.150 | 1.000 |  | 1.000 | 1.000 | 0.204 | 1.000 | 1.000 | 1.000 |  | 1.000 | 0.089 | **0.010** | 0.263 | **0.032** | 1.000 |
| Coxiellaceae | **0.009** | **< 0.001** | 1.000 | 1.000 | 0.357 | **0.026** |  | 1.000 | 1.000 | 0.185 | 1.000 | 0.649 | **0.026** |  | 1.000 | 1.000 | 1.000 | 1.000 | 1.000 | 0.974 |
| Cryomorphaceae | **< 0.001** | **< 0.001** | **0.011** | 1.000 | 1.000 | 0.067 |  | **< 0.001** | **< 0.001** | **< 0.001** | 1.000 | **0.049** | 0.067 |  | **< 0.001** | **< 0.001** | **< 0.001** | 1.000 | 0.475 | 0.651 |
| Cryptosporangiaceae | **< 0.001** | **0.004** | **0.002** | 1.000 | 1.000 | 1.000 |  | 1.000 | 1.000 | 1.000 | 1.000 | 1.000 | 1.000 |  | 0.498 | 0.410 | 0.437 | 1.000 | 1.000 | 1.000 |
| Cystobacteraceae | **< 0.001** | **0.002** | 0.185 | 1.000 | 1.000 | 1.000 |  | **< 0.001** | **< 0.001** | **< 0.001** | 1.000 | 1.000 | 1.000 |  | **0.010** | **< 0.001** | 0.058 | 1.000 | 1.000 | 0.545 |
| Cytophagaceae | 1.000 | 0.101 | 1.000 | 1.000 | **0.035** | **0.007** |  | 1.000 | 0.136 | 1.000 | 0.615 | 1.000 | **0.007** |  | 1.000 | 1.000 | **< 0.001** | 1.000 | **< 0.001** | **< 0.001** |
| Demequinaceae | 1.000 | **< 0.001** | 0.055 | **< 0.001** | 0.160 | 1.000 |  | 1.000 | **0.008** | 0.253 | **0.031** | 0.488 | 1.000 |  | 0.829 | **< 0.001** | **< 0.001** | **< 0.001** | 0.114 | 1.000 |
| Desulfuromonadaceae | 1.000 | 1.000 | 0.826 | 0.107 | **0.011** | 1.000 |  | 0.864 | 1.000 | 1.000 | **0.018** | **0.013** | 1.000 |  | 1.000 | 1.000 | 1.000 | 1.000 | 0.899 | 1.000 |
| Ectothiorhodospiraceae | 1.000 | **0.007** | **0.017** | 0.902 | 1.000 | 1.000 |  | **0.002** | **0.001** | **< 0.001** | 1.000 | 1.000 | 1.000 |  | 0.080 | 0.309 | 0.059 | 1.000 | 1.000 | 1.000 |
| Enterobacteriaceae | 1.000 | 0.057 | **< 0.001** | 1.000 | **0.015** | 1.000 |  | 1.000 | **0.033** | 0.142 | 0.658 | 1.000 | 1.000 |  | 1.000 | 1.000 | 1.000 | 1.000 | 1.000 | 1.000 |
| Erysipelotrichaceae | 0.061 | **< 0.001** | **< 0.001** | 0.255 | 1.000 | 1.000 |  | **0.007** | 1.000 | 1.000 | **< 0.001** | **< 0.001** | 1.000 |  | **0.003** | 1.000 | 1.000 | 0.520 | 0.811 | 1.000 |
| Erythrobacteraceae | 1.000 | **< 0.001** | 0.216 | **0.005** | 1.000 | **< 0.001** |  | 1.000 | **< 0.001** | 1.000 | **< 0.001** | 1.000 | **< 0.001** |  | 0.877 | **< 0.001** | **< 0.001** | **< 0.001** | 0.605 | **0.035** |
| Family I (Cyanobacteria) | **0.001** | **< 0.001** | **0.023** | 0.154 | 1.000 | 0.183 |  | **< 0.001** | **< 0.001** | **< 0.001** | **< 0.001** | 1.000 | 0.183 |  | **< 0.001** | **< 0.001** | **< 0.001** | **< 0.001** | 1.000 | **< 0.001** |
| Flavobacteriaceae | **< 0.001** | **< 0.001** | 0.061 | **< 0.001** | 0.102 | **< 0.001** |  | **< 0.001** | **< 0.001** | 1.000 | 0.368 | **0.012** | **< 0.001** |  | **< 0.001** | **< 0.001** | 1.000 | 1.000 | **< 0.001** | **< 0.001** |
| Gaiellaceae | **0.004** | **< 0.001** | **< 0.001** | 0.998 | 1.000 | 1.000 |  | **< 0.001** | **< 0.001** | **< 0.001** | 0.080 | 1.000 | 1.000 |  | 0.200 | **< 0.001** | **< 0.001** | **0.027** | 1.000 | 1.000 |
| Gemmatimonadaceae | **0.029** | 1.000 | 1.000 | **< 0.001** | **< 0.001** | 0.099 |  | 0.365 | 1.000 | 0.152 | 0.788 | **< 0.001** | 0.099 |  | 1.000 | **0.036** | **< 0.001** | 0.065 | **< 0.001** | 1.000 |
| Geobacteraceae | 1.000 | **< 0.001** | **< 0.001** | **< 0.001** | **< 0.001** | 1.000 |  | 1.000 | **< 0.001** | **< 0.001** | **< 0.001** | **< 0.001** | 1.000 |  | 1.000 | **< 0.001** | **< 0.001** | **< 0.001** | **< 0.001** | 0.844 |
| Geodermatophilaceae | 1.000 | **< 0.001** | 1.000 | 0.156 | 1.000 | 0.194 |  | 1.000 | **0.007** | 1.000 | **0.048** | 1.000 | 0.194 |  | 1.000 | 0.296 | 1.000 | 0.093 | 1.000 | 1.000 |
| Holophagaceae | 1.000 | 0.526 | 0.904 | 0.759 | 1.000 | 0.129 |  | 1.000 | 1.000 | 0.786 | 1.000 | 0.056 | 0.129 |  | 1.000 | 1.000 | 0.111 | 1.000 | 0.114 | **< 0.001** |
| Hydrogenophilaceae | 1.000 | 0.717 | 1.000 | 1.000 | 1.000 | 1.000 |  | 1.000 | 1.000 | 1.000 | 1.000 | 1.000 | 1.000 |  | 0.760 | 0.068 | 0.121 | 1.000 | 1.000 | 1.000 |
| Hyphomicrobiaceae | **< 0.001** | **< 0.001** | **< 0.001** | 1.000 | 0.171 | 1.000 |  | **< 0.001** | **< 0.001** | **< 0.001** | 1.000 | 0.074 | 1.000 |  | **0.045** | **0.003** | **0.029** | 1.000 | 1.000 | 1.000 |
| Intrasporangiaceae | 0.207 | **< 0.001** | **< 0.001** | **< 0.001** | **< 0.001** | 0.653 |  | 0.768 | **< 0.001** | **< 0.001** | **< 0.001** | **< 0.001** | 0.653 |  | 0.399 | **< 0.001** | **< 0.001** | **< 0.001** | **< 0.001** | 1.000 |
| Kineosporiaceae | **< 0.001** | **< 0.001** | **< 0.001** | 1.000 | **< 0.001** | 1.000 |  | 1.000 | **< 0.001** | **< 0.001** | 0.128 | **< 0.001** | 1.000 |  | 0.059 | **< 0.001** | **< 0.001** | 0.203 | **< 0.001** | 1.000 |
| Kofleriaceae | 1.000 | 1.000 | 1.000 | 1.000 | 1.000 | 1.000 |  | 1.000 | **0.027** | 0.062 | 1.000 | 1.000 | 1.000 |  | 1.000 | 1.000 | 1.000 | 1.000 | 1.000 | 1.000 |
| Ktedonobacteraceae | 1.000 | 1.000 | 1.000 | 0.352 | 0.263 | 1.000 |  | **0.005** | **< 0.001** | **< 0.001** | 1.000 | 0.708 | 1.000 |  | 1.000 | 1.000 | 1.000 | 1.000 | 1.000 | 1.000 |
| Lachnospiraceae | **< 0.001** | **< 0.001** | **0.019** | 1.000 | 1.000 | 1.000 |  | **< 0.001** | **< 0.001** | **< 0.001** | 1.000 | 1.000 | 1.000 |  | **< 0.001** | **< 0.001** | 0.078 | 1.000 | 0.509 | 1.000 |
| Legionellaceae | **0.018** | 1.000 | **0.009** | 0.528 | **< 0.001** | **< 0.001** |  | 1.000 | 0.447 | 0.204 | 1.000 | **0.010** | **< 0.001** |  | 1.000 | 1.000 | 0.470 | 1.000 | 0.957 | 0.113 |
| Leptospiraceae | 1.000 | 1.000 | 1.000 | 1.000 | 1.000 | 1.000 |  | 1.000 | 1.000 | 0.703 | 1.000 | 1.000 | 1.000 |  | 1.000 | 1.000 | 1.000 | 1.000 | 1.000 | 1.000 |
| Methylobacteriaceae | 1.000 | 0.511 | 1.000 | 0.759 | 1.000 | 1.000 |  | **< 0.001** | **0.007** | **0.002** | 1.000 | 1.000 | 1.000 |  | 1.000 | 0.100 | 1.000 | 1.000 | 1.000 | 1.000 |
| Methylophilaceae | 1.000 | 1.000 | 1.000 | 1.000 | 1.000 | 1.000 |  | **< 0.001** | **0.005** | **< 0.001** | 1.000 | 1.000 | 1.000 |  | 0.494 | **0.002** | 0.102 | 1.000 | 1.000 | 1.000 |
| Microbacteriaceae | 1.000 | **0.037** | **0.002** | 0.483 | **0.027** | 1.000 |  | 0.138 | **0.021** | **0.043** | 1.000 | 1.000 | 1.000 |  | 1.000 | 1.000 | 1.000 | 1.000 | 1.000 | 1.000 |
| Micrococcaceae | 1.000 | **< 0.001** | **< 0.001** | **< 0.001** | **< 0.001** | 1.000 |  | 1.000 | **< 0.001** | **< 0.001** | **< 0.001** | **< 0.001** | 1.000 |  | 0.146 | **< 0.001** | **< 0.001** | **0.002** | 0.605 | 1.000 |
| Micromonosporaceae | **0.001** | **< 0.001** | **< 0.001** | 1.000 | **< 0.001** | **< 0.001** |  | **< 0.001** | **< 0.001** | **< 0.001** | 1.000 | **< 0.001** | **< 0.001** |  | 0.200 | **0.002** | **< 0.001** | 1.000 | **0.005** | 0.376 |
| Mycobacteriaceae | **0.001** | **< 0.001** | **< 0.001** | **< 0.001** | 0.374 | 1.000 |  | **0.012** | **< 0.001** | **< 0.001** | **< 0.001** | **0.038** | 1.000 |  | **< 0.001** | **< 0.001** | **< 0.001** | 0.229 | 1.000 | 1.000 |
| Myxococcaceae | 1.000 | 0.424 | **< 0.001** | **0.001** | **< 0.001** | **0.001** |  | **0.022** | 1.000 | 0.253 | 1.000 | **< 0.001** | **0.001** |  | 1.000 | 1.000 | **< 0.001** | 1.000 | **< 0.001** | **< 0.001** |
| Nakamurellaceae | **< 0.001** | **< 0.001** | **< 0.001** | **< 0.001** | 1.000 | **< 0.001** |  | **< 0.001** | **< 0.001** | **< 0.001** | **< 0.001** | 0.886 | **< 0.001** |  | **< 0.001** | **< 0.001** | **< 0.001** | **< 0.001** | 1.000 | **< 0.001** |
| Nannocystaceae | 1.000 | **0.018** | 0.561 | 0.179 | 1.000 | 1.000 |  | 1.000 | 0.076 | 1.000 | **0.001** | 0.121 | 1.000 |  | 1.000 | **0.014** | 0.493 | **0.047** | 0.899 | 1.000 |
| Neisseriaceae | 1.000 | **0.018** | 1.000 | 0.684 | 1.000 | 1.000 |  | 1.000 | 1.000 | 1.000 | 1.000 | 1.000 | 1.000 |  | 0.190 | 1.000 | 1.000 | 1.000 | 1.000 | 1.000 |
| Nitrosomonadaceae | **< 0.001** | **< 0.001** | **0.002** | **0.003** | **< 0.001** | **< 0.001** |  | **< 0.001** | **< 0.001** | **< 0.001** | **< 0.001** | **< 0.001** | **< 0.001** |  | **< 0.001** | **< 0.001** | **< 0.001** | **0.015** | **< 0.001** | **< 0.001** |
| Nitrospiraceae | 1.000 | **< 0.001** | **< 0.001** | **0.044** | **< 0.001** | 1.000 |  | 0.148 | **< 0.001** | **< 0.001** | **< 0.001** | **< 0.001** | 1.000 |  | **0.003** | **< 0.001** | **0.003** | **0.010** | 1.000 | **0.007** |
| Nocardiaceae | **0.005** | 0.186 | 0.074 | 1.000 | 1.000 | 1.000 |  | 1.000 | **< 0.001** | **< 0.001** | **0.030** | **0.045** | 1.000 |  | **0.003** | **< 0.001** | 0.052 | 1.000 | 1.000 | 0.707 |
| Nocardioidaceae | 1.000 | **< 0.001** | **< 0.001** | **0.044** | **< 0.001** | 1.000 |  | 1.000 | **< 0.001** | **< 0.001** | **0.027** | **< 0.001** | 1.000 |  | 1.000 | **< 0.001** | **< 0.001** | 0.163 | **0.008** | 1.000 |
| Opitutaceae | **0.014** | **< 0.001** | **< 0.001** | 0.156 | 1.000 | 1.000 |  | 0.698 | **< 0.001** | **< 0.001** | **0.005** | 0.967 | 1.000 |  | **< 0.001** | **< 0.001** | **< 0.001** | 0.229 | 1.000 | 1.000 |
| Oxalobacteraceae | 0.211 | **0.032** | 0.248 | 1.000 | 1.000 | 1.000 |  | **0.007** | **< 0.001** | **< 0.001** | 0.082 | **0.004** | 1.000 |  | 0.146 | **< 0.001** | **< 0.001** | 1.000 | 1.000 | 1.000 |
| Paenibacillaceae 2 | 0.782 | 1.000 | 0.867 | 1.000 | 1.000 | 1.000 |  | 1.000 | 1.000 | 0.995 | 1.000 | 0.649 | 1.000 |  | 1.000 | 1.000 | 1.000 | 1.000 | 1.000 | 1.000 |
| Peptococcaceae 1 | 1.000 | 1.000 | 1.000 | 1.000 | 1.000 | 1.000 |  | 0.149 | 1.000 | 1.000 | **0.012** | **0.002** | 1.000 |  | 1.000 | 1.000 | 0.570 | 1.000 | 0.605 | 1.000 |
| Phaselicystidaceae | 1.000 | 1.000 | 1.000 | 1.000 | 1.000 | 1.000 |  | 1.000 | 1.000 | 1.000 | 1.000 | 1.000 | 1.000 |  | 1.000 | 1.000 | 1.000 | 1.000 | 1.000 | 1.000 |
| Phyllobacteriaceae | 0.118 | 1.000 | **0.012** | 1.000 | **< 0.001** | **< 0.001** |  | 0.082 | 1.000 | 0.067 | 1.000 | **< 0.001** | **< 0.001** |  | 1.000 | 1.000 | 1.000 | 1.000 | 1.000 | 1.000 |
| Planctomycetaceae | 1.000 | 0.364 | **0.016** | 1.000 | **0.004** | **0.001** |  | 1.000 | 1.000 | **< 0.001** | 1.000 | **< 0.001** | **0.001** |  | 1.000 | **0.013** | 1.000 | 0.329 | 0.496 | **< 0.001** |
| Planococcaceae | **< 0.001** | **< 0.001** | **< 0.001** | 0.066 | 0.081 | 1.000 |  | **0.007** | **< 0.001** | **< 0.001** | **0.026** | **< 0.001** | 1.000 |  | **< 0.001** | **< 0.001** | **< 0.001** | 0.549 | 0.496 | 1.000 |
| Polyangiaceae | 1.000 | 1.000 | **0.003** | 0.220 | **< 0.001** | 0.882 |  | 0.698 | 0.057 | **< 0.001** | 1.000 | 0.071 | 0.882 |  | 1.000 | 1.000 | **< 0.001** | 1.000 | **0.032** | 0.376 |
| Pseudomonadaceae | 0.106 | **< 0.001** | 1.000 | 0.528 | 1.000 | 1.000 |  | 0.511 | **< 0.001** | **< 0.001** | 0.139 | 1.000 | 1.000 |  | **< 0.001** | **< 0.001** | **< 0.001** | 1.000 | 1.000 | 1.000 |
| Pseudonocardiaceae | **< 0.001** | **< 0.001** | **< 0.001** | 0.759 | **< 0.001** | 1.000 |  | **< 0.001** | **< 0.001** | **< 0.001** | **0.012** | **< 0.001** | 1.000 |  | **< 0.001** | **< 0.001** | **< 0.001** | 1.000 | 0.231 | 1.000 |
| Rhizobiaceae | **< 0.001** | **< 0.001** | **< 0.001** | 0.968 | 1.000 | 1.000 |  | **< 0.001** | **< 0.001** | **< 0.001** | 1.000 | 1.000 | 1.000 |  | 0.252 | **< 0.001** | **< 0.001** | 0.093 | 0.111 | 1.000 |
| Rhodobacteraceae | 0.171 | 1.000 | 1.000 | 1.000 | **0.001** | 1.000 |  | **< 0.001** | 0.502 | 1.000 | 0.139 | **< 0.001** | 1.000 |  | 1.000 | **< 0.001** | **0.004** | **< 0.001** | 0.105 | 1.000 |
| Rhodocyclaceae | 0.118 | **< 0.001** | 1.000 | 1.000 | 1.000 | **0.043** |  | 1.000 | 0.201 | 1.000 | 0.681 | 1.000 | **0.043** |  | **0.027** | **< 0.001** | 0.085 | 1.000 | 1.000 | 1.000 |
| Rhodospirillaceae | 1.000 | **< 0.001** | 1.000 | **< 0.001** | 1.000 | **< 0.001** |  | 0.394 | **< 0.001** | 1.000 | **< 0.001** | **0.029** | **< 0.001** |  | **< 0.001** | **0.006** | **< 0.001** | **< 0.001** | 1.000 | **< 0.001** |
| Ruminococcaceae | 0.207 | **0.003** | **0.010** | 1.000 | 1.000 | 1.000 |  | 0.149 | 0.329 | 0.253 | 1.000 | 1.000 | 1.000 |  | 0.200 | 0.291 | 1.000 | 1.000 | 1.000 | 1.000 |
| Sanguibacteraceae | 1.000 | 1.000 | 1.000 | 1.000 | 1.000 | 1.000 |  | 1.000 | 1.000 | 1.000 | 1.000 | 1.000 | 1.000 |  | 1.000 | 1.000 | 1.000 | 1.000 | 1.000 | 1.000 |
| Sinobacteraceae | 1.000 | **< 0.001** | **< 0.001** | **< 0.001** | **< 0.001** | 1.000 |  | 1.000 | 0.456 | **0.002** | **0.048** | **< 0.001** | 1.000 |  | 1.000 | **< 0.001** | **< 0.001** | **< 0.001** | **< 0.001** | 1.000 |
| Solirubrobacteraceae | **< 0.001** | **< 0.001** | **0.048** | 0.064 | 1.000 | **0.002** |  | **0.024** | **< 0.001** | 1.000 | 0.337 | 1.000 | **0.002** |  | 0.333 | **0.002** | 0.923 | 1.000 | 1.000 | 1.000 |
| Sphingobacteriaceae | **< 0.001** | **< 0.001** | 0.100 | 1.000 | 0.107 | 1.000 |  | 0.135 | **< 0.001** | 0.229 | 1.000 | 1.000 | 1.000 |  | **0.026** | **0.017** | 1.000 | 1.000 | **0.005** | **0.007** |
| Sphingomonadaceae | 0.074 | **< 0.001** | **< 0.001** | 1.000 | **< 0.001** | **< 0.001** |  | 1.000 | **0.012** | **< 0.001** | 1.000 | **< 0.001** | **< 0.001** |  | 0.536 | **< 0.001** | **< 0.001** | 1.000 | **< 0.001** | **< 0.001** |
| Streptomycetaceae | **< 0.001** | **< 0.001** | **0.006** | 1.000 | 0.630 | **0.003** |  | 0.080 | **< 0.001** | 1.000 | 1.000 | 0.649 | **0.003** |  | 1.000 | 1.000 | 1.000 | 1.000 | 0.825 | 0.054 |
| Streptosporangiaceae | 1.000 | 1.000 | 0.535 | 1.000 | 0.278 | 1.000 |  | **< 0.001** | **0.002** | 0.159 | 1.000 | 0.821 | 1.000 |  | 1.000 | 1.000 | 0.461 | 1.000 | 1.000 | 1.000 |
| Thermoactino-mycetaceae 1 | **< 0.001** | **< 0.001** | **< 0.001** | 1.000 | 1.000 | **0.007** |  | 1.000 | 0.865 | 1.000 | 1.000 | 0.299 | **0.007** |  | 0.422 | 0.820 | 1.000 | 1.000 | 1.000 | 1.000 |
| Thermoactino-mycetaceae 2 | 0.183 | 0.526 | 1.000 | 1.000 | 0.311 | 1.000 |  | 1.000 | 0.362 | 1.000 | 0.659 | 1.000 | 1.000 |  | 1.000 | 1.000 | 1.000 | 1.000 | 1.000 | 1.000 |
| Thermomonosporaceae | 1.000 | 1.000 | 1.000 | 1.000 | 1.000 | 1.000 |  | 1.000 | 1.000 | 1.000 | 0.368 | 1.000 | 1.000 |  | 1.000 | 1.000 | 1.000 | 1.000 | 1.000 | 1.000 |
| Veillonellaceae | 1.000 | 1.000 | 1.000 | 1.000 | 1.000 | 1.000 |  | 1.000 | 0.182 | 1.000 | 1.000 | 1.000 | 1.000 |  | 1.000 | 1.000 | 1.000 | 1.000 | 1.000 | 1.000 |
| Verrucomicrobiaceae | 1.000 | 1.000 | 1.000 | 1.000 | **0.014** | **0.031** |  | 0.152 | 1.000 | 1.000 | 1.000 | **< 0.001** | **0.031** |  | 1.000 | 1.000 | 1.000 | 1.000 | 0.821 | 1.000 |
| Xanthobacteraceae | 1.000 | 1.000 | 1.000 | 1.000 | 1.000 | 1.000 |  | 1.000 | 1.000 | 0.438 | 1.000 | 0.815 | 1.000 |  | 1.000 | 0.077 | 1.000 | 1.000 | 1.000 | 1.000 |
| Xanthomonadaceae | **< 0.001** | **< 0.001** | 0.360 | **0.012** | **< 0.001** | **< 0.001** |  | **< 0.001** | **< 0.001** | 1.000 | **0.022** | **< 0.001** | **< 0.001** |  | **0.013** | **< 0.001** | **0.014** | **0.009** | **< 0.001** | **< 0.001** |
| Unclassified families | 1.000 | 1.000 | 1.000 | 1.000 | 1.000 | 1.000 |  | 1.000 | 1.000 | 1.000 | 1.000 | 1.000 | 1.000 |  | 1.000 | 1.000 | 1.000 | 1.000 | 1.000 | 1.000 |

Significant values are given in bold.
