# Supplementary material for: Bacterial Preferences for Specific Soil Particle Size Fractions Revealed by Community Analyses
Source: Front Microbiol. 2018 Feb 23;9:149. doi: 10.3389/fmicb.2018.00149 (PMC5829042; doi:10.3389/fmicb.2018.00149)
Supplement: Supplementary file 12 [file Table12.DOCX]

Table S12 Significance values for genus level of bacteria after Bonferroni-correction to account for multiple pair-wise comparisons between sand/POM, coarse silt, fine silt, and clay for all three replicates, i.e. UNF, NPK, and AM

| **Genus** | **UNF** | | | | | |  | **NPK** | | | | | |  | **AM** | | | | | |
| --- | --- | --- | --- | --- | --- | --- | --- | --- | --- | --- | --- | --- | --- | --- | --- | --- | --- | --- | --- | --- |
|  | **Sand/POM - Coarse silt** | **Sand/POM - Fine silt** | **Sand/POM - Clay** | **Coarse silt - Fine silt** | **Coarse silt - Clay** | **Fine silt - Clay** |  | **Sand/POM - Coarse silt** | **Sand/POM - Fine silt** | **Sand/POM - Clay** | **Coarse silt - Fine silt** | **Coarse silt - Clay** | **Fine silt - Clay** |  | **Sand/POM - Coarse silt** | **Sand/POM - Fine silt** | **Sand/POM - Clay** | **Coarse silt - Fine silt** | **Coarse silt - Clay** | **Fine silt - Clay** |
| Acidiferrobacter | 1.000 | **0.013** | **0.010** | 1.000 | 0.899 | 1.000 |  | **0.003** | **0.002** | **< 0.001** | 1.000 | 1.000 | 1.000 |  | 0.119 | 0.626 | 0.053 | 1.000 | 1.000 | 1.000 |
| Acidothermus | 1.000 | 1.000 | 1.000 | 1.000 | 1.000 | 0.994 |  | 1.000 | 1.000 | 1.000 | 1.000 | 1.000 | 1.000 |  | 0.977 | 1.000 | 1.000 | 0.092 | 1.000 | 1.000 |
| Actinoallomurus | 0.551 | **0.015** | 0.306 | 1.000 | 1.000 | 1.000 |  | 1.000 | 0.327 | 1.000 | 0.992 | 1.000 | 1.000 |  | 1.000 | 1.000 | 1.000 | 1.000 | 1.000 | 1.000 |
| Actinoplanes | **< 0.001** | **< 0.001** | **< 0.001** | 0.151 | **< 0.001** | 0.673 |  | **< 0.001** | **< 0.001** | **< 0.001** | 1.000 | 0.362 | 1.000 |  | 0.394 | **0.048** | **< 0.001** | 1.000 | **0.008** | 0.058 |
| Actinospica | 1.000 | 0.394 | 0.904 | 0.534 | 0.967 | 1.000 |  | 1.000 | 0.371 | 1.000 | 1.000 | 1.000 | 1.000 |  | 1.000 | 1.000 | 1.000 | 1.000 | 1.000 | 1.000 |
| Adhaeribacter | 1.000 | 1.000 | 1.000 | 1.000 | **0.013** | 0.066 |  | 1.000 | 1.000 | 0.152 | 0.586 | 1.000 | **0.012** |  | 1.000 | 1.000 | **< 0.001** | 1.000 | **< 0.001** | **0.014** |
| Aeromicrobium | **< 0.001** | **< 0.001** | 1.000 | 1.000 | **< 0.001** | **< 0.001** |  | 0.120 | 1.000 | **< 0.001** | 1.000 | **< 0.001** | **< 0.001** |  | **0.006** | 0.284 | 1.000 | 1.000 | **< 0.001** | **< 0.001** |
| Afipia | **< 0.001** | **< 0.001** | **< 0.001** | **0.013** | **0.011** | 1.000 |  | **< 0.001** | **< 0.001** | **< 0.001** | **0.002** | 0.541 | 1.000 |  | **< 0.001** | **< 0.001** | **< 0.001** | 0.186 | 1.000 | 1.000 |
| Agromyces | 0.109 | 0.194 | 1.000 | 1.000 | 0.474 | 1.000 |  | **< 0.001** | 0.416 | 0.269 | 0.968 | 0.651 | 1.000 |  | 0.119 | **< 0.001** | 0.599 | 1.000 | 1.000 | 0.217 |
| Albidiferax | 0.059 | 0.051 | 0.163 | 1.000 | 1.000 | 1.000 |  | 1.000 | 0.609 | 1.000 | 1.000 | 1.000 | 1.000 |  | 0.080 | **< 0.001** | **0.003** | 1.000 | 1.000 | 1.000 |
| Alicyclobacillus | 1.000 | 1.000 | 1.000 | 1.000 | 1.000 | 1.000 |  | 0.757 | 1.000 | 1.000 | 1.000 | 1.000 | 1.000 |  | 1.000 | 1.000 | 1.000 | 1.000 | 1.000 | 1.000 |
| Allocatelliglobosispora | 0.134 | **0.012** | 1.000 | 1.000 | 0.633 | 0.109 |  | 0.678 | 0.570 | 1.000 | 1.000 | 0.099 | 0.140 |  | 1.000 | 0.115 | 1.000 | 0.648 | 1.000 | **0.005** |
| Amaricoccus | 1.000 | 0.326 | 0.188 | 0.749 | 0.369 | 1.000 |  | 1.000 | 1.000 | 0.125 | **0.048** | **< 0.001** | 1.000 |  | 0.394 | **< 0.001** | **0.004** | **0.002** | 1.000 | 0.113 |
| Aminobacter | **0.005** | **< 0.001** | 1.000 | 1.000 | 0.102 | **0.046** |  | 1.000 | 1.000 | 1.000 | 1.000 | 0.156 | 0.496 |  | 1.000 | 1.000 | 1.000 | 1.000 | 1.000 | 1.000 |
| Ammoniphilus | 0.708 | 1.000 | 1.000 | 1.000 | 1.000 | 1.000 |  | 1.000 | 1.000 | 1.000 | 1.000 | 1.000 | 1.000 |  | 1.000 | 1.000 | 1.000 | 1.000 | 1.000 | 1.000 |
| Amycolatopsis | 0.094 | **< 0.001** | **< 0.001** | **0.009** | **< 0.001** | 1.000 |  | **0.001** | **< 0.001** | **< 0.001** | **0.009** | **< 0.001** | 1.000 |  | **< 0.001** | **< 0.001** | **< 0.001** | 1.000 | 0.056 | 0.934 |
| Anaeromyxobacter | 1.000 | 1.000 | 1.000 | 1.000 | 1.000 | 1.000 |  | 1.000 | 1.000 | 1.000 | 1.000 | 1.000 | 1.000 |  | 1.000 | 1.000 | 1.000 | 1.000 | 1.000 | 1.000 |
| Angustibacter | 1.000 | 1.000 | **0.002** | 1.000 | **< 0.001** | **0.001** |  | 0.382 | 1.000 | 1.000 | 1.000 | **< 0.001** | 0.690 |  | 1.000 | 1.000 | 1.000 | 1.000 | **0.006** | **0.025** |
| Aquicella | **0.002** | **< 0.001** | 0.680 | 1.000 | 1.000 | 0.220 |  | 1.000 | 1.000 | 0.468 | 1.000 | 1.000 | 0.216 |  | 1.000 | 1.000 | 1.000 | 1.000 | 1.000 | 1.000 |
| Arenimonas | 0.290 | **0.013** | **< 0.001** | 1.000 | **< 0.001** | **< 0.001** |  | **0.004** | **0.009** | **< 0.001** | 1.000 | **< 0.001** | **< 0.001** |  | 0.119 | **0.006** | **< 0.001** | 1.000 | **< 0.001** | **< 0.001** |
| Armatimonadetes gp4 | 1.000 | 1.000 | **0.010** | 0.607 | **< 0.001** | 0.756 |  | 1.000 | 0.915 | **0.015** | 0.992 | **0.019** | 1.000 |  | 1.000 | **< 0.001** | **0.002** | **0.023** | 0.349 | 1.000 |
| Armatimonadetes gp5 | 1.000 | 1.000 | 1.000 | 1.000 | 1.000 | 1.000 |  | 1.000 | 1.000 | 1.000 | 1.000 | 1.000 | 1.000 |  | 1.000 | 1.000 | 0.345 | 1.000 | 1.000 | 1.000 |
| Armatimonas/ Armatimonadetes gp1 | **< 0.001** | **< 0.001** | **< 0.001** | 1.000 | 1.000 | 1.000 |  | **0.004** | **< 0.001** | **< 0.001** | 0.062 | 1.000 | 1.000 |  | 0.897 | 0.058 | 1.000 | 1.000 | 1.000 | 1.000 |
| Arthrobacter | 1.000 | **< 0.001** | **0.001** | **< 0.001** | **0.007** | **0.025** |  | 1.000 | **< 0.001** | **< 0.001** | **< 0.001** | **0.002** | **0.003** |  | 0.062 | **< 0.001** | **< 0.001** | **< 0.001** | 1.000 | **< 0.001** |
| Asticcacaulis | **< 0.001** | **< 0.001** | **< 0.001** | 1.000 | 1.000 | 1.000 |  | **0.044** | 0.350 | 1.000 | 1.000 | 1.000 | 1.000 |  | 0.801 | 1.000 | 1.000 | 1.000 | 1.000 | 1.000 |
| Bacillus | **< 0.001** | **< 0.001** | **< 0.001** | **0.017** | 1.000 | 1.000 |  | **< 0.001** | **< 0.001** | **< 0.001** | **0.010** | 0.106 | 1.000 |  | 0.106 | **< 0.001** | **< 0.001** | 0.969 | 1.000 | 1.000 |
| Blastococcus | 1.000 | **0.010** | 0.188 | 0.316 | **0.012** | **< 0.001** |  | 1.000 | **< 0.001** | 1.000 | 0.101 | 0.635 | **< 0.001** |  | 1.000 | **0.004** | 1.000 | **0.002** | 1.000 | **< 0.001** |
| Blastopirellula | 1.000 | 1.000 | 0.059 | 1.000 | 1.000 | 1.000 |  | 0.165 | **0.002** | 0.082 | 1.000 | 1.000 | 1.000 |  | 1.000 | 1.000 | 1.000 | 1.000 | 1.000 | 1.000 |
| Bosea | 1.000 | 0.497 | **0.033** | 1.000 | 1.000 | 1.000 |  | 1.000 | 1.000 | 1.000 | 1.000 | 1.000 | 1.000 |  | 1.000 | 1.000 | 1.000 | 1.000 | 1.000 | 1.000 |
| Bradyrhizobium | **0.009** | 1.000 | **< 0.001** | 0.316 | **< 0.001** | **< 0.001** |  | 1.000 | **0.002** | **< 0.001** | **0.007** | **< 0.001** | **< 0.001** |  | 1.000 | 1.000 | **< 0.001** | 1.000 | **< 0.001** | **< 0.001** |
| Brevundimonas | 1.000 | **0.001** | 0.668 | 0.164 | 1.000 | 0.467 |  | **0.002** | **0.002** | 1.000 | 1.000 | 0.255 | 0.333 |  | 0.106 | 0.144 | 0.919 | 1.000 | 1.000 | 1.000 |
| Burkholderia | **< 0.001** | 1.000 | **< 0.001** | **0.024** | 1.000 | **< 0.001** |  | 1.000 | **< 0.001** | 1.000 | **0.043** | 1.000 | **0.012** |  | 1.000 | 0.129 | 1.000 | 0.186 | 1.000 | **0.020** |
| Byssovorax | **0.009** | **< 0.001** | 1.000 | 1.000 | **0.001** | **< 0.001** |  | **0.002** | **< 0.001** | 1.000 | 1.000 | 0.098 | **< 0.001** |  | 0.888 | **0.003** | 0.518 | 1.000 | **< 0.001** | **< 0.001** |
| Candidatus Solibacter | 1.000 | **0.017** | **0.013** | **0.043** | **0.021** | 1.000 |  | 1.000 | **< 0.001** | **< 0.001** | **0.002** | 0.293 | 1.000 |  | 0.921 | **< 0.001** | **0.048** | **0.020** | 1.000 | 0.331 |
| Catellatospora | 1.000 | 0.561 | 1.000 | 1.000 | 1.000 | 1.000 |  | 1.000 | 0.135 | 1.000 | 0.969 | 1.000 | 1.000 |  | 1.000 | 1.000 | 1.000 | 1.000 | 1.000 | 1.000 |
| Catelliglobosispora | 0.495 | **0.017** | 0.054 | 1.000 | 1.000 | 1.000 |  | 1.000 | 0.218 | 0.627 | 0.780 | 1.000 | 1.000 |  | 1.000 | 0.587 | 0.909 | 1.000 | 1.000 | 1.000 |
| Catenulispora | 1.000 | 1.000 | 1.000 | 1.000 | 1.000 | 1.000 |  | 1.000 | 1.000 | 1.000 | 1.000 | 1.000 | 1.000 |  | 1.000 | 1.000 | 0.448 | 1.000 | 0.479 | 1.000 |
| Caulobacter | **< 0.001** | **< 0.001** | **< 0.001** | 1.000 | 1.000 | 0.813 |  | **< 0.001** | **< 0.001** | **< 0.001** | 0.931 | 1.000 | 0.190 |  | **< 0.001** | **< 0.001** | **< 0.001** | 1.000 | 1.000 | 1.000 |
| Cellulomonas | 1.000 | 1.000 | **< 0.001** | 0.224 | **< 0.001** | **0.008** |  | 1.000 | 1.000 | **0.027** | 0.136 | **< 0.001** | 1.000 |  | 1.000 | 1.000 | **0.013** | 0.123 | **< 0.001** | 1.000 |
| Cellulosilyticum | 1.000 | 1.000 | 1.000 | 1.000 | 1.000 | 1.000 |  | 1.000 | 1.000 | 1.000 | 1.000 | 1.000 | 1.000 |  | 1.000 | 1.000 | 1.000 | 1.000 | 1.000 | 1.000 |
| Cellvibrio | 1.000 | 0.080 | 1.000 | 1.000 | 1.000 | 1.000 |  | 1.000 | 1.000 | 1.000 | 1.000 | 0.536 | 1.000 |  | 1.000 | **0.049** | **0.007** | 0.969 | 0.199 | 1.000 |
| Chitinibacter | 1.000 | 0.076 | 0.344 | 1.000 | 1.000 | 1.000 |  | 0.310 | 1.000 | 1.000 | 1.000 | 1.000 | 1.000 |  | 0.465 | 1.000 | 0.250 | 1.000 | 1.000 | 1.000 |
| Chitinophaga | **< 0.001** | **< 0.001** | **< 0.001** | 0.209 | **0.012** | 1.000 |  | **< 0.001** | **0.010** | 0.115 | 0.748 | 0.073 | 1.000 |  | 0.106 | 0.054 | 1.000 | 1.000 | 1.000 | 1.000 |
| Chondromyces | 1.000 | 0.341 | **0.033** | **0.035** | **0.001** | 1.000 |  | 1.000 | 1.000 | **< 0.001** | 0.914 | **< 0.001** | **0.036** |  | 1.000 | 1.000 | **0.014** | 1.000 | **0.027** | 0.324 |
| Chthonomonas/ Armatimonadetes gp3 | 1.000 | 1.000 | 1.000 | 1.000 | 1.000 | 1.000 |  | 0.606 | **< 0.001** | **< 0.001** | 0.586 | 1.000 | 1.000 |  | 1.000 | 1.000 | 0.695 | 1.000 | 1.000 | 1.000 |
| Clostridium III | 0.551 | 0.195 | 1.000 | 1.000 | 1.000 | 1.000 |  | 1.000 | 1.000 | 1.000 | 1.000 | 1.000 | 1.000 |  | 0.980 | 0.658 | 1.000 | 1.000 | 1.000 | 0.936 |
| Clostridium sensu stricto | 1.000 | 1.000 | 0.076 | 1.000 | 0.232 | 1.000 |  | **< 0.001** | **< 0.001** | **< 0.001** | 1.000 | 1.000 | 1.000 |  | **0.012** | **0.004** | 0.067 | 1.000 | 1.000 | 1.000 |
| Clostridium XlVa | 1.000 | 0.442 | 1.000 | **0.036** | 1.000 | 0.538 |  | **< 0.001** | **< 0.001** | **0.001** | 1.000 | 1.000 | 1.000 |  | 0.370 | 0.342 | 1.000 | 1.000 | 1.000 | 1.000 |
| Comamonas | 0.426 | 1.000 | 0.259 | 1.000 | 1.000 | 1.000 |  | 0.069 | 1.000 | 1.000 | 1.000 | 1.000 | 1.000 |  | 1.000 | **0.005** | 0.188 | 0.113 | 1.000 | 1.000 |
| Conexibacter | 1.000 | **0.004** | 0.937 | 0.308 | 1.000 | 1.000 |  | 1.000 | 0.209 | 1.000 | 1.000 | 1.000 | 1.000 |  | 1.000 | **0.003** | 0.492 | **0.014** | 1.000 | 1.000 |
| Corallococcus | 1.000 | 0.080 | **< 0.001** | **0.001** | **< 0.001** | **< 0.001** |  | **< 0.001** | 1.000 | **0.001** | 0.604 | **< 0.001** | **< 0.001** |  | 1.000 | 1.000 | **< 0.001** | 1.000 | **< 0.001** | **< 0.001** |
| Cryptosporangium | **< 0.001** | **0.004** | **< 0.001** | 1.000 | 1.000 | 1.000 |  | 1.000 | 1.000 | 1.000 | 1.000 | 1.000 | 1.000 |  | 0.698 | 0.709 | 0.392 | 1.000 | 1.000 | 1.000 |
| Cystobacter | **0.044** | **< 0.001** | 0.609 | 1.000 | 1.000 | 0.350 |  | **< 0.001** | **< 0.001** | **0.004** | 1.000 | 1.000 | 0.268 |  | 0.063 | **< 0.001** | 0.053 | 0.116 | 1.000 | 0.064 |
| Cytophaga | 1.000 | 1.000 | 0.668 | 1.000 | 1.000 | 1.000 |  | 1.000 | 0.415 | **0.001** | 1.000 | 0.054 | 1.000 |  | 1.000 | 0.669 | 0.066 | 1.000 | 1.000 | 1.000 |
| Demequina | 1.000 | **< 0.001** | **< 0.001** | **< 0.001** | **< 0.001** | 1.000 |  | 1.000 | 0.117 | **0.006** | 0.065 | **0.003** | 1.000 |  | 0.956 | **< 0.001** | **< 0.001** | **0.017** | **0.005** | 1.000 |
| Desulfuromonas | 1.000 | 1.000 | 1.000 | 0.120 | 0.129 | 1.000 |  | 1.000 | 0.570 | 1.000 | **0.015** | 0.096 | 1.000 |  | 1.000 | 1.000 | 1.000 | 1.000 | 1.000 | 1.000 |
| Devosia | **0.034** | **< 0.001** | **< 0.001** | 0.195 | 1.000 | 1.000 |  | 1.000 | **0.024** | 1.000 | 1.000 | 1.000 | 1.000 |  | **0.003** | **< 0.001** | **0.001** | 1.000 | 1.000 | 1.000 |
| Dokdonella | **< 0.001** | **< 0.001** | **< 0.001** | **< 0.001** | **0.001** | 1.000 |  | 0.313 | **< 0.001** | **< 0.001** | **< 0.001** | **< 0.001** | 1.000 |  | 0.054 | **< 0.001** | **< 0.001** | **< 0.001** | 0.062 | 1.000 |
| Dongia | **< 0.001** | **< 0.001** | **< 0.001** | **0.005** | **0.032** | 1.000 |  | **0.012** | **< 0.001** | **< 0.001** | **0.003** | 0.136 | 1.000 |  | **< 0.001** | **< 0.001** | **< 0.001** | **< 0.001** | **0.001** | 1.000 |
| Duganella | **0.046** | **< 0.001** | **< 0.001** | 1.000 | 1.000 | 1.000 |  | **0.020** | **< 0.001** | **0.001** | 1.000 | 1.000 | 1.000 |  | 1.000 | **< 0.001** | **< 0.001** | 0.084 | 0.342 | 1.000 |
| Dyella | 1.000 | 1.000 | 1.000 | 1.000 | 1.000 | 1.000 |  | 1.000 | 1.000 | 1.000 | 1.000 | 1.000 | 1.000 |  | 1.000 | 1.000 | 1.000 | 1.000 | 1.000 | 1.000 |
| Edaphobacter | 1.000 | 1.000 | 1.000 | 1.000 | 1.000 | 1.000 |  | 1.000 | **0.008** | 0.098 | **0.029** | 0.257 | 1.000 |  | 1.000 | 0.068 | 1.000 | 1.000 | 1.000 | 0.557 |
| Escherichia/Shigella | 1.000 | 0.080 | **< 0.001** | 1.000 | **0.008** | 0.467 |  | 1.000 | 0.156 | **0.010** | 1.000 | 0.668 | 1.000 |  | 1.000 | 1.000 | 1.000 | 1.000 | 1.000 | 1.000 |
| Ferruginibacter | 0.665 | 0.868 | 1.000 | 1.000 | **0.004** | **0.010** |  | 1.000 | 1.000 | **0.022** | 1.000 | **0.024** | 0.174 |  | 1.000 | 1.000 | **0.045** | 1.000 | **0.039** | 0.315 |
| Flavisolibacter | 1.000 | 1.000 | 1.000 | 1.000 | 1.000 | 1.000 |  | **0.014** | **0.008** | 0.168 | 1.000 | 1.000 | 1.000 |  | 1.000 | 1.000 | 1.000 | 1.000 | 1.000 | 1.000 |
| Flavobacterium | **< 0.001** | **< 0.001** | **< 0.001** | **0.012** | 1.000 | **< 0.001** |  | **0.010** | **< 0.001** | 1.000 | 1.000 | 1.000 | **0.002** |  | **< 0.001** | **< 0.001** | 1.000 | 1.000 | **< 0.001** | **0.002** |
| Fluviicola | **< 0.001** | **< 0.001** | **0.002** | 1.000 | 0.395 | 0.502 |  | **< 0.001** | **< 0.001** | **< 0.001** | 1.000 | 0.655 | 0.975 |  | **< 0.001** | **< 0.001** | **< 0.001** | 1.000 | 1.000 | 1.000 |
| Gaiella | **< 0.001** | **< 0.001** | **0.005** | **0.032** | 1.000 | **< 0.001** |  | **< 0.001** | **< 0.001** | **< 0.001** | **< 0.001** | 1.000 | **< 0.001** |  | **0.012** | **< 0.001** | **0.003** | **< 0.001** | 1.000 | **< 0.001** |
| Geminicoccus | 0.109 | **< 0.001** | **< 0.001** | **0.008** | 1.000 | 0.406 |  | 1.000 | **< 0.001** | 1.000 | **< 0.001** | 0.177 | 0.442 |  | 1.000 | **0.004** | 1.000 | **< 0.001** | 0.401 | 1.000 |
| Gemmata | 1.000 | 0.220 | 1.000 | **0.022** | 1.000 | 0.538 |  | 1.000 | 1.000 | 0.935 | 0.969 | 1.000 | 0.236 |  | 1.000 | 0.067 | 1.000 | 0.069 | 1.000 | 0.774 |
| Gemmatimonas | **< 0.001** | 1.000 | **< 0.001** | **< 0.001** | **< 0.001** | **< 0.001** |  | **< 0.001** | 0.451 | **< 0.001** | 0.621 | **< 0.001** | **< 0.001** |  | 1.000 | 0.371 | **< 0.001** | 0.628 | **< 0.001** | **< 0.001** |
| Geobacter | 1.000 | **< 0.001** | **0.003** | **< 0.001** | **< 0.001** | **0.007** |  | 1.000 | **< 0.001** | **< 0.001** | **< 0.001** | **< 0.001** | 0.168 |  | 1.000 | **< 0.001** | **< 0.001** | **< 0.001** | **< 0.001** | 1.000 |
| Georgfuchsia | 1.000 | 1.000 | 1.000 | 1.000 | 1.000 | 1.000 |  | 1.000 | 1.000 | 0.585 | 1.000 | 1.000 | 1.000 |  | 0.712 | 0.316 | 0.919 | 1.000 | 1.000 | 1.000 |
| Geothrix | 1.000 | 0.561 | 1.000 | 1.000 | 1.000 | 0.125 |  | 1.000 | 1.000 | 1.000 | 1.000 | 1.000 | 1.000 |  | 1.000 | 1.000 | 0.635 | 1.000 | 0.763 | **0.014** |
| Gp1 (Acidobactetria) | 1.000 | **0.002** | 1.000 | 0.971 | 1.000 | **0.012** |  | **< 0.001** | **< 0.001** | **0.004** | **0.015** | 1.000 | **0.002** |  | 0.956 | **< 0.001** | 1.000 | **< 0.001** | 1.000 | **< 0.001** |
| Gp2 (Acidobactetria) | 1.000 | **0.028** | **0.014** | 1.000 | 0.741 | 1.000 |  | 0.510 | **< 0.001** | **< 0.001** | **0.024** | 1.000 | 1.000 |  | 1.000 | **0.003** | 0.285 | **< 0.001** | **0.002** | 1.000 |
| Gp3 (Acidobactetria) | 1.000 | **< 0.001** | **< 0.001** | **< 0.001** | **< 0.001** | **0.017** |  | 1.000 | **< 0.001** | **< 0.001** | **< 0.001** | **< 0.001** | **< 0.001** |  | 1.000 | **< 0.001** | **0.034** | **< 0.001** | **0.001** | **< 0.001** |
| Gp4 (Acidobactetria) | 1.000 | 1.000 | **0.001** | 1.000 | **< 0.001** | 0.320 |  | 1.000 | 0.417 | **< 0.001** | 1.000 | **< 0.001** | 0.438 |  | 1.000 | 1.000 | 1.000 | 1.000 | 1.000 | 1.000 |
| Gp5 (Acidobactetria) | 1.000 | 0.561 | 1.000 | 1.000 | 1.000 | 1.000 |  | 1.000 | 0.276 | 0.125 | 1.000 | 1.000 | 1.000 |  | 1.000 | 0.725 | 1.000 | 0.301 | 1.000 | 1.000 |
| Gp6 (Acidobactetria) | 1.000 | 1.000 | 1.000 | 1.000 | 1.000 | 1.000 |  | 0.344 | **< 0.001** | **0.003** | 0.236 | 1.000 | 1.000 |  | 1.000 | 1.000 | 0.183 | 0.252 | 1.000 | **< 0.001** |
| Gp7 (Acidobactetria) | 0.875 | 1.000 | 0.382 | 0.971 | 1.000 | 0.478 |  | **0.018** | **< 0.001** | **< 0.001** | 1.000 | 0.779 | 1.000 |  | 1.000 | 0.105 | 0.250 | 1.000 | 1.000 | 1.000 |
| Gp10 (Acidobactetria) | **0.007** | **< 0.001** | 0.472 | **< 0.001** | 1.000 | **< 0.001** |  | 1.000 | **< 0.001** | 1.000 | **< 0.001** | 0.642 | **< 0.001** |  | 0.214 | **< 0.001** | 1.000 | **0.008** | 1.000 | **< 0.001** |
| Gp11 (Acidobactetria) | 1.000 | **< 0.001** | **0.001** | **< 0.001** | **< 0.001** | 1.000 |  | 1.000 | 0.156 | **0.049** | 1.000 | 1.000 | 1.000 |  | 1.000 | **0.002** | 0.158 | **0.002** | 0.221 | 1.000 |
| Gp13 (Acidobactetria) | 1.000 | **< 0.001** | **0.007** | **0.001** | **0.006** | 1.000 |  | 0.382 | **< 0.001** | **< 0.001** | 0.055 | 1.000 | 1.000 |  | 1.000 | **0.007** | **0.007** | 0.123 | 0.113 | 1.000 |
| Gp16 (Acidobactetria) | **0.015** | **< 0.001** | **< 0.001** | 0.055 | 0.565 | 1.000 |  | **< 0.001** | **< 0.001** | **< 0.001** | **0.035** | 0.255 | 1.000 |  | 0.529 | **< 0.001** | **< 0.001** | **< 0.001** | 0.664 | 1.000 |
| Gp17 (Acidobactetria) | 0.064 | 1.000 | **< 0.001** | **0.004** | **< 0.001** | **< 0.001** |  | 0.067 | 1.000 | **< 0.001** | **0.002** | **< 0.001** | **< 0.001** |  | 1.000 | 1.000 | **< 0.001** | 0.123 | **< 0.001** | **< 0.001** |
| Gp22 (Acidobactetria) | 1.000 | 1.000 | 1.000 | 1.000 | 0.079 | 0.052 |  | 1.000 | **0.013** | 1.000 | **0.048** | 1.000 | **0.039** |  | 1.000 | 1.000 | 1.000 | 0.567 | 1.000 | 0.171 |
| Gp25 (Acidobactetria) | 1.000 | 1.000 | **0.016** | 1.000 | 0.174 | **0.001** |  | 0.411 | 0.276 | **< 0.001** | 1.000 | 0.063 | 0.083 |  | 1.000 | 1.000 | 1.000 | 1.000 | 1.000 | 1.000 |
| GpI (Cyanobacteria) | **0.001** | **< 0.001** | 0.680 | **0.044** | 0.905 | **< 0.001** |  | **< 0.001** | **< 0.001** | **< 0.001** | **< 0.001** | 1.000 | **< 0.001** |  | **< 0.001** | **< 0.001** | **< 0.001** | **< 0.001** | 1.000 | **< 0.001** |
| Granulicella | 1.000 | 1.000 | 1.000 | 1.000 | 1.000 | 1.000 |  | 1.000 | **0.036** | **0.015** | 0.101 | 0.051 | 1.000 |  | 1.000 | 1.000 | 1.000 | 1.000 | 1.000 | 1.000 |
| Haliea | 0.426 | 1.000 | 1.000 | 1.000 | 1.000 | 1.000 |  | 1.000 | 1.000 | 1.000 | 1.000 | 0.620 | 1.000 |  | 1.000 | 1.000 | 1.000 | 1.000 | 1.000 | 1.000 |
| Hamadaea | 1.000 | 1.000 | 0.194 | 1.000 | 0.075 | 0.362 |  | 1.000 | 1.000 | 1.000 | 1.000 | 1.000 | 1.000 |  | 1.000 | 1.000 | 1.000 | 1.000 | 0.781 | 1.000 |
| Herbaspirillum | 1.000 | 1.000 | 0.522 | 1.000 | 1.000 | 0.092 |  | **< 0.001** | **< 0.001** | **< 0.001** | **< 0.001** | **< 0.001** | **< 0.001** |  | 1.000 | 0.076 | 1.000 | **0.002** | 1.000 | 0.324 |
| Hyphomicrobium | **< 0.001** | **< 0.001** | **< 0.001** | 1.000 | 1.000 | 1.000 |  | **< 0.001** | **< 0.001** | **< 0.001** | 1.000 | 1.000 | 1.000 |  | **0.001** | **< 0.001** | 0.185 | 0.389 | 1.000 | **0.001** |
| Ilumatobacter | 1.000 | 0.195 | 0.342 | 1.000 | 1.000 | 1.000 |  | 1.000 | 0.767 | 1.000 | 1.000 | 1.000 | 1.000 |  | 1.000 | 1.000 | 1.000 | 1.000 | 1.000 | 1.000 |
| Janibacter | 0.339 | **< 0.001** | **0.003** | **< 0.001** | 1.000 | **0.033** |  | **< 0.001** | **< 0.001** | **< 0.001** | **< 0.001** | 0.992 | 0.822 |  | **0.034** | **< 0.001** | **< 0.001** | **0.010** | 1.000 | 1.000 |
| Kaistia | 0.257 | 1.000 | 1.000 | 1.000 | **0.005** | 1.000 |  | 1.000 | 0.570 | **0.041** | 1.000 | 0.120 | 1.000 |  | 0.456 | 1.000 | 1.000 | 0.790 | **0.003** | 1.000 |
| Kineococcus | 0.471 | **0.020** | 1.000 | 1.000 | 1.000 | 0.335 |  | 0.898 | **0.032** | 1.000 | 1.000 | 1.000 | 0.957 |  | 1.000 | 1.000 | 1.000 | 1.000 | 1.000 | 1.000 |
| Kineosporia | **< 0.001** | **< 0.001** | **< 0.001** | **0.002** | **< 0.001** | **< 0.001** |  | **0.003** | **< 0.001** | **< 0.001** | **< 0.001** | **< 0.001** | **< 0.001** |  | **< 0.001** | **< 0.001** | **< 0.001** | **< 0.001** | **< 0.001** | **< 0.001** |
| Kofleria | 1.000 | 1.000 | 0.411 | 1.000 | 1.000 | 1.000 |  | 1.000 | 0.082 | **0.029** | 1.000 | 0.651 | 1.000 |  | 1.000 | 1.000 | 1.000 | 1.000 | 1.000 | 1.000 |
| Kribbella | 0.159 | **< 0.001** | 0.522 | 0.538 | **< 0.001** | **< 0.001** |  | **0.013** | **< 0.001** | 1.000 | **0.004** | **< 0.001** | **< 0.001** |  | 0.119 | **< 0.001** | 0.053 | 0.384 | **< 0.001** | **< 0.001** |
| Ktedonobacter | 1.000 | 1.000 | 1.000 | 0.228 | 1.000 | 1.000 |  | **0.003** | **< 0.001** | **< 0.001** | 1.000 | 1.000 | 1.000 |  | 1.000 | 1.000 | 1.000 | 1.000 | 1.000 | 1.000 |
| Kutzneria | 1.000 | 1.000 | 0.410 | 1.000 | 0.679 | 1.000 |  | 1.000 | **< 0.001** | **< 0.001** | **< 0.001** | **0.006** | 1.000 |  | **0.009** | 0.155 | **0.001** | 1.000 | 1.000 | 1.000 |
| Labrys | 1.000 | 1.000 | 0.104 | 1.000 | 1.000 | 1.000 |  | 1.000 | 0.101 | 1.000 | 1.000 | 1.000 | 1.000 |  | 1.000 | **0.003** | 1.000 | 0.389 | 1.000 | **0.008** |
| Legionella | **0.015** | 1.000 | 0.321 | 0.413 | **< 0.001** | **0.019** |  | 1.000 | 1.000 | 0.468 | 1.000 | 0.164 | **0.006** |  | 1.000 | 1.000 | 1.000 | 1.000 | 1.000 | 1.000 |
| Leucobacter | 1.000 | 1.000 | 1.000 | 1.000 | 1.000 | 1.000 |  | 1.000 | 1.000 | 1.000 | 1.000 | 1.000 | 1.000 |  | 1.000 | 1.000 | 1.000 | 1.000 | 0.149 | 1.000 |
| Longispora | 0.340 | 1.000 | 1.000 | 1.000 | **0.006** | 0.097 |  | **0.040** | 1.000 | 1.000 | 1.000 | 0.056 | 1.000 |  | 1.000 | 0.721 | 0.482 | 1.000 | 0.253 | **0.002** |
| Luteibacter | 1.000 | **0.037** | **0.030** | 0.127 | 0.072 | 1.000 |  | **0.030** | **0.022** | 1.000 | 1.000 | 1.000 | 1.000 |  | 1.000 | 0.344 | 1.000 | 1.000 | 1.000 | 1.000 |
| Luteolibacter | 1.000 | 0.296 | 1.000 | 1.000 | 1.000 | 1.000 |  | 1.000 | 1.000 | 1.000 | 1.000 | 1.000 | 1.000 |  | 1.000 | 1.000 | 1.000 | 1.000 | 1.000 | 1.000 |
| Lysinibacillus | 1.000 | **< 0.001** | 0.146 | **0.001** | 1.000 | 0.351 |  | 0.194 | **< 0.001** | **0.007** | 0.065 | 1.000 | 0.529 |  | 0.465 | **0.002** | 0.392 | 1.000 | 1.000 | 1.000 |
| Lysobacter | **0.022** | **0.002** | **< 0.001** | 1.000 | 1.000 | 1.000 |  | 0.670 | **< 0.001** | 0.161 | 1.000 | 1.000 | 1.000 |  | 1.000 | 0.898 | 1.000 | 1.000 | 1.000 | 1.000 |
| Marmoricola | 1.000 | 0.099 | 1.000 | **0.006** | 1.000 | 0.379 |  | 1.000 | 1.000 | 1.000 | 1.000 | 1.000 | 1.000 |  | 1.000 | **< 0.001** | **0.035** | **0.002** | 0.297 | 1.000 |
| Massilia | 1.000 | **0.022** | **0.004** | 1.000 | 1.000 | 1.000 |  | 1.000 | 0.575 | 0.275 | 1.000 | 1.000 | 1.000 |  | 0.977 | **0.002** | **< 0.001** | 1.000 | 0.763 | 1.000 |
| Mesorhizobium | **0.009** | 1.000 | 0.083 | 1.000 | **< 0.001** | **< 0.001** |  | 0.606 | 1.000 | **< 0.001** | **0.002** | **< 0.001** | **0.011** |  | 1.000 | **0.025** | 1.000 | **0.019** | 0.947 | 1.000 |
| Methylobacterium | 1.000 | 1.000 | 1.000 | 1.000 | 1.000 | 1.000 |  | **< 0.001** | **< 0.001** | **< 0.001** | 1.000 | 1.000 | 1.000 |  | 1.000 | 1.000 | 1.000 | 1.000 | 1.000 | 1.000 |
| Methylophilus | 1.000 | 1.000 | 1.000 | 1.000 | 1.000 | 1.000 |  | **0.005** | 0.051 | **< 0.001** | 1.000 | 1.000 | 1.000 |  | 0.888 | **0.004** | 0.059 | 1.000 | 1.000 | 1.000 |
| Microbacterium | 1.000 | 1.000 | 1.000 | 1.000 | 1.000 | 1.000 |  | 1.000 | 1.000 | 1.000 | 1.000 | 1.000 | 1.000 |  | 1.000 | 1.000 | 1.000 | 1.000 | 1.000 | 1.000 |
| Micromonospora | 0.075 | **< 0.001** | **< 0.001** | 1.000 | **0.010** | 1.000 |  | 0.835 | **0.003** | **0.001** | 1.000 | 1.000 | 1.000 |  | 1.000 | 1.000 | 0.909 | 1.000 | 1.000 | 1.000 |
| Microvirga | 1.000 | **< 0.001** | **0.005** | **< 0.001** | 0.492 | **< 0.001** |  | 1.000 | **< 0.001** | **< 0.001** | **< 0.001** | 0.340 | 0.089 |  | 0.529 | **< 0.001** | **0.007** | **< 0.001** | 1.000 | **< 0.001** |
| Modestobacter | 1.000 | 0.060 | 1.000 | **0.008** | 1.000 | **< 0.001** |  | 1.000 | **0.020** | 1.000 | **0.024** | 1.000 | **0.001** |  | 1.000 | 1.000 | 1.000 | 0.092 | 1.000 | 0.076 |
| Mucilaginibacter | **< 0.001** | **< 0.001** | **< 0.001** | 1.000 | 1.000 | 1.000 |  | **0.046** | **0.002** | **0.005** | 1.000 | 1.000 | 1.000 |  | 0.063 | **< 0.001** | 0.069 | 1.000 | 1.000 | 1.000 |
| Mycobacterium | **< 0.001** | **< 0.001** | **< 0.001** | **< 0.001** | 1.000 | **< 0.001** |  | **< 0.001** | **< 0.001** | **< 0.001** | **< 0.001** | 1.000 | **< 0.001** |  | **< 0.001** | **< 0.001** | **< 0.001** | **< 0.001** | 1.000 | **< 0.001** |
| Myxococcus | 1.000 | 1.000 | 0.078 | 1.000 | **0.012** | **0.002** |  | **0.001** | **< 0.001** | 0.585 | 0.714 | 0.259 | **< 0.001** |  | 1.000 | 0.577 | 0.788 | 1.000 | 0.257 | **0.002** |
| Nakamurella | **< 0.001** | **< 0.001** | **< 0.001** | **< 0.001** | 0.113 | **< 0.001** |  | **< 0.001** | **< 0.001** | **< 0.001** | **< 0.001** | 1.000 | **< 0.001** |  | **< 0.001** | **< 0.001** | **< 0.001** | **< 0.001** | 1.000 | **< 0.001** |
| Niastella | **< 0.001** | **< 0.001** | **< 0.001** | 1.000 | 1.000 | 1.000 |  | **< 0.001** | **< 0.001** | **< 0.001** | 1.000 | 1.000 | 1.000 |  | **0.007** | **< 0.001** | **< 0.001** | 1.000 | 1.000 | 1.000 |
| Nitrosospira | **< 0.001** | **< 0.001** | 0.178 | **< 0.001** | **< 0.001** | **< 0.001** |  | **< 0.001** | **< 0.001** | **< 0.001** | **< 0.001** | **< 0.001** | **< 0.001** |  | **< 0.001** | **< 0.001** | **< 0.001** | **< 0.001** | **< 0.001** | **< 0.001** |
| Nitrospira | 1.000 | **< 0.001** | **< 0.001** | **0.008** | **0.005** | 1.000 |  | **0.014** | **< 0.001** | **< 0.001** | **< 0.001** | **0.014** | 1.000 |  | **0.007** | **< 0.001** | 0.599 | **< 0.001** | 1.000 | **< 0.001** |
| Nocardia | 1.000 | 1.000 | 1.000 | 1.000 | 1.000 | 1.000 |  | 1.000 | 1.000 | 1.000 | 1.000 | 1.000 | 1.000 |  | 1.000 | 1.000 | 1.000 | 1.000 | 1.000 | 1.000 |
| Nocardioides | 1.000 | **< 0.001** | **0.002** | 0.164 | 0.626 | 1.000 |  | 0.660 | **< 0.001** | **< 0.001** | 0.158 | 0.255 | 1.000 |  | 1.000 | **< 0.001** | **0.001** | 0.061 | 1.000 | 1.000 |
| Nonomuraea | 1.000 | 1.000 | 1.000 | 1.000 | 1.000 | 1.000 |  | 0.348 | 1.000 | 1.000 | 1.000 | 1.000 | 1.000 |  | 1.000 | 1.000 | 1.000 | 1.000 | 1.000 | 1.000 |
| Noviherbaspirillum | 0.290 | 1.000 | 1.000 | **0.005** | 1.000 | 0.061 |  | 1.000 | 0.264 | 1.000 | 0.603 | 1.000 | 1.000 |  | 1.000 | 0.868 | 1.000 | **0.002** | 1.000 | 0.736 |
| Novosphingobium | **< 0.001** | **< 0.001** | 1.000 | 1.000 | **< 0.001** | **< 0.001** |  | **< 0.001** | **< 0.001** | 1.000 | 1.000 | **< 0.001** | **< 0.001** |  | **< 0.001** | **< 0.001** | 1.000 | 1.000 | **< 0.001** | **< 0.001** |
| Ohtaekwangia | **< 0.001** | **< 0.001** | **< 0.001** | **0.001** | **0.001** | 1.000 |  | 0.344 | **< 0.001** | **< 0.001** | **< 0.001** | **< 0.001** | 1.000 |  | 0.075 | **< 0.001** | **< 0.001** | **< 0.001** | **< 0.001** | 0.351 |
| Opitutus | **0.001** | **< 0.001** | **< 0.001** | 1.000 | 1.000 | 1.000 |  | 1.000 | **0.024** | 0.274 | 0.625 | 1.000 | 1.000 |  | **0.029** | 0.067 | **< 0.001** | 1.000 | 1.000 | 1.000 |
| Panacagrimonas | 0.528 | 1.000 | 0.908 | 1.000 | **0.002** | 0.144 |  | 1.000 | **0.012** | **< 0.001** | **0.048** | **0.002** | 1.000 |  | 0.497 | 1.000 | 1.000 | 1.000 | **0.002** | 0.324 |
| Pedobacter | 0.931 | 1.000 | 1.000 | 1.000 | 1.000 | 1.000 |  | 1.000 | 1.000 | 1.000 | 1.000 | 1.000 | 1.000 |  | 1.000 | 1.000 | 1.000 | 1.000 | 1.000 | 1.000 |
| Pedomicrobium | **< 0.001** | **< 0.001** | **< 0.001** | 1.000 | 1.000 | 0.483 |  | **< 0.001** | **< 0.001** | **< 0.001** | 0.160 | 1.000 | 1.000 |  | 0.643 | **< 0.001** | 1.000 | 1.000 | 1.000 | **0.006** |
| Phaselicystis | 1.000 | 1.000 | 1.000 | 1.000 | 1.000 | 1.000 |  | 1.000 | 1.000 | 1.000 | 1.000 | 1.000 | 1.000 |  | 1.000 | 1.000 | 1.000 | 1.000 | 1.000 | 1.000 |
| Phenylobacterium | **< 0.001** | **< 0.001** | **0.002** | 1.000 | 1.000 | 1.000 |  | 0.066 | **0.005** | 1.000 | 1.000 | 0.655 | 0.166 |  | 1.000 | 0.075 | 1.000 | 1.000 | 1.000 | 1.000 |
| Phycicoccus | 1.000 | **< 0.001** | **< 0.001** | **< 0.001** | **< 0.001** | 1.000 |  | 1.000 | **< 0.001** | **< 0.001** | **< 0.001** | **< 0.001** | 1.000 |  | 1.000 | **< 0.001** | **< 0.001** | **< 0.001** | **0.011** | 1.000 |
| Phyllobacterium | 0.343 | 1.000 | 1.000 | 0.085 | 0.219 | 1.000 |  | 1.000 | **0.003** | **< 0.001** | 1.000 | **0.004** | 1.000 |  | 1.000 | 1.000 | 1.000 | 1.000 | 1.000 | 1.000 |
| Pirellula | 1.000 | **0.032** | 1.000 | 1.000 | 1.000 | 0.849 |  | 1.000 | 1.000 | 1.000 | 1.000 | 1.000 | 1.000 |  | 1.000 | **0.042** | 0.431 | 1.000 | 1.000 | 1.000 |
| Planctomyces | 1.000 | **0.048** | 1.000 | 1.000 | 0.255 | **< 0.001** |  | 1.000 | 0.724 | 0.558 | 1.000 | 0.395 | **0.001** |  | 1.000 | 0.091 | 1.000 | 1.000 | 1.000 | **0.037** |
| Planifilum | 0.243 | 0.553 | 1.000 | 1.000 | 0.177 | 0.602 |  | 1.000 | 0.396 | 0.822 | 0.925 | 1.000 | 1.000 |  | 1.000 | 1.000 | 1.000 | 1.000 | 1.000 | 1.000 |
| Polaromonas | 0.729 | 0.805 | 0.054 | 1.000 | 1.000 | 1.000 |  | 0.149 | **0.021** | 0.253 | 1.000 | 1.000 | 1.000 |  | **< 0.001** | **< 0.001** | **< 0.001** | 1.000 | 1.000 | 1.000 |
| Porphyrobacter | 1.000 | 0.261 | 1.000 | 0.270 | 1.000 | 1.000 |  | 1.000 | **0.001** | 1.000 | **< 0.001** | 1.000 | **< 0.001** |  | 1.000 | 0.160 | 1.000 | 1.000 | 1.000 | 1.000 |
| Pseudolabrys | 1.000 | 1.000 | 1.000 | 1.000 | 1.000 | 1.000 |  | 1.000 | 1.000 | 1.000 | 1.000 | 1.000 | 1.000 |  | 1.000 | 1.000 | 1.000 | 1.000 | 1.000 | 1.000 |
| Pseudomonas | 0.159 | **< 0.001** | 0.080 | 1.000 | 1.000 | 1.000 |  | 1.000 | **0.002** | **< 0.001** | 0.992 | 0.342 | 1.000 |  | **< 0.001** | **< 0.001** | **0.001** | 1.000 | 1.000 | 1.000 |
| Pseudoxanthomonas | **0.015** | 1.000 | 1.000 | 1.000 | 0.085 | 1.000 |  | 1.000 | **0.037** | 1.000 | 0.760 | 1.000 | 1.000 |  | 1.000 | 0.120 | 1.000 | 1.000 | 1.000 | 0.582 |
| Pyxidicoccus | 1.000 | 0.312 | 1.000 | 0.259 | 1.000 | 1.000 |  | 0.606 | 1.000 | 1.000 | 1.000 | 0.377 | 1.000 |  | 1.000 | 1.000 | 1.000 | 1.000 | 1.000 | 1.000 |
| Rhizobium | **< 0.001** | **< 0.001** | **< 0.001** | 1.000 | 1.000 | 1.000 |  | **< 0.001** | **< 0.001** | **< 0.001** | 1.000 | 1.000 | 1.000 |  | **0.014** | **< 0.001** | **< 0.001** | 1.000 | **< 0.001** | 0.324 |
| Rhizomicrobium | 1.000 | 1.000 | 1.000 | 1.000 | 1.000 | 1.000 |  | 1.000 | 1.000 | 1.000 | 1.000 | 1.000 | 1.000 |  | 0.562 | 0.925 | 1.000 | 1.000 | 1.000 | 1.000 |
| Rhodanobacter | 1.000 | 0.088 | 1.000 | 0.423 | 1.000 | 1.000 |  | 1.000 | 1.000 | 1.000 | 1.000 | 1.000 | 1.000 |  | 1.000 | 1.000 | 1.000 | 1.000 | 1.000 | 1.000 |
| Rhodococcus | **< 0.001** | **< 0.001** | 0.913 | 1.000 | 0.174 | 0.813 |  | 1.000 | **0.024** | **< 0.001** | 0.340 | **< 0.001** | 0.321 |  | **< 0.001** | **< 0.001** | 0.205 | 1.000 | 0.701 | **< 0.001** |
| Rhodopila | 1.000 | 1.000 | 1.000 | 1.000 | 1.000 | 1.000 |  | 1.000 | 1.000 | 1.000 | 1.000 | 1.000 | 1.000 |  | 1.000 | 1.000 | 1.000 | 1.000 | 1.000 | 1.000 |
| Roseomonas | 1.000 | **< 0.001** | **< 0.001** | **< 0.001** | **0.008** | **0.044** |  | **< 0.001** | **< 0.001** | **< 0.001** | **< 0.001** | 0.255 | 0.140 |  | 1.000 | **< 0.001** | **< 0.001** | **< 0.001** | 0.062 | 0.113 |
| Rummeliibacillus | 1.000 | 0.100 | 1.000 | 1.000 | 1.000 | 1.000 |  | 0.606 | 0.156 | **0.028** | 1.000 | 1.000 | 1.000 |  | 1.000 | 1.000 | 1.000 | 1.000 | 1.000 | 1.000 |
| Saccharopolyspora | 0.070 | 0.341 | **0.020** | 1.000 | 1.000 | 1.000 |  | 1.000 | 1.000 | 1.000 | 0.062 | 0.564 | 1.000 |  | 1.000 | 0.107 | **0.012** | 1.000 | 1.000 | 1.000 |
| Sanguibacter | 1.000 | 1.000 | 1.000 | 1.000 | 1.000 | 1.000 |  | 1.000 | 1.000 | 1.000 | 1.000 | 1.000 | 1.000 |  | 1.000 | 1.000 | 1.000 | 1.000 | 1.000 | 1.000 |
| Schlesneria | 0.253 | **0.013** | 1.000 | 1.000 | 1.000 | 0.478 |  | 0.852 | 1.000 | 1.000 | 1.000 | 1.000 | 1.000 |  | **0.045** | **0.048** | 1.000 | 1.000 | 0.601 | 0.934 |
| Sediminibacterium | 1.000 | 0.080 | 1.000 | 1.000 | 1.000 | 0.420 |  | 1.000 | 1.000 | 1.000 | 1.000 | 1.000 | 1.000 |  | 1.000 | 1.000 | 1.000 | 1.000 | 1.000 | 1.000 |
| Segetibacter | 0.451 | **< 0.001** | **0.004** | 0.209 | 1.000 | 1.000 |  | 1.000 | **< 0.001** | **0.009** | **0.003** | 1.000 | 0.673 |  | 1.000 | **0.033** | 0.919 | 0.886 | 1.000 | 1.000 |
| Skermanella | **0.001** | **< 0.001** | **0.026** | **< 0.001** | 1.000 | **< 0.001** |  | 1.000 | **< 0.001** | 0.749 | **< 0.001** | 1.000 | **< 0.001** |  | 0.571 | **< 0.001** | 0.720 | **< 0.001** | 1.000 | **< 0.001** |
| Solirubrobacter | **< 0.001** | **< 0.001** | 1.000 | **0.004** | **0.001** | **< 0.001** |  | **< 0.001** | **< 0.001** | 1.000 | 0.062 | **0.011** | **< 0.001** |  | 0.182 | **< 0.001** | 1.000 | 0.308 | 1.000 | **< 0.001** |
| Solitalea | 1.000 | 1.000 | 0.161 | 1.000 | 0.515 | 1.000 |  | 1.000 | 1.000 | **0.041** | 1.000 | 0.140 | 1.000 |  | 1.000 | 1.000 | **0.002** | 1.000 | 0.701 | 0.055 |
| Sorangium | 1.000 | 0.051 | 1.000 | 1.000 | 1.000 | **0.046** |  | 1.000 | 1.000 | 1.000 | 0.969 | 1.000 | 1.000 |  | 0.182 | **< 0.001** | 1.000 | 1.000 | **< 0.001** | **< 0.001** |
| Sphingobium | **< 0.001** | 0.068 | 0.069 | 0.749 | 0.655 | 1.000 |  | 0.060 | 0.112 | 0.101 | 1.000 | 1.000 | 1.000 |  | 0.340 | 0.308 | 0.067 | 1.000 | 1.000 | 1.000 |
| Sphingomonas | **< 0.001** | **< 0.001** | 1.000 | 0.547 | **< 0.001** | **< 0.001** |  | **0.030** | **< 0.001** | **0.018** | 0.853 | **< 0.001** | **< 0.001** |  | 0.369 | **< 0.001** | 1.000 | 0.359 | **< 0.001** | **< 0.001** |
| Sphingopyxis | 1.000 | 1.000 | 1.000 | 1.000 | 0.250 | 0.352 |  | 1.000 | 1.000 | 0.711 | 1.000 | 1.000 | 1.000 |  | **0.025** | **0.024** | 1.000 | 1.000 | 0.199 | 0.324 |
| Stenotrophomonas | **0.002** | **0.001** | **0.009** | 1.000 | 1.000 | 1.000 |  | 1.000 | 0.971 | 0.087 | 1.000 | 0.502 | 1.000 |  | 1.000 | 1.000 | 1.000 | 1.000 | 1.000 | 1.000 |
| Steroidobacter | 1.000 | **< 0.001** | **< 0.001** | **< 0.001** | **< 0.001** | **0.010** |  | 0.606 | 1.000 | **< 0.001** | **0.027** | **< 0.001** | **< 0.001** |  | 1.000 | **0.002** | **< 0.001** | **0.005** | **< 0.001** | **< 0.001** |
| Streptomyces | **< 0.001** | **< 0.001** | **< 0.001** | 1.000 | 1.000 | 1.000 |  | 0.588 | **0.001** | 0.062 | 1.000 | 1.000 | 1.000 |  | 1.000 | 1.000 | 1.000 | 1.000 | 1.000 | 1.000 |
| Streptosporangium | 1.000 | 1.000 | 1.000 | 1.000 | 1.000 | 1.000 |  | **0.031** | 0.158 | **0.040** | 1.000 | 1.000 | 1.000 |  | 1.000 | 0.699 | 1.000 | 1.000 | 1.000 | 1.000 |
| Symbiobacterium | 0.159 | **0.017** | 1.000 | 1.000 | 1.000 | 1.000 |  | 0.476 | 0.780 | 1.000 | 1.000 | 1.000 | 1.000 |  | 1.000 | 1.000 | 1.000 | 1.000 | 1.000 | 1.000 |
| Terrabacter | 1.000 | **< 0.001** | **< 0.001** | **< 0.001** | **0.011** | **0.002** |  | 1.000 | **< 0.001** | **< 0.001** | **< 0.001** | **0.005** | **0.002** |  | 1.000 | **< 0.001** | **< 0.001** | **< 0.001** | **0.005** | 0.150 |
| Terrimonas | 1.000 | 1.000 | 0.146 | 1.000 | **0.002** | **0.002** |  | 1.000 | **0.030** | **< 0.001** | 1.000 | 0.156 | 1.000 |  | 1.000 | 1.000 | 1.000 | 1.000 | 1.000 | 1.000 |
| Thermoactinomyces | **0.004** | **< 0.001** | 1.000 | 1.000 | **0.040** | **0.007** |  | 1.000 | 1.000 | 1.000 | 1.000 | 1.000 | 0.095 |  | 1.000 | 1.000 | 1.000 | 1.000 | 1.000 | 1.000 |
| Thermoflavimicrobium | **0.028** | **< 0.001** | 0.353 | 1.000 | 1.000 | 0.467 |  | 1.000 | 1.000 | 1.000 | 1.000 | 0.539 | 0.168 |  | 1.000 | 0.174 | 1.000 | 1.000 | 1.000 | 1.000 |
| Thiobacillus | 1.000 | 0.684 | 1.000 | 1.000 | 1.000 | 1.000 |  | 1.000 | 1.000 | 1.000 | 1.000 | 1.000 | 1.000 |  | 0.757 | 0.104 | 0.092 | 1.000 | 1.000 | 1.000 |
| Tumebacillus | **< 0.001** | **< 0.001** | **< 0.001** | 1.000 | **< 0.001** | **0.006** |  | **< 0.001** | **< 0.001** | **< 0.001** | 1.000 | **0.019** | 1.000 |  | **< 0.001** | **< 0.001** | 0.919 | 1.000 | 0.092 | **0.006** |
| Turicibacter | 0.135 | **< 0.001** | **0.045** | 0.308 | 1.000 | 0.478 |  | **0.021** | 0.470 | 1.000 | **< 0.001** | **0.001** | 1.000 |  | **0.027** | 0.525 | 1.000 | 1.000 | 0.225 | 1.000 |
| Undibacterium | 1.000 | 0.187 | **0.005** | 1.000 | 0.665 | 1.000 |  | 1.000 | **0.008** | 0.095 | 1.000 | 1.000 | 1.000 |  | 0.235 | **< 0.001** | **< 0.001** | 0.969 | 0.783 | 1.000 |
| Ureibacillus | 0.109 | 0.721 | 1.000 | 1.000 | 0.070 | 0.756 |  | 0.670 | 0.156 | 1.000 | 1.000 | 0.381 | 0.140 |  | 0.497 | 0.388 | 1.000 | 1.000 | 1.000 | 1.000 |
| Vampirovibrio | 1.000 | 1.000 | 1.000 | 1.000 | 1.000 | 1.000 |  | **0.038** | 0.108 | 0.558 | 1.000 | 1.000 | 1.000 |  | 1.000 | 1.000 | 1.000 | 1.000 | 1.000 | 1.000 |
| Variovorax | 1.000 | **0.001** | **< 0.001** | **0.004** | **< 0.001** | **< 0.001** |  | 1.000 | 0.658 | **< 0.001** | 0.969 | **< 0.001** | **< 0.001** |  | 0.127 | 0.579 | **< 0.001** | 1.000 | 1.000 | 0.324 |
| Virgisporangium | 1.000 | 1.000 | **0.009** | 0.276 | **< 0.001** | 0.299 |  | 1.000 | 0.997 | **< 0.001** | 1.000 | **< 0.001** | **0.003** |  | 1.000 | **0.003** | **< 0.001** | **0.050** | **< 0.001** | 0.392 |
| Williamsia | 1.000 | 1.000 | 1.000 | 1.000 | 1.000 | 1.000 |  | 1.000 | 1.000 | 1.000 | 1.000 | 1.000 | 1.000 |  | 1.000 | 1.000 | 1.000 | 1.000 | 1.000 | 1.000 |
| Zavarzinella | 1.000 | **0.049** | 1.000 | 1.000 | 1.000 | **0.033** |  | 1.000 | 1.000 | **0.041** | 1.000 | 1.000 | 1.000 |  | 1.000 | 0.578 | 1.000 | 1.000 | 1.000 | 0.392 |
| Unclassified genera | 1.000 | 1.000 | **< 0.001** | 1.000 | **< 0.001** | **< 0.001** |  | 1.000 | **< 0.001** | 1.000 | **< 0.001** | 1.000 | **< 0.001** |  | 1.000 | **0.026** | **< 0.001** | **< 0.001** | 0.113 | **< 0.001** |

Significant values are given in bold.
