# Supplementary material for: Bacterial Preferences for Specific Soil Particle Size Fractions Revealed by Community Analyses
Source: Front Microbiol. 2018 Feb 23;9:149. doi: 10.3389/fmicb.2018.00149 (PMC5829042; doi:10.3389/fmicb.2018.00149)
Supplement: Supplementary file 14 [file Table14.DOCX]

Table S14 Significance values for archaeal OTUs as in Table S13 but without a sand/POM-NPK sample that the authors considered as outlier

| **OTU** | **UNF** | | | | | |  | **NPK** | | | | | |  | **AM** | | | | | |
| --- | --- | --- | --- | --- | --- | --- | --- | --- | --- | --- | --- | --- | --- | --- | --- | --- | --- | --- | --- | --- |
|  | **Sand/POM - Coarse silt** | **Sand/POM - Fine silt** | **Sand/POM - Clay** | **Coarse silt - Fine silt** | **Coarse silt - Clay** | **Fine silt - Clay** |  | **Sand/POM - Coarse silt** | **Sand/POM - Fine silt** | **Sand/POM - Clay** | **Coarse silt - Fine silt** | **Coarse silt - Clay** | **Fine silt - Clay** |  | **Sand/POM - Coarse silt** | **Sand/POM - Fine silt** | **Sand/POM - Clay** | **Coarse silt - Fine silt** | **Coarse silt - Clay** | **Fine silt - Clay** |
| OTU_120 | 1.000 | 1.000 | 1.000 | 1.000 | 0.305 | 0.290 |  | 1.000 | 1.000 | 1.000 | 1.000 | 1.000 | 1.000 |  | 1.000 | 1.000 | 1.000 | 1.000 | 1.000 | **0.003** |
| OTU_1968 | 1.000 | 1.000 | 1.000 | 1.000 | 1.000 | 1.000 |  | 1.000 | 1.000 | 1.000 | 0.352 | 1.000 | **0.005** |  | 1.000 | 1.000 | 1.000 | 1.000 | 1.000 | 1.000 |
| OTU_2451 | 1.000 | 1.000 | 1.000 | 1.000 | **0.005** | **0.012** |  | 1.000 | 1.000 | 1.000 | 1.000 | 1.000 | 1.000 |  | 1.000 | 1.000 | 1.000 | 1.000 | 1.000 | 1.000 |
| OTU_250 | 1.000 | 0.387 | 1.000 | 1.000 | 1.000 | 1.000 |  | 1.000 | **0.007** | 1.000 | 0.505 | 1.000 | **0.042** |  | 1.000 | 1.000 | 1.000 | 1.000 | 1.000 | 1.000 |
| OTU_270 | 1.000 | 1.000 | 1.000 | 1.000 | 1.000 | 1.000 |  | 1.000 | 0.068 | 0.809 | 1.000 | 1.000 | 1.000 |  | 1.000 | 1.000 | 1.000 | 1.000 | 1.000 | 1.000 |
| OTU_276 | 1.000 | **< 0.001** | 1.000 | **< 0.001** | 1.000 | **< 0.001** |  | 1.000 | **0.007** | 1.000 | **< 0.001** | 1.000 | **< 0.001** |  | 1.000 | **0.004** | 1.000 | 1.000 | 1.000 | **< 0.001** |
| OTU_2833 | 1.000 | 1.000 | 1.000 | 1.000 | 1.000 | 1.000 |  | 1.000 | 1.000 | 1.000 | 1.000 | 0.922 | 1.000 |  | 1.000 | 1.000 | 1.000 | 1.000 | 1.000 | 1.000 |
| OTU_2962 | 1.000 | 1.000 | 1.000 | 1.000 | 1.000 | 1.000 |  | 1.000 | 1.000 | 1.000 | 1.000 | 1.000 | 1.000 |  | 1.000 | 1.000 | 1.000 | 1.000 | 1.000 | 1.000 |
| OTU_3605 | 1.000 | 1.000 | 1.000 | 1.000 | 1.000 | 1.000 |  | 1.000 | 1.000 | 1.000 | 1.000 | 1.000 | 1.000 |  | 1.000 | 1.000 | 1.000 | 1.000 | 1.000 | 1.000 |
| OTU_3680 | 1.000 | 1.000 | 1.000 | 0.325 | 1.000 | 1.000 |  | 1.000 | 1.000 | 1.000 | 1.000 | 1.000 | 1.000 |  | 1.000 | 1.000 | 1.000 | 1.000 | 1.000 | 1.000 |
| OTU_387 | 1.000 | **0.028** | 0.627 | **0.002** | 0.113 | 1.000 |  | 1.000 | 0.231 | 1.000 | 1.000 | 1.000 | 1.000 |  | 1.000 | 1.000 | 1.000 | 1.000 | 1.000 | 1.000 |
| OTU_412 | 1.000 | 1.000 | 1.000 | 1.000 | 1.000 | 1.000 |  | 1.000 | 1.000 | 1.000 | 1.000 | 1.000 | 1.000 |  | 1.000 | 1.000 | 1.000 | 1.000 | 1.000 | 1.000 |
| OTU_4561 | 1.000 | 1.000 | 1.000 | 1.000 | 1.000 | 1.000 |  | 1.000 | 1.000 | 1.000 | 1.000 | 1.000 | 1.000 |  | 1.000 | 1.000 | 1.000 | 1.000 | 1.000 | 1.000 |
| OTU_4703 | 1.000 | 0.828 | 1.000 | 1.000 | 1.000 | 1.000 |  | 1.000 | 1.000 | 1.000 | 1.000 | 0.922 | 1.000 |  | 1.000 | 1.000 | 1.000 | 1.000 | 1.000 | 1.000 |
| OTU_4736 | 1.000 | 1.000 | 1.000 | 1.000 | 1.000 | 1.000 |  | 1.000 | 1.000 | 1.000 | 1.000 | 1.000 | 1.000 |  | 1.000 | 1.000 | 1.000 | 1.000 | 1.000 | 1.000 |
| OTU_50 | 1.000 | 0.578 | 0.133 | 1.000 | 1.000 | 1.000 |  | 1.000 | 1.000 | 1.000 | 1.000 | 1.000 | 1.000 |  | 1.000 | 1.000 | 1.000 | 1.000 | 1.000 | 1.000 |
| OTU_5245 | 1.000 | 1.000 | 1.000 | 1.000 | 1.000 | 1.000 |  | 1.000 | 1.000 | 1.000 | 1.000 | 1.000 | 1.000 |  | 1.000 | 1.000 | 1.000 | 1.000 | 1.000 | 1.000 |
| OTU_5573 | 1.000 | 1.000 | 1.000 | 1.000 | 1.000 | 1.000 |  | 1.000 | 1.000 | 1.000 | 1.000 | 0.922 | 1.000 |  | 1.000 | 1.000 | 1.000 | 1.000 | 1.000 | 1.000 |
| OTU_6174 | 1.000 | 1.000 | 1.000 | 1.000 | 0.114 | 1.000 |  | 1.000 | 1.000 | 1.000 | 1.000 | 1.000 | 1.000 |  | 1.000 | 1.000 | 1.000 | 1.000 | 1.000 | 1.000 |
| OTU_6785 | 1.000 | 1.000 | 1.000 | 1.000 | 1.000 | 1.000 |  | 1.000 | 1.000 | 1.000 | 1.000 | 1.000 | 1.000 |  | 1.000 | 1.000 | 1.000 | 1.000 | 1.000 | 1.000 |
| OTU_6921 | 1.000 | 1.000 | 1.000 | 1.000 | 1.000 | 1.000 |  | 1.000 | 1.000 | 1.000 | 1.000 | 1.000 | 1.000 |  | 1.000 | 1.000 | 1.000 | 1.000 | 1.000 | 1.000 |
| OTU_741 | 1.000 | **0.028** | 1.000 | **< 0.001** | 1.000 | **0.010** |  | 1.000 | 0.090 | 1.000 | **0.029** | 1.000 | 0.103 |  | 1.000 | 1.000 | 1.000 | 0.089 | 1.000 | 0.578 |
