# Supplementary figures and images for: Bacterial Preferences for Specific Soil Particle Size Fractions Revealed by Community Analyses
Source: Front Microbiol. 2018 Feb 23;9:149. doi: 10.3389/fmicb.2018.00149 (PMC5829042; doi:10.3389/fmicb.2018.00149)

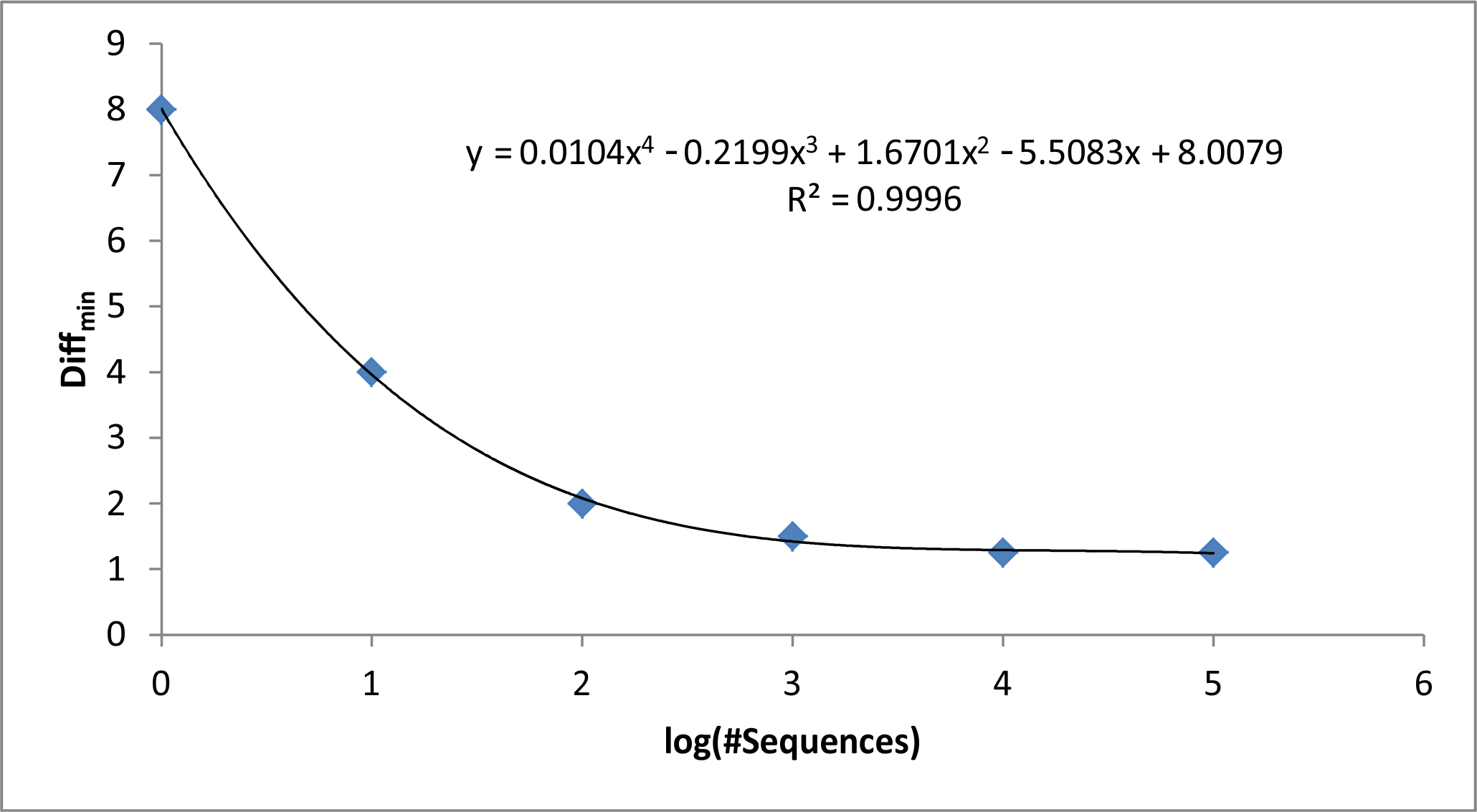

Supplement: Supplementary file 15 [file Image1.TIF]

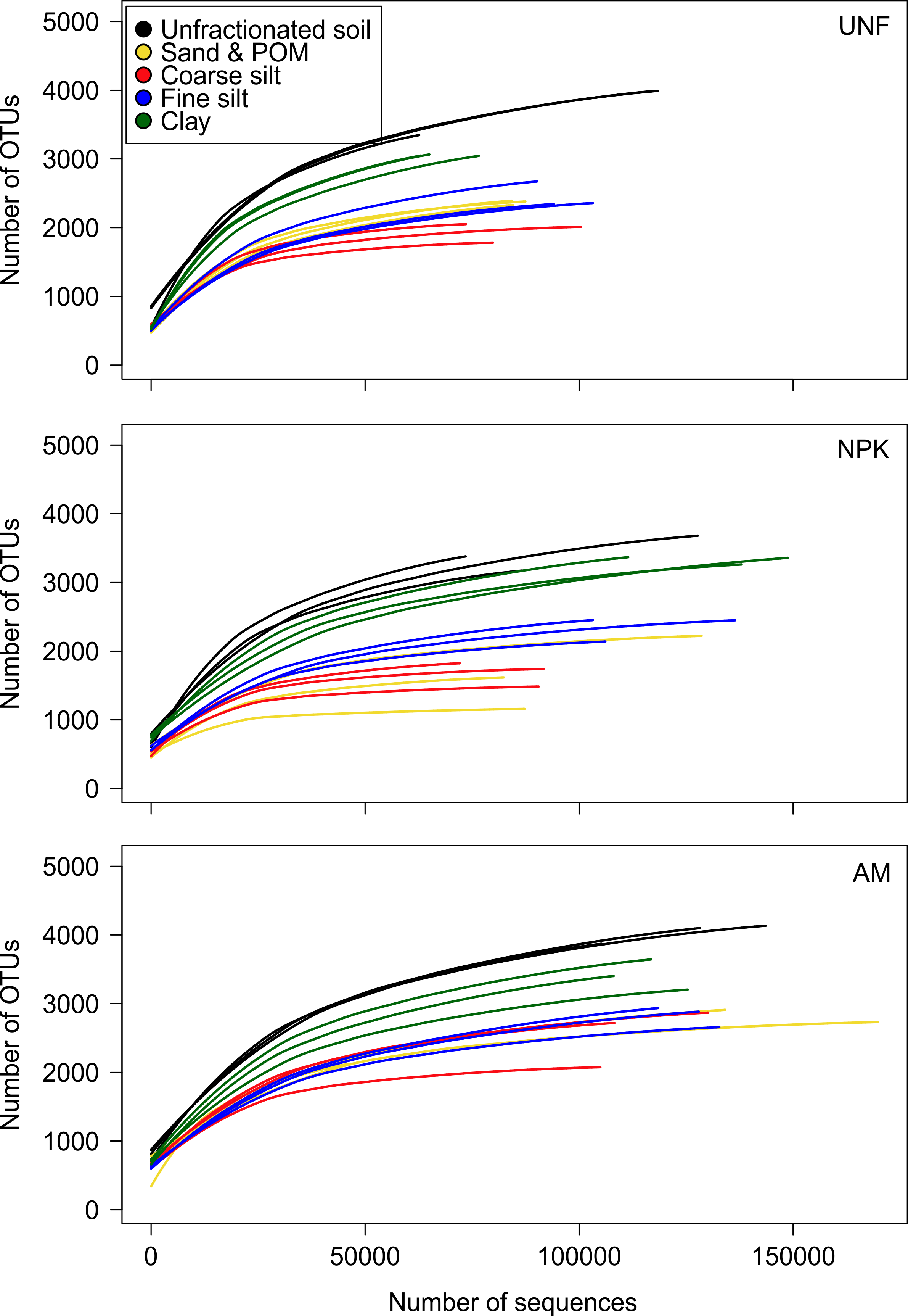

Supplement: Supplementary file 16 [file Image2.TIF]

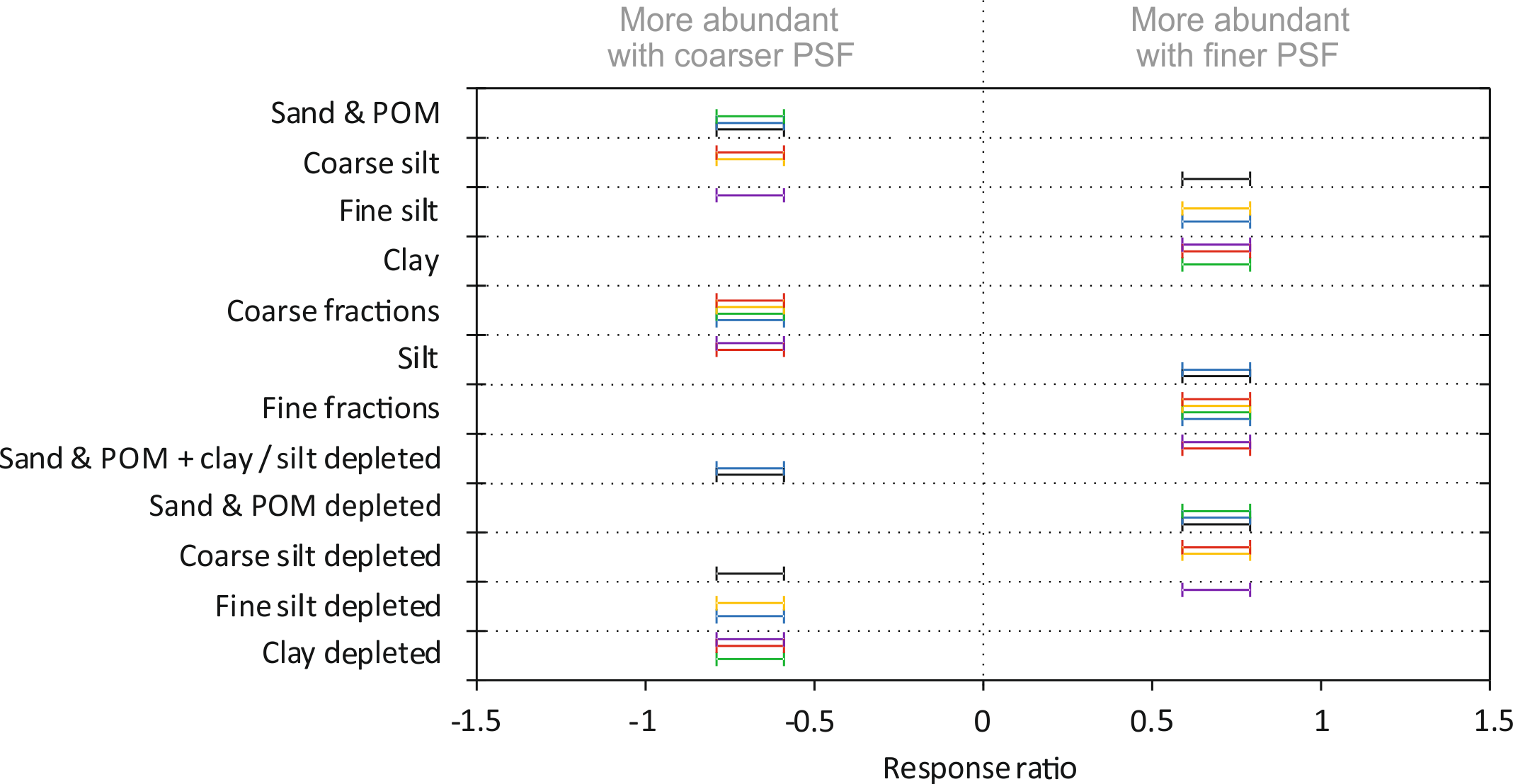

Supplement: Supplementary file 17 [file Image3.tif]

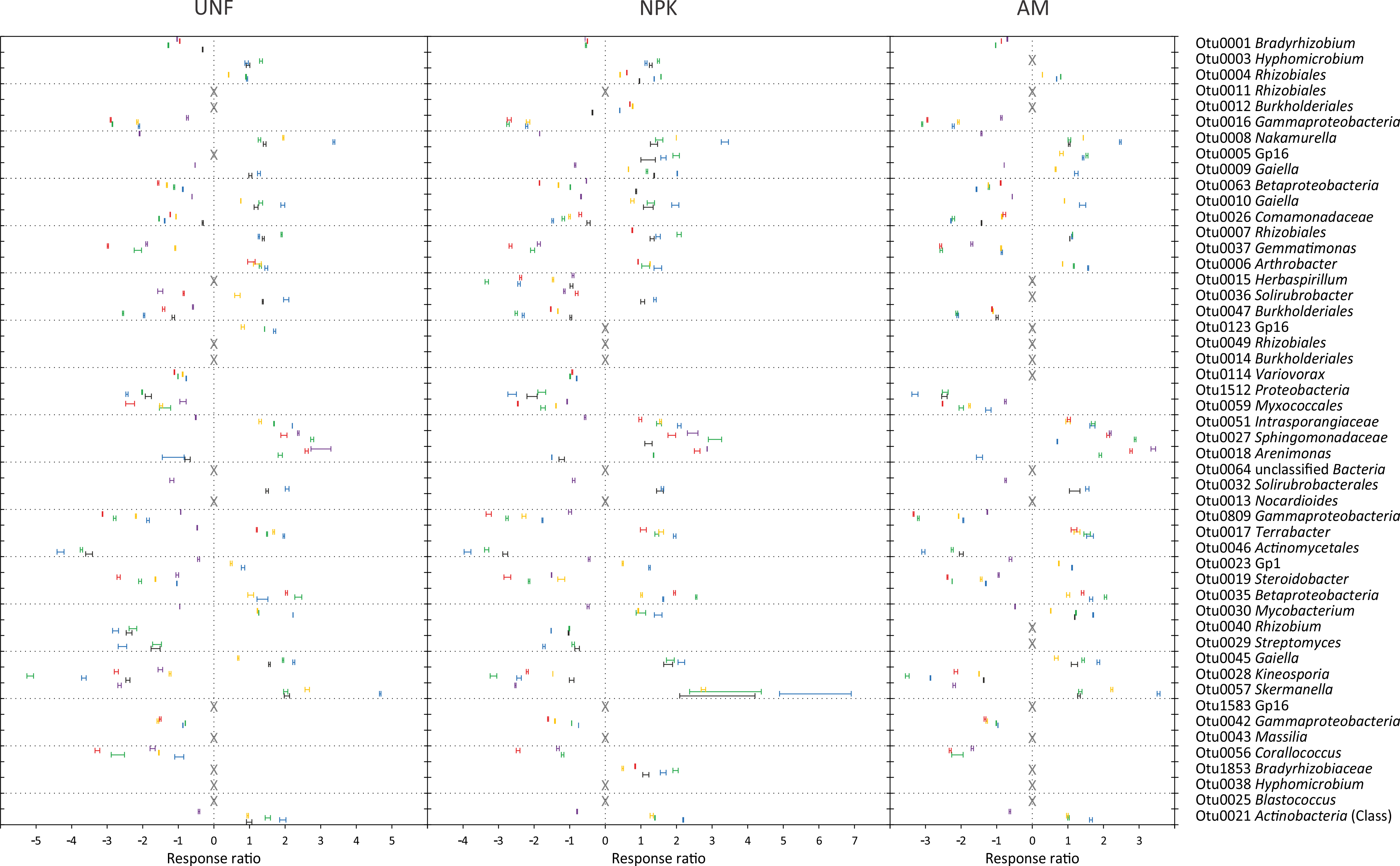

Supplement: Supplementary file 18 [file Image4.TIF]

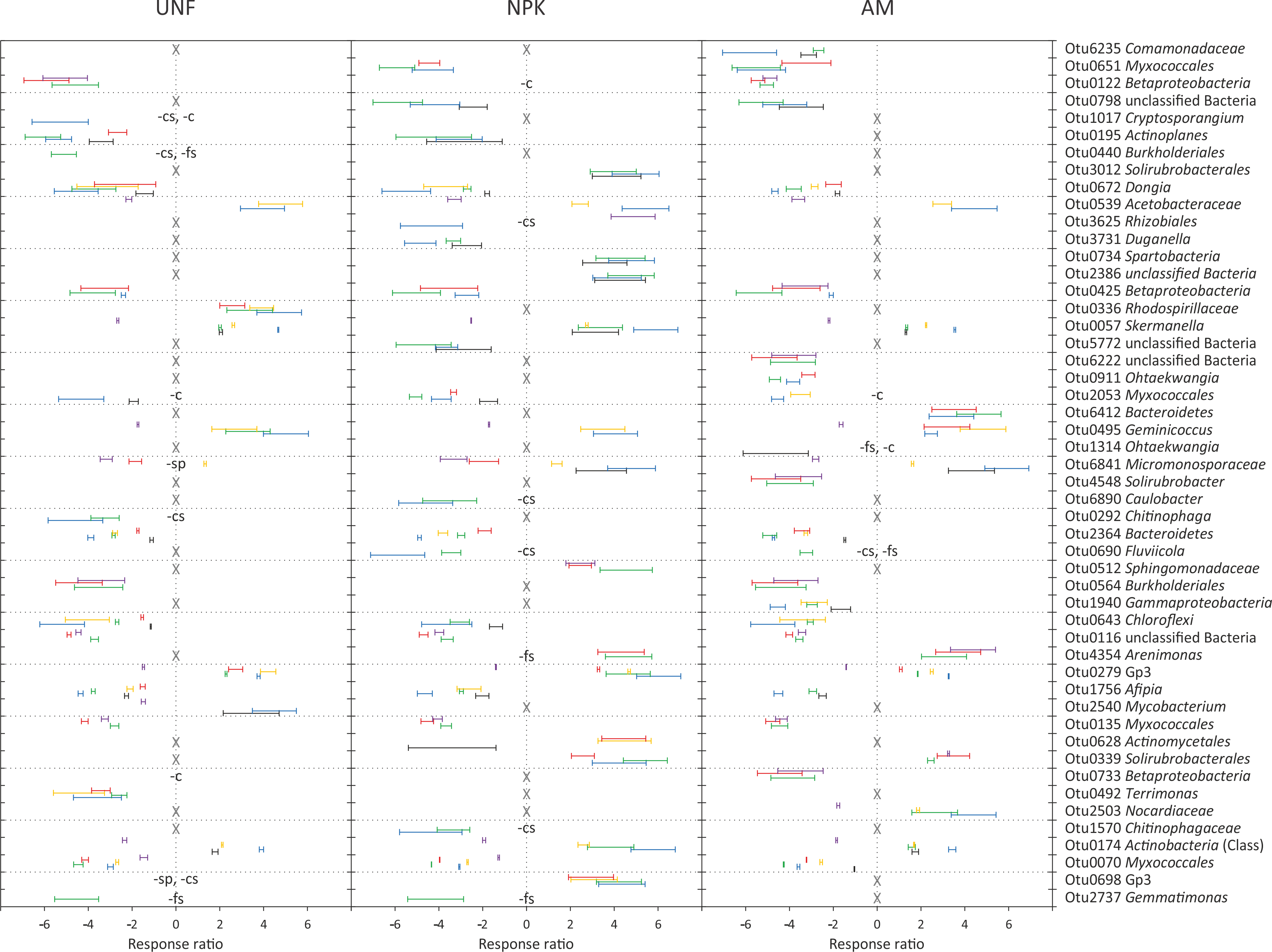

Supplement: Supplementary file 19 [file Image5.TIF]

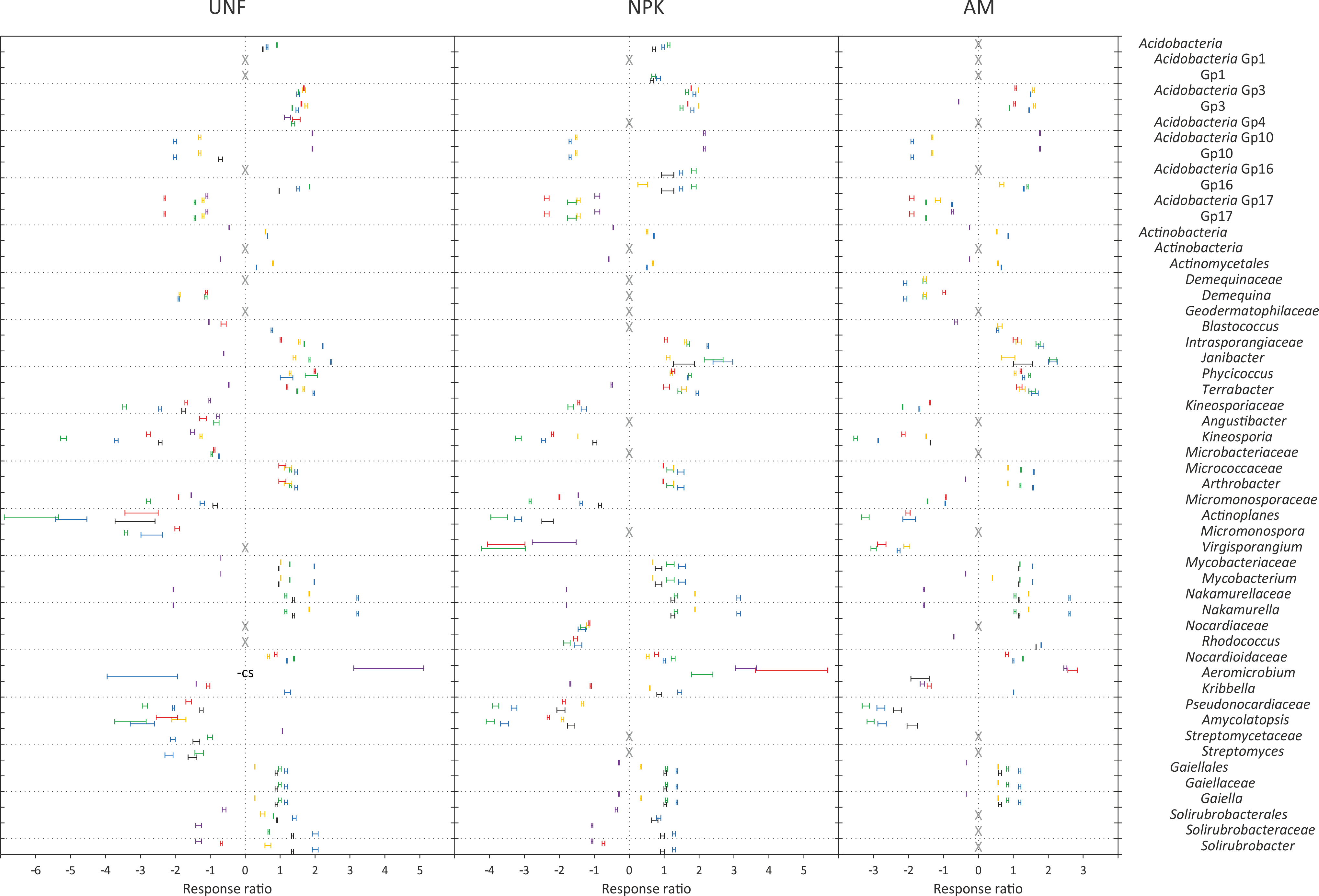

Supplement: Supplementary file 20 [file Image6.TIF]

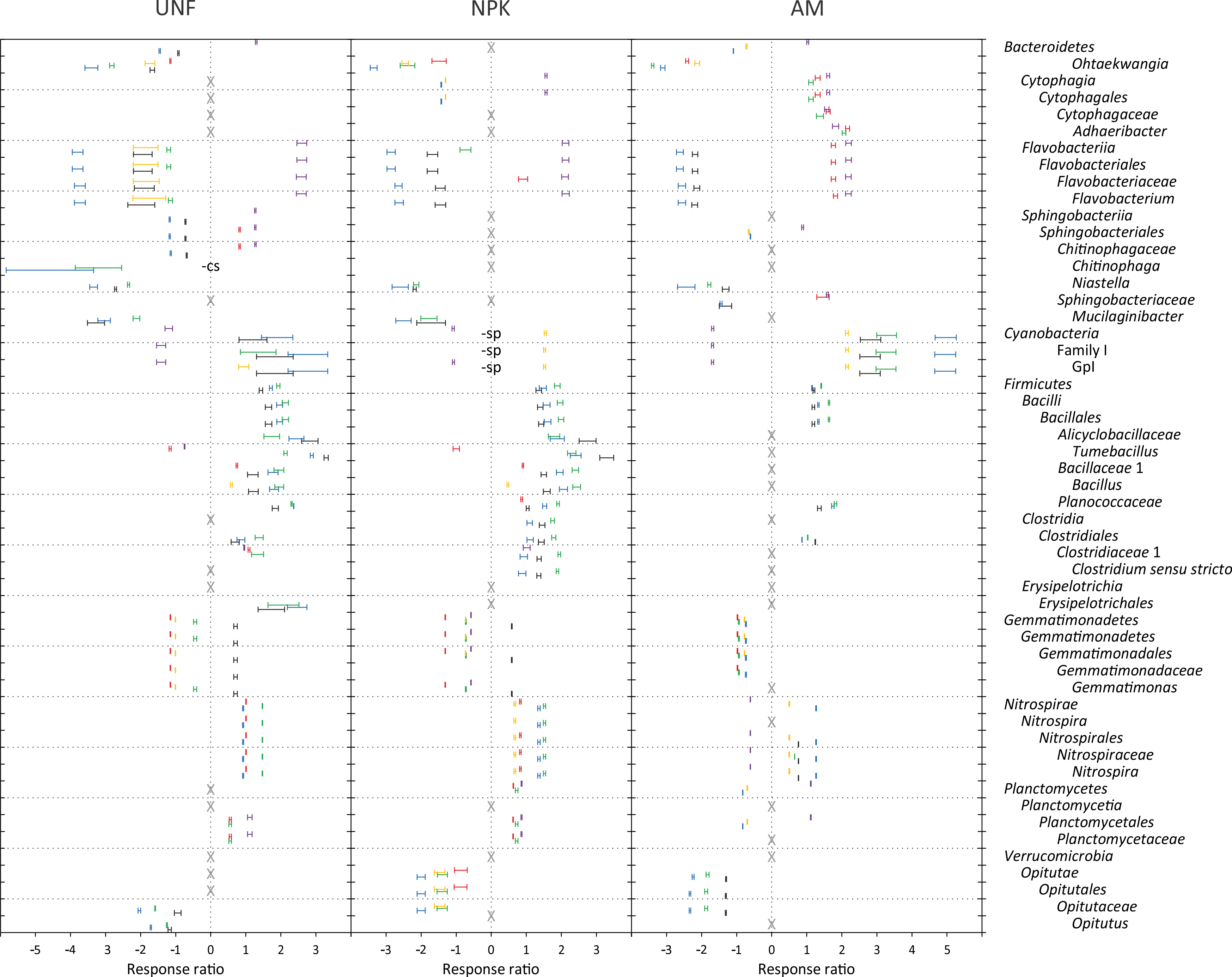

Supplement: Supplementary file 21 [file Image7.TIF]

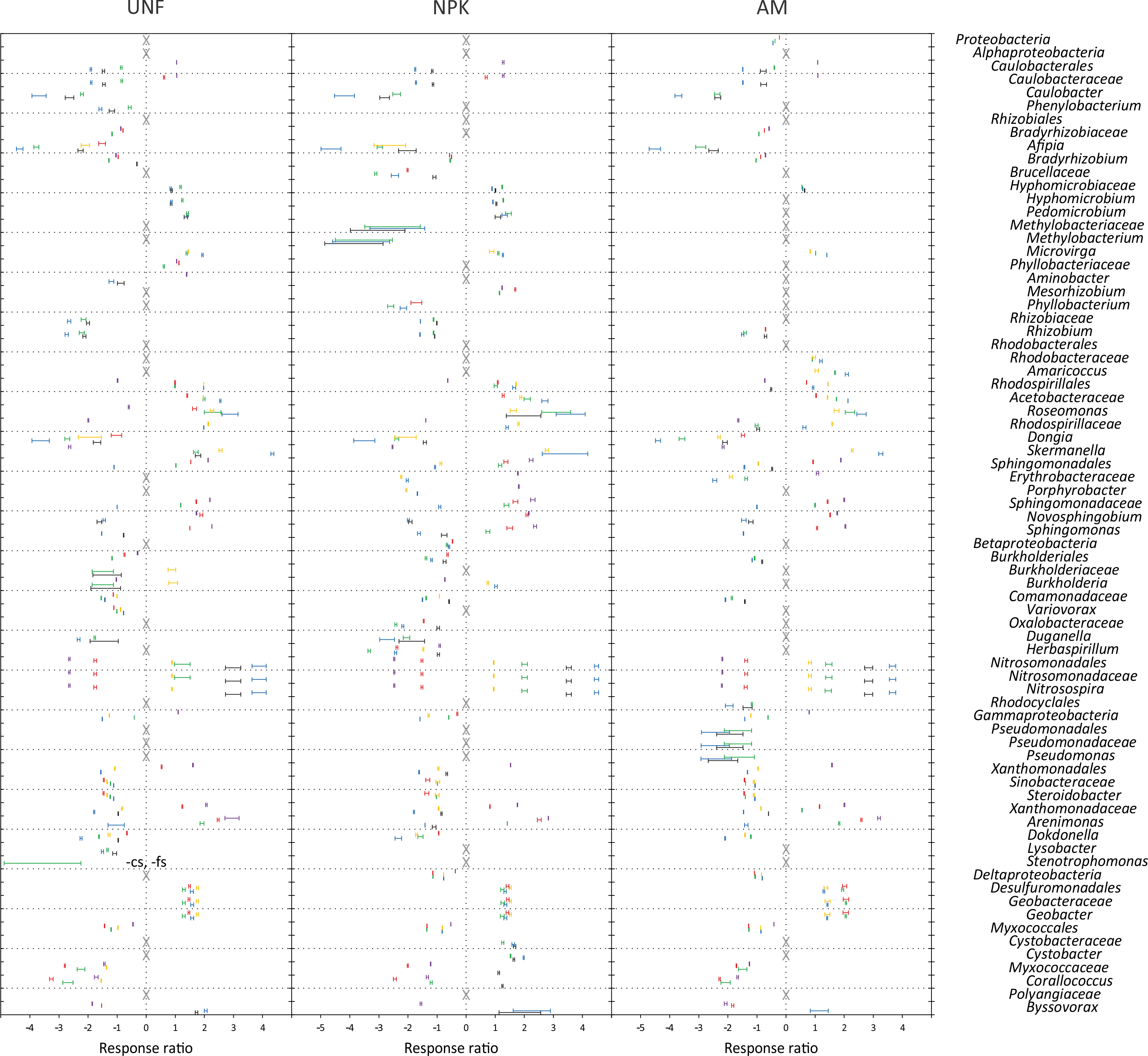

Supplement: Supplementary file 22 [file Image8.TIF]

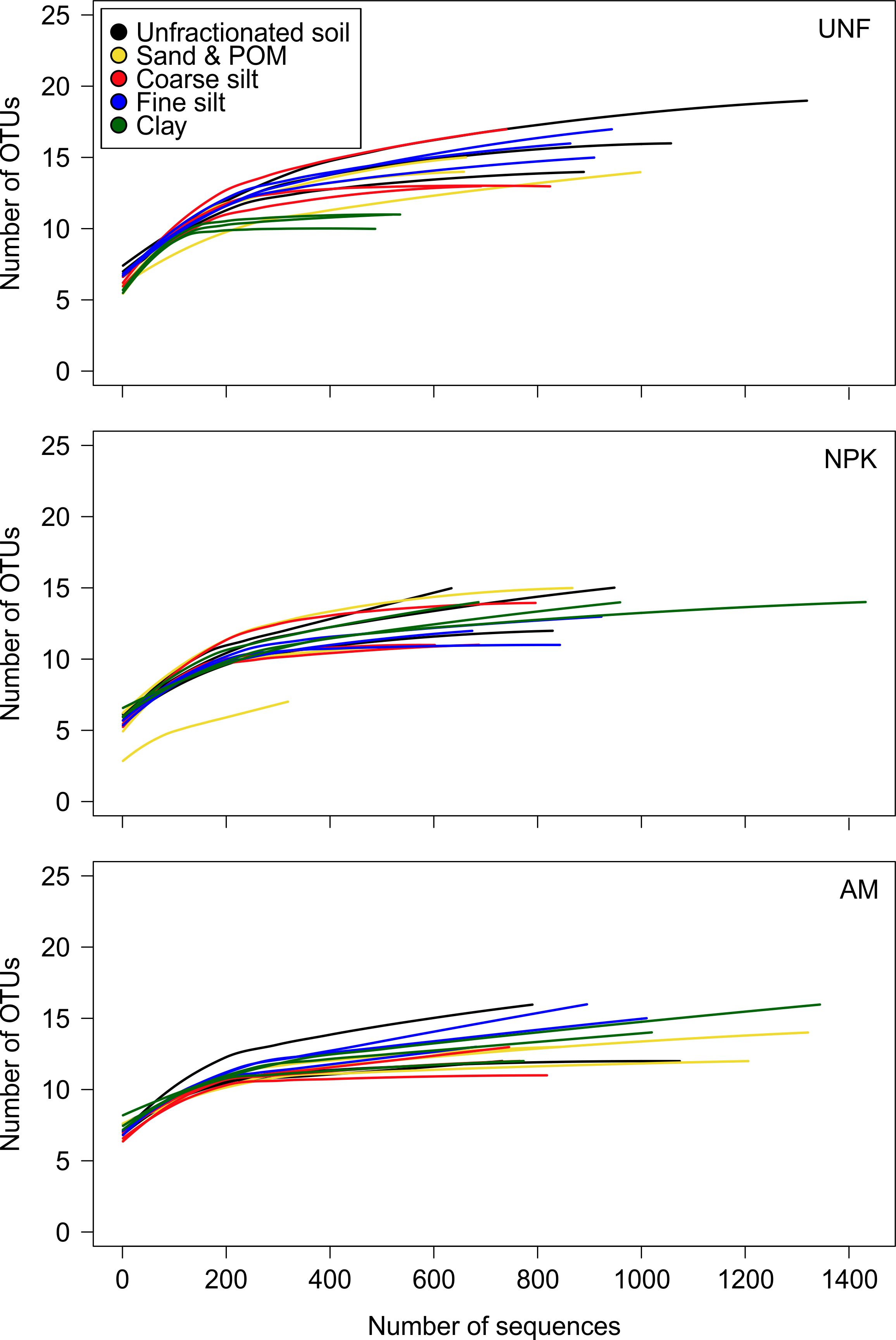

Supplement: Supplementary file 23 [file Image9.TIF]

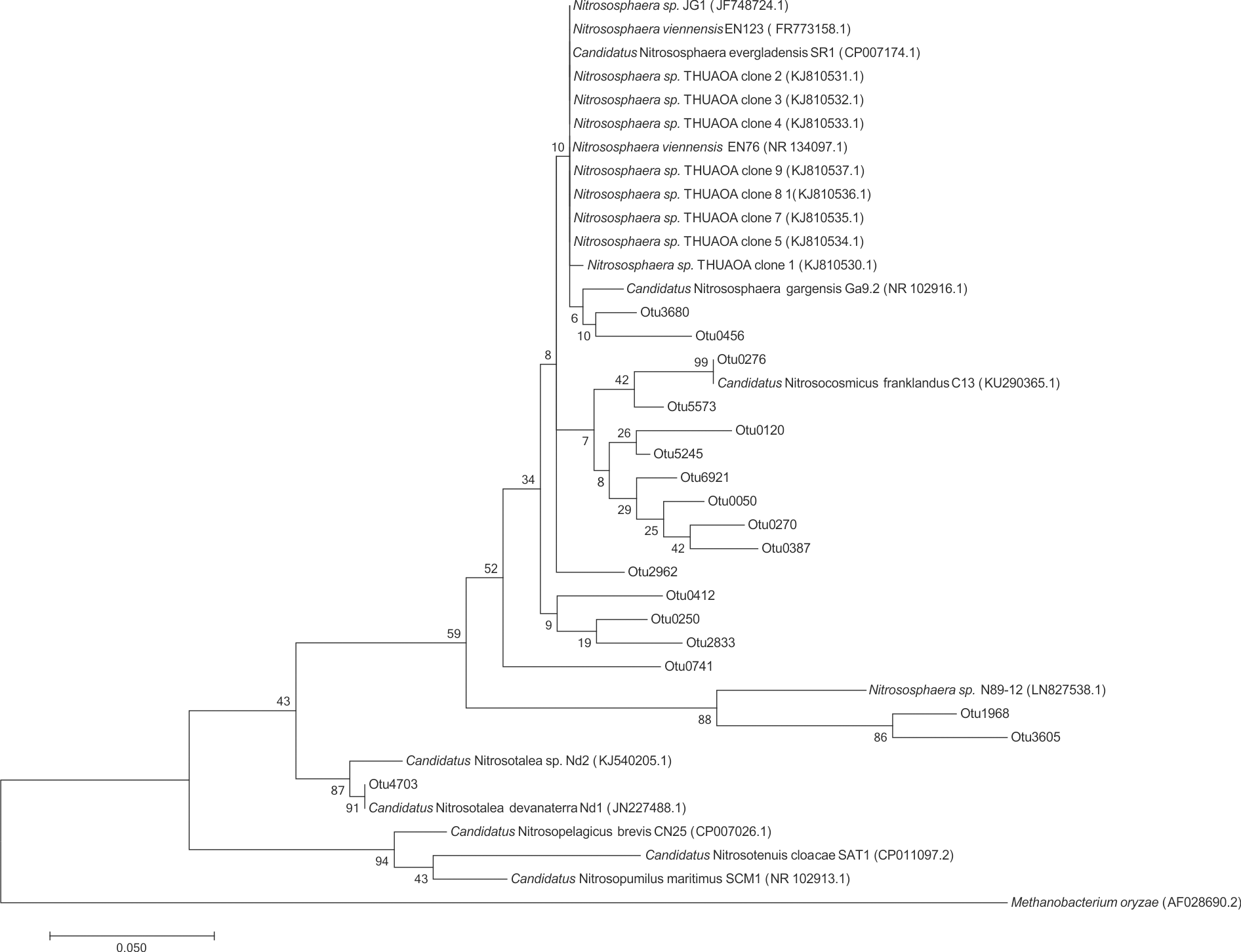

Supplement: Supplementary file 24 [file Image10.TIF]
