# Supplementary material for: Bacterial Preferences for Specific Soil Particle Size Fractions Revealed by Community Analyses
Source: Front Microbiol. 2018 Feb 23;9:149. doi: 10.3389/fmicb.2018.00149 (PMC5829042; doi:10.3389/fmicb.2018.00149)
Supplement: Supplementary file 25 [file DataSheet1.DOCX]

**Supplementary Material Figure legends**

Fig. S1 Equation to estimate the minimal meaningful difference (Diff_min_) between two synusiae based on the larger ones’ number of sequences being valid for up to 60,635 sequences

Fig. S2 Rarefaction curves of bacterial sequences

Fig. S3 Examples of response ratio results for interpreting PSF preferences as given in Figs. S4–S6. For explanation see Section S1

Fig. S4 Response ratios regarding preferences for particle size fractions of the 50 most abundant OTUs. For interpretation see Section S1

Fig. S5 Response ratios of the 50 OTUs showing the strongest preferences as averaged over the three soil variants. Particle size fractions where the taxa were significantly absent are indicated by “-sp” for sand and particulate organic matter, “-cs” for coarse silt, “-fs” for fine silt, and “-c” for clay. For interpretation see Section S1

Fig. S6 Response ratios of taxa above OTU-level indicating significant differences between particle size fractions. Particle size fractions where the taxa were significantly absent are indicated by “-sp” for sand and particulate organic matter, “-cs” for coarse silt, and “-fs” for fine silt. For interpretation see Section S1

Fig. S7 Fig. S6 continued

Fig. S8 Figs. S6–S7 continued

Fig. S9 Rarefaction curves of archaeal sequences

Fig. S10 Maximum Likelihood-tree of OTUs identified as *Nitrososphaera* based on the Kimura 2-parameter model with accession numbers of reference sequences in parentheses and bootstrap values at nodes
