# Supplementary material for: Bacterial Preferences for Specific Soil Particle Size Fractions Revealed by Community Analyses
Source: Front Microbiol. 2018 Feb 23;9:149. doi: 10.3389/fmicb.2018.00149 (PMC5829042; doi:10.3389/fmicb.2018.00149)
Supplement: Supplementary file 26 [file DataSheet2.docx]

**SUPPLEMENTARY MATERIAL**

**S1. Introduction and method regarding the statistical graphics (Figs. S4 – S8) given in the Supplementary Material**

The objective of this study was to detect and characterise preferences of bacterial taxa for soil particle size fractions (PSFs), i.e. sand & particulate organic matter (POM), coarse silt, fine silt, and clay. The relative abundances at each of these PSFs were compared pairwise as described in the Materials and Methods section of the main text. In total there were six comparisons with the statistical significance differences modified by meaningful differences. Three significant differences were set as the minimum to detect “preference”. In the main text, taxa showing any preference or depletion are displayed with the relative abundance of the most abundant population set to 1, and the other populations given as fractions of this most abundant one. This, however, is simplified as it does not show which PSF significantly differ from each other and how large the respective errors are.

In Figures S4–S8 only those of the six comparisons with statistically significant and meaningful differences are shown. Taxa with less than three significant comparisons are indicated by a grey “X” or are missing at all. The differences and their errors are given as response ratios (Hedges et al., 1999), i.e. they are scaled by natural logarithm. The ratios are clay to fine silt (given in purple), clay to coarse silt (red), fine silt to coarse silt (gold), clay to sand/POM (green), fine silt to sand/POM (blue), and coarse silt to sand/POM (black). This ratio-pattern of finer to coarser PSFs leads to positive ratios when the abundance is higher at the finer fraction and vice versa (Fig. S3). The pattern of the values, either positive or negative, for the comparisons allows interpreting the data. Figure S3 gives examples which patterns indicate the simplest preferences or depletions. However, the proper results (Figs. S4–S8) also contain more complex patterns indicating the sequence of preference.

These graphics were prepared using Excel 2010 (Version 14.0.7165.5000, Microsoft, Redmond, WA) and joined and edited using CorelDRAW X8 (Version 18.0.0.448, Corel Corporation, Ottawa, Canada).

Reference:

Hedges, L. V., Gurevitch, J., Curtis, P. S. (1999). The meta-analysis of response ratios in experimental ecology. *Ecology* 80, 1150-1156. doi: 10.2307/177062
